# The inherent community structure of hyperbolic networks

**DOI:** 10.1038/s41598-021-93921-2

**Published:** 2021-08-06

**Authors:** Bianka Kovács, Gergely Palla

**Affiliations:** 1 Department of Biological Physics, Eötvös Loránd University, Pázmány P. stny. 1/A, Budapest, 1117 Hungary; 2 MTA-ELTE Statistical and Biological Physics Research Group, Pázmány P. stny. 1/A, Budapest, 1117 Hungary; 3 Health Services Management Training Centre, Semmelweis University, Kútvölgyi út 2, Budapest, 1125 Hungary

**Keywords:** Complex networks, Statistical physics

## Abstract

A remarkable approach for grasping the relevant statistical features of real networks with the help of random graphs is offered by hyperbolic models, centred around the idea of placing nodes in a low-dimensional hyperbolic space, and connecting node pairs with a probability depending on the hyperbolic distance. It is widely appreciated that these models can generate random graphs that are small-world, highly clustered and scale-free at the same time; thus, reproducing the most fundamental common features of real networks. In the present work, we focus on a less well-known property of the popularity-similarity optimisation model and the ${\mathbb {S}}^1/{\mathbb {H}}^2$ model from this model family, namely that the networks generated by these approaches also contain communities for a wide range of the parameters, which was certainly not an intention at the design of the models. We extracted the communities from the studied networks using well-established community finding methods such as Louvain, Infomap and label propagation. The observed high modularity values indicate that the community structure can become very pronounced under certain conditions. In addition, the modules found by the different algorithms show good consistency, implying that these are indeed relevant and apparent structural units. Since the appearance of communities is rather common in networks representing real systems as well, this feature of hyperbolic models makes them even more suitable for describing real networks than thought before.

## Introduction

Complex network theory is a rapidly expanding interdisciplinary field, strongly interwoven with statistical physics, concentrating on the interesting non-trivial statistical features of the graphs representing the connections/interactions between entities of complex systems^1–3^. Over the last two decades, the vast number of studies of real networks have shown that some of these features seem to be almost universal, such as the small-world property^4,5^, the relatively high clustering coefficient^6^, the inhomogeneous degree distribution^7,8^, and the presence of communities^9–11^. Grasping these properties in a unified modelling framework is a non-trivial problem; however, a very notable approach pointing in this direction is given by hyperbolic network models^12–18^ centred around the idea of placing nodes on a hyperbolic plane, and drawing links with a probability depending on the metric distance.

Probably the most well-known model from this family is the popularity-similarity optimisation (PSO) model^12^, working in the native disk representation of the two-dimensional hyperbolic space. Here the nodes are introduced one by one with logarithmically increasing radial coordinates and uniformly random angular coordinates, and the newly appearing nodes connect to the previous ones with a probability decreasing with the hyperbolic distance. This model is known to be capable of generating networks that are small-world, highly clustered and scale-free at the same time. Roughly speaking, the degree of the nodes is determined by their radial coordinate—with the inner nodes becoming eventually hubs—and due to a parameter-controlled outward shift of the nodes (corresponding to popularity fading), the decay exponent of the degree distribution is also tuneable in the model. By changing the cutoff of the connection probability as a function of the hyperbolic distance with another parameter called the temperature, the clustering coefficient of the resulting random graphs can be adjusted as well.

Another remarkable hyperbolic network model, capable of generating small-world, highly clustered and scale-free random graphs is given by the ${\mathbb {S}}^1/{\mathbb {H}}^2$ model^17,19^. In the ${\mathbb {S}}^1$ model nodes are placed on a circle and are given a hidden variable drawn from a power-law distribution. Here the connection probability depends on the angular distance between the nodes and the hidden variables. By converting the hidden variables to radial coordinates in the native disk representation of the hyperbolic plane, we arrive to the equivalent ${\mathbb {H}}^2$ model, where the connection probabilities depend on the hyperbolic distance between the nodes in a similar way as in the PSO model.

In parallel with the success of hyperbolic models, there have also been several studies carried out focusing on possible hidden metric spaces behind real networks, starting with the examination of the self-similarity of scale-free networks^17^, followed by reports on the hyperbolicity of protein interaction networks^20,21^, the Internet^22–24^, brain networks^25^, or the world trade network^26^. Furthermore, a connection between the navigability of networks and hyperbolic spaces was shown^22,27^, the geometric nature of weights^28^ and clustering^29,30^ was demonstrated, methods for measuring the hyperbolicity of networks were introduced^31^. Hyperbolic networks are also closely related to network models based on simplicial complexes^32,33^, where the emergent geometry of the generated random graphs was shown to be hyperbolic. In addition, significant achievements were obtained related to the problem of hyperbolic embedding as well^13,19,23,34–36^, where the task is to find the most suitable node coordinates in a hyperbolic space given an input network topology.

Returning to hyperbolic network models, in the recent years there have also been efforts devoted to the development of generative methods capable of producing hyperbolic random graphs with an apparent community structure^14–16,18^. Clusters or communities in hyperbolic networks usually correspond to separated angular regions^37–42^. In accordance with this, in Refs.^15,16^ the uniform angular distribution of the nodes was replaced by a multimodal distribution, where communities arise naturally at the peaks. The appearance of communities in Refs.^14,18^ was achieved by applying a geometric preferential attachment process, also inducing the formation of denser angular regions corresponding to communities.

Although the above-mentioned ideas do provide very interesting models with ’built-in’ community formation, in the present paper we would like to draw the attention to the lesser-known but somewhat surprising fact that angular inhomogeneity is not a necessary condition for the presence of communities in hyperbolic network models, and that communities can appear in networks generated by the ’plain’ PSO model or the ${\mathbb {S}}^1/{\mathbb {H}}^2$ model as well. This was first shown for the E-PSO model (a generalisation of the PSO model^13^) in Refs.^37,39^ and for the ${\mathbb {S}}^1/{\mathbb {H}}^2$ model in Ref.^38^, along with the proposition of the “Community-Sector hypothesis”, supposing that most members of a community gather in the same angular sector on the hyperbolic plane. In the closely related study of Ref.^40^, the dependence of the modularity (a commonly used quality score for communities introduced in Ref.^43^) on the temperature parameter $T\in [0,1)$ of the E-PSO model (controlling the clustering coefficient) for communities found by the Louvain method^44^ was also studied to some extent. According to the results, the modularity can be even above 0.7 when *T* is low, and gradually decreases when *T* is increased; however, can still stay above 0.3 when *T* approaches 1. In parallel with these studies, in Ref.^45^ the analogy between the hyperbolic embedding and the community structure was studied mostly for real networks and partly for synthetic graphs generated by the PSO model, where again, the PSO networks were observed to have a notable community structure, just like the real networks.

Even though the above results already provide important signs related to the presence of communities in hyperbolic networks with homogeneous angular node distribution, here we revisit this phenomenon in a detailed in-depth study, motivated by the following. First of all, in spite that a modularity value above 0.3 can be a good community indicator in practice^46^, it is important to note that a high modularity value alone is not always accompanied by a true modular structure, as e.g. Erdős–Rényi random graphs^47^ or scale-free networks obtained with the Barabási–Albert model^48^ can also yield modularity values above 0.8 under certain circumstances^49,50^. Thus, in order to have a truly solid claim about the presence of communities in random graph models without any explicit community formation mechanism, it is best to back up the large modularity values with further analysis of the supposed modular structure from multiple aspects.

Another task of high importance is the more detailed exploration of the parameter space. Apart from simple parameters such as the network size and the average degree, both the PSO model and the ${\mathbb {S}}^1/{\mathbb {H}}^2$ model have basically two parameters: one controlling the decay exponent $\gamma$ of the scale-free degree distribution and the other controlling the clustering coefficient. By analysing the effect of these parameters on the communities, we can gain a clear picture about what sort of modular structure can be expected when the aim is to generate a hyperbolic random graph with specified $\gamma$ and clustering coefficient values.

Along this line, here we generate random graphs according to the PSO and the ${\mathbb {S}}^1/{\mathbb {H}}^2$ models in a wide range of parameter settings and examine their community structure with the help of three well-established community finding algorithms given by the Louvain method^44^, the Infomap algorithm^51^ and asynchronous label propagation^52^. The Louvain approach is known to be a very efficient modularity maximising method, while the other two algorithms included do not build on the modularity and extract the modular structure of the studied networks based on different concepts. By applying independent community finding methods, the comparison between the found modules can reveal whether they correspond to strong, significant structures that can be located consistently in several different ways or not. In order to gain a quantitative comparison between the communities found by the different methods, we rely on the concept of the adjusted mutual information (AMI)^53^, a well-known information-theoretic similarity measure. Besides the modularity, we also examine the angular separation index (ASI) of the communities^42^ corresponding to a measure developed specifically for hyperbolic networks, characterising the angular mixing of the groups of nodes (communities) on the native disk.

The paper is organised as follows. In “Methods and preliminaries” section we describe the PSO and ${\mathbb {S}}^1/{\mathbb {H}}^2$ models used for network generation, together with a short summary of the applied community finding methods and the quality measures used for evaluating the detected community structures. This is followed by the details of our analyses in “Results” section, whereas we discuss the implications of our findings in “Discussion and conclusions” section.

## Methods and preliminaries

We begin the description of the used methods with a brief introduction to hyperbolic network models, including both the PSO model and the ${\mathbb {S}}^1/{\mathbb {H}}^2$ model. Then we continue with summarising the applied community-related measures: the concept of modularity, the angular separation index and the adjusted mutual information. We close the section with the description of the used community finding algorithms, given by asynchronous label propagation, the Louvain algorithm and the Infomap method.

### Hyperbolic network models

When studying the underlying hyperbolic geometry of complex networks, commonly the native representation of the two-dimensional hyperbolic space is used^54^, in which the hyperbolic plane of constant curvature $K<0$ is represented by a disk of infinite radius in the Euclidean plane (for which $K=0$). In this representation the Euclidean angles between hyperbolic lines are equal to their hyperbolic values, and the radial coordinate *r* of a point (defined as its Euclidean distance from the disk centre) is equal to its hyperbolic distance from the disk centre. The hyperbolic distance between two points is measured along their connecting hyperbolic line, which is either the arc of the Euclidean circle going through the given points and intersecting the disk’s boundary perpendicularly or—if the disk centre falls on the Euclidean line connecting the two points in question—the corresponding diameter of the disk. The hyperbolic distance *x* between two points at polar coordinates $(r,\theta )$ and $(r',\theta ')$ fulfills the hyperbolic law of cosines written as

1 $$\begin{aligned} \mathrm {cosh}(\zeta x)=\mathrm {cosh}(\zeta r)\,\mathrm {cosh}(\zeta r')-\mathrm {sinh}(\zeta r)\,\mathrm {sinh}(\zeta r')\,\mathrm {cos}(\Delta \theta ), \end{aligned}$$

where $\zeta =\sqrt{-K}$ and $\Delta \theta =\pi -|\pi -|\theta -\theta '||$ is the angle between the examined points. According to Ref.^54^, for $2\cdot \sqrt{e^{-2\zeta r}+e^{-2\zeta r'}}<\Delta \theta$ and sufficiently large $\zeta r$ and $\zeta r'$, the hyperbolic distance can be approximated as

2 $$\begin{aligned} x\approx r+r'+\frac{2}{\zeta }\cdot \ln \left( \frac{\Delta \theta }{2}\right) . \end{aligned}$$

#### The PSO model for network generation

In the popularity–similarity optimisation model, nodes are placed one by one in the above-described native disk representation of the hyperbolic plane and connected with probabilities depending on the hyperbolic distance. The parameters of the model can be listed as follows:

- The curvature $K<0$ of the hyperbolic plane, controlled by $\zeta =\sqrt{-K}>0$. Changing the value of $\zeta$ corresponds to a simple rescaling of the hyperbolic distances; the usual custom is to set the value of $\zeta$ to 1 (i.e. set *K* to $-1$).
- The final number of nodes $N\in {\mathbb {Z}}^+$ in the network.
- The number of connections $m\in {\mathbb {Z}}^+$ established by the newly appearing nodes, corresponding to the half of the average degree $\langle k\rangle$. (The first *m* nodes of the network form a complete graph).
- The popularity fading parameter $\beta \in (0,1]$, controlling the outward drift of the nodes on the native disk. The exponent $\gamma$ of the power-law decaying tail of the degree distribution is related to the popularity fading parameter as $\gamma =1+1/\beta$.
- The temperature $T\in [0,1)$, controlling the average clustering of the network, where a lower temperature results in a higher average clustering coefficient.

During the random graph generation process, initially the network is empty, and at each time step $i=1,2,...,N$ a new node joins the network as follows:

1. The new node *i* appears at polar coordinates $(r_{ii},\theta _i)$, where the radial coordinate $r_{ii}$ is set to $\frac{2}{\zeta }\mathrm {ln}(i)$ and the angular coordinate $\theta _i$ is sampled from $[0,2\pi )$ uniformly at random.
2. The radial coordinate of each previously (at time $j<i$) appeared node *j* is increased according to the formula $r_{ji}=\beta r_{jj}+(1-\beta )r_{ii}$ in order to simulate popularity fading.
3. The new node *i* establishes connections with previously appeared nodes. Only single links are permitted.
  - If the number of previously appeared nodes is not larger than *m*, node *i* connects to all of them.
  - Otherwise, the new node *i* connects to *m* of the previously appeared nodes, where the connection probabilities are determined by the hyperbolic distances between the node pairs, which can be calculated based on Eq. (1). If $T=0$, node *i* simply connects to the *m* hyperbolically closest nodes, whereas at temperatures $T>0$, any previous node $j=1,2,...,i-1$ gets connected to node *i* with probability
    3 $$\begin{aligned} p(x_{ij})=\frac{1}{1+e^{\frac{\zeta }{2T}(x_{ij}-R_i)}}, \end{aligned}$$
    where the cutoff distance $R_i$ is set to
    4 $$\begin{aligned} R_i =\left\{ \begin{array}{ll} r_{ii}-\frac{2}{\zeta }\mathrm {ln}\left( \frac{2T}{\mathrm {sin}(T\pi )}\cdot \frac{1-e^{-\frac{\zeta }{2}(1-\beta )r_{ii}}}{m(1-\beta )}\right) &{} \text{ if }\; \beta <1, \\ r_{ii}-\frac{2}{\zeta }\mathrm {ln}\left( \frac{T}{\mathrm {sin}(T\pi )}\cdot \frac{\zeta r_{ii}}{m}\right) &{} \text{ if }\; \beta =1, \end{array} \right. \end{aligned}$$
    ensuring that the expected number of nodes connecting to the new node *i* at its arrival is equal to *m*.

#### The ${\mathbb {S}}^1/{\mathbb {H}}^2$ model for network generation

In the ${\mathbb {S}}^1$ model^17^, first the *N* number of nodes are placed on a one-dimensional sphere (i.e. a circle) and each is given a hidden variable $\kappa _i\in [\kappa _0,\infty ),\,i=1,2,...,N$. Then, each pair of nodes becomes connected with a probability taking into account both the angular distance and the hidden variables. In the below-described algorithm^19^, $\kappa _i$ corresponds to the expected degree ${\bar{k}}_i$ of node *i* in the thermodynamic limit. Thus, the connection rule can be phrased in a simple, intuitive way, namely the nodes that are closer in the hidden metric space underlying the network are more likely to be connected, but in the meantime nodes with higher degree obtain farther-reaching connections as well. In the equivalent ${\mathbb {H}}^2$ model^19^, the hidden variable $\kappa _i$ is converted into the radial coordinate $r_i$ in the native representation of the hyperbolic plane, and the connection probability depends on the hyperbolic distance between the nodes that expresses the effect of both the similarity and the node degrees (the popularity).

The parameters of these models can be listed as follows:

- The total number of nodes *N*.
- The average degree $\langle k\rangle$.
- The exponent $\gamma$ of the tail of the degree distribution following a power law of the form $P(k)\sim k^{-\gamma }$. Although these models can accommodate any degree distribution in general, here we use only power laws with $\gamma >2$ values, in order to generate networks having similar properties as in case of the PSO model.
- The parameter $1<\alpha$, controlling the average clustering coefficient $\langle c\rangle$ of the generated network ($\lim \nolimits _{\alpha \rightarrow 1}\langle c\rangle =0$).

In the ${\mathbb {S}}^1$ model, a network of *N* number of nodes—each of them indexed by $i\in [1,N]$—is generated through the following steps:

1. For each node *i* an angular coordinate $\theta _i$ is sampled from the interval $[0,2\pi )$ uniformly at random.
2. For each node *i* a hidden variable $\kappa _i$ is sampled from the interval $[\kappa _0,\infty )$ according to the distribution $\rho (\kappa )=(\gamma -1)\cdot \frac{\kappa ^{-\gamma }}{\kappa _0^{1-\gamma }}$, where $\kappa _0=\frac{\gamma -2}{\gamma -1}\cdot \langle k\rangle$.
3. Each pair of nodes $i-j$ is connected with probability
  5 $$\begin{aligned} p_{ij}=\frac{1}{1+\left( \frac{N\cdot \Delta \theta _{ij}}{2\pi \cdot \mu \cdot \kappa _i\cdot \kappa _j}\right) ^{\alpha }}, \end{aligned}$$
  where $\Delta \theta _{ij}=\pi -|\pi -|\theta _i-\theta _j||$ is the angular distance between the nodes and $\mu =\frac{\alpha }{2\pi \langle k\rangle }\cdot \sin \left( \frac{\pi }{\alpha }\right)$.

To facilitate a straightforward comparison with the PSO model, we converted the hidden variable associated to the nodes into a radial coordinate in the native representation of the hyperbolic plane (at $K=-1$ curvature) as

6 $$\begin{aligned} r_i={\hat{R}}-2\ln \left( \frac{\kappa _i}{\kappa _0}\right) , \end{aligned}$$

where ${\hat{R}}=2\ln \left( \frac{N}{\mu \pi \kappa _0^2}\right)$. Note that using this hyperbolic representation (i.e. the ${\mathbb {H}}^2$ model) the connection probability (5) becomes $p_{ij}=\left[ 1+e^{\frac{\alpha }{2}\cdot (x_{ij}-{\hat{R}})}\right] ^{-1}$, depending on the hyperbolic distance $x_{ij}$ in the same way as the connection probability in Eq. (3).

### Finding and evaluating communities

Communities (also referred to as modules, cohesive groups, clusters) are frequently occurring structural units in complex networks having usually a larger internal and a smaller external link density, lacking however a widely accepted unique definition. Finding, evaluating and comparing communities are all non-trivial problems, with a vast number of different solutions suggested in the literature^9–11^. Here we first describe the concept of modularity, corresponding to the most widely used measure for quantifying the quality of communities. This is followed by the angular separation index, providing a score specific for hyperbolic networks, measuring the angular intermixing between communities in the hyperbolic disk, and the adjusted mutual information, allowing the quantitative comparison between community partitions found by different methods. In our studies, we have picked three well-grounded, commonly used methods for detecting communities, namely the asynchronous label propagation, the Louvain algorithm, and Infomap.

#### Modularity

Probably the most well-known quality measure for communities is given by the modularity^43^, comparing the observed density of links between the members of the same community with the expected link density based on some random null model, written in general as

7 $$\begin{aligned} Q = \frac{1}{2L}\sum _{i=1}^N\sum _{j=1}^N\left[ A_{ij} -P_{ij}\right] \delta _{c_i,c_j}, \end{aligned}$$

where *N* is the number of nodes in the network, $A_{ij}$ denotes an element the adjacency matrix ($A_{ij}\equiv A_{ji}=1$ if *i* is connected to *j* and otherwise $A_{ij}\equiv A_{ji}=0$), $P_{ij}$ gives the connection probability between nodes *i* and *j* in the null model, *L* stands for the total number of links in the network, $c_i$ is the community to which node *i* belongs and the Kronecker delta $\delta _{c_i,c_j}$ ensures that non-zero contribution can come only from node pairs in the same community. This quality measure can take values in the $Q\in [-1/2,1]$ interval, where larger values of *Q* indicate stronger communities that have a significantly larger internal link density compared to the random expectation.

In practice, a natural choice for the null model is provided by the configuration model, where the connection probability between nodes *i* and *j* can be given with the node degrees $k_i$ and $k_j$ simply as $P_{ij}=\frac{k_ik_j}{2L}$. This form has also been extended to weighted networks^55^, where the number of links *L* is replaced by $M=\frac{1}{2}\cdot \sum \nolimits _{i=1}^N\sum \nolimits _{j=1}^N w_{ij}$ (with $w_{ij}$ denoting the link weight between nodes *i* and *j*), and the node degrees are replaced by the node strengths defined e.g. for node *i* as $s_i = \sum _{\ell =1}^N w_{i\ell }$, resulting in

8 $$\begin{aligned} Q=\frac{1}{2M}\cdot \sum \limits _{i=1}^N\sum \limits _{j=1}^N \left[ w_{ij}-\frac{s_is_j}{2M}\right] \delta _{c_i,c_j}. \end{aligned}$$

In order to take into account the hyperbolic distances along the links, we adopted the practice suggested in Ref.^35^, and used in our community analysis a link weight defined as

9 $$\begin{aligned} w_{ij}\equiv w_{ji}=\frac{1}{1+x_{ij}} \end{aligned}$$

for adjacent nodes *i* and *j*, where the hyperbolic distance $x_{ij}$ was calculated based on Eq. (1) using $\zeta =1$.

#### Angular separation index

In networks embedded into the hyperbolic disk, communities usually occupy well-defined angular regions, having little or no overlap with the region of the other communities^37–42^. A quantitative score characterising this tendency is given by the angular separation index (ASI)^42^. Its basic idea is to compare the number of “mistakes” in the angular arrangement—i.e. the number $o_i$ of nodes belonging to other communities falling between the boundaries of the given module *i*—summed over all the *C* communities of the network with the highest total number of mistakes obtained with the same clustering of the nodes when the angular coordinates are shuffled at random. Formally, the ASI can be expressed as

10 $$\begin{aligned} \mathrm {ASI} = 1-\frac{\sum \nolimits _{i=1}^{C}o_i}{\max \limits _{r}\left( \sum \nolimits _{i=1}^{C}o_i^{(r)}\right) }, \end{aligned}$$

where the maximisation in the denominator is over a fixed number of random shuffles (we used 1000 shuffles, i.e. $r=1,2,...,1000$, as suggested in Ref.^42^). Accordingly, an ASI value close to 1 indicates well-separated clusters with a low intermixing in the angular coordinates of the members, and an ASI value close to 0 is obtained when the angular arrangement of the members of different clusters is random.

#### Adjusted mutual information

In the field of community detection, together with the rapid increase in the number of different algorithms proposed, came the need for well-grounded methods for comparing the results of the different approaches. Since e.g. the number of found communities and the sizes of the modules can show large variations across the different methods, judging the extent of similarity between two community partitions is non-trivial. Given two sets of communities *A* and *B* over the same network, hosting $C_A$ and $C_B$ number of communities each, a well-known information-theoretic similarity measure is offered by the normalised mutual information (NMI)^56,57^, that can be defined based on the mutual information

11 $$\begin{aligned} \mathrm {MI}(A,B) = -\sum _{i=1}^{C_A}\sum _{j=1}^{C_B}\frac{N_{ij}}{N}\ln \left( \frac{N_{ij}N}{N_iN_j}\right) \end{aligned}$$

and the entropies

12 $$\begin{aligned} H(A) = -\sum _{i=1}^{C_A}\frac{N_i}{N}\ln \left( \frac{N_i}{N}\right) , \;\;\; H(B) = -\sum _{j=1}^{C_B}\frac{N_j}{N}\ln \left( \frac{N_j}{N}\right) , \end{aligned}$$

where $N_{ij}$ denotes the number of shared members of communities *i* and *j*, $N_i$ and $N_j$ stand for the number of nodes in the individual communities, and the total number of nodes in the network is given by *N*. There are several different possibilities for normalising the mutual information $\mathrm {MI}(A,B)$, e.g. we can divide it by the maximum, the arithmetic mean or the geometric mean of the entropies *H*(*A*) and *H*(*B*)^53^. In the present study we used the maximum of the entropies; thus, throughout the paper

13 $$\begin{aligned} \mathrm {NMI}(A,B)\equiv \frac{ \mathrm {MI}(A,B)}{\max \left[ H(A),H(B)\right] }. \end{aligned}$$

This quantity becomes 1 if and only if the partitions *A* and *B* are identical, otherwise its value is lower than 1.

The concept of adjusted mutual information (AMI) supplements this consistent upper bound with a consistent zero expectation corresponding to the similarity we can expect by random chance^53,58^. To achieve this, the average mutual information of random partitions $A'$ and $B'$ is subtracted from the nominator, and the average maximum entropy of random partitions is subtracted from the denominator yielding

14 $$\begin{aligned} \mathrm {AMI}(A,B)=\frac{\mathrm {MI}(A,B)-\langle \mathrm {MI}(A',B')\rangle _{\mathrm{rand}}}{\max \left[ H(A),H(B)\right] -\langle \max \left[ H(A'),H(B')\right] \rangle _{\mathrm{rand}}}. \end{aligned}$$

#### Asynchronous label propagation algorithm for community detection

The asynchronous label propagation algorithm^52^ simulates the diffusion of labels along the links in the examined network, where the nodes are labelled by the identifier of the community to which they belong, and these labels are regularly updated based on the labels of the neighbouring nodes using a majority rule. The idea behind this method is that as the labels propagate, the densely connected groups of nodes will reach a consensus on a unique label. This approach is not aimed at optimising any predefined measure or function.

Initially, a unique community label is assigned to each node in the network. Afterwards, the following asynchronous update process is repeated until every node in the network has at least as many neighbours within its own community as it has in any other communities:

1. Nodes are arranged in a random order.
2. According to this order, we iterate over the nodes and update their label one by one based on their neighbours: each node joins the community to which most of its neighbours currently belong. Note that the label of the neighbours may have already been updated in the given iteration. The neighbouring labels are weighted based on the strength of their link connected to the current node, and ties in the weighted number of neighbours are broken at random.

Due to the random propagation of the labels, in this approach it is possible that distinct communities may eventually settle to the same label. Therefore, after the termination of the above algorithm, we also applied a breadth-first search on the subgraphs of each individual community to separate the disconnected (i.e. connected only via nodes of different communities in the original network) groups of nodes having the same label, as suggested in Ref.^52^.

#### Louvain algorithm for community detection

Though finding the exact maximum of modularity is a computationally hard problem^59^, over the years several heuristic modularity optimisation methods were proposed^9,10^, and one of the most popular among these is the Louvain algorithm^44^. This approach is capable of unfolding a complete hierarchical community structure (where modules can be composed of submodules) within a relatively short time even for extremely large networks. The algorithm is repeating two phases iteratively until the modularity stops improving:

1. Searching for a local maximum in the modularity at the given organisation level of the network.
  First, a unique community is assigned to each node of the current network.
  This is followed by a repeated iteration over the nodes until the modularity does not increase any further (or, in our case, until the gain in the modularity decreases below a threshold of $\Delta Q_{\mathrm{min}}=10^{-7}$).
    We evaluate the changes in the modularity that would take place if the current node *i* was transferred to the community of each of its neighbours.
    If all the calculated modularity changes are negative, node *i* stays in its current community. Otherwise, we carry out the transfer of node *i* where the improvement in the modularity is the largest.
2. Moving up to the next organisation level of the system represented by the network between the just found communities:
  Each community is considered as a single node.
  A self-loop is created for each new node, weighted by twice the sum of the link weights within the corresponding community.
  The new nodes are connected by links weighted by the sum of the link weights between the corresponding community members on the previous organisation level.

In our investigations, we weighted the links in the examined hyperbolic networks according to Eq. (9) and considered only the final partition (i.e. the top-level community structure, having the highest modularity among the different organisation levels) found by the implementation of the algorithm available.

#### Infomap algorithm for community detection

The Infomap algorithm, as suggested by its name, provides an information-theoretic approach for finding communities in networks^51^ based on a correspondence between the optimal community structure and the most parsimonious description of an infinitely long random walk trajectory on the network. The random walk can be considered as a proxy for the flow in the network (travelling passengers, spreading ideas, etc.), making its components interdependent to varying extents. It is intuitive to assume that communities correspond to localized regions of the network where random walkers spend a lot of time. We can take advantage of this property of communities when aiming for the most compact description of a random walker trajectory as follows.

In a simple approach, the trajectory is corresponding to the sequence of the visited nodes, each labelled with a unique codeword. However, trajectories can be defined more concisely by using a map-like description following the principle of geographic maps, where e.g. the same street names appear in multiple cities. In a similar manner, after naming the communities, the code words of the nodes can be recycled among the different communities, and only the members of the same community have to be given unique names. By limiting the number of different code words used to denote the nodes, the length of these code words can be reduced, leading to a considerable saving in the length of the trajectory description. Naturally, the recycling of the code words also comes at a cost, namely one has to indicate when the random walker leaves a given community to enter a new one by specifying the code word of the new community. Nevertheless, if communities are well separated from each other, then the transition between communities is not frequent, and we gain in the length of the trajectory description even with this extra cost taken into account.

For a map-like trajectory description based on a given community structure, the efficiency can be evaluated by the so-called map equation^51^, expressing the optimal code length (i.e. the theoretical lower bound of the code length) for an average movement of an infinitely long random walk. The Infomap algorithm itself searches for the multi-level, hierarchical network partition minimising the map equation in a heuristic manner, splitting modules into submodules, subsubmodules and so on in order to reduce the description length. If the splitting of a given leaf in the community hierarchy does not decrease the description length anymore, the downward growth of the given branch in the hierarchy is stopped. In our community analysis, we used link weights calculated according to Eq. (9) and queried from the output of the algorithm the communities corresponding to the leaves of the community hierarchy.

## Results

We generated random graphs using the PSO and the ${\mathbb {S}}^1/{\mathbb {H}}^2$ models in a wide range of parameter settings, and used the obtained networks as inputs for the community finding methods given by the asynchronous label propagation, the Louvain and the Infomap algorithms. According to the results, the hyperbolic random graphs seemed to possess a strong community structure for several regions of the parameter space.

**Figure 1:**
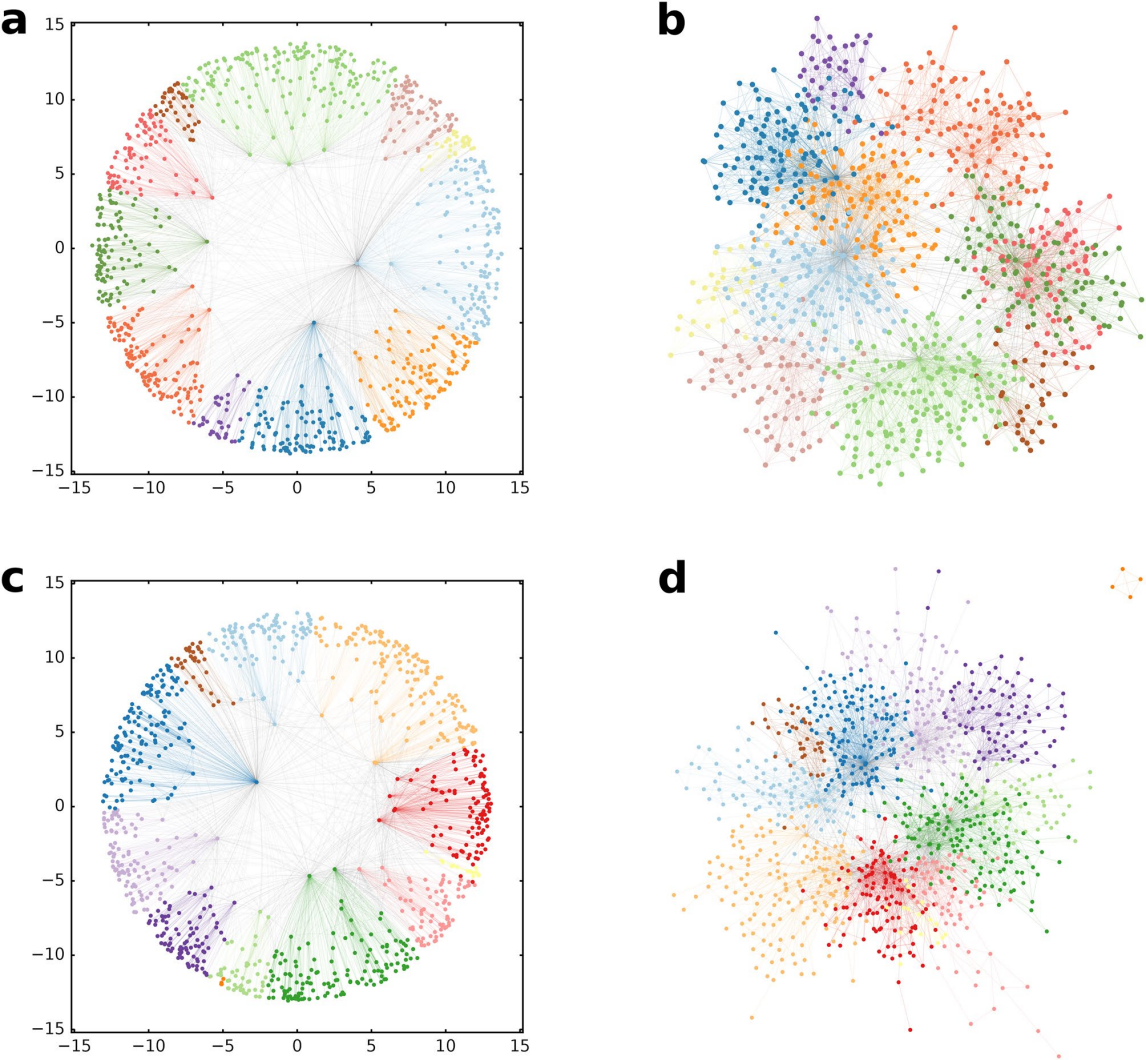
Communities found by the Louvain algorithm in hyperbolic networks. (**a**) The obtained communities (colour coded) in a network with $N=1000$ number of nodes, generated by the PSO model with parameters $m=5$ (corresponding to $\langle k\rangle = 10$), $\beta = 0.7$ (corresponding to $\gamma =2.43$) and $T=0.2$ (resulting in an average clustering coefficient of 0.58). The layout shows the network in the native disk representation of the two-dimensional hyperbolic space of curvature $K=-1$, with the nodes arranged according to their coordinates assigned during the network generation process. The weighted modularity for the found partition is $Q=0.75$ and the angular separation index is $\mathrm {ASI}=1.0$. (**b**) Layout of the network shown in (**a**) on the Euclidean plane. (**c**) The detected communities in a network generated by the ${\mathbb {S}}^1/{\mathbb {H}}^2$ model with parameters $N=1000$, $\langle k\rangle =10$, $\gamma =2.43$ and $\alpha =5$ (resulting in an average clustering coefficient of 0.71), shown in the native disk representation of the hyperbolic plane of curvature $K=-1$. The weighted modularity of the shown partition is $Q=0.74$, the angular separation index is $\mathrm {ASI}=0.998$. (**d**) Layout of the same network as in (**c**) on the Euclidean plane.

As an illustration, in Fig. 1 we show the partition found by the Louvain algorithm in networks of size $N=1000$ both according to the layout in the native disk representation of the two-dimensional hyperbolic space and according to a standard layout in the Euclidean plane. In Fig. 1a,c, the sets of nodes grouped together by Louvain occupy well-defined angular regions in the hyperbolic disk with barely any overlap with the region of the neighbouring communities. However, according to Fig. 1b,d, the detected communities are clearly outlined even in such layouts which do not build on the hyperbolic origin of the networks.

**Figure 2:**
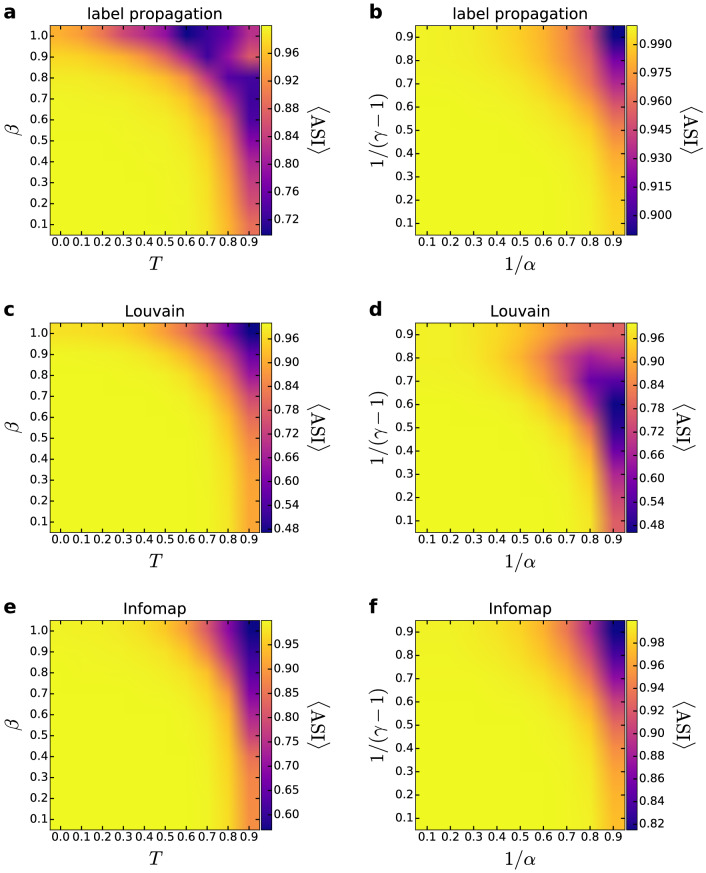
Angular separation index in the PSO and the ${\mathbb {S}}^1/{\mathbb {H}}^2$ models. The results for the PSO model are given in the left column (**a**,**c**,**e**), whereas the ASI obtained for the ${\mathbb {S}}^1/{\mathbb {H}}^2$ model appears in the right column (**b**,**d**,**f**). The ASI for the communities detected by asynchronous label propagation is given in the top row (**a**,**b**), the ASI regarding the results of Louvain is shown in the middle row (**c**,**d**) and the ASI for the partitions found by Infomap is presented in the bottom row (**e**,**f**). We show the measured ASI (indicated by the color, averaged over 100 samples) as a function of the model parameters *T* and $\beta$, or $1/\alpha$ and $1/(\gamma -1)$ for networks of size $N=10{,}000$ and expected average degree $\langle k\rangle =10$.

We found that the angular separation of the detected modules exemplified by Fig. 1a,c is quite general in the hyperbolic disk. Using the angular separation index (ASI), we evaluated quantitatively the angular separation of the modules obtained with the asynchronous label propagation, the Louvain and the Infomap algorithms for a large variety of the network generation parameters. In the case of the PSO model, for both the temperature *T* and the popularity fading parameter $\beta$ we took 10 equidistant data points between 0 and 1 (altogether 100 parameter combinations in the $T-\beta$ parameter plane) and generated 100 networks with each parameter setting. In the case of the ${\mathbb {S}}^1/{\mathbb {H}}^2$ model, to allow a straightforward comparison with the results seen for the PSO model, instead of the original model parameters $\alpha$ and $\gamma$ we changed to $1/\alpha$ (analogous to the temperature *T* in the PSO model) and $1/(\gamma -1)$ (equivalent to the popularity fading parameter $\beta$ in the PSO model). Similarly to the studies of the PSO model, we considered a $9\times 9$ grid in the $1/\alpha -1/(\gamma -1)$ parameter plane (our simulations relied on finite $\alpha$ values and $\gamma >2$; thus, the $T=1/\alpha =0$ and the $\beta =1/(\gamma -1)=1$ points are not contained in the studied grid), and generated 100 networks for each parameter combination. As it is shown in Fig. 2, for PSO and ${\mathbb {S}}^1/{\mathbb {H}}^2$ networks of size $N=10{,}000$ and average degree $\langle k\rangle =10$ a considerably high ASI can be obtained with all three community finding methods for most of the $T-\beta$ and $\alpha -\gamma$ parameter settings.

**Figure 3:**
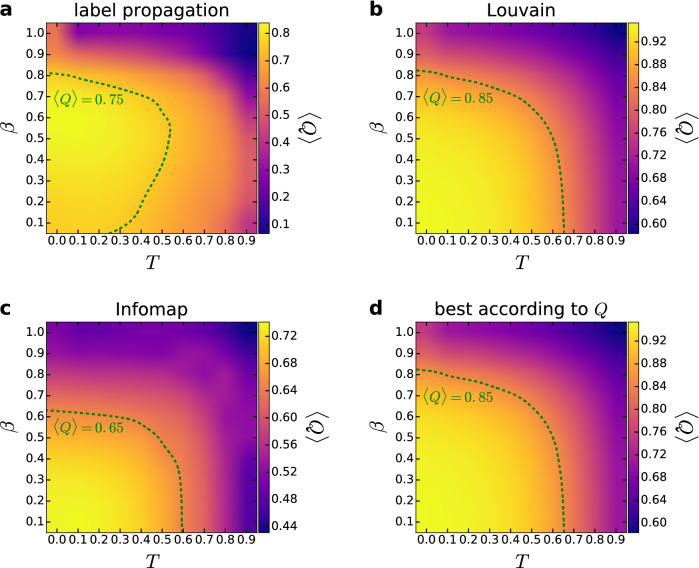
Modularity in the PSO model. We show the weighted modularity *Q* (indicated by the color, averaged over 100 samples) as a function of the model parameters *T* and $\beta$ for networks of size $N=10{,}000$ and expected average degree $\langle k\rangle =10$. The panels correspond to the results obtained with asynchronous label propagation (**a**), Louvain (**b**), Infomap (**c**), and when the best community partition is taken from the three methods according to *Q* (**d**).

**Figure 4:**
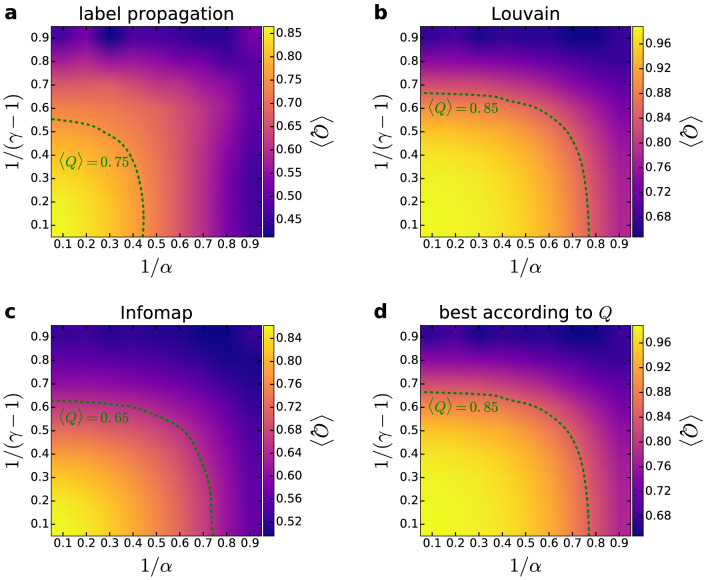
Modularity in the ${{S}}^1/{{H}}^2$ model. We show the weighted modularity *Q* (indicated by the color, averaged over 100 samples) as a function of the model parameters $1/\alpha$ and $1/(\gamma -1)$ for networks of size $N=10{,}000$ and expected average degree $\langle k\rangle =10$. The panels correspond to the results obtained with asynchronous label propagation (**a**), Louvain (**b**), Infomap (**c**), and when the best community partition is taken from the three methods according to *Q* (**d**).

In order to verify that the angularly separated modules detected by the asynchronous label propagation, the Louvain and the Infomap algorithms are indeed relevant structural units of the networks, we measured the quality of the extracted community partitions by the weighted modularity *Q* given in Eq. (8). In Figs. 3 and 4, we show the corresponding results for networks of size $N=10{,}000$ and expected average degree $\langle k\rangle =10$, where the weighted modularity is plotted as a function of the model parameters with the help of heat maps. According to Fig. 3, for a considerably large region in the parameter plane the modularity averaged over 100 networks is larger than 0.65 for the communities found by Infomap (Fig. 3c), larger than 0.75 for the communities extracted by asynchronous label propagation (Fig. 3a) and larger than 0.85 for the communities located by Louvain (Fig. 3b). For Louvain and Infomap, the highest scores in the modularity are achieved at low *T* and $\beta$ parameters, corresponding to networks with a high average clustering coefficient and a rather homogeneous degree distribution. The modularity is high in this region also for the asynchronous label propagation; however, in this case the highest modularity values occur for mid-range $\beta$ values. When $\beta$ approaches 1, the observed *Q* seems to decrease for all community finding methods. Nevertheless, *Q* can still take relatively high values at e.g. $\beta =0.6$, where the generated network is expected to be scale-free with a degree decay exponent of $\gamma \simeq 2.67$. According to the results displayed in Fig. 4, the maximum of *Q* for the ${\mathbb {S}}^1/{\mathbb {H}}^2$ model is in the low-value regime of the $1/\alpha -1/(\gamma -1)$ parameter plane for all three community finding methods, where the modularity values seem to be higher by a small margin compared to the case of the PSO model, e.g. reaching up to $\langle Q\rangle =0.99$ for the communities found by Louvain.

As mentioned in the Introduction, a large modularity value alone does not always indicate a true modular structure as e.g. both Erdős–Rényi random graphs and Barabási–Albert random graphs have been shown to display relatively high modularity values under certain circumstances^49,50^. However, for random graphs generated by the aforementioned two classical models with the same size and link density as in Figs. 3 and 4, the modularity can reach up to only about 0.28, which is significantly smaller compared to the *Q* values we observed in the studied hyperbolic networks. Furthermore, in the present study 2 out of the 3 community finding methods applied are not based on modularity maximisation, and they still find communities that yield high *Q* values.

**Figure 5:**
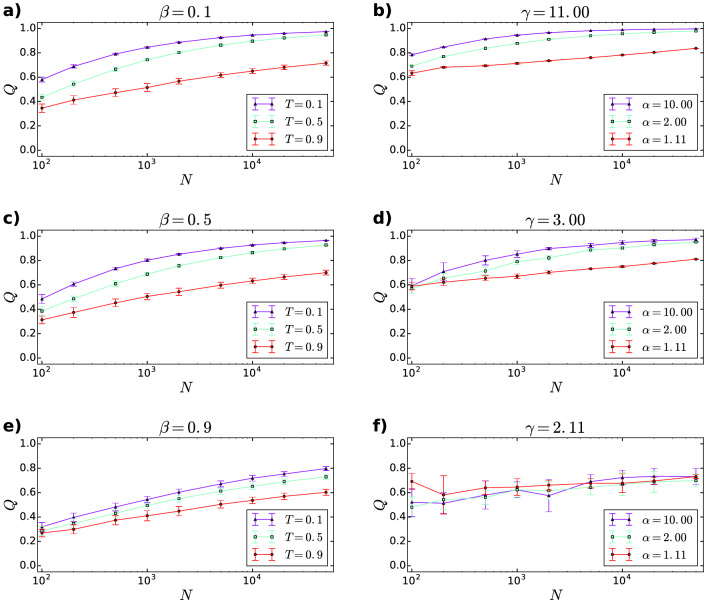
Size dependence of the modularity in the PSO model and the ${{S}}^1/{{H}}^2$ model. We plotted the highest weighted modularity *Q* achieved between the asynchronous label propagation, the Louvain and the Infomap algorithms, as a function of the number of nodes *N*. The panels on the left display the results for the PSO model, whereas the panels on the right correspond to the ${\mathbb {S}}^1/{\mathbb {H}}^2$ model. The expected average degree $\langle k\rangle$ was set to 10 in each case. The further model parameters, such as the $\beta$, *T*, $\alpha$ and $\gamma$ values appear as panel titles and legends. Each data point was obtained by averaging over 10 networks of the given parameter set. The error bars indicate the standard deviations among the 10 networks.

A further question arising related to the results shown in Figs. 3 and 4 is whether we are facing a finite size effect of some sort, where the large modularity observed at the current system size $N=10{,}000$ will decrease when the networks are enlarged, eventually approaching zero in the thermodynamic limit. The results displayed in Fig. 5 clearly show that this is not the case, as the highest weighted modularity *Q* achieved between the asynchronous label propagation, the Louvain and the Infomap algorithms increases as a function of *N* for both the PSO and the ${\mathbb {S}}^1/{\mathbb {H}}^2$ networks for almost all parameter settings. The only exception occurs in Fig. 5f, where it is hard to judge whether *Q* remains constant or is slightly increasing for the red coloured curve corresponding to $\alpha =1.11$ and $\gamma =2.11$ in the ${\mathbb {S}}^1/{\mathbb {H}}^2$ model. Based on Fig. 5, we can conclude that in the parameter regime where high *Q* values were observed in the present work, the modularity seems to show an increasing tendency when the studied hyperbolic networks are enlarged and the parameters (other than the number of nodes) of the network generation process are kept constant.

**Figure 6:**
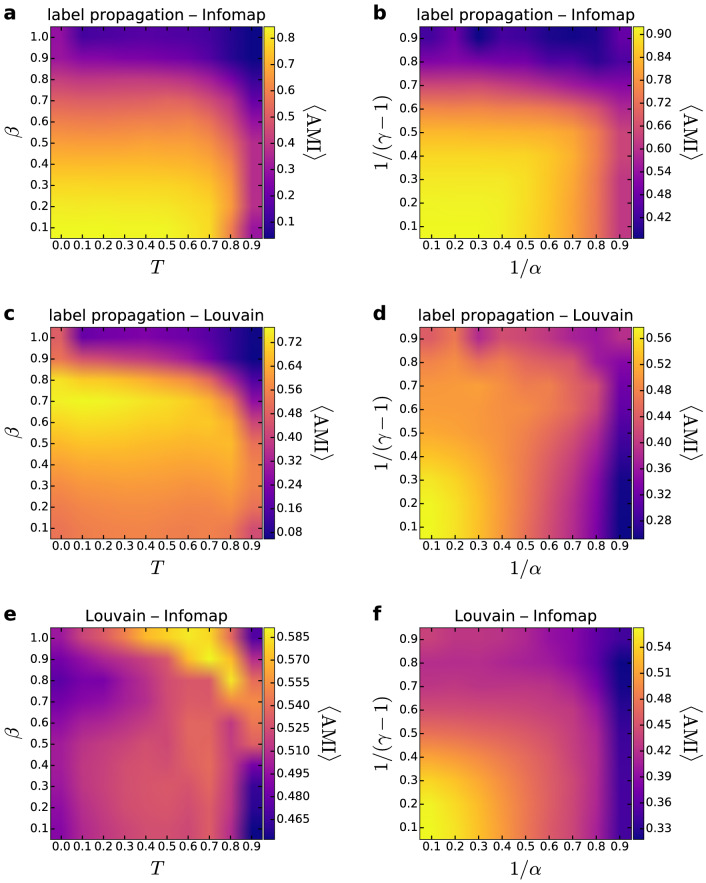
Adjusted mutual information between the different community partitions. The results for the PSO model are given in the left column (**a**,**c**,**e**), whereas the AMI obtained for the ${\mathbb {S}}^1/{\mathbb {H}}^2$ model appears in the right column (**b**,**d**,**f**). The AMI between the communities detected by asynchronous label propagation and Infomap is given in the top row (**a**,**b**), the AMI regarding the results of asynchronous label propagation and Louvain is shown in the middle row (**c**,**d**) and the AMI between the partitions found by Louvain and Infomap is presented in the bottom row (**e**,**f**). We show the measured AMI (indicated by the color, averaged over 100 samples) as a function of the model parameters *T* and $\beta$, or $1/\alpha$ and $1/(\gamma -1)$ for networks of size $N=10{,}000$ and expected average degree $\langle k\rangle =10$.

In order to examine the significance of the found communities from another aspect, we also compared the community partitions obtained with the different methods using the adjusted mutual information. The results are displayed in Fig. 6 with the help of heat maps, showing the AMI averaged over 100 networks as a function of the model parameters in the studied parameter planes. According to the figure, the highest similarity values occur between the communities found by asynchronous label propagation and Infomap (Fig. 6a,b). These can reach up to even $\langle \mathrm {AMI}\rangle =0.9$, indicating an almost one-to-one correspondence between the modules of the different partitions. On the other hand, the lowest similarity values can be observed for Louvain and Infomap (Fig. 6e,f), where the typical value of the AMI is about 0.5. However, this is still in the range of acceptable consistency between the different partitions and is definitely way higher than what we would expect e.g. for random partitions. Therefore, based on Fig. 6 we can say that in those parameter regions where the communities are characterised by relatively high modularity scores, the partitions obtained with the different community detection methods also show significant consistency with each other. This fact reassures that the modules we observe in the studied hyperbolic networks are indeed relevant and apparent structural units that can be detected based on multiple approaches in a consistent way.

**Figure 7:**
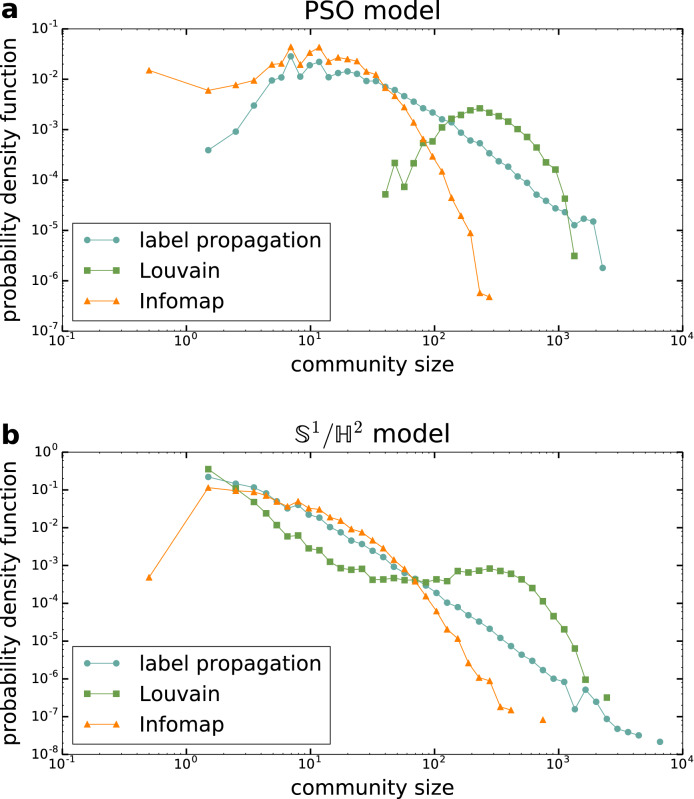
Community size distributions. (**a**) The probability density function of the community size in the PSO model according to asynchronous label propagation (blue circles), Louvain (green squares) and Infomap (orange triangles) based on 100 networks of size $N=10{,}000$, expected average degree $\langle k\rangle =10$, temperature $T=0.2$ and popularity fading parameter $\beta =0.7$. (**b**) The probability density function of the community size in the ${\mathbb {S}}^1/{\mathbb {H}}^2$ model with the same symbol and colour coding as in (**a**), based on 100 networks of size $N=10{,}000$, expected average degree $\langle k\rangle =10$, $1/\alpha =0.2$ and $1/(\gamma -1)=0.7$.

A basic statistic regarding the revealed community structures is given by the community size distribution, which is exemplified by Fig. 7 for the three examined community finding methods. According to that, the size of the communities found by the asynchronous label propagation follows more or less a power law for both the PSO model (Fig. 7a) and the ${\mathbb {S}}^1/{\mathbb {H}}^2$ model (Fig. 7b). In the regime of small and middle-sized communities, the curve corresponding to Infomap seems to be close to that; however, towards the larger sizes it decays faster. In contrast, the community size distribution yielded by Louvain is quite distinct from the curves obtained with both asynchronous label propagation and Infomap, mostly due to a peak at higher community sizes for both the PSO model and the ${\mathbb {S}}^1/{\mathbb {H}}^2$ model. This difference between the community size distributions is in correspondence with the results seen for the AMI, where the output of Infomap and asynchronous label propagation turned out to be more similar to each other than to Louvain.

An interesting question related to the visibly strong community structure obtained with the studied hyperbolic models is how does it relate to the community structure of such networks where the angular distribution of the nodes is non-uniform, as in the case of the hyperbolic network models proposed in Refs.^14–16,18^. To address this question, here we define a transition between PSO networks with uniform angular node distribution and PSO networks generated with clear angular separation between modules in a similar fashion to the nPSO model introduced in Refs.^15,16^, but with uniform angular distribution within the supposed communities instead of Gaussian distribution. Our related framework begins with generating a PSO network and then running a community finding algorithm on the resulting network for locating its modules (we used Louvain for this purpose). Based on the found communities, we can then generate PSO networks with equally-sized gaps between the supposed modules by dividing the $[0,2\pi )$ interval into subintervals having a width proportional to the size of the given community, where the aggregated width of the subintervals can be expressed as $2\pi (1-g)$ when the aggregated width of the gaps is $2\pi g$. The number of nodes placed in a given subinterval is equal to the number of members of the corresponding community, and the angular coordinate of these nodes is distributed uniformly at random within the subinterval. Otherwise, the network generation process is identical to that in the original PSO model.

**Figure 8:**
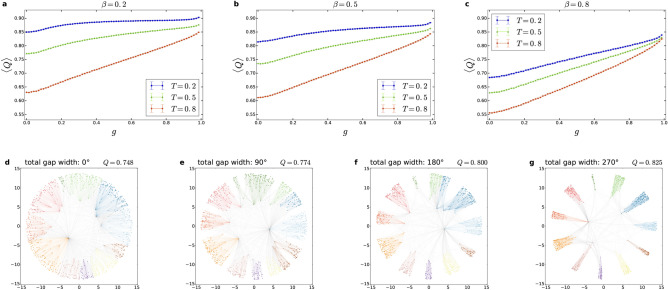
Transition to non-uniform angular node distribution in the case of the PSO model. The weighted modularity *Q* averaged over 100 networks of size $N=1000$ and expected average degree $\langle k\rangle = 10$ is shown as a function of the relative gap size *g* between the modules for $\beta =0.2$ in (**a**), for $\beta =0.5$ in (**b**) and for $\beta =0.8$ in (**c**). (The error bars indicate the 95% confidence intervals.) In (**d**–**g**), we show a series of network layouts at increasing gap widths for $N=1000$, $\langle k\rangle = 10$, $\beta =0.7$ and $T=0.2$, where the colours indicate the communities found by the Louvain algorithm.

In Fig. 8, we show results obtained from this framework, where the top panels depict the modularity for communities found by the Louvain algorithm as a function of the relative gap size *g*, and the bottom panels provide layout examples at different values of *g*. According to the figure, although *Q* increases as a function of the relative gap size *g* as expected, this increase is rather mild, except for large $\beta$ or *T* parameters. In other words, the modularity in the uniform PSO model can be quite close to the *Q* that we obtain for modules with high angular separation, and therefore, the communities we observe in the uniform PSO model can be viewed also as a meaningful limit for the modular structure of systems where the angular distribution of the nodes is non-uniform.

As a closing of this section, we draw the attention to Supplementary Information 2–4, listing further results on the communities found in the studied hyperbolic networks at different system sizes and average degree values. In addition, in Supplementary Information 5 we also examine what happens in the PSO model if the angular distribution of the nodes is strictly equidistant instead of homogeneous random. The qualitative behaviour of the communities found during these investigations is basically the same as seen here. Moreover, in Supplementary Information 2–4 our analysis is repeated on an extension of the PSO model known as the E-PSO model^13^ (described in Supplementary Information 1), yielding results that are again very similar to what we have detailed here. Finally, in Supplementary Information 6 we show the results obtained for the examined PSO, E-PSO and ${\mathbb {S}}^1/{\mathbb {H}}^2$ networks when setting all the link weights to 1 instead of using the link weights given in Eq. (9).

## Discussion and conclusions

Motivated by interesting signs of modules in hyperbolic networks with homogeneous angular node distribution reported in Refs.^37–40,45^, here we revisited the question of community structure in the PSO and the ${\mathbb {S}}^1/{\mathbb {H}}^2$ models in a detailed in-depth study. Although for both of these models the model construction itself lacks any intentionally built-in community structure, the networks generated in these approaches still possess apparently strong communities for a wide range of the model parameters, as indicated by the high modularity values measured on the results of three independent community finding algorithms, namely asynchronous label propagation, Louvain and Infomap. The significance of the found communities is supported by the fact that only 1 out of the 3 applied methods is based on modularity optimisation, and that the comparison between the different partitions yielded reasonably high AMI values, indicating a considerable consistency between the results. Furthermore, the modularity values that can be achieved in Erdős–Rényi random graphs or Barabási–Albert scale-free networks at the same average degree are way lower compared to the *Q* values we observed in the hyperbolic networks. In addition, the high modularity of the studied hyperbolic networks cannot be regarded as a finite size effect, since *Q* showed an increasing tendency as a function of the system size in the parameter regimes corresponding to an apparent, strong community structure. Moreover, the ASI (which is a quality measure independent of the modularity) was also very high for the major part of the parameter space.

The parameter plane in which we examined the behaviour of the modularity corresponded to the $(T,\beta )\in [0,1)\times (0,1]$ plane in the PSO model and the analogous $(\frac{1}{\alpha },\frac{1}{\gamma -1}) \in (0,1)\times (0,1)$ plane in the ${\mathbb {S}}^1/{\mathbb {H}}^2$ model. The intuitive meaning of these parameters can be summarised as follows: the average clustering coefficient of the generated networks is regulated by the temperature *T* and its counterpart $1/\alpha$ (lower values result in higher average clustering coefficients), while the power-law decay exponent $\gamma$ of the degree distribution is controlled by the popularity fading parameter $\beta$ in the case of the PSO model according to the formula $\gamma =1+1/\beta$ and is itself a parameter of the ${\mathbb {S}}^1/{\mathbb {H}}^2$ model. According to our results, when changing these parameters, the behaviour of the modularity follows a similar pattern for both hyperbolic models and all three community finding algorithms, except for the PSO model combined with asynchronous label propagation.

Putting aside the above-mentioned exception, for increasing *T* (or $1/\alpha )$, together with a decrease in the average clustering coefficient the modularity also decreases (which is absolutely natural), and when $\beta$ (or equivalently, $1/(\gamma -1)$) is increased, resulting in a more fat-tailed degree distribution, *Q* decreases again. However, the dependence of the modularity on the model parameters is not at all linear, instead we can observe a high, slightly decreasing plateau in the parameter plane with the maximum values in the origin and a relatively narrow belt of lower *Q* values at the feet of the plateau, placed far from the origin. For the communities found by asynchronous label propagation in the networks generated by the PSO model, the behaviour is slightly different: although *Q* is high close to the origin, for increasing $\beta$ it shows a slow increasing tendency, reaching its maximum in the medium $\beta$ range, followed by a drop for high $\beta$ values, similarly to the results seen for the other combinations between network generation models and community finding methods.

When considering the parameter settings close to the origin ($T\rightarrow 0,\,\beta \rightarrow 0$ in the PSO model and $1/\alpha \rightarrow 0,\,1/(\gamma -1)\rightarrow 0$ in the ${\mathbb {S}}^1/{\mathbb {H}}^2$ model), which yield the largest modularity values in most of the cases, it is important to note that the corresponding networks are homogeneous in terms of the degree (the degree decay exponent $\gamma$ is large) and do not resemble scale-free real networks. The existence of such regime in the parameter space seems to be congruent with the small-world transition identified by the renormalisation group approach in Ref.^60^, i.e., communities are the strongest where the small-world property disappears under renormalisation, and the networks have a highly local nature. However, when $\beta$ is increased (or equivalently, $\gamma$ is decreased), the modularity decreases only by a small magnitude for quite some range. E.g., at $\beta =0.6$, corresponding to $\gamma \simeq 2.67$, the modularity averaged over 100 networks can still reach up to $\langle Q\rangle =0.929$ in the PSO model and $\langle Q\rangle =0.898$ in the ${\mathbb {S}}^1/{\mathbb {H}}^2$ model. In other words, when setting the degree decay exponent to moderate values often seen in real systems with the help of $\beta$ or by directly tuning $\gamma$, the networks obtained with the studied models can still possess a strong community structure if the other parameter (*T* or $1/\alpha$, controlling the clustering coefficient) is not pushed to extremely high values, meaning that the clustering coefficient is not reduced to extremely low values.

The regime where *Q* drops to lower values is on the one hand where $\beta \rightarrow 1$ (or equivalently $\gamma \rightarrow 2$ from above), corresponding to extremely fat-tailed degree distributions, and where $T\rightarrow 1$ (or equivalently $\alpha \rightarrow 1$ from above), corresponding to networks with clustering coefficients close to zero. Thus, if one would like to generate scale-free hyperbolic networks having communities and a degree decay exponent close to $\gamma =2$, it might be a better option to choose the models in Refs.^14–16,18^, where the community formation is helped by the non-uniform angular distribution of the nodes. Nevertheless, except the mentioned extreme regimes, the studied “traditional” hyperbolic models seem to produce a strong enough community structure that can be taken as a simple model for the apparent modular structures often observed in real systems.

A remaining interesting question is why do the observed communities arise despite the absence of any explicit community formation mechanisms built into the construction of the studied models? In short, the same model properties that allow the development of a large clustering coefficient in the generated random graphs on the level of nodes also make the emergence of communities possible on a slightly larger scale. Communities are local structures in the sense that members connect to each other with a larger link density than to the rest of the system. As mentioned in the Introduction and as it can be seen in Fig. 1a,c, in hyperbolic networks such units correspond to well-defined angular regions^37–42^ with a relatively low number of links across them. Thus, as noted in Ref.^45^, the community structure of a network can be also viewed as a coarse version of its layout in the hyperbolic space.

In our view, the key element in the formation of communities in the studied models is that due to the hyperbolicity (negative curvature) of the native disk, for a node newly appearing at the periphery it is much easier to connect radially than “sideways” (i.e. to nodes with similarly large radial coordinate), as indicated by e.g. the distance formula in Eq. (2). If the angular separation between the previously arrived nodes that are placed at smaller radii is large enough, they can become distinct attractive community cores to which the new nodes can connect with only a small interference (cross-links) between the different angular regions. In the PSO model, the condition for a large enough separation between the inner nodes is that they are pushed outwards (according to the popularity fading) relatively fast, i.e. $\beta$ is not large. In parallel, the cutoff in the connection probability as a function of the hyperbolic distance must also be sharp enough for localised connections; thus, *T* must not be set large either to support community formation. A similar line of arguments holds also for the ${\mathbb {S}}^1/{\mathbb {H}}^2$ model. When $\gamma$ is large, then due to the relatively rapid decay in the degree distribution, the hidden variables $\kappa _i$ take low values that are mapped to relatively high radial coordinates even for the inner nodes, helping the formation of community cores. In parallel, a large $\alpha$ parameter in the ${\mathbb {S}}^1/{\mathbb {H}}^2$ model has a similar effect to a low *T* value in the PSO model, sharpening the cutoff in the connection probability as a function of the metric distance.

We also compared the community structure in the PSO model to the communities in networks with a non-uniform angular distribution of the nodes in a simple framework, motivated by the fact that the embedding of real networks is often non-homogeneous in terms of the angular coordinates, similarly to the hyperbolic models with built-in community formation introduced in Refs.^14–16,18^. Our framework enables a continuous transition between the homogeneous angular node distribution of the PSO model and an angular distribution with empty gaps between the supposed modules, where the angular coordinates are distributed uniformly at random inside the allowed angular regions. According to our results, the modularity shows only a mild increase as a function of the relative gap size for the majority of the parameter settings. Thus, the modules in the original PSO model can be quite close in strength to modules occurring in hyperbolic networks with a non-uniform angular node distribution, and the modular structure of the PSO model as a whole can be treated as a limiting case for those hyperbolic systems where the community structure is accompanied with a non-uniform distribution in the angular coordinates of the nodes.

Our findings are also closely related to the community structures observed in networks grown with the help of simplicial complexes^32,33^ that were also shown to be hyperbolic. Explicit community formation is not built in these models either; however, the simplicial complexes form complete subgraphs (cliques), and when aggregating such dense structures, the appearance of communities seems to be more natural compared to the models studied here, where links are introduced one by one. Nevertheless, the formation of communities observed here deepens further the connection between hyperbolic networks and the models introduced in Refs.^32,33^, that are known to possess a strong community structure.

In conclusion, our study draws the attention to the important but less known fact that the PSO and ${\mathbb {S}}^1/{\mathbb {H}}^2$ models are capable of generating random graphs that are not just small-world, highly clustered and scale-free, but in addition contain communities as well. Although the advantageous properties of hyperbolic models were already appreciated in the literature, this recognition makes them even more suitable for modelling real systems than thought before. In real systems, communities provide very important units at an intermediate level of the structural organisation of the network. Our detailed study of the behaviour of the community structure as a function of the model parameters show that modules are formed also in hyperbolic networks in an “automatic” way, simply as a consequence of the connection rules and the nature of the underlying hyperbolic geometry. These findings add a novel perspective and motivation for the studies and applications of hyperbolic network models.

## Supplementary Information

Supplementary Information 1.

Supplementary Information 2.

Supplementary Information 3.

Supplementary Information 4.

Supplementary Information 5.

Supplementary Information 6.

## References

[1] Albert R, Barabási A-L (2002). Statistical mechanics of complex networks. Rev. Mod. Phys..

[2] Newman MEJ (2006). The Structure and Dynamics of Networks.

[3] Holme P, Saramäki J (2012). Temporal networks. Phys. Rep..

[4] Milgram S (1967). The small world problem. Psychol. Today.

[5] Kochen M (1989). The Small World.

[6] Watts DJ, Strogatz SH (1998). Collective dynamics of “small-world” networks. Nature.

[7] Faloutsos M, Faloutsos P, Faloutsos C (1999). On power-law relationships of the internet topology. Comput. Commun. Rev..

[8] Barabási A-L, Albert R (1999). Emergence of scaling in random networks. Science.

[9] Fortunato S (2010). Community detection in graphs. Phys. Rep..

[10] Fortunato S, Hric D (2016). Community detection in networks: A user guide. Phys. Rep..

[11] Cherifi H, Palla G, Szymanski B, Lu X (2019). On community structure in complex networks: Challenges and opportunities. Appl. Netw. Sci..

[12] Papadopoulos F, Kitsak M, Serrano MÁ, Boguñá M, Krioukov D (2012). Popularity versus similarity in growing networks. Nature.

[13] Papadopoulos F, Psomas C, Krioukov D (2015). Network mapping by replaying hyperbolic growth. IEEE ACM Trans. Netw..

[14] Zuev K, Boguñá M, Bianconi G, Krioukov D (2015). Emergence of soft communities from geometric preferential attachment. Sci. Rep..

[15] Muscoloni A, Cannistraci CV (2018). A nonuniform popularity-similarity optimization (NPSO) model to efficiently generate realistic complex networks with communities. New J. Phys..

[16] Muscoloni A, Cannistraci CV (2018). Leveraging the nonuniform PSO network model as a benchmark for performance evaluation in community detection and link prediction. New J. Phys..

[17] Serrano MA, Krioukov D, Boguñá M (2008). Self-similarity of complex networks and hidden metric spaces. Phys. Rev. Lett..

[18] García-Pérez G, Serrano M, Boguñá M (2017). Soft communities in similarity space. J. Stat. Phys..

[19] García-Pérez G, Allard A, Serrano MÁ, Boguñá M (2019). Mercator: Uncovering faithful hyperbolic embeddings of complex networks. New J. Phys..

[20] Higham, D. J., Rašajski, M. & Pržulj, N. Fitting a geometric graph to a protein-protein interaction network. *Bioinformatics* **24**, 1093–1099. 10.1093/bioinformatics/btn079 (2008). http://oup.prod.sis.lan/bioinformatics/article-pdf/24/8/1093/16884271/btn079.pdf. 10.1093/bioinformatics/btn079 18344248

[21] Kuchaiev O, Rašajski M, Higham DJ, Pržulj N (2009). Geometric de-noising of protein-protein interaction networks. PLoS Comput. Biol..

[22] Boguñá M, Krioukov D, Claffy K (2009). Navigability of complex networks. Nat. Phys..

[23] Boguñá M, Papadopoulos F, Krioukov D (2010). Sustaining the internet with hyperbolic mapping. Nat. Commun..

[24] Bianconi G (2015). Interdisciplinary and physics challenges of network theory. Europhys. Lett..

[25] Cannistraci C, Alanis-Lobato G, Ravasi T (2013). From link-prediction in brain connectomes and protein interactomes to the local-community-paradigm in complex networks. Sci. Rep..

[26] García-Pérez G, Boguñá M, Allard A, Serrano MÁ (2016). The hidden hyperbolic geometry of international trade: World trade atlas 1870–2013. Sci. Rep..

[27] Gulyás A, Bíró J, Kőrösi A, Rétvári G, Krioukov D (2015). Navigable networks as nash equilibria of navigation games. Nat. Commun..

[28] Allard A, Serrano M, García-Pérez G, Boguñá M (2017). The geometric nature of weights in real complex networks. Nat. Commun..

[29] Candellero E, Fountoulakis N, Bonato A, Graham FC, Prałat P (2014). Clustering and the hyperbolic geometry of complex networks. Algorithms and Models for the Web Graph.

[30] Krioukov D (2016). Clustering implies geometry in networks. Phys. Rev. Lett..

[31] Borassi M, Chessa A, Caldarelli G (2015). Hyperbolicity measures democracy in real-world networks. Phys. Rev. E.

[32] Bianconi G, Rahmede C (2017). Emergent hyperbolic network geometry. Sci. Rep..

[33] Mulder D, Bianconi G (2018). Network geometry and complexity. J. Stat. Phys..

[34] Alanis-Lobato G, Mier P, Andrade-Navarro M (2016). Efficient embedding of complex networks to hyperbolic space via their laplacian. Sci. Rep..

[35] Muscoloni A, Thomas JM, Ciucci S, Bianconi G, Cannistraci CV (2017). Machine learning meets complex networks via coalescent embedding in the hyperbolic space. Nat. Commun..

[36] Kovács B, Palla G (2021). Optimisation of the coalescent hyperbolic embedding of complex networks. Sci. Rep..

[37] Wang Z, Li Q, Jin F, Xiong W, Wu Y (2016). Hyperbolic mapping of complex networks based on community information. Phys. A Stat. Mech. Appl..

[38] Wang Z, Li Q, Xiong W, Jin F, Wu Y (2016). Fast community detection based on sector edge aggregation metric model in hyperbolic space. Phys. A Stat. Mech. Appl..

[39] Wang Z, Wu Y, Li Q, Jin F, Xiong W (2016). Link prediction based on hyperbolic mapping with community structure for complex networks. Phys. A Stat. Mech. Appl..

[40] Wang Z, Sun L, Cai M, Xie P (2019). Fast hyperbolic mapping based on the hierarchical community structure in complex networks. J. Stat. Mech. Theory Exp..

[41] Bruno, M. *et al.* Community detection in the hyperbolic space (2019). arXiv:1906.09082 [physics.soc-ph] (Preprint).

[42] Muscoloni, A. & Cannistraci, C. V. Angular separability of data clusters or network communities in geometrical space and its relevance to hyperbolic embedding (2019). arXiv:1907.00025 [cs.LG] (Preprint).

[43] Newman MEJ, Girvan M (2004). Finding and evaluating community structure in networks. Phys. Rev. E.

[44] Blondel VD, Guillaume J-L, Lambiotte R, Lefebvre E (2008). Fast unfolding of communities in large networks. J. Stat. Mech. Theory Exp..

[45] Faqeeh A, Osat S, Radicchi F (2018). Characterizing the analogy between hyperbolic embedding and community structure of complex networks. Phys. Rev. Lett..

[46] Clauset A, Newman MEJ, Moore C (2004). Finding community structure in very large networks. Phys. Rev. E.

[47] Erdős P, Rényi A (1960). On the evolution of random graphs. Publ. Math. Inst. Hungar. Acad. Sci..

[48] Barabási, A.-L. & Albert, R. Emergence of scaling in random networks. *Science* **286**, 509–512. 10.1126/science.286.5439.509 (1999). https://science.sciencemag.org/content/286/5439/509.full.pdf. 10.1126/science.286.5439.509 10521342

[49] Guimerà R, Sales-Pardo M, Amaral LAN (2004). Modularity from fluctuations in random graphs and complex networks. Phys. Rev. E.

[50] Good BH, Montjoye Y-A, Clauset A (2010). Performance of modularity maximization in practical contexts. Phys. Rev. E.

[51] Rosvall M, Bergstrom CT (2011). Multilevel compression of random walks on networks reveals hierarchical organization in large integrated systems. PLoS One.

[52] Raghavan UN, Albert R, Kumara S (2007). Near linear time algorithm to detect community structures in large-scale networks. Phys. Rev. E.

[53] Vinh NX, Epps J, Bailey J (2010). Information theoretic measures for clusterings comparison: Variants, properties, normalization and correction for chance. J. Mach. Learn. Res..

[54] Krioukov D, Papadopoulos F, Kitsak M, Vahdat A, Boguñá M (2010). Hyperbolic geometry of complex networks. Phys. Rev. E.

[55] Newman MEJ (2004). Analysis of weighted networks. Phys. Rev. E.

[56] Danon L, Díaz-Guilera A, Duch J, Arenas A (2005). Comparing community structure identification. J. Stat. Mech..

[57] Lancichinetti A, Fortunato S, Kertész J (2009). Detecting the overlapping and hierarchical community structure in complex networks. New J. Phys..

[58] McCarthy AD, Matula DW (2018). Normalized mutual information exaggerates community detection performance. SIAM Workshop Netw. Sci..

[59] Brandes, U. *et al.* Maximizing modularity is hard. (2006). arXiv:physics/0608255 (arXiv Physics e-prints).

[60] García-Pérez G, Boguñá M, Serrano MÁ (2018). Multiscale unfolding of real networks by geometric renormalization. Nat. Phys..

