# Supplementary material for: The inherent community structure of hyperbolic networks
Source: Sci Rep. 2021 Aug 6;11:16050. doi: 10.1038/s41598-021-93921-2 (PMC8346486; doi:10.1038/s41598-021-93921-2)
Supplement: Supplementary file 1 — Supplementary Information 1. [file 41598_2021_93921_MOESM1_ESM.pdf]

The inherent community structure of hyperbolic networks
Supplementary A: The E-PSO model

Bianka Kovács[1] **and Gergely Palla**[1,2,3,*]

[1]Dept. of Biological Physics, Eötvös Loránd University, H-1117 Budapest, Pázmány P. stny. 1/A, Hungary
[2]MTA-ELTE Statistical and Biological Physics Research Group, H-1117 Budapest, Pázmány P. stny. 1/A, Hungary
[3]Health Services Management Training Centre, Semmelweis University, H-1125 Budapest, Kútvölgyi út 2, Hungary.
[*]pallag@hal.elte.hu

In our studies of the community structure of hyperbolic networks, besides the PSO model and the $\mathbb{S}^1/\mathbb{H}^2$ model, we also used the E-PSO model for random graph generation. The results on the communities found in this model are presented in Supplementary B, C, D and F, whereas in the present document we provide a brief introduction to the model itself.

The popularity-similarity optimisation (PSO) model[1] was generalised in the Supplementary Notes of Ref.[1] by the introduction of so-called *internal* links: in this *generalised PSO model*, in addition to the m number of *external* links connecting the new node to the already existing nodes, at each time step an L number of further *internal* connections are created between disconnected pairs of previously appeared nodes, where the formation of all types of links is determined by the usual distance-dependent probabilities. It is straightforward to extend this model to negative values of the parameter L[2], in which case after connecting the new node to m number of the already existing nodes, the total number of links between the previously appeared nodes is changed by $L<0$, i.e. $|L|$ number of internal links are removed at each time step. To maintain the trend that mostly hyperbolically close nodes are connected to each other, it is natural to use the same probability formula for retaining a link as for the link creation and, accordingly, the complementary probability of link creation as the probability of link removal.

The E-PSO model[3] is an equivalent of the generalised PSO model using solely external links, i.e. all edges of the network are established by the actual new node. Contrary to the original and the generalised PSO models, in the E-PSO model the number of links created at a time step is time-dependent. The expected number of links emerging at time $i\in[1,N]$ can be given as

$$\bar{m}_i = m+\bar{L}_i \simeq m+L\cdot\frac{2(1-\beta)}{(1-N^{-(1-\beta)})^2(2\beta-1)}\left[\left(\frac{N}{i}\right)^{2\beta-1}-1\right]\left(1-i^{-(1-\beta)}\right), \tag{A1}$$

where $\bar{L}_i$ is the expected total number of internal links from previous nodes on the node appearing at iteration i at the end of the network generation process in the generalised PSO model parametrised by the number of nodes N, the number m of external links created at each time step, the change L in the number of internal links at each time step and the popularity fading parameter β. Compared to the generalised PSO model, with the E-PSO model one can generate even large networks relatively fast, since in the generalised PSO model the hyperbolic distance needs to be calculated for all node pairs at each time step to update the probabilities of internal link creation or retainment in accordance with the updated node positions, whereas in the E-PSO model always only the distances from the newly appeared node need to be determined.

Not only the original PSO model, but the generalised PSO model and the analogous E-PSO model are capable of producing scale-free networks (with a degree decay exponent $\gamma=1+1/\beta$) that are highly clustered (in the case of small temperature T) and have the small-world property. Moreover, in the generalised PSO model and the E-PSO model, with the introduction of the parameter L even it becomes adjustable how the average internal degree of the subgraphs spanning between nodes having a degree larger than a certain threshold depends on the degree threshold[2]: for $0<L$ the average internal degree increases with the degree threshold and for $L=0$ (corresponding to the original PSO model) the average internal degree does not depend on the degree threshold until the degree threshold remains below a value at which the subgraphs become extremely small, while for $L<0$ the average internal degree gradually decreases as the degree threshold increases. Note that in these generalised versions of the PSO model the expected average degree of the resulted network can be calculated as $\langle k\rangle=2(m+L)$ instead of $\langle k\rangle=2m$.

References

1. Papadopoulos, F., Kitsak, M., Serrano, M. Á., Boguñá, M. & Krioukov, D. Popularity versus similarity in growing networks. *Nature* **489**, 537 EP –, DOI: 10.1038/nature11459 (2012).

2. Kovács, B. & Palla, G. Optimisation of the coalescent hyperbolic embedding of complex networks (2020). Preprint at https://arXiv:2009.04702 [cs.SI].

3. Papadopoulos, F., Psomas, C. & Krioukov, D. Network mapping by replaying hyperbolic growth. *IEEE/ACM Transactions on Netw.* **23**, 198–211, DOI: 10.1109/TNET.2013.2294052 (2015).
