# Supplementary material for: The inherent community structure of hyperbolic networks
Source: Sci Rep. 2021 Aug 6;11:16050. doi: 10.1038/s41598-021-93921-2 (PMC8346486; doi:10.1038/s41598-021-93921-2)
Supplement: Supplementary file 2 — Supplementary Information 2. [file 41598_2021_93921_MOESM2_ESM.pdf]

The inherent community structure of hyperbolic networks
Supplementary B: Quality of the detected communities

Bianka Kovács[1] and Gergely Palla[1,2,3,*]

[1]Dept. of Biological Physics, Eötvös Loránd University, H-1117 Budapest, Pázmány P. stny. 1/A, Hungary
[2]MTA-ELTE Statistical and Biological Physics Research Group, H-1117 Budapest, Pázmány P. stny. 1/A, Hungary
[3]Health Services Management Training Centre, Semmelweis University, H-1125 Budapest, Kútvölgyi út 2, Hungary.
[*]pallag@hal.elte.hu

We studied the quality of the community structures detected by the asynchronous label propagation[1,2], the Louvain[3,4] and the Infomap[5,6] algorithms in PSO[7], E-PSO[8,9] and $\mathbb{S}^1/\mathbb{H}^2$[10–12] networks of various parameter combinations. The isolated nodes emerging in the case of the $\mathbb{S}^1/\mathbb{H}^2$ model and occasionally also in the networks generated by the E-PSO model of $L<0$ were removed before the community detection, meaning that the actual size of the examined networks does not necessarily reach the number of nodes N inputted in these models. Each community detection algorithm was executed once for each network. Figs. B1–B3 show the achieved highest weighted modularity averaged over 100 networks of each parameter setting together with the corresponding standard deviations. Figs. B4–B12 present how the performance of the three different community detection algorithms depends on the parameters of the examined network generation models. The weighted modularity was calculated according to equations (8) and (9) of the main article, using the code available from Ref.[13].

Fig. B1 depicts the effect on the achieved highest weighted modularity of changing the number of nodes N, the expected average degree $\langle k\rangle$, the popularity fading parameter β and the temperature T in the PSO model, which corresponds to the E-PSO model with $L=0$. For large N and small $\langle k\rangle$ most of the nodes have the possibility to create connections only with hyperbolically close nodes, while for small $N/\langle k\rangle$ ratios the nodes are forced more often to connect even with farther nodes to create all the expected number of links. For this reason, a larger $N/\langle k\rangle$ ratio leads to connections that are more strongly determined by the hyperbolic distances, and thus to a more clear separation between the angular regions of the hyperbolic disk, i.e. a community structure with higher modularity. Besides, by sharpening the cutoff in the connection probability, small values of T also facilitate the localisation of the node-node connections; thus, with the decrease of the temperature T the modularity of the detected community structures increases. Furthermore, for smaller values of β the inner nodes drift faster away from each other during the network growth, forming thereby more separated attraction centres for the outer nodes, due to which most of the network nodes can make a more definite choice between the community centres, which leads to communities with less external connections, i.e. larger modularity.

Fig. B2 display how the parameters m and L affect the achieved highest weighted modularity in E-PSO networks. Based on these, not only the expected average degree $\langle k\rangle=2(m+L)$, but even m and L itself has an effect on the community structure of the generated networks. According to equation (A1) of Supplementary A, for $L<0$ the number of links created by a new node at its appearance is an increasing function of the node's appearance time, meaning that the early-appearing, inner nodes connect to few nodes only, whereas the outer nodes, for which the number of realisable connections (i.e. the number of previously appeared nodes) is not limited as much, form more connections. This way, nodes of any appearance time have the possibility to create connections only with hyperbolically close nodes. On the other hand, for $0<L$ the inner nodes create at their appearance a relatively large number of connections with the previously appeared nodes, which – in the absence of enough hyperbolically close candidates – leads to the emergence of connections between not so close nodes too. Taking into consideration the concept that the more the connections are restricted to hyperbolically close node pairs, the stronger the arising community structure, we can conclude that for a given expected average degree $\langle k\rangle$ the modularity can be increased by decreasing the parameter L and, at the same time, increasing the parameter m accordingly.

According to Fig. B3, the strength of the community structure in $\mathbb{S}^1/\mathbb{H}^2$ networks depends the same way on the model parameters as in PSO networks: the achieved highest weighted modularity is higher for larger number of nodes N, smaller average degree $\langle k\rangle$, larger degree decay exponent γ (corresponding to smaller popularity fading parameter β in the E-PSO model) and larger α (which is analogous to lower temperature T in the E-PSO model).

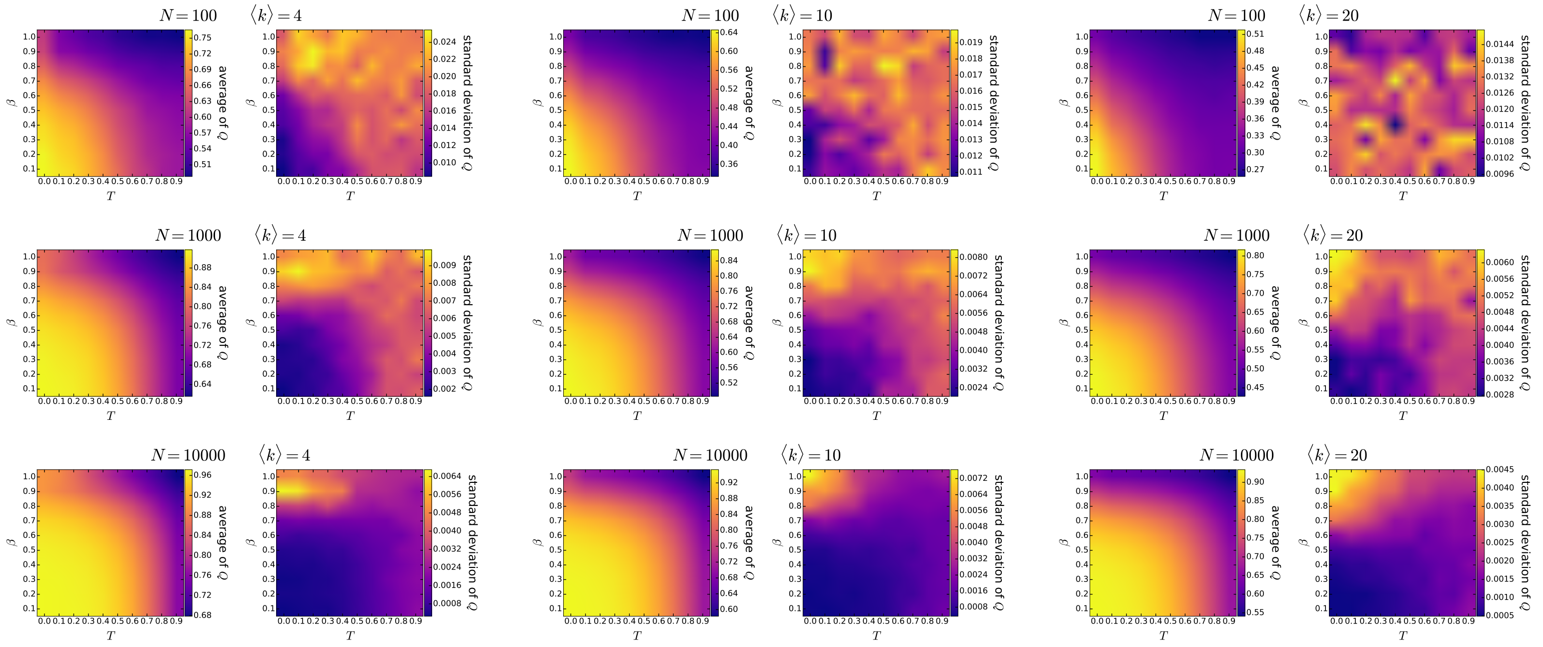

Figure B1. The mean and the standard deviation of the highest weighted modularity Q achieved among the *asynchronous label propagation*, the *Louvain* and the *Infomap* algorithms in 100 *PSO* networks of different parametrisations. Each pair of subplots depicts the effect of changing the popularity fading parameter β and the temperature T, with the number of nodes N and the expected average degree $\langle k \rangle = 2m$ given in the title of the subplot pair. The curvature of the hyperbolic plane K was always set to -1, i.e. we used $\zeta = 1$.

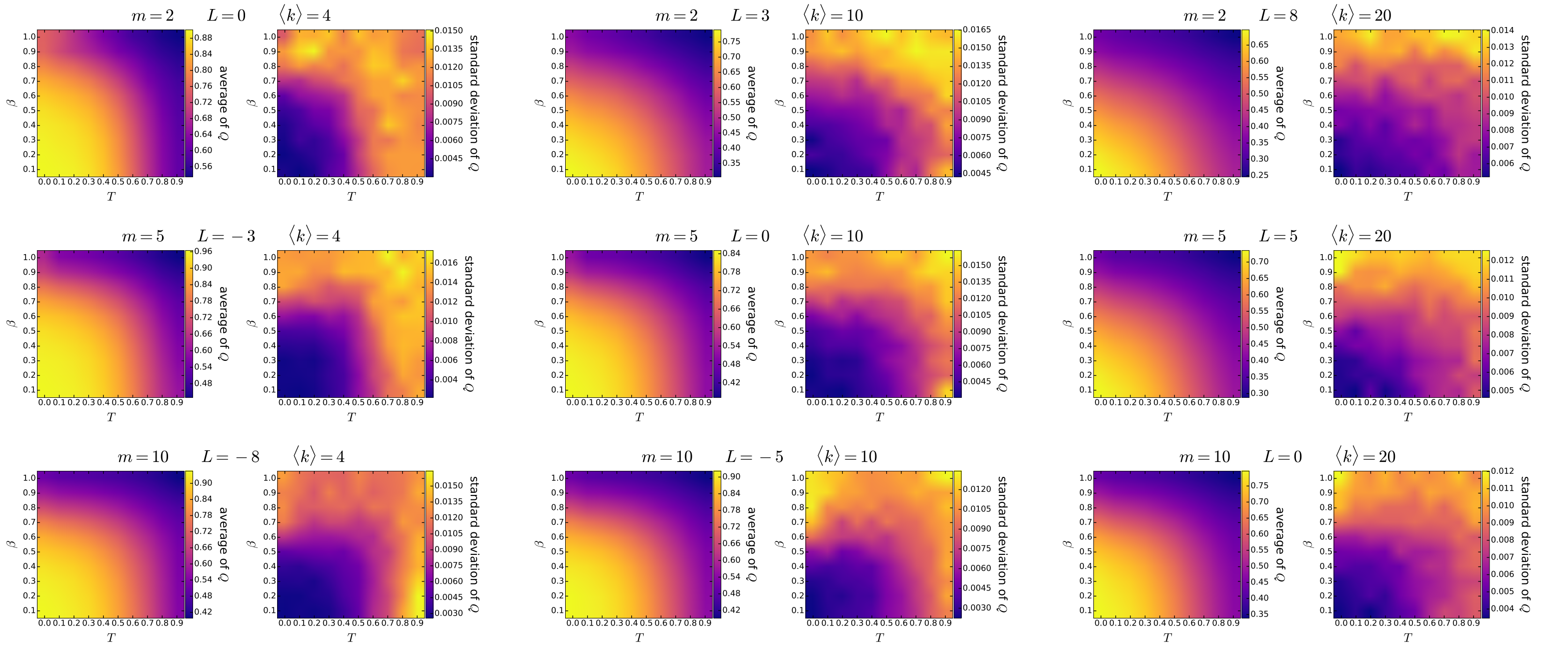

Figure B2. The mean and the standard deviation of the highest weighted modularity Q achieved among the *asynchronous label propagation*, the *Louvain* and the *Infomap* algorithms in 100 *E-PSO* networks of different parametrisations. Each pair of subplots depicts the effect of changing the popularity fading parameter β and the temperature T, with the parameters m and L given in the title of the subplot pair together with the corresponding expected average degree $\langle k \rangle = 2(m+L)$. The number of nodes N was 1000 in each case. The curvature of the hyperbolic plane K was always set to -1, i.e. we used $\zeta = 1$.

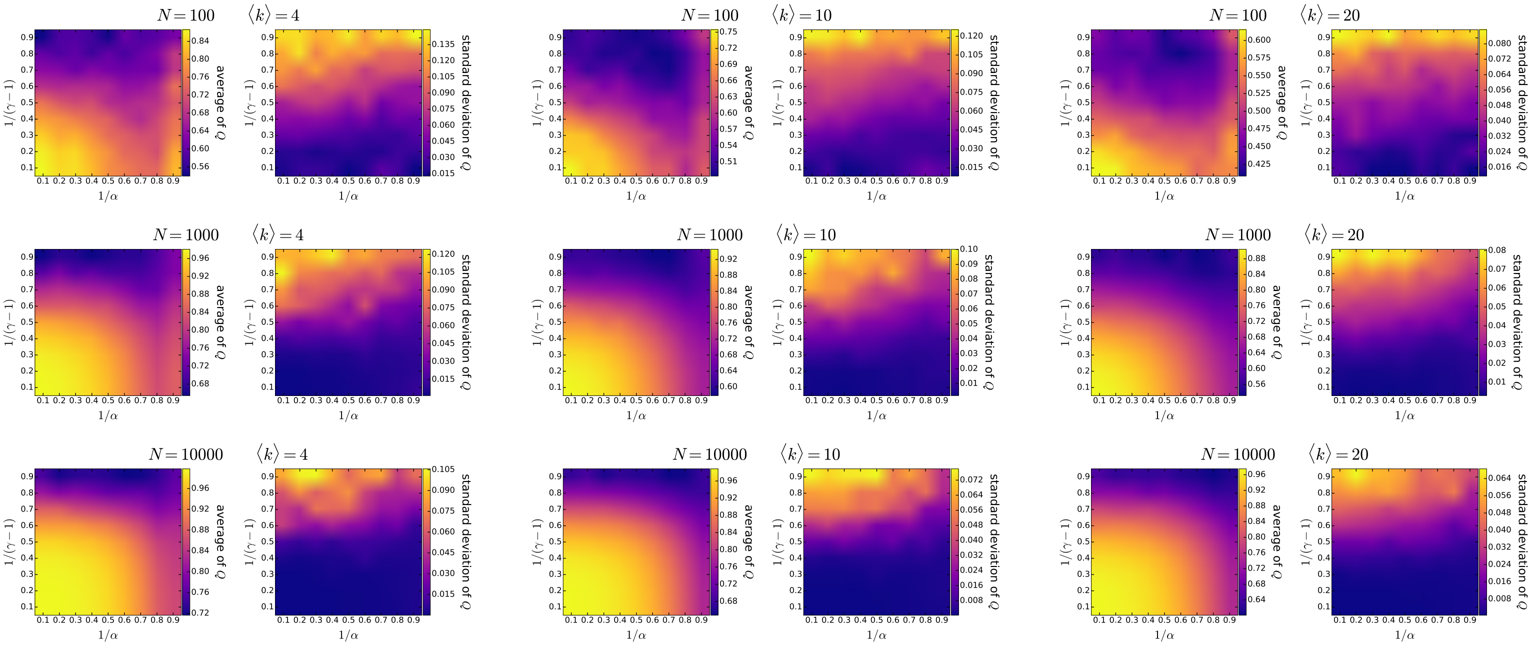

Figure B3. The mean and the standard deviation of the highest weighted modularity Q achieved among the *asynchronous label propagation*, the *Louvain* and the *Infomap* algorithms in 100 $\mathbb{S}^1/\mathbb{H}^2$ networks of different parametrisations. Each pair of subplots depicts the effect of changing $1/(\gamma-1)$ (equivalent to the popularity fading parameter β in the E-PSO model) and $1/\alpha$ (analogous to the temperature T in the E-PSO model), with the number of nodes N and the expected average degree $\langle k \rangle$ given in the title of the subplot pair. We used $K = -1$ as the curvature of the hyperbolic plane in each case.

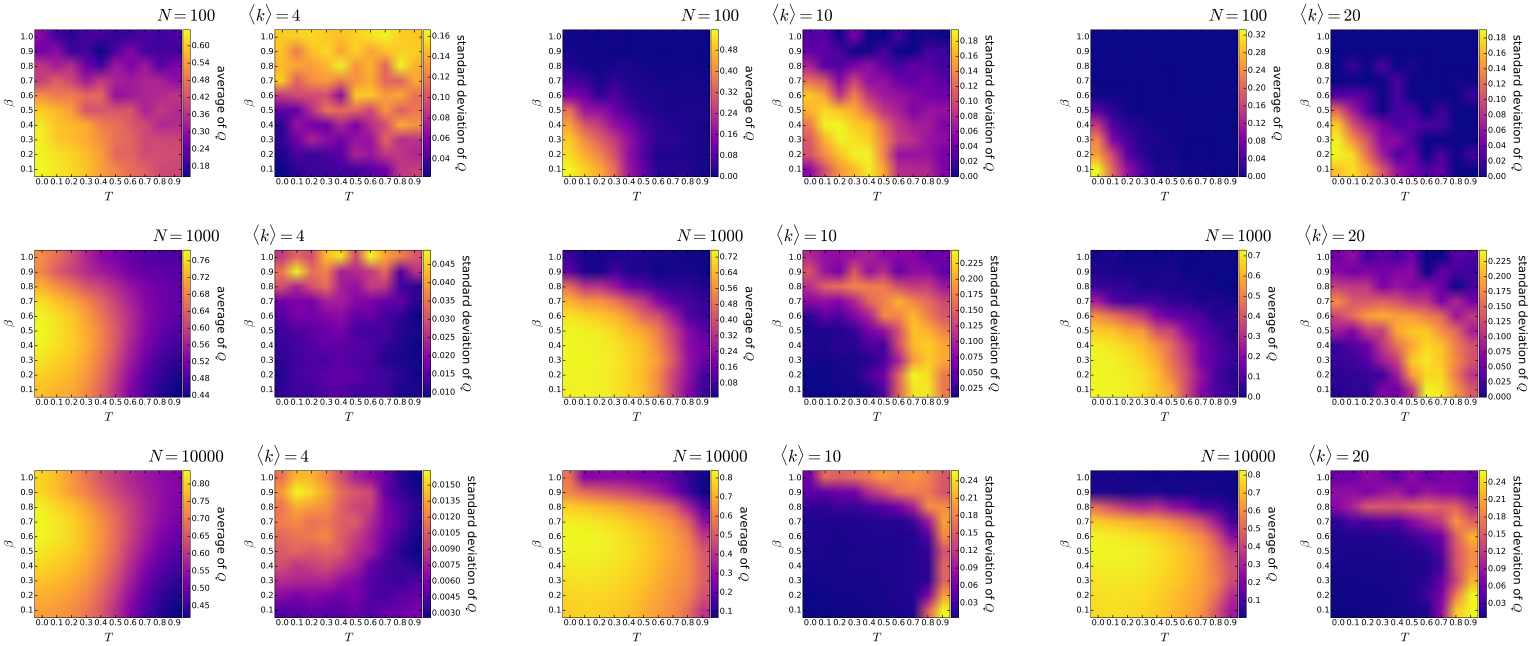

Figure B4. The mean and the standard deviation of the weighted modularity Q of the community structure detected by the *asynchronous label propagation* algorithm in 100 *PSO* networks of different parametrisations. Each pair of subplots depicts the effect of changing the popularity fading parameter β and the temperature T, with the number of nodes N and the expected average degree $\langle k \rangle = 2m$ given in the title of the subplot pair. The curvature of the hyperbolic plane K was always set to -1, i.e. we used $\zeta = 1$.

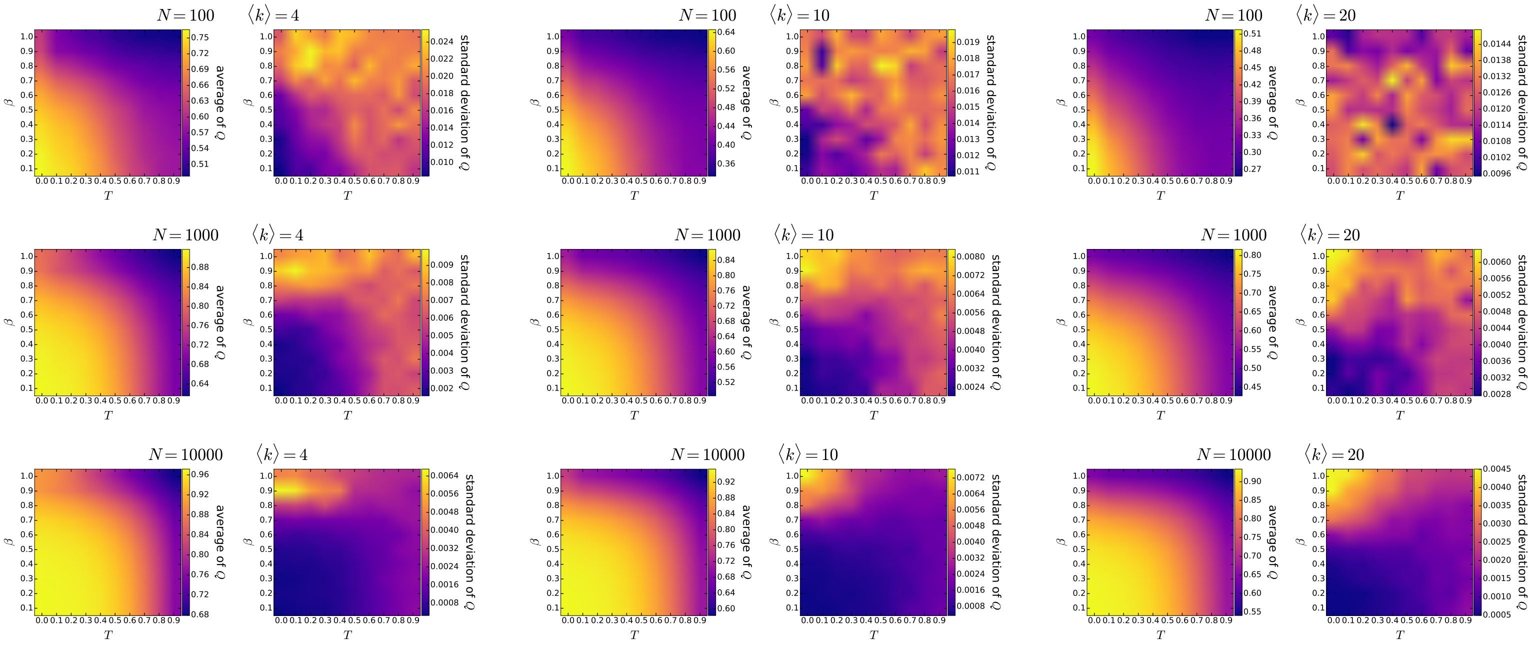

Figure B5. The mean and the standard deviation of the weighted modularity Q of the community structure detected by the *Louvain* algorithm in 100 *PSO* networks of different parametrisations. Each pair of subplots depicts the effect of changing the popularity fading parameter β and the temperature T, with the number of nodes N and the expected average degree $\langle k \rangle = 2m$ given in the title of the subplot pair. The curvature of the hyperbolic plane K was always set to -1, i.e. we used $\zeta = 1$.

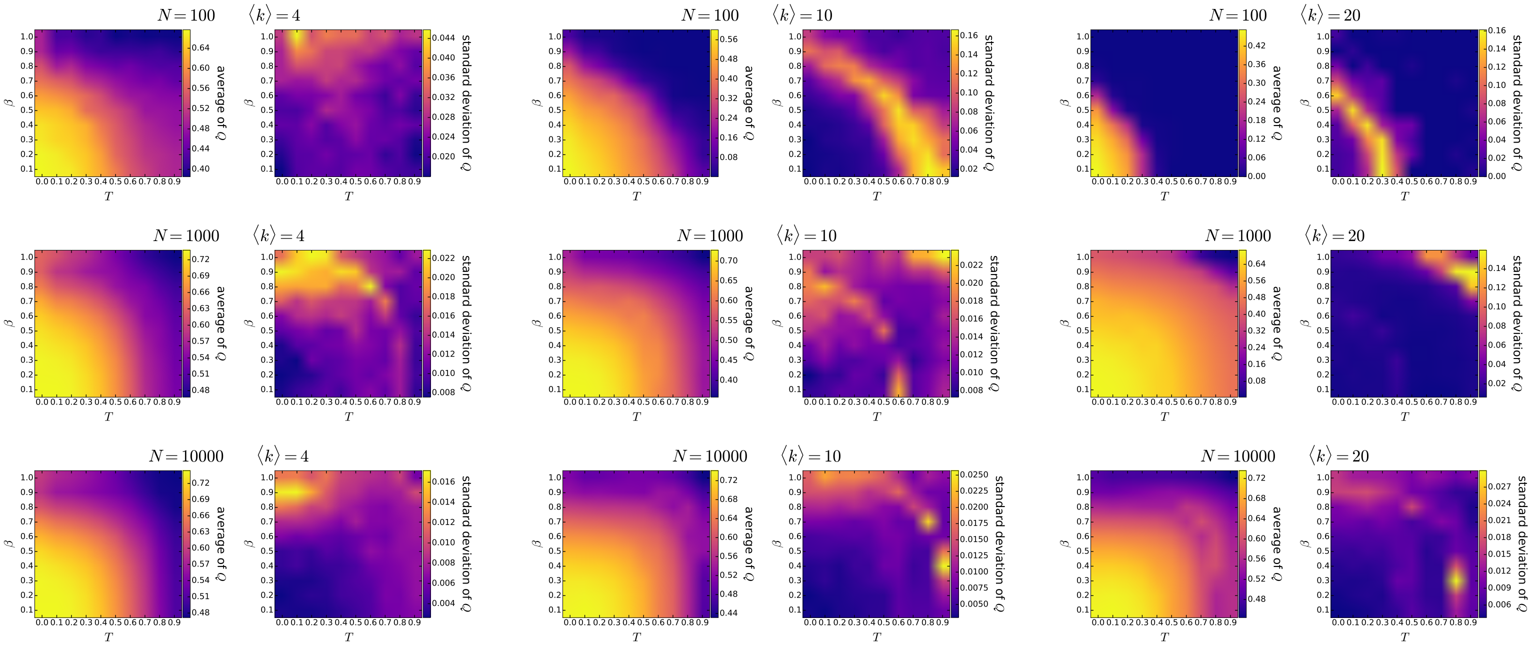

Figure B6. The mean and the standard deviation of the weighted modularity Q of the community structure detected by the *Infomap* algorithm in 100 *PSO* networks of different parametrisations. Each pair of subplots depicts the effect of changing the popularity fading parameter β and the temperature T, with the number of nodes N and the expected average degree $\langle k \rangle = 2m$ given in the title of the subplot pair. The curvature of the hyperbolic plane K was always set to -1, i.e. we used $\zeta = 1$.

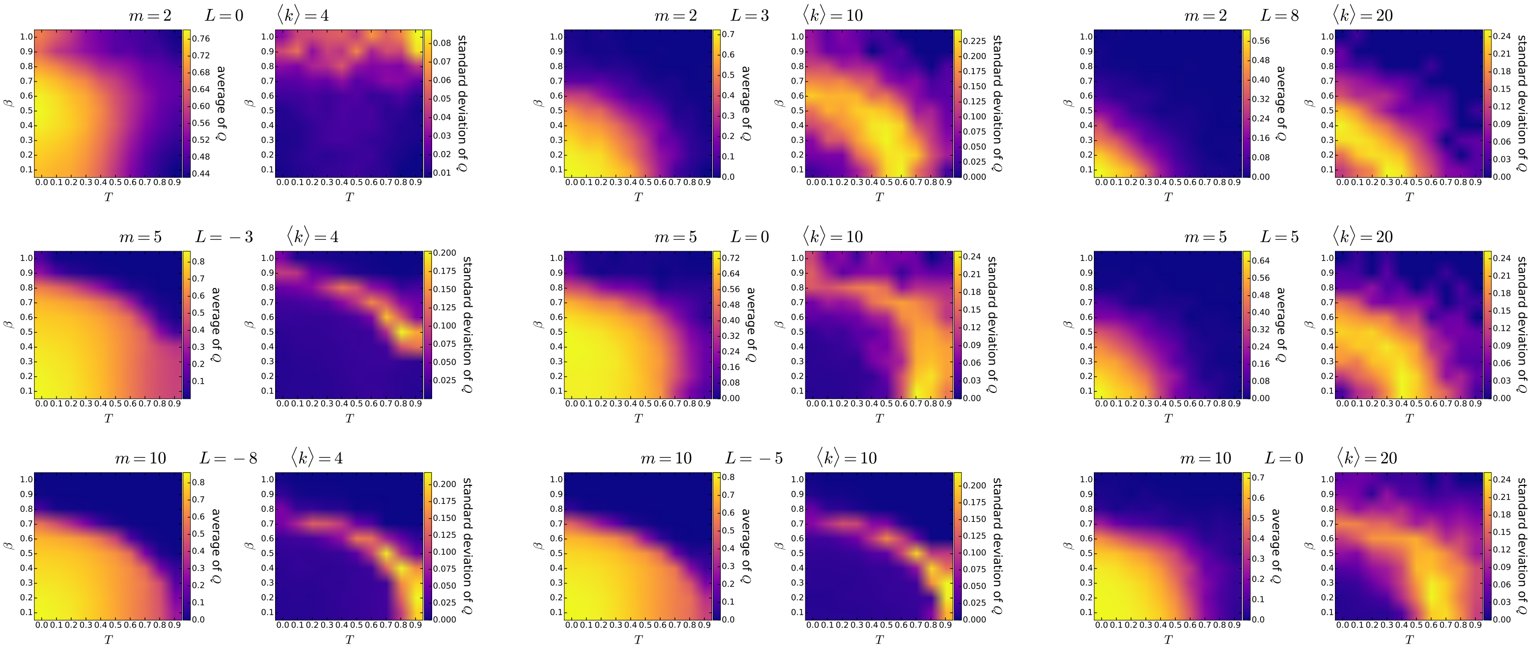

Figure B7. The mean and the standard deviation of the weighted modularity Q of the community structure detected by the *asynchronous label propagation* algorithm in 100 *E-PSO* networks of different parametrisations. Each pair of subplots depicts the effect of changing the popularity fading parameter β and the temperature T, with the parameters m and L given in the title of the subplot pair together with the corresponding expected average degree $\langle k \rangle = 2(m+L)$. The number of nodes N was 1000 in each case. The curvature of the hyperbolic plane K was always set to -1, i.e. we used $\zeta = 1$.

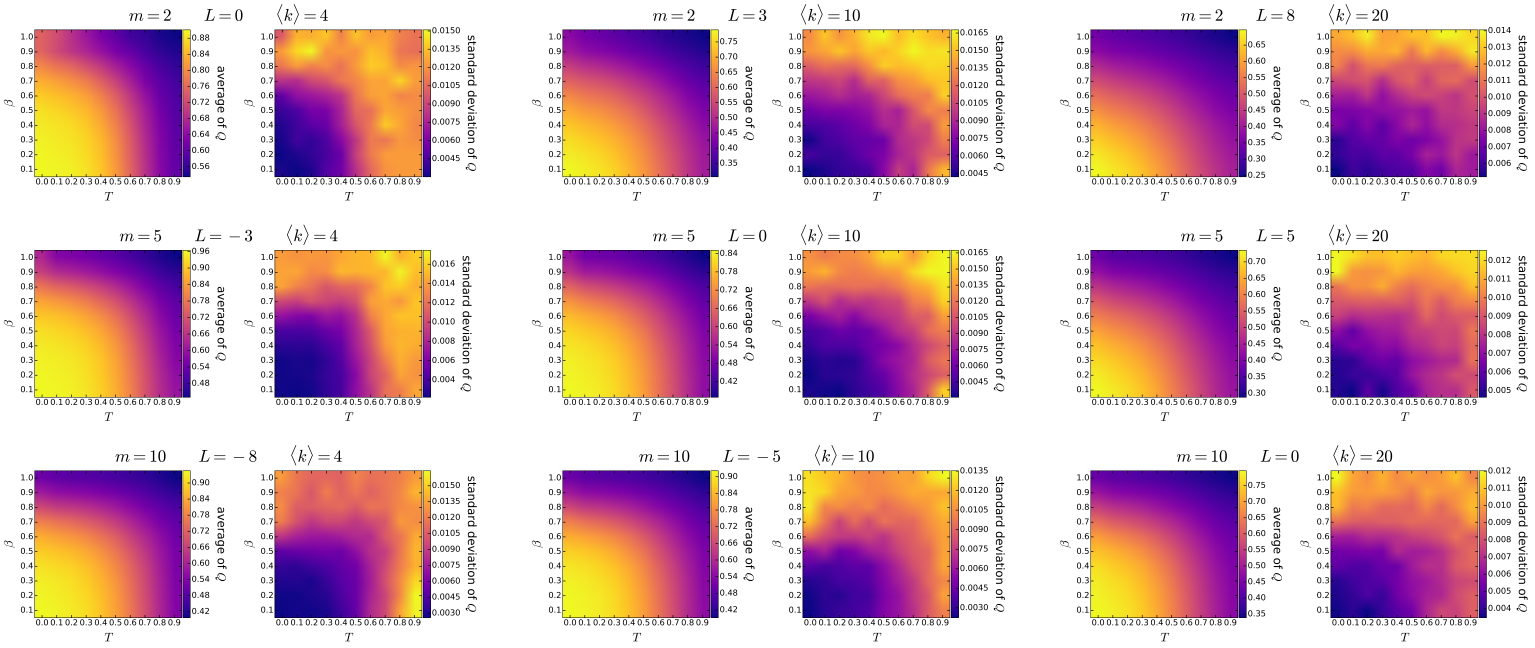

Figure B8. The mean and the standard deviation of the weighted modularity Q of the community structure detected by the *Louvain* algorithm in 100 *E-PSO* networks of different parametrisations. Each pair of subplots depicts the effect of changing the popularity fading parameter β and the temperature T, with the parameters m and L given in the title of the subplot pair together with the corresponding expected average degree $\langle k \rangle = 2(m+L)$. The number of nodes N was 1000 in each case. The curvature of the hyperbolic plane K was always set to -1, i.e. we used $\zeta = 1$.

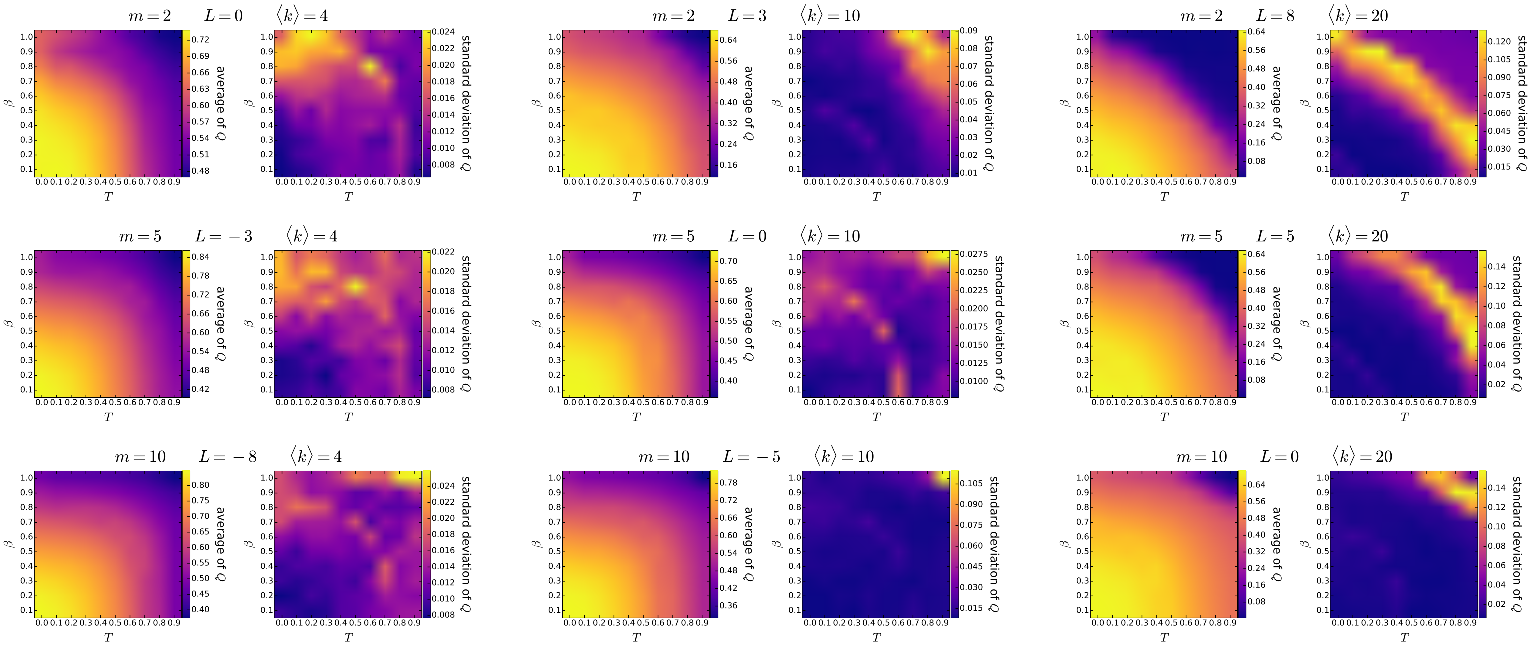

Figure B9. The mean and the standard deviation of the weighted modularity Q of the community structure detected by the *Infomap* algorithm in 100 *E-PSO* networks of different parametrisations. Each pair of subplots depicts the effect of changing the popularity fading parameter β and the temperature T, with the parameters m and L given in the title of the subplot pair together with the corresponding expected average degree $\langle k\rangle = 2(m+L)$. The number of nodes N was 1000 in each case. The curvature of the hyperbolic plane K was always set to -1, i.e. we used $\zeta = 1$.

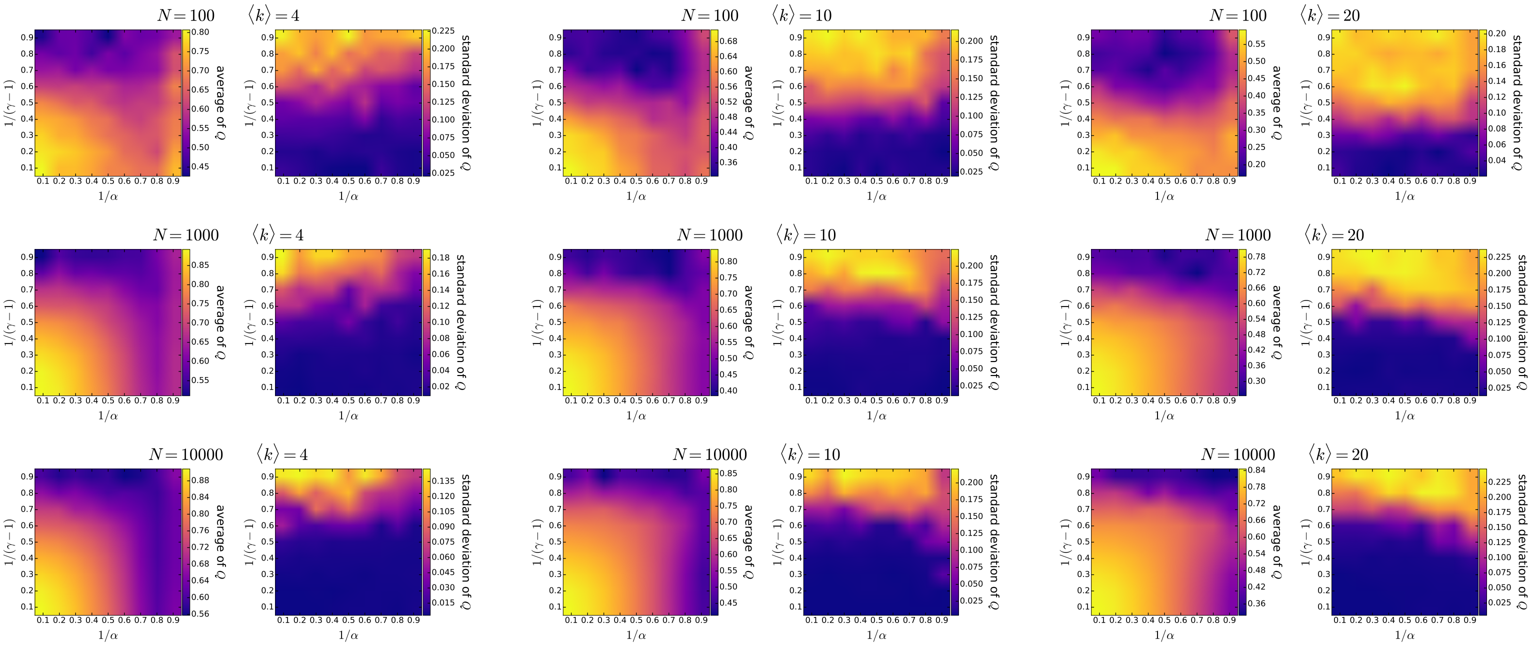

Figure B10. The mean and the standard deviation of the weighted modularity Q of the community structure detected by the *asynchronous label propagation* algorithm in 100 $\mathbb{S}^1/\mathbb{H}^2$ networks of different parametrisations. Each pair of subplots depicts the effect of changing $1/(\gamma-1)$ (equivalent to the popularity fading parameter β in the E-PSO model) and $1/\alpha$ (analogous to the temperature T in the E-PSO model), with the number of nodes N and the expected average degree $\langle k\rangle$ given in the title of the subplot pair. We used $K=-1$ as the curvature of the hyperbolic plane in each case.

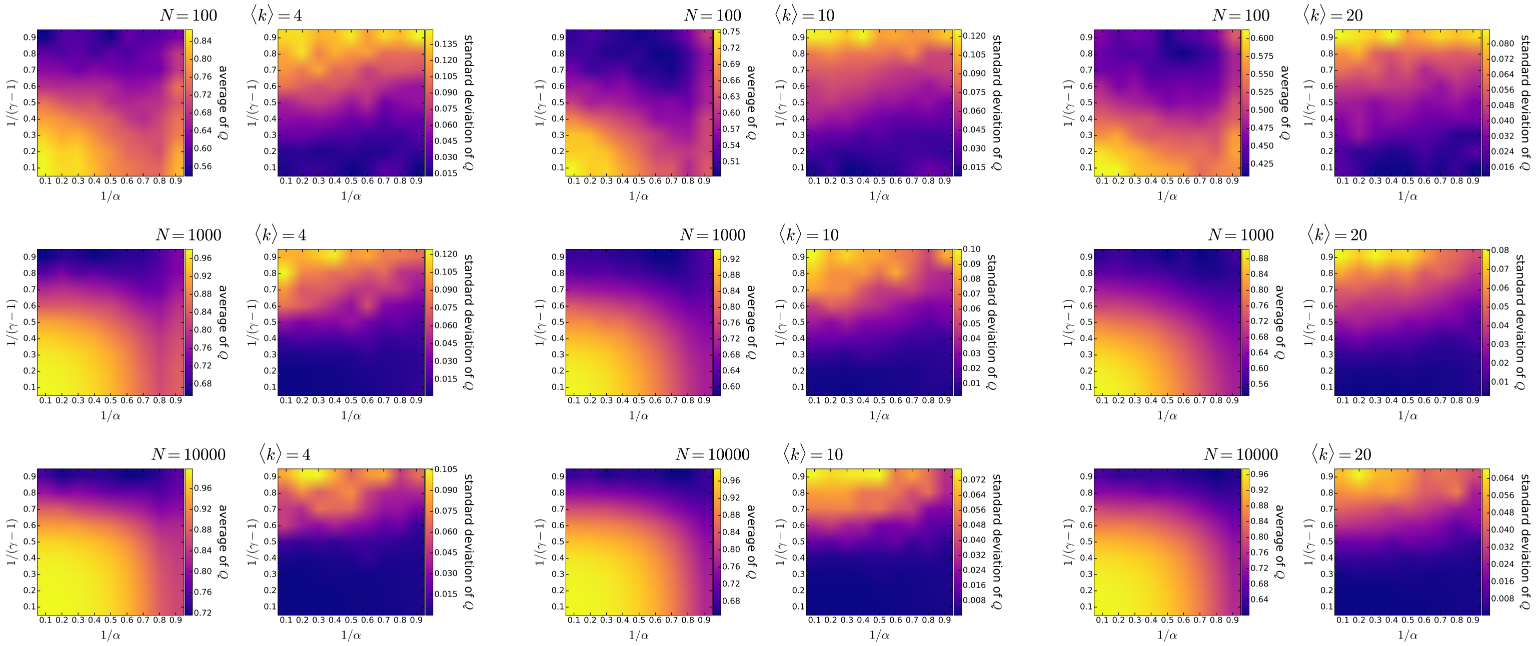

Figure B11. The mean and the standard deviation of the weighted modularity Q of the community structure detected by the *Louvain* algorithm in 100 $\mathbb{S}^1/\mathbb{H}^2$ networks of different parametrisations. Each pair of subplots depicts the effect of changing $1/(\gamma-1)$ (equivalent to the popularity fading parameter β in the E-PSO model) and $1/\alpha$ (analogous to the temperature T in the E-PSO model), with the number of nodes N and the expected average degree $\langle k \rangle$ given in the title of the subplot pair. We used $K = -1$ as the curvature of the hyperbolic plane in each case.

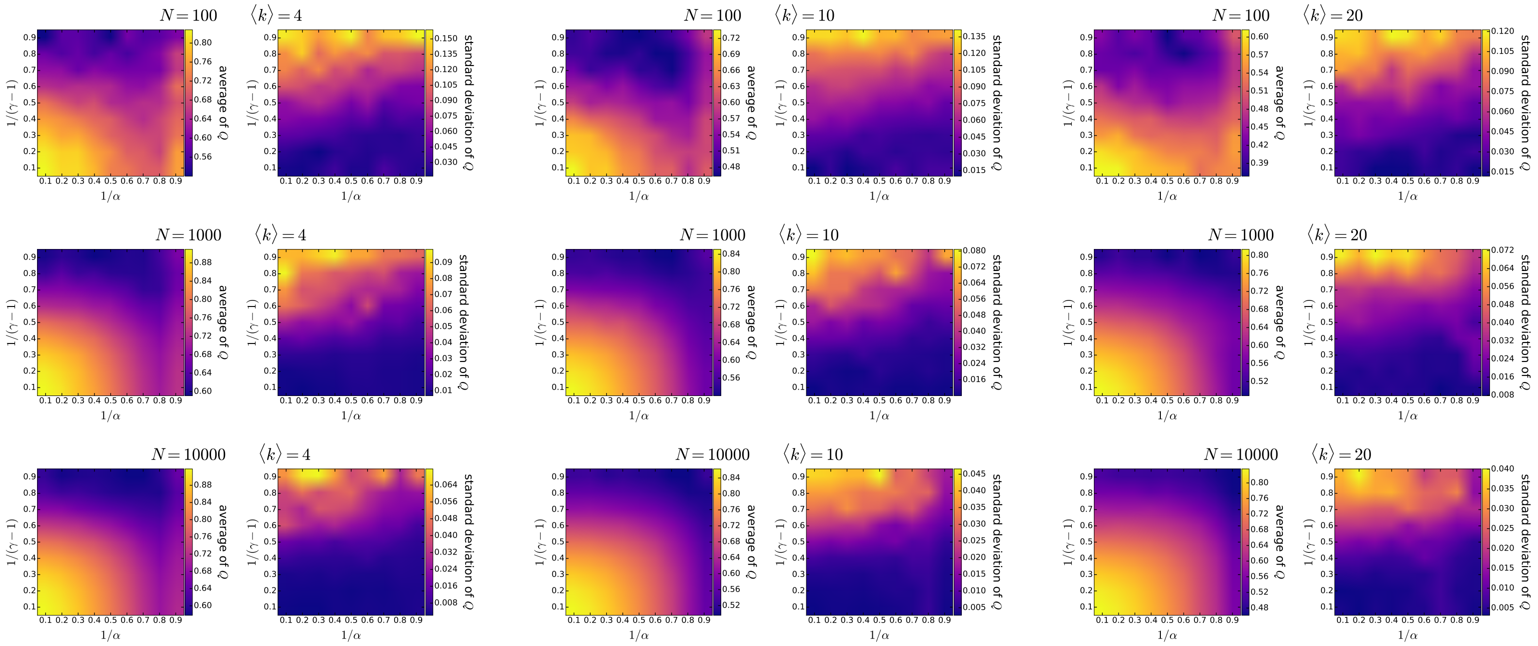

Figure B12. The mean and the standard deviation of the weighted modularity Q of the community structure detected by the *Infomap* algorithm in 100 $\mathbb{S}^1/\mathbb{H}^2$ networks of different parametrisations. Each pair of subplots depicts the effect of changing $1/(\gamma-1)$ (equivalent to the popularity fading parameter β in the E-PSO model) and $1/\alpha$ (analogous to the temperature T in the E-PSO model), with the number of nodes N and the expected average degree $\langle k \rangle$ given in the title of the subplot pair. We used $K = -1$ as the curvature of the hyperbolic plane in each case.

References

1. Raghavan, U. N., Albert, R. & Kumara, S. Near linear time algorithm to detect community structures in large-scale networks. *Phys. Rev. E* **76**, 036106, DOI: 10.1103/PhysRevE.76.036106 (2007).
2. We used the python function 'asyn_lpa_communities', an implementation of the asynchronous label propagation algorithm available in the 'networkx.algorithms.community.label_propagation' package.
3. Blondel, V. D., Guillaume, J.-L., Lambiotte, R. & Lefebvre, E. Fast unfolding of communities in large networks. *J. Stat. Mech. Theory Exp.* **2008**, P10008, DOI: 10.1088/1742-5468/2008/10/p10008 (2008).
4. We used the python implementation of the louvain algorithm available at https://github.com/taynaud/python-louvain. (Accessed: 14/07/2020).
5. Rosvall, M. & Bergstrom, C. T. Multilevel compression of random walks on networks reveals hierarchical organization in large integrated systems. *PLOS ONE* **6**, 1–10, DOI: 10.1371/journal.pone.0018209 (2011).
6. We used the python package for the infomap algorithm available at https://pypi.org/project/infomap/. (Accessed: 14/07/2020).
7. Papadopoulos, F., Kitsak, M., Serrano, M. Á., Boguñá, M. & Krioukov, D. Popularity versus similarity in growing networks. *Nature* **489**, 537 EP –, DOI: 10.1038/nature11459 (2012).
8. Papadopoulos, F., Psomas, C. & Krioukov, D. Network mapping by replaying hyperbolic growth. *IEEE/ACM Transactions on Netw.* **23**, 198–211, DOI: 10.1109/TNET.2013.2294052 (2015).
9. Kovács, B. & Palla, G. Optimisation of the coalescent hyperbolic embedding of complex networks (2020). Preprint at https://arXiv:2009.04702 [cs.SI].
10. Serrano, M. A., Krioukov, D. & Boguñá, M. Self-similarity of complex networks and hidden metric spaces. *Phys. Rev. Lett.* **100**, 078701, DOI: 10.1103/PhysRevLett.100.078701 (2008).
11. García-Pérez, G., Allard, A., Serrano, M. Á. & Boguñá, M. Mercator: uncovering faithful hyperbolic embeddings of complex networks. *New J. Phys.* **21**, 123033, DOI: 10.1088/1367-2630/ab57d2 (2019).
12. We used the c++ implementation of the $\mathbb{S}^1/\mathbb{H}^2$ model available at https://github.com/networkgeometry/mercator. (Accessed: 14/07/2020).
13. We calculated the modularity values with the python function 'modularity' available in the 'networkx.algorithms.community.quality' package.
