# Supplementary material for: The inherent community structure of hyperbolic networks
Source: Sci Rep. 2021 Aug 6;11:16050. doi: 10.1038/s41598-021-93921-2 (PMC8346486; doi:10.1038/s41598-021-93921-2)
Supplement: Supplementary file 3 — Supplementary Information 3. [file 41598_2021_93921_MOESM3_ESM.pdf]

The inherent community structure of hyperbolic networks
Supplementary C: Community size distributions

Bianka Kovács[1] **and Gergely Palla**[1,2,3,*]

[1]Dept. of Biological Physics, Eötvös Loránd University, H-1117 Budapest, Pázmány P. stny. 1/A, Hungary
[2]MTA-ELTE Statistical and Biological Physics Research Group, H-1117 Budapest, Pázmány P. stny. 1/A, Hungary
[3]Health Services Management Training Centre, Semmelweis University, H-1125 Budapest, Kútvölgyi út 2, Hungary.
*pallag@hal.elte.hu

Here we present the characteristics of the community size distributions obtained with the asynchronous label propagation[1,2], the Louvain[3,4] and the Infomap[5,6] algorithms for the PSO[7], E-PSO[8,9] and $\mathbb{S}^1/\mathbb{H}^2$[10–12] networks of various parameter combinations. Each community detection algorithm was executed once for each network. The isolated nodes emerging in the case of the $\mathbb{S}^1/\mathbb{H}^2$ model and occasionally also in the networks generated by the E-PSO model of $L<0$ were removed before the community detection, meaning that the actual size of the examined networks does not necessarily reach the number of nodes N inputted in these models. We generated 100 networks with each parametrisation and investigated the sample created by assembling the occurring community sizes from all the 100 networks. Figs. C1–C18 display how the mean and the standard deviation of this sample depend on the network generation parameters, as well as some examples for the corresponding community size distributions. Figs. C1–C6 deal with the E-PSO model with $L=0$ (i.e. the PSO model), Figs. C7–C12 show the effect of changing the parameter L, and Figs. C13–C18 refers to the $\mathbb{S}^1/\mathbb{H}^2$ model. The community size distribution is typically bell-shaped according to the Louvain algorithm, whereas rather skewed according to the Infomap and the asynchronous label propagation algorithms. In the parameter regime where we observed low Q values, the community finding methods tend to merge the nodes into large communities of sizes comparable with N.

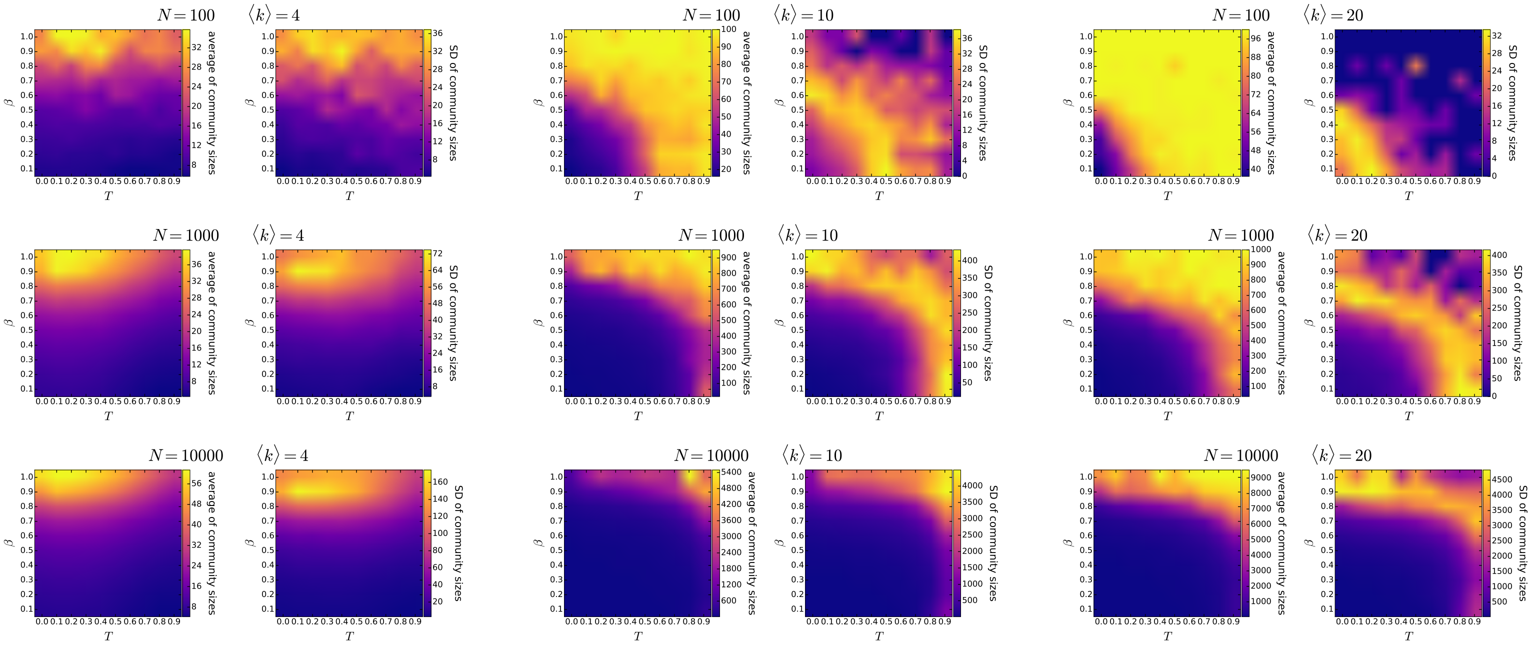

Figure C1. The mean and the standard deviation of the size of communities detected by the *asynchronous label propagation* algorithm in 100 *PSO* networks of different parametrisations. Each pair of subplots depicts the effect of changing the popularity fading parameter β and the temperature T, with the number of nodes N and the expected average degree $\langle k\rangle = 2m$ given in the title of the subplot pair. The curvature of the hyperbolic plane K was always set to -1, i.e. we used $\zeta = 1$.

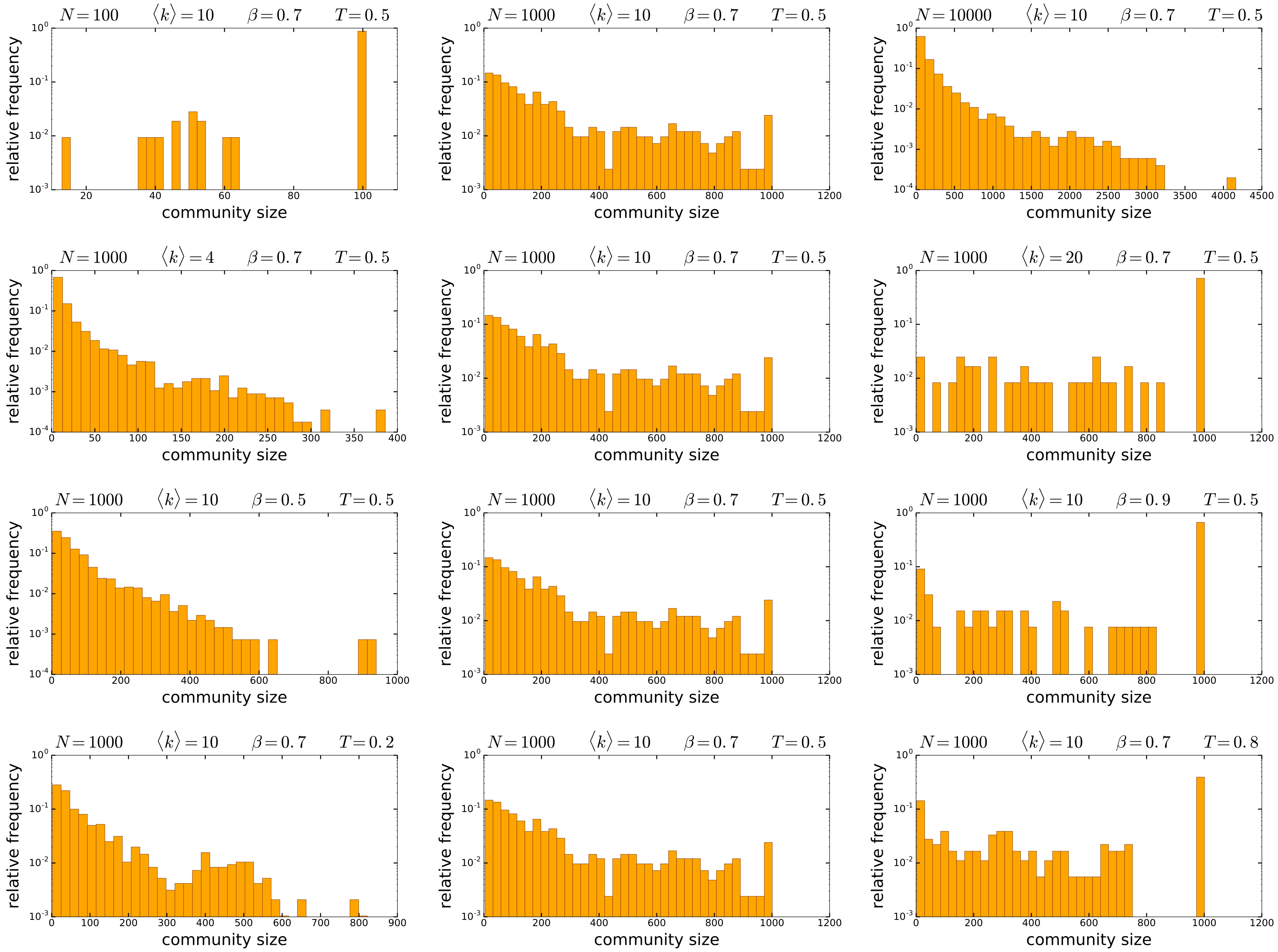

Figure C2. The size distribution of the communities detected by the *asynchronous label propagation* algorithm in 100 *PSO* networks of different parametrisations. The parameters of the network generation are listed in the title for each subplot. The curvature of the hyperbolic plane K was always set to -1, i.e. we used $\zeta = 1$. Each row of the figure demonstrates the effect of the change in a given network generation parameter: from top to bottom, the number of nodes N, the expected average degree $\langle k \rangle = 2m$, the popularity fading parameter β and the temperature T.

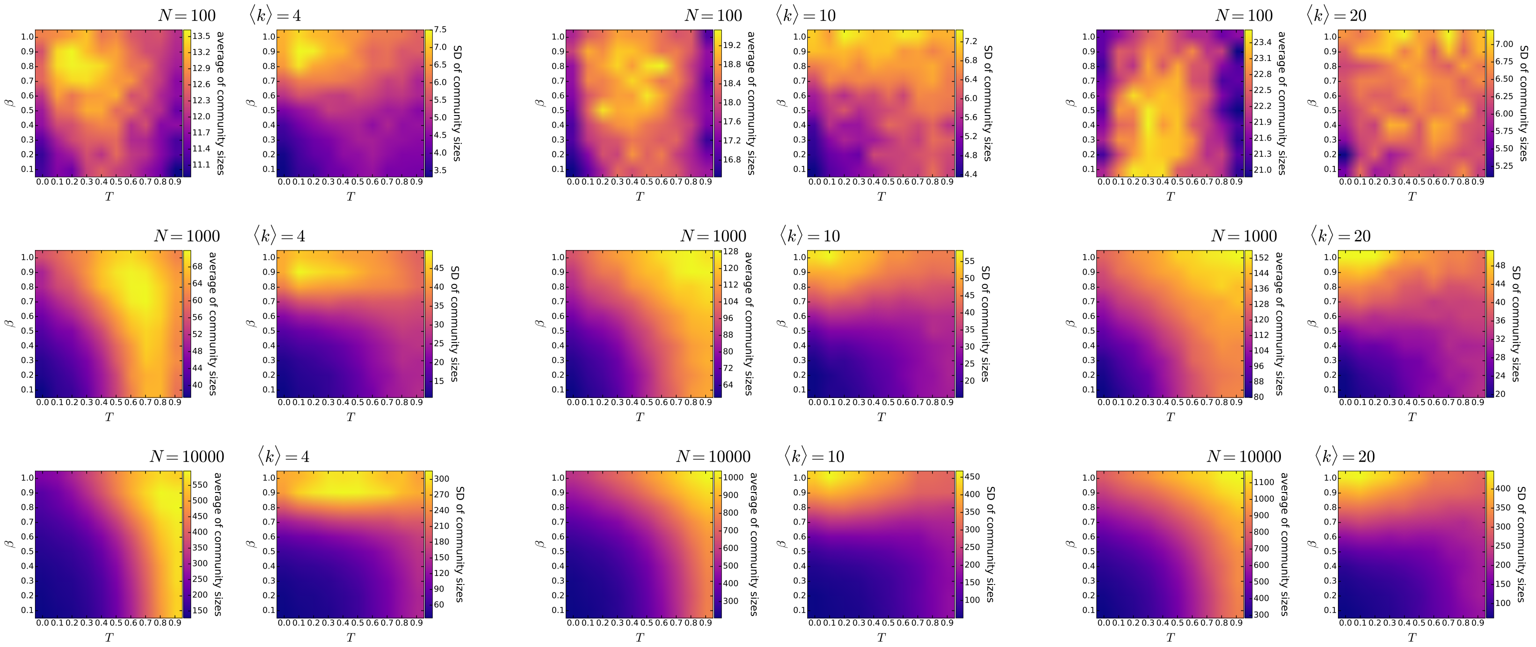

Figure C3. The mean and the standard deviation of the size of communities detected by the *Louvain* algorithm in 100 *PSO* networks of different parametrisations. Each pair of subplots depicts the effect of changing the popularity fading parameter β and the temperature T, with the number of nodes N and the expected average degree $\langle k \rangle = 2m$ given in the title of the subplot pair. The curvature of the hyperbolic plane K was always set to -1, i.e. we used $\zeta = 1$.

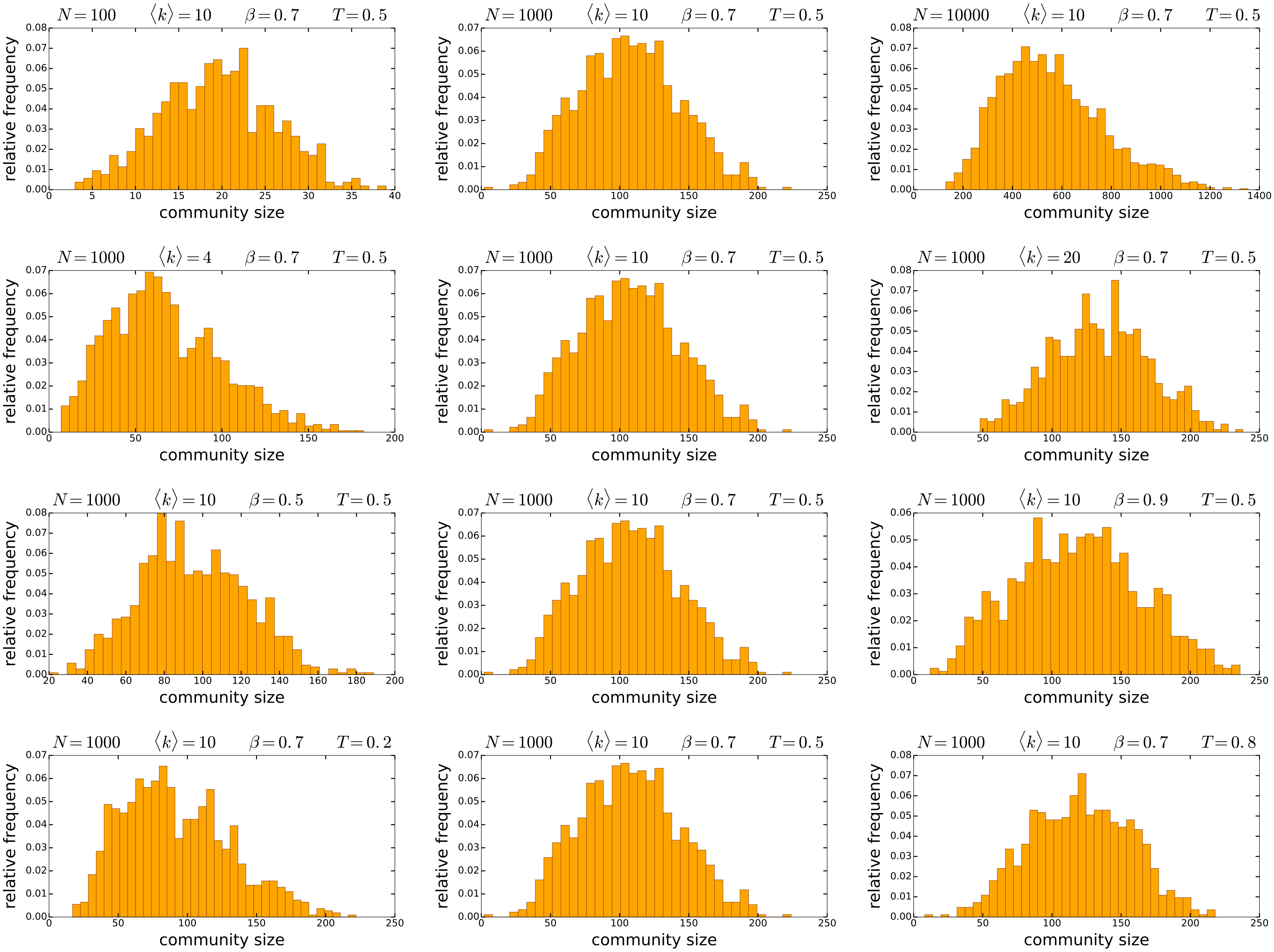

Figure C4. The size distribution of the communities detected by the *Louvain* algorithm in 100 *PSO* networks of different parametrisations. The parameters of the network generation are listed in the title for each subplot. The curvature of the hyperbolic plane K was always set to -1, i.e. we used $\zeta = 1$. Each row of the figure demonstrates the effect of the change in a given network generation parameter: from top to bottom, the number of nodes N, the expected average degree $\langle k \rangle = 2m$, the popularity fading parameter β and the temperature T.

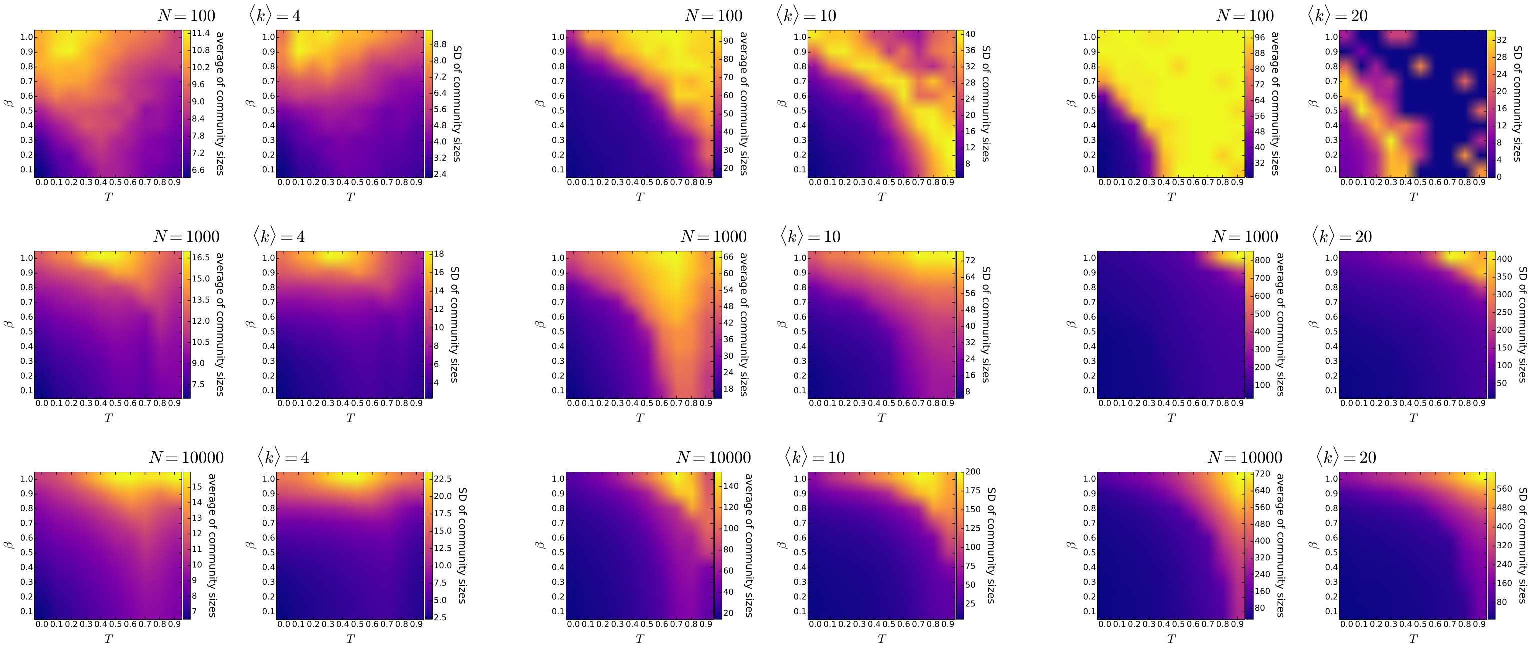

Figure C5. The mean and the standard deviation of the size of communities detected by the *Infomap* algorithm in 100 *PSO* networks of different parametrisations. Each pair of subplots depicts the effect of changing the popularity fading parameter β and the temperature T, with the number of nodes N and the expected average degree $\langle k \rangle = 2m$ given in the title of the subplot pair. The curvature of the hyperbolic plane K was always set to -1, i.e. we used $\zeta = 1$.

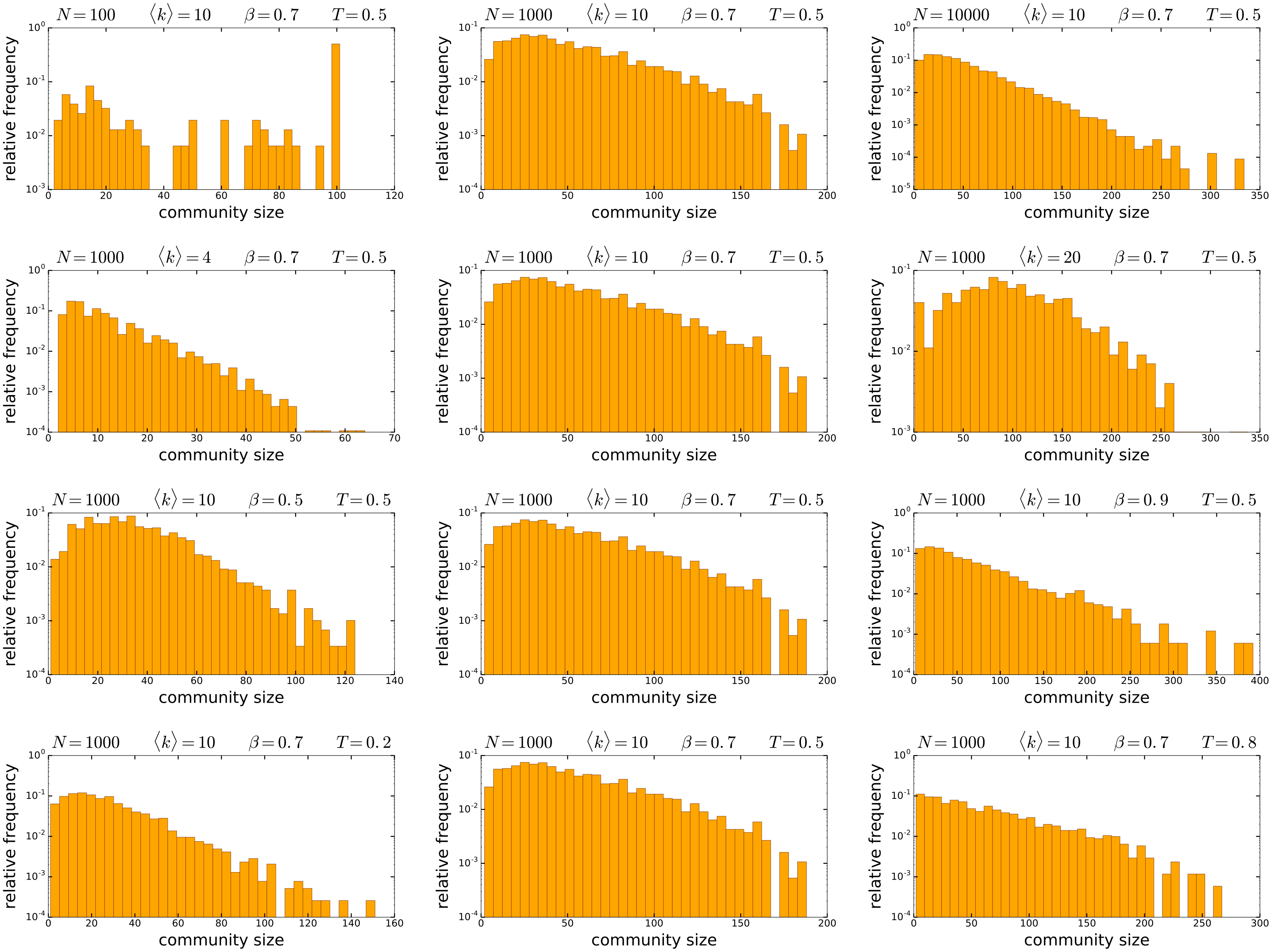

Figure C6. The size distribution of the communities detected by the *Infomap* algorithm in 100 *PSO* networks of different parametrisations. The parameters of the network generation are listed in the title for each subplot. The curvature of the hyperbolic plane K was always set to -1, i.e. we used $\zeta = 1$. Each row of the figure demonstrates the effect of the change in a given network generation parameter: from top to bottom, the number of nodes N, the expected average degree $\langle k \rangle = 2m$, the popularity fading parameter β and the temperature T.

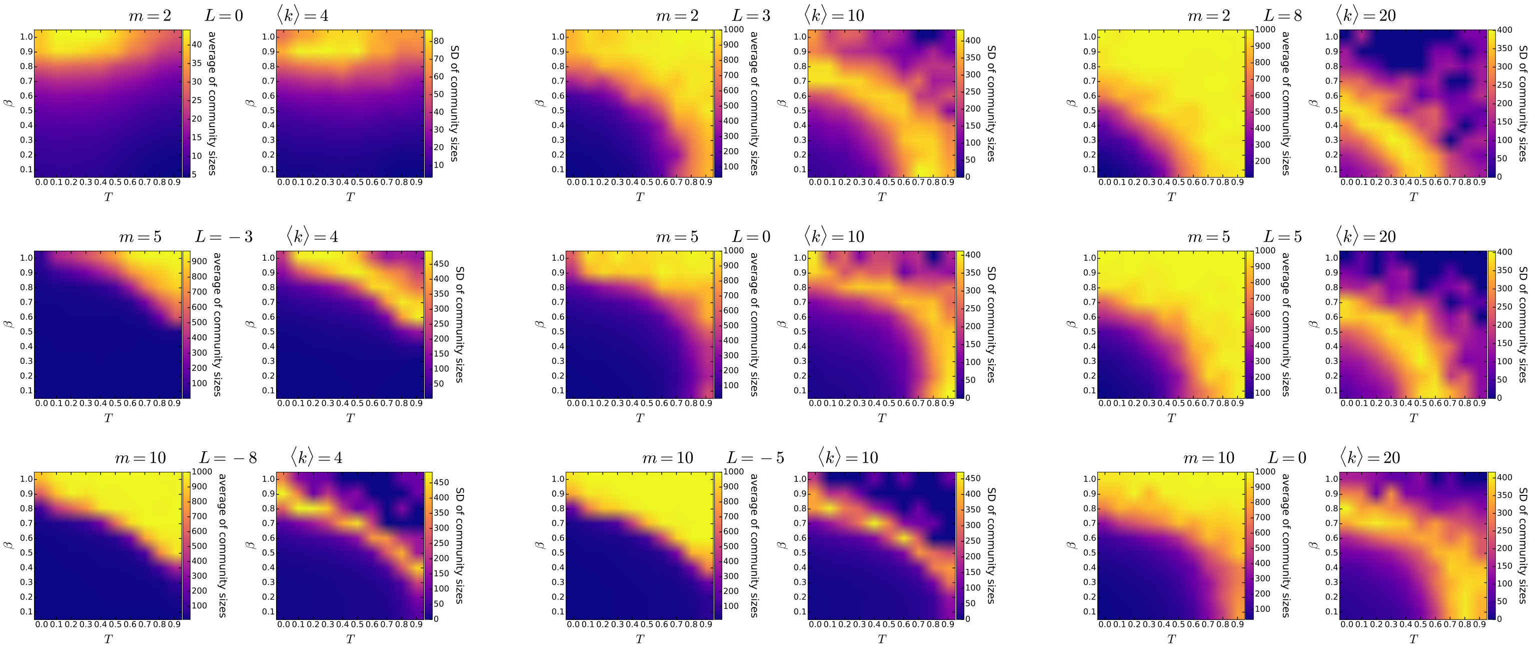

Figure C7. The mean and the standard deviation of the size of communities detected by the *asynchronous label propagation* algorithm in 100 *E-PSO* networks of different parametrisations. Each pair of subplots depicts the effect of changing the popularity fading parameter β and the temperature T, with the parameters m and L given in the title of the subplot pair together with the corresponding expected average degree $\langle k \rangle = 2(m+L)$. The number of nodes N was 1000 in each case. The curvature of the hyperbolic plane K was always set to -1, i.e. we used $\zeta = 1$.

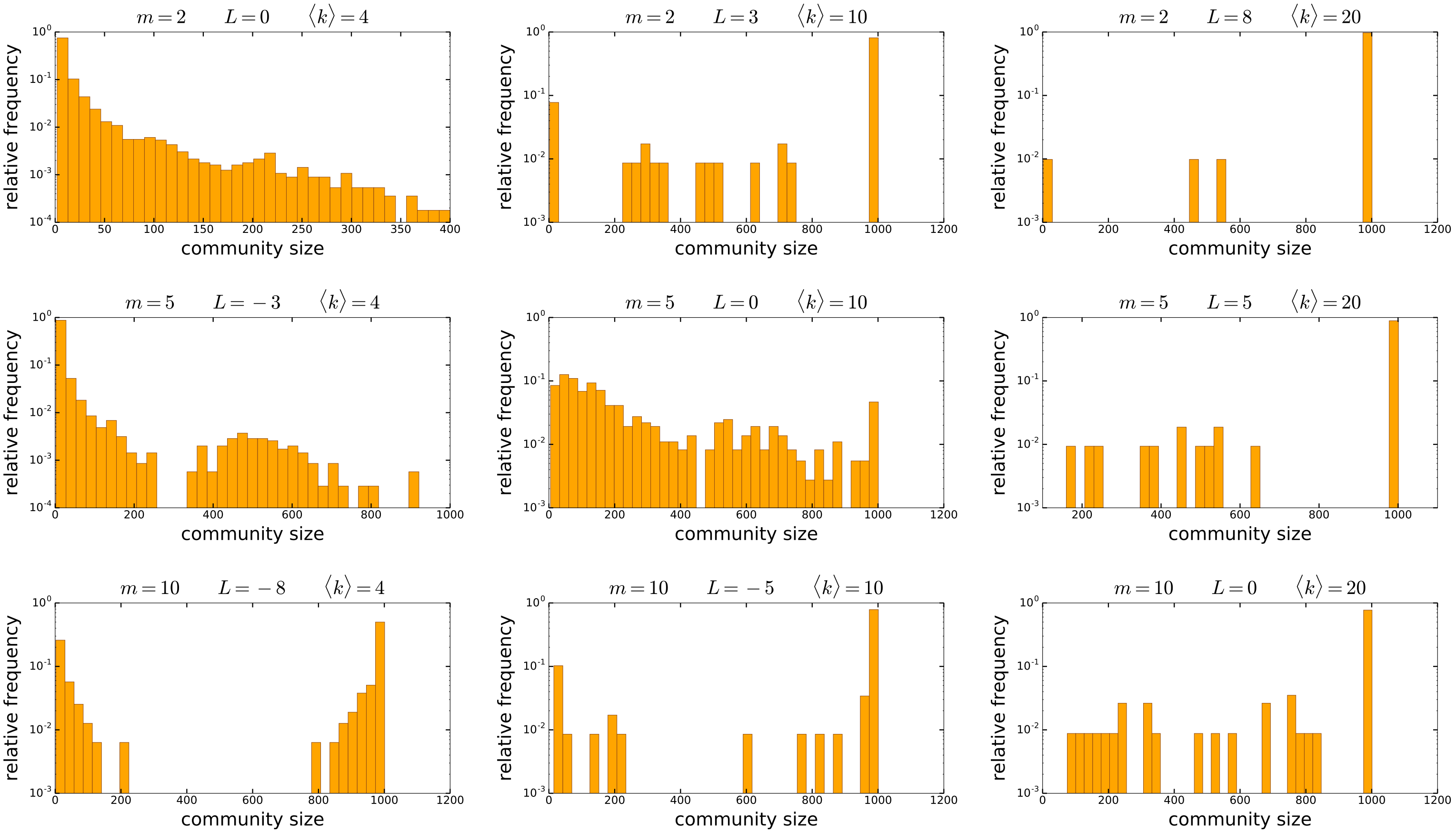

Figure C8. The size distribution of the communities detected by the *asynchronous label propagation* algorithm in 100 *E-PSO* networks of different parametrisations. We used $\zeta = 1$, i.e. $K = -1$ as the curvature of the hyperbolic plane, the number of nodes N was 1000, the popularity fading parameter β was 0.7 and the temperature T was 0.5 in each case. The parameters m and L are given in the title for each subplot together with the corresponding expected average degree $\langle k\rangle = 2(m+L)$.

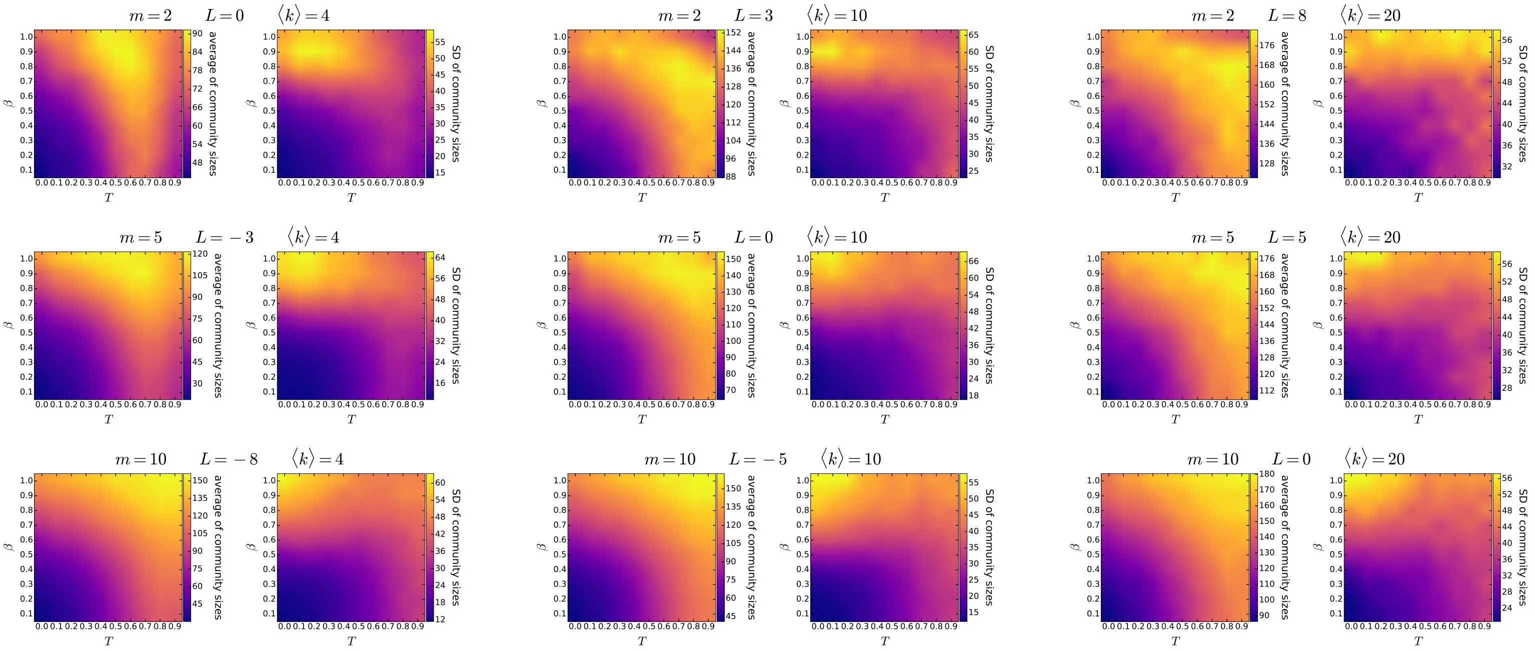

Figure C9. The mean and the standard deviation of the size of communities detected by the *Louvain* algorithm in 100 *E-PSO* networks of different parametrisations. Each pair of subplots depicts the effect of changing the popularity fading parameter β and the temperature T, with the parameters m and L given in the title of the subplot pair together with the corresponding expected average degree $\langle k\rangle = 2(m+L)$. The number of nodes N was 1000 in each case. The curvature of the hyperbolic plane K was always set to -1, i.e. we used $\zeta = 1$.

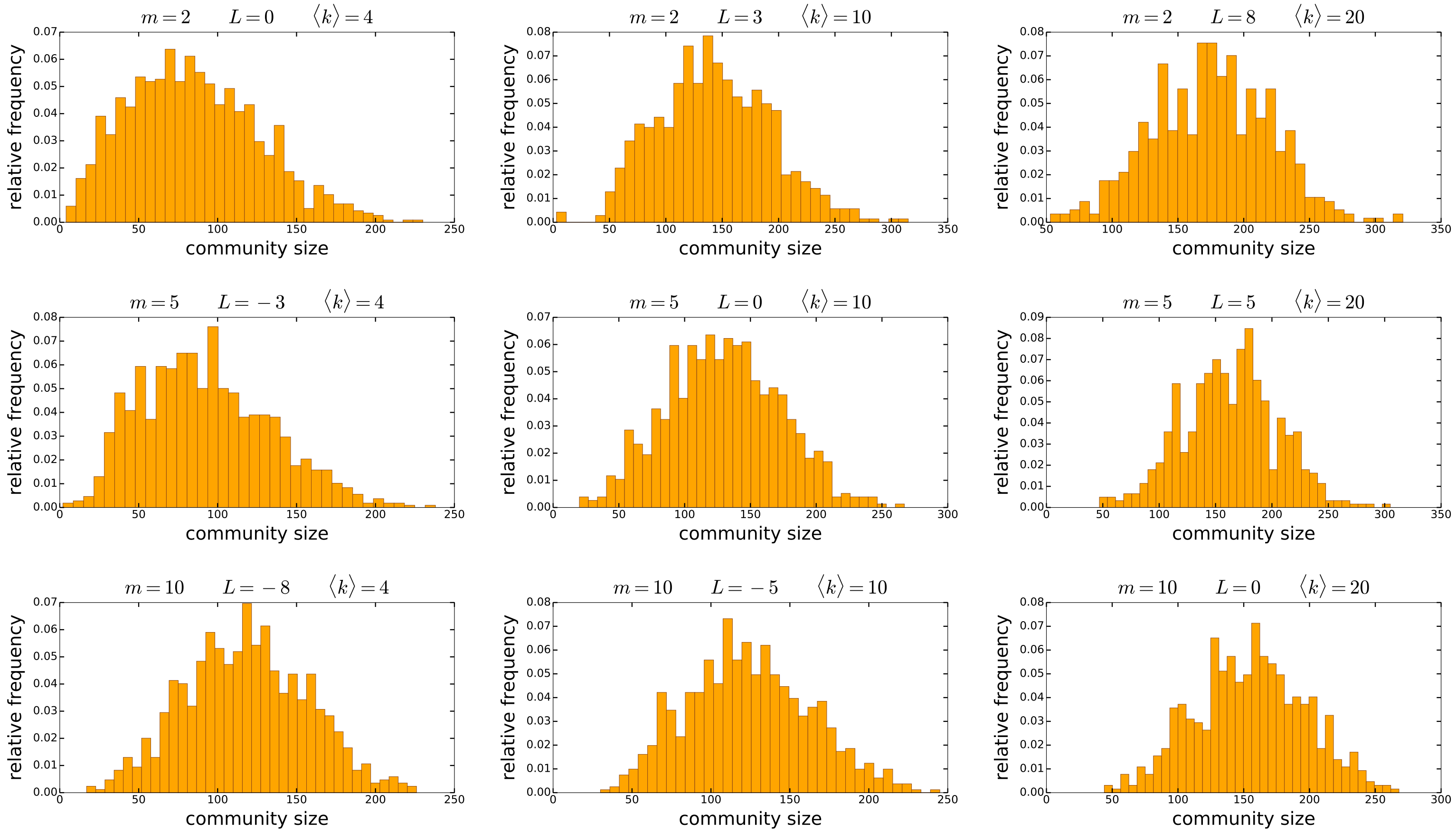

Figure C10. The size distribution of the communities detected by the *Louvain* algorithm in 100 *E-PSO* networks of different parametrisations. We used $\zeta = 1$, i.e. $K = -1$ as the curvature of the hyperbolic plane, the number of nodes N was 1000, the popularity fading parameter β was 0.7 and the temperature T was 0.5 in each case. The parameters m and L are given in the title for each subplot together with the corresponding expected average degree $\langle k \rangle = 2(m+L)$.

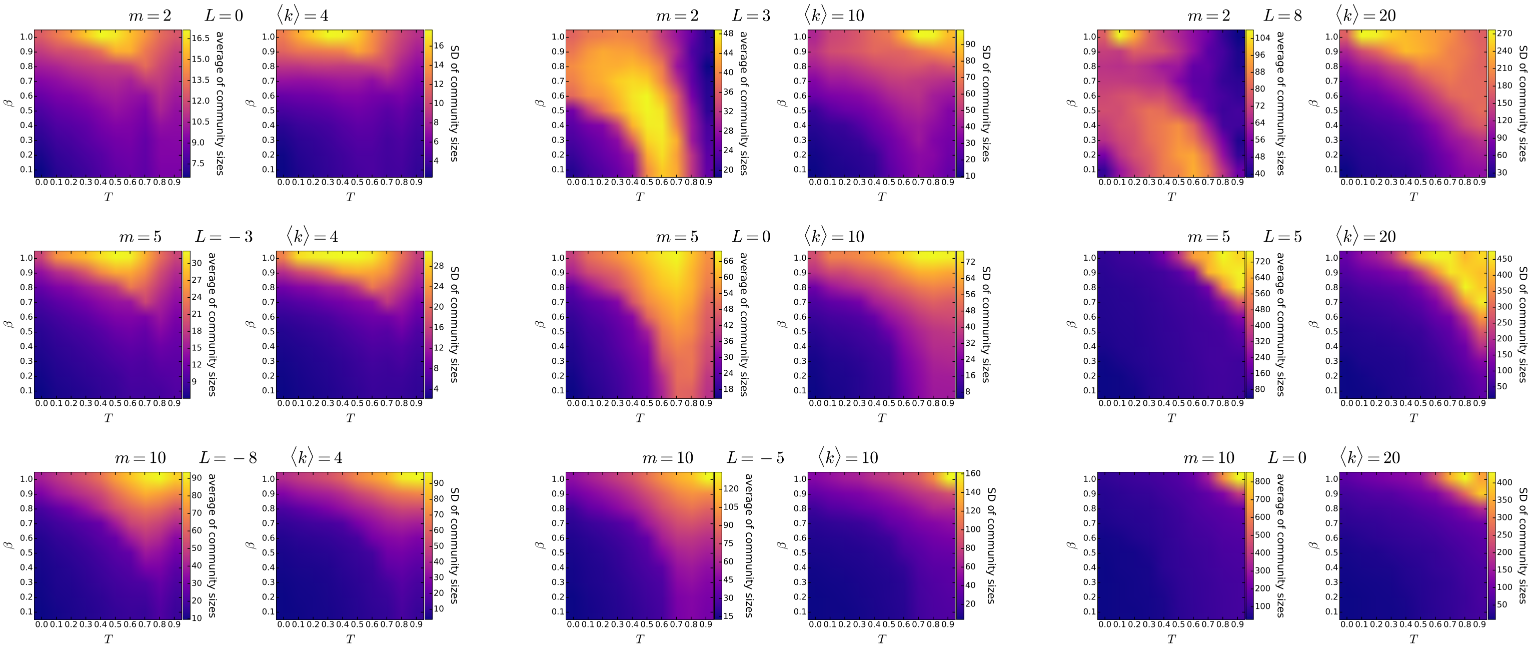

Figure C11. The mean and the standard deviation of the size of communities detected by the *Infomap* algorithm in 100 *E-PSO* networks of different parametrisations. Each pair of subplots depicts the effect of changing the popularity fading parameter β and the temperature T, with the parameters m and L given in the title of the subplot pair together with the corresponding expected average degree $\langle k \rangle = 2(m+L)$. The number of nodes N was 1000 in each case. The curvature of the hyperbolic plane K was always set to -1, i.e. we used $\zeta = 1$.

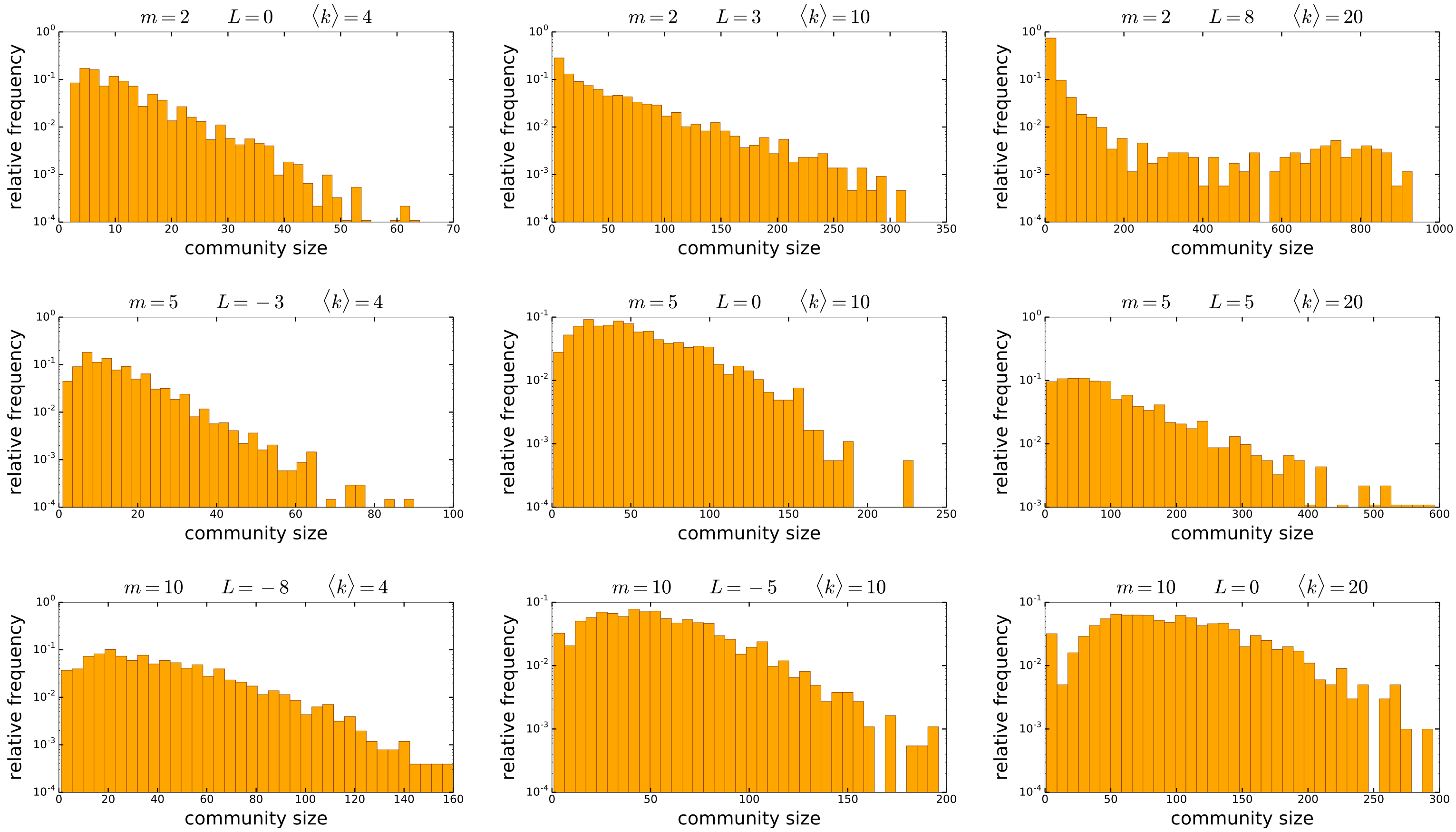

Figure C12. The size distribution of the communities detected by the *Infomap* algorithm in 100 *E-PSO* networks of different parametrisations. We used $\zeta = 1$, i.e. $K = -1$ as the curvature of the hyperbolic plane, the number of nodes N was 1000, the popularity fading parameter β was 0.7 and the temperature T was 0.5 in each case. The parameters m and L are given in the title for each subplot together with the corresponding expected average degree $\langle k \rangle = 2(m+L)$.

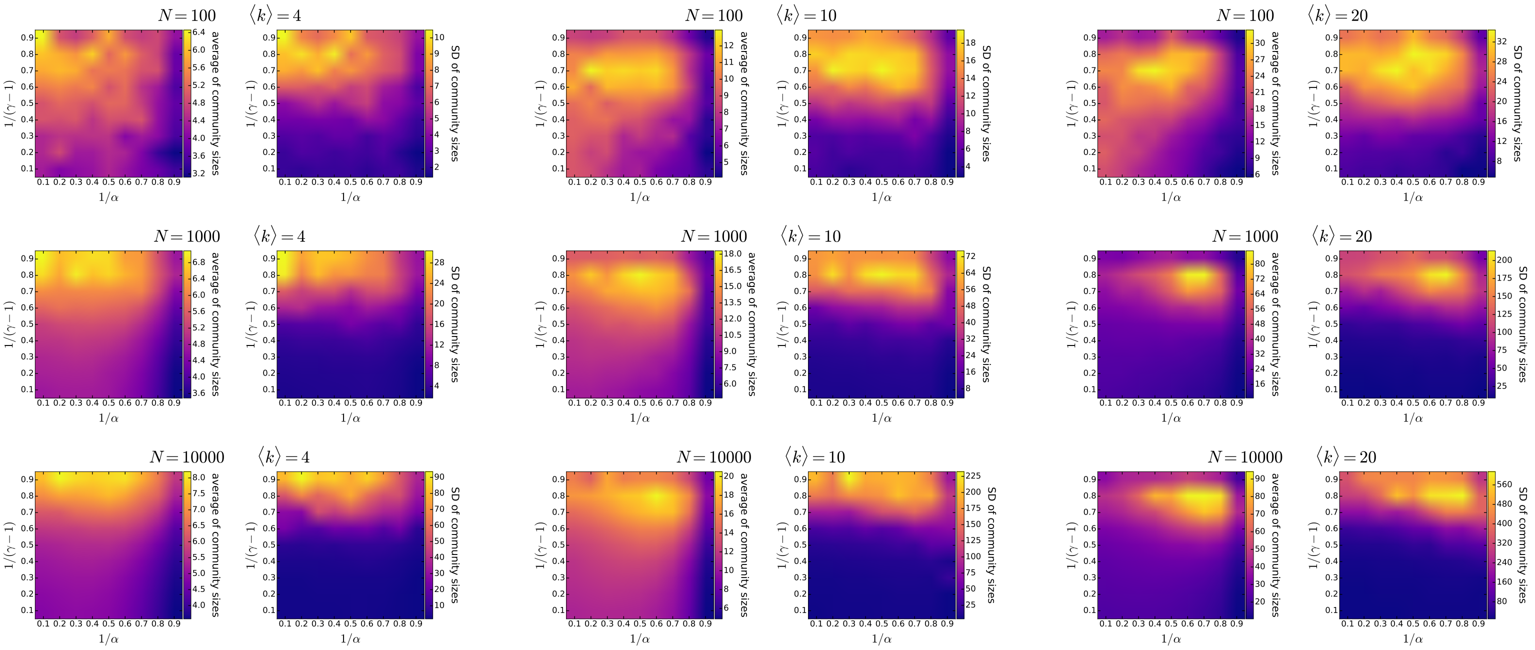

Figure C13. The mean and the standard deviation of the size of communities detected by the *asynchronous label propagation* algorithm in 100 $\mathbb{S}^1/\mathbb{H}^2$ networks of different parametrisations. Each pair of subplots depicts the effect of changing $1/(\gamma-1)$ (equivalent to the popularity fading parameter β in the E-PSO model) and $1/\alpha$ (analogous to the temperature T in the E-PSO model), with the number of nodes N and the expected average degree $\langle k \rangle$ given in the title of the subplot pair. We used $K = -1$ as the curvature of the hyperbolic plane in each case.

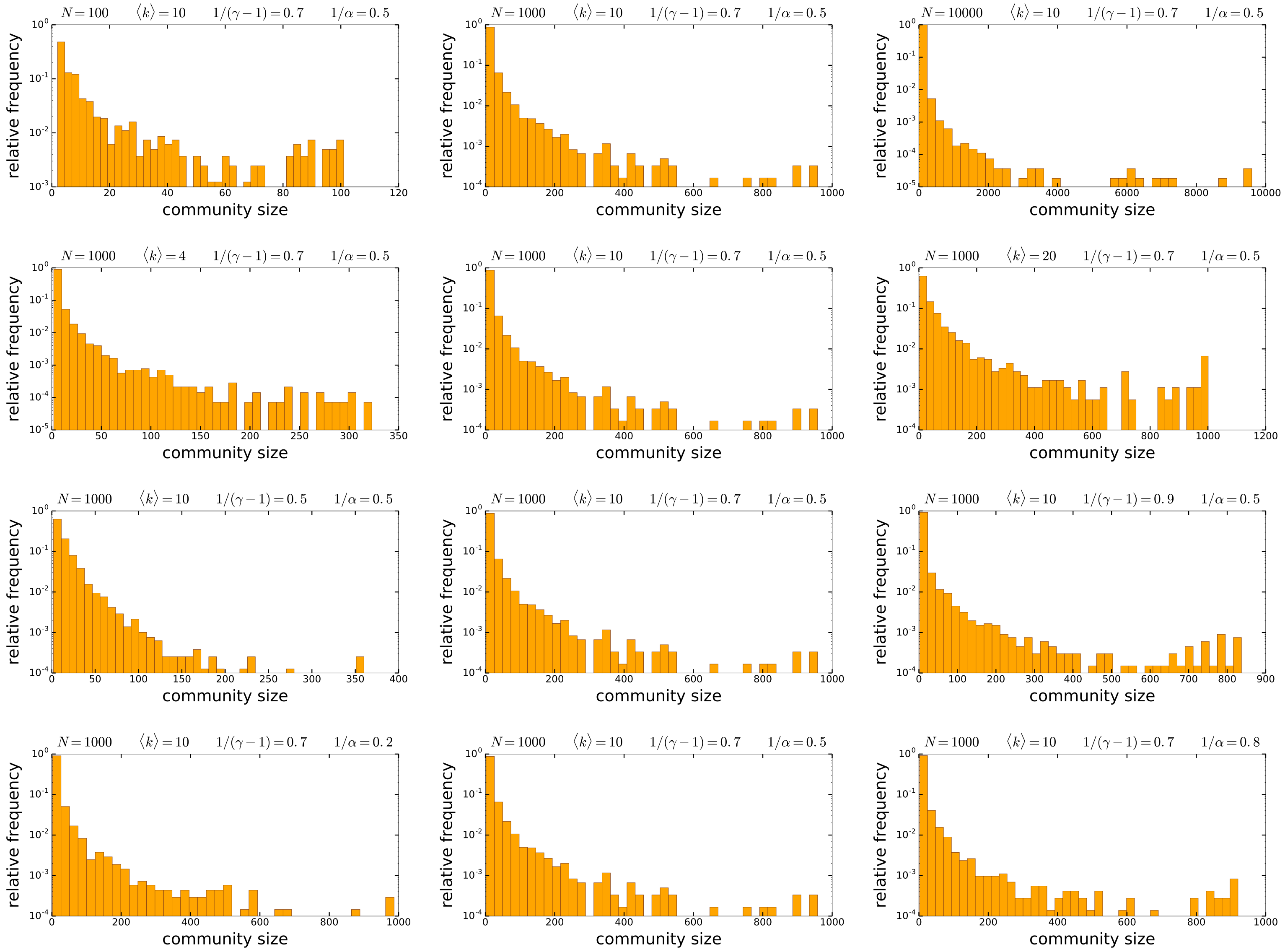

Figure C14. The size distribution of the communities detected by the *asynchronous label propagation* algorithm in 100 $\mathbb{S}^1/\mathbb{H}^2$ networks of different parametrisations. The parameters of the network generation are listed in the title for each subplot. We used $K=-1$ as the curvature of the hyperbolic plane in each case. Each row of the figure demonstrates the effect of the change in a given network generation parameter: from top to bottom, the number of nodes N, the expected average degree $\langle k\rangle$, $1/(\gamma-1)$ (equivalent to the popularity fading parameter β in the E-PSO model) and $1/\alpha$ (analogous to the temperature T in the E-PSO model).

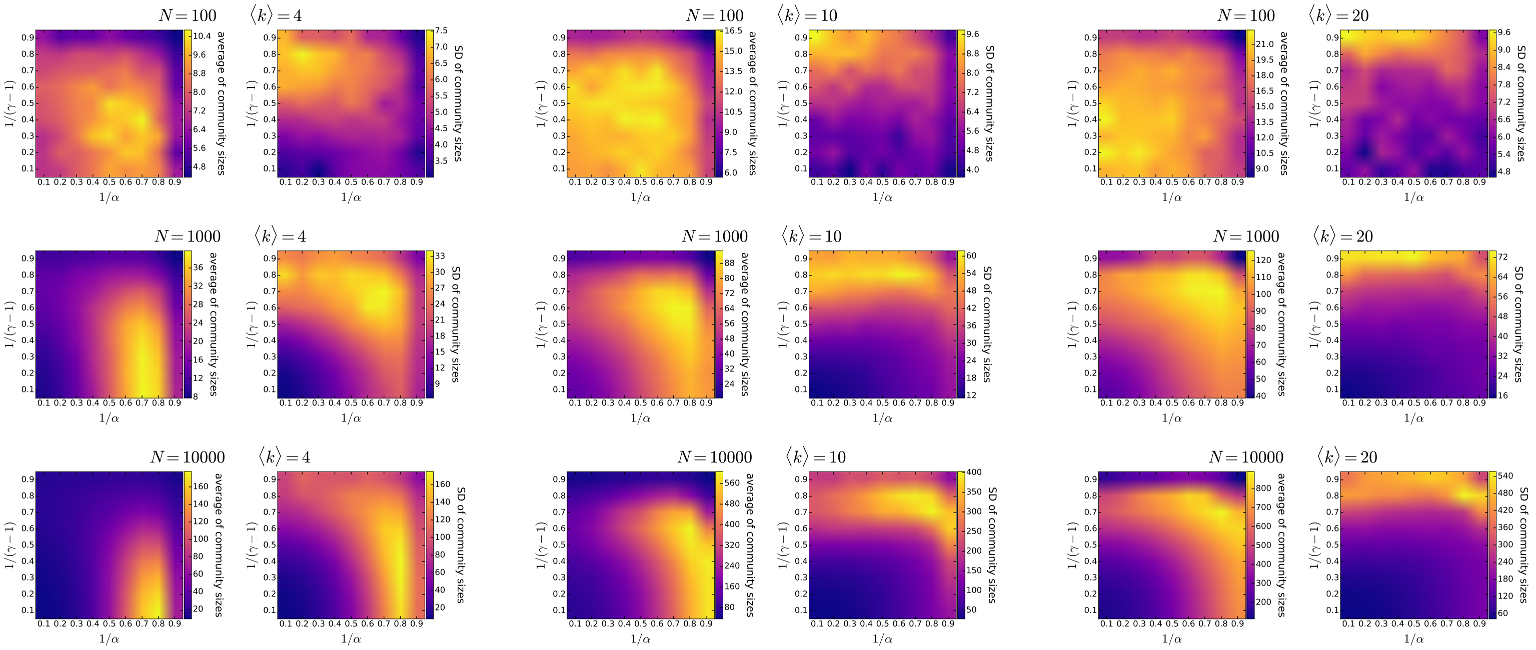

Figure C15. The mean and the standard deviation of the size of communities detected by the *Louvain* algorithm in 100 $\mathbb{S}^1/\mathbb{H}^2$ networks of different parametrisations. Each pair of subplots depicts the effect of changing $1/(\gamma-1)$ (equivalent to the popularity fading parameter β in the E-PSO model) and $1/\alpha$ (analogous to the temperature T in the E-PSO model), with the number of nodes N and the expected average degree $\langle k\rangle$ given in the title of the subplot pair. We used $K=-1$ as the curvature of the hyperbolic plane in each case.

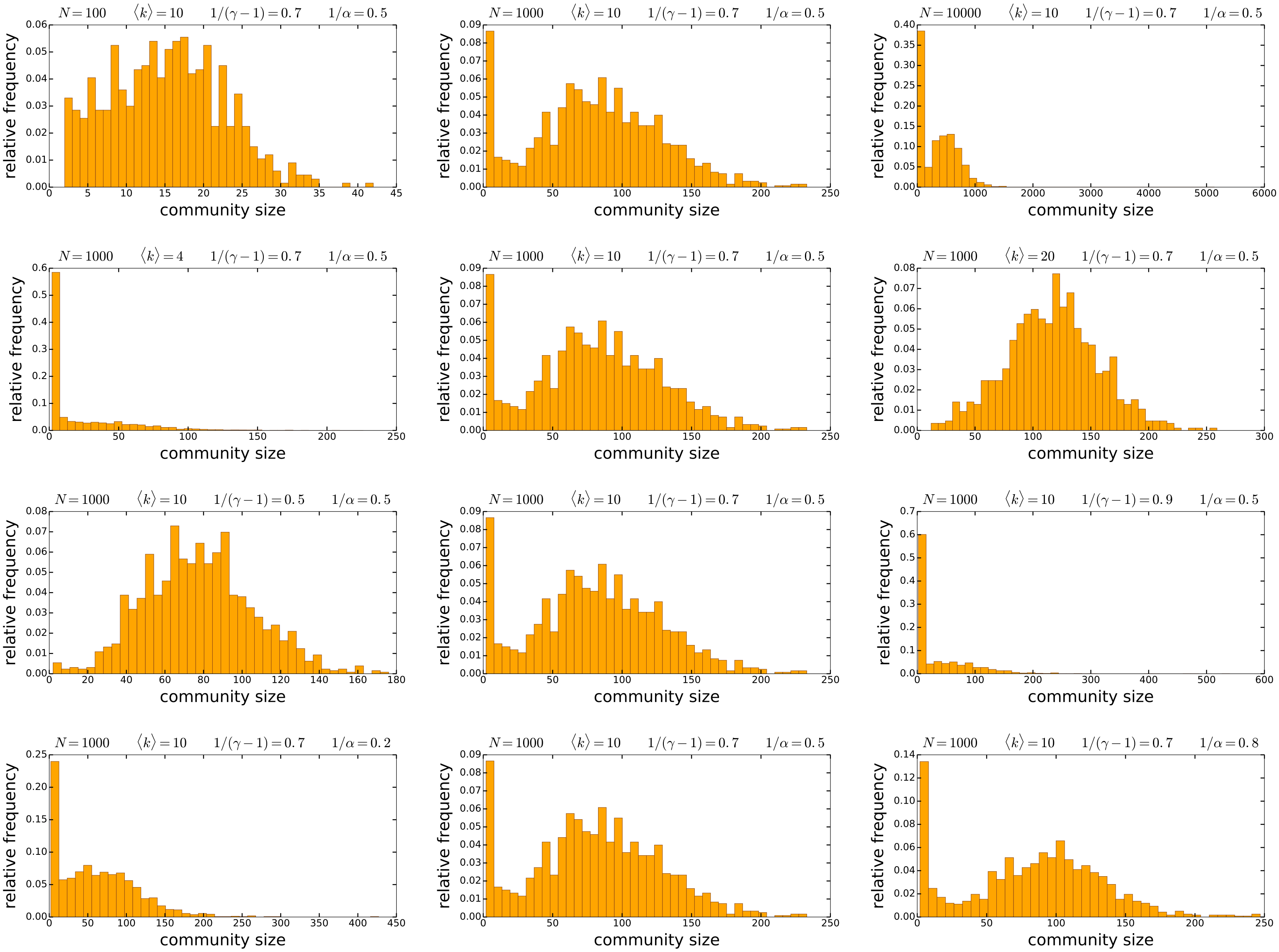

Figure C16. The size distribution of the communities detected by the *Louvain* algorithm in 100 $\mathbb{S}^1/\mathbb{H}^2$ networks of different parametrisations. The parameters of the network generation are listed in the title for each subplot. We used $K = -1$ as the curvature of the hyperbolic plane in each case. Each row of the figure demonstrates the effect of the change in a given network generation parameter: from top to bottom, the number of nodes N, the expected average degree $\langle k \rangle$, $1/(\gamma - 1)$ (equivalent to the popularity fading parameter β in the E-PSO model) and $1/\alpha$ (analogous to the temperature T in the E-PSO model).

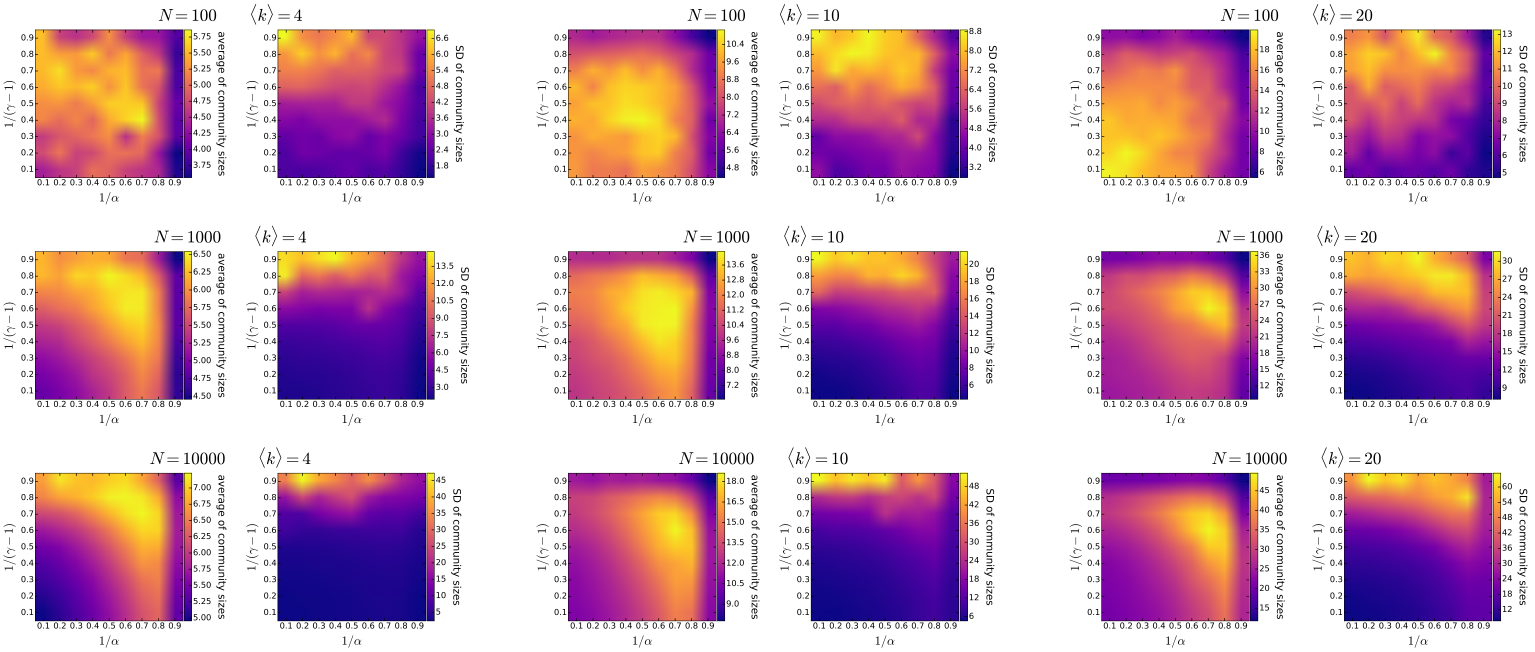

Figure C17. The mean and the standard deviation of the size of communities detected by the *Infomap* algorithm in 100 $\mathbb{S}^1/\mathbb{H}^2$ networks of different parametrisations. Each pair of subplots depicts the effect of changing $1/(\gamma-1)$ (equivalent to the popularity fading parameter β in the E-PSO model) and $1/\alpha$ (analogous to the temperature T in the E-PSO model), with the number of nodes N and the expected average degree $\langle k \rangle$ given in the title of the subplot pair. We used $K = -1$ as the curvature of the hyperbolic plane in each case.

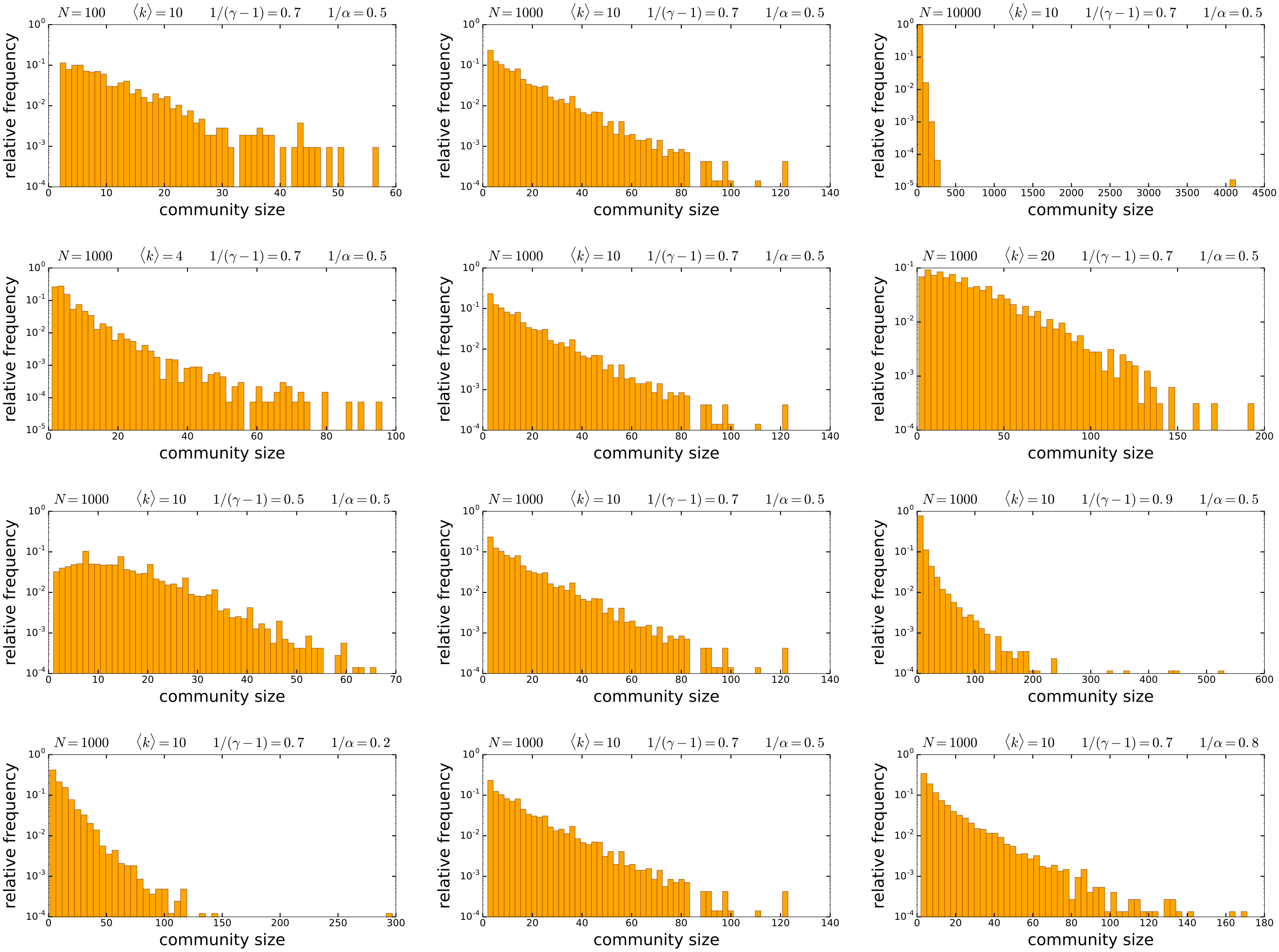

Figure C18. The size distribution of the communities detected by the *Infomap* algorithm in 100 $\mathbb{S}^1/\mathbb{H}^2$ networks of different parametrisations. The parameters of the network generation are listed in the title for each subplot. We used $K=-1$ as the curvature of the hyperbolic plane in each case. Each row of the figure demonstrates the effect of the change in a given network generation parameter: from top to bottom, the number of nodes N, the expected average degree $\langle k\rangle$, $1/(\gamma-1)$ (equivalent to the popularity fading parameter β in the E-PSO model) and $1/\alpha$ (analogous to the temperature T in the E-PSO model).

References

1. Raghavan, U. N., Albert, R. & Kumara, S. Near linear time algorithm to detect community structures in large-scale networks. *Phys. Rev. E* **76**, 036106, DOI: 10.1103/PhysRevE.76.036106 (2007).
2. We used the python function 'asyn_lpa_communities', an implementation of the asynchronous label propagation algorithm available in the 'networkx.algorithms.community.label_propagation' package.
3. Blondel, V. D., Guillaume, J.-L., Lambiotte, R. & Lefebvre, E. Fast unfolding of communities in large networks. *J. Stat. Mech. Theory Exp.* **2008**, P10008, DOI: 10.1088/1742-5468/2008/10/p10008 (2008).
4. We used the python implementation of the louvain algorithm available at https://github.com/taynaud/python-louvain. (Accessed: 14/07/2020).
5. Rosvall, M. & Bergstrom, C. T. Multilevel compression of random walks on networks reveals hierarchical organization in large integrated systems. *PLOS ONE* **6**, 1–10, DOI: 10.1371/journal.pone.0018209 (2011).
6. We used the python package for the infomap algorithm available at https://pypi.org/project/infomap/. (Accessed: 14/07/2020).
7. Papadopoulos, F., Kitsak, M., Serrano, M. Á., Boguñá, M. & Krioukov, D. Popularity versus similarity in growing networks. *Nature* **489**, 537 EP –, DOI: 10.1038/nature11459 (2012).
8. Papadopoulos, F., Psomas, C. & Krioukov, D. Network mapping by replaying hyperbolic growth. *IEEE/ACM Transactions on Netw.* **23**, 198–211, DOI: 10.1109/TNET.2013.2294052 (2015).
9. Kovács, B. & Palla, G. Optimisation of the coalescent hyperbolic embedding of complex networks (2020). Preprint at https://arXiv:2009.04702 [cs.SI].
10. Serrano, M. A., Krioukov, D. & Boguñá, M. Self-similarity of complex networks and hidden metric spaces. *Phys. Rev. Lett.* **100**, 078701, DOI: 10.1103/PhysRevLett.100.078701 (2008).
11. García-Pérez, G., Allard, A., Serrano, M. Á. & Boguñá, M. Mercator: uncovering faithful hyperbolic embeddings of complex networks. *New J. Phys.* **21**, 123033, DOI: 10.1088/1367-2630/ab57d2 (2019).
12. We used the c++ implementation of the $\mathbb{S}^1/\mathbb{H}^2$ model available at https://github.com/networkgeometry/mercator. (Accessed: 14/07/2020).
