# Supplementary material for: The inherent community structure of hyperbolic networks
Source: Sci Rep. 2021 Aug 6;11:16050. doi: 10.1038/s41598-021-93921-2 (PMC8346486; doi:10.1038/s41598-021-93921-2)
Supplement: Supplementary file 4 — Supplementary Information 4. [file 41598_2021_93921_MOESM4_ESM.pdf]

The inherent community structure of hyperbolic networks
Supplementary D: Adjusted mutual information of the community structures found by different community detection algorithms

Bianka Kovács[1] **and Gergely Palla**[1,2,3,*]

[1]Dept. of Biological Physics, Eötvös Loránd University, H-1117 Budapest, Pázmány P. stny. 1/A, Hungary
[2]MTA-ELTE Statistical and Biological Physics Research Group, H-1117 Budapest, Pázmány P. stny. 1/A, Hungary
[3]Health Services Management Training Centre, Semmelweis University, H-1125 Budapest, Kútvölgyi út 2, Hungary.
[*]pallag@hal.elte.hu

We compared the community structures found by the asynchronous label propagation[1,2], the Louvain[3,4] and the Infomap[5,6] algorithms in the PSO[7], E-PSO[8,9] and $\mathbb{S}^1/\mathbb{H}^2$[10–12] networks of various parameter combinations. Each community detection algorithm was executed once for each network. The isolated nodes emerging in the case of the $\mathbb{S}^1/\mathbb{H}^2$ model and occasionally also in the networks generated by the E-PSO model of $L < 0$ were removed before the community detection, meaning that the actual size of the examined networks does not necessarily reach the number of nodes N inputted in these models. We generated 100 networks with each parameter setting and calculated the adjusted mutual information (AMI)[13–15] of the resulted 3 partitions for each network. Figs. D1–D3 display the average and the standard deviation of the AMI between the community structures obtained with asynchronous label propagation and Louvain, Figs. D4–D6 compare the result of asynchronous label propagation with the result of Infomap, while the consistency between the community structures detected by Louvain and Infomap is examined in Figs. D7–D9.

According to these figures, asynchronous label propagation and Infomap produce the most similar partitions, while the most different is the result of Louvain and Infomap. In most of the cases, the AMI depends similarly on the network generation parameters as the weighted modularity Q. For the high modularity regions of the parameter space (large number of nodes N, small average degree $\langle k\rangle$, small popularity fading parameter β or large degree decay exponent γ and low temperature T or large α) the AMI is relatively large for each pair of community detection methods, indicating in these parameter regions the emergence of really apparent communities that are detectable for all the 3 investigated algorithms alike.

In addition, we also examined the stability of the communities in the PSO model when the node coordinates are kept fixed, while the links between the nodes are re-generated from scratch. According to Fig. D10, the repetition of the link generation process does not alter strongly the modules we find. Namely, the adjusted mutual information of the partitions found by a given community detection algorithm on the different realisations of the edge list is typically only slightly lower than the AMI of the community structures found by the algorithm on the exact same network in different runs.

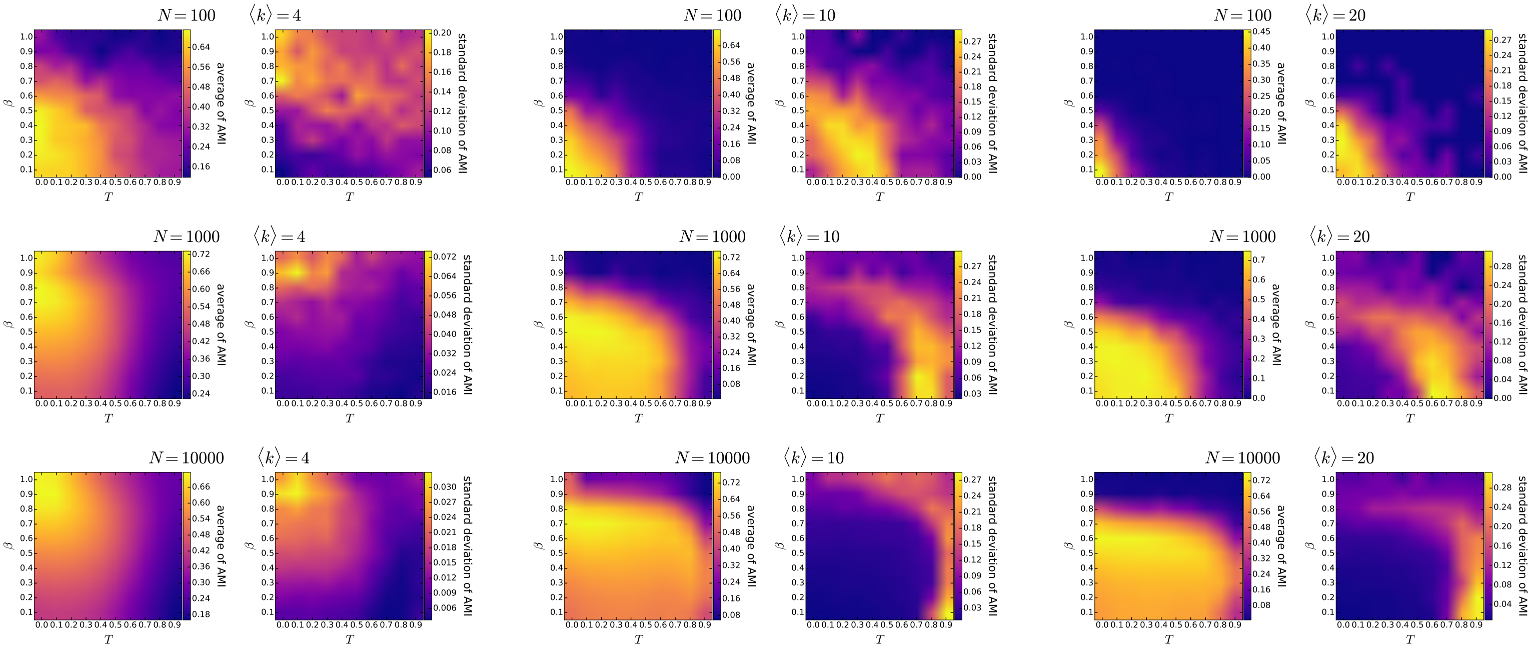

Figure D1. The mean and the standard deviation of the adjusted mutual information of the two community structures detected by the *asynchronous label propagation* and the *Louvain* algorithms in 100 *PSO* networks of different parametrisations. Each pair of subplots depicts the effect of changing the popularity fading parameter β and the temperature T, with the number of nodes N and the expected average degree $\langle k \rangle = 2m$ given in the title of the subplot pair. The curvature K of the hyperbolic plane was always set to -1, i.e. we used $\zeta = 1$.

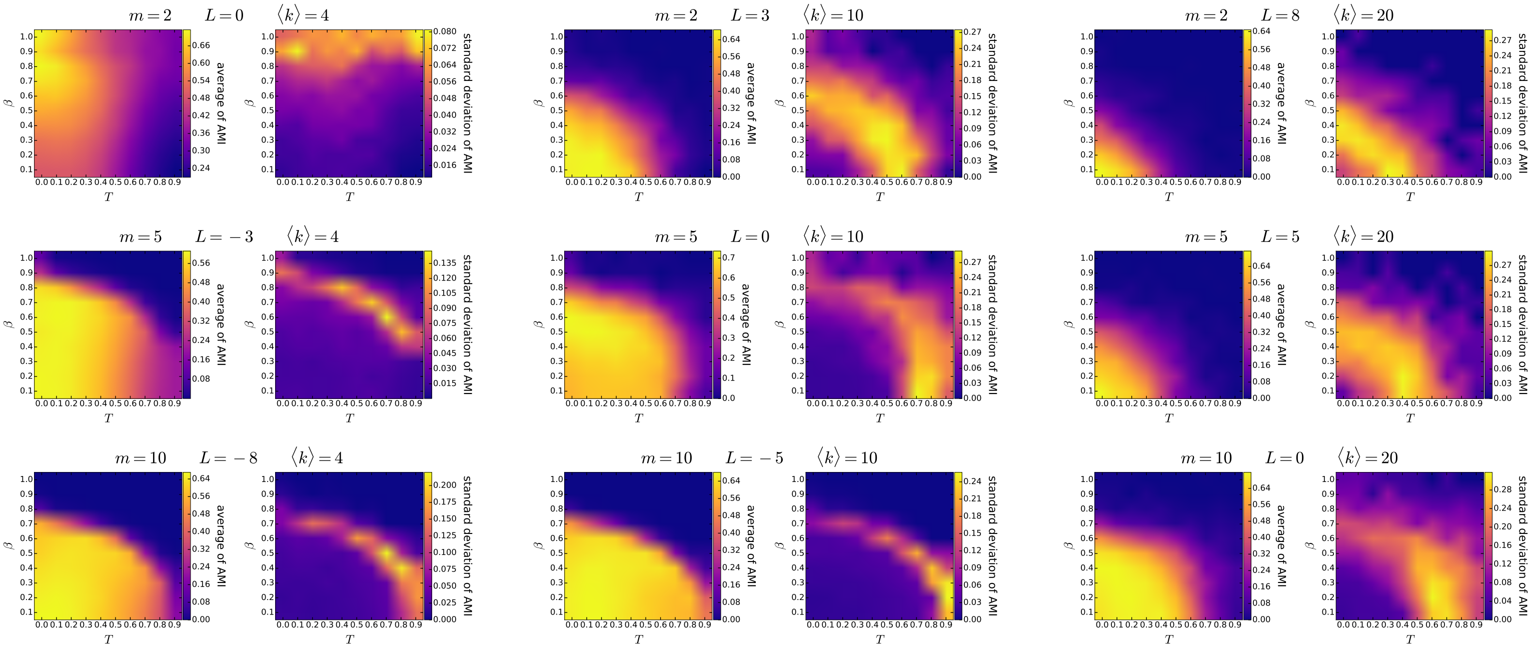

Figure D2. The mean and the standard deviation of the adjusted mutual information of the two community structures detected by the *asynchronous label propagation* and the *Louvain* algorithms in 100 *E-PSO* networks of different parametrisations. Each pair of subplots depicts the effect of changing the popularity fading parameter β and the temperature T, with the parameters m and L given in the title of the subplot pair together with the corresponding expected average degree $\langle k \rangle = 2(m+L)$. The number of nodes N was 1000 in each case. The curvature K of the hyperbolic plane was always set to -1, i.e. we used $\zeta = 1$.

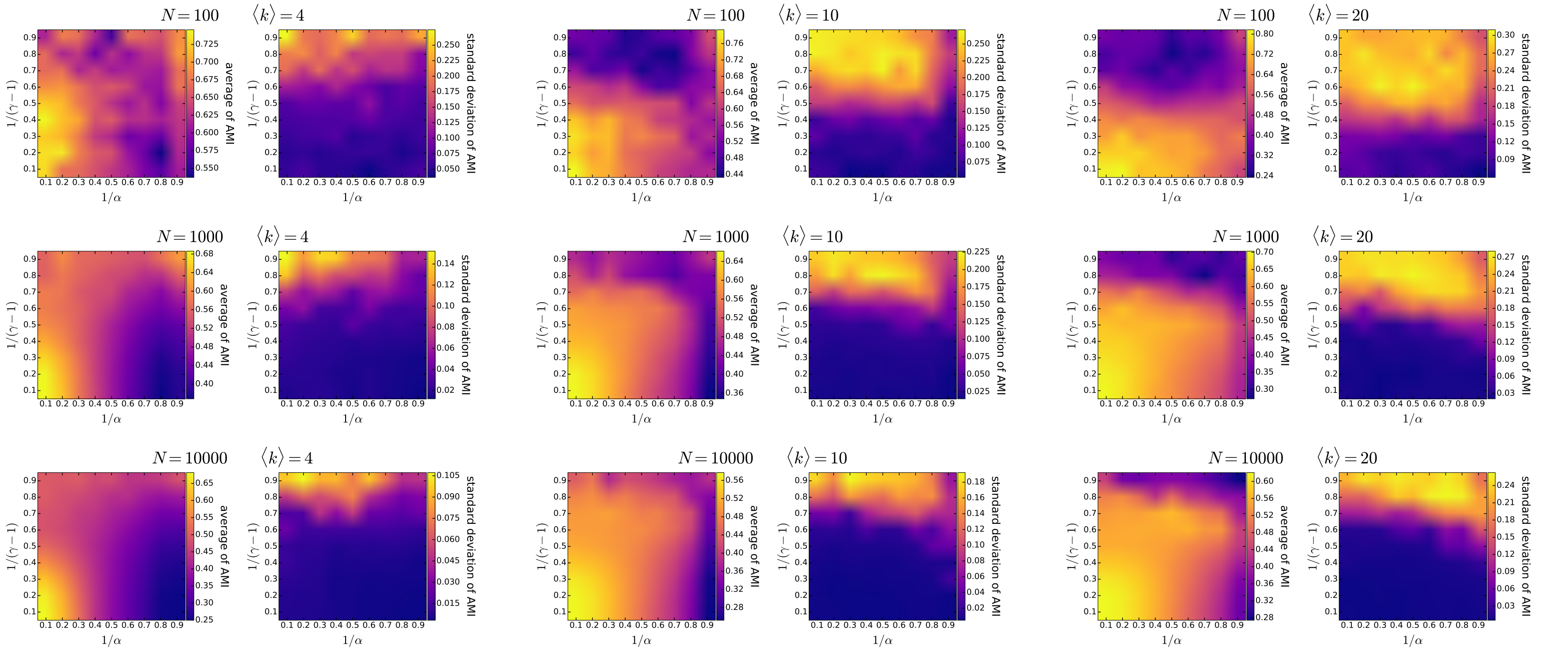

Figure D3. The mean and the standard deviation of the adjusted mutual information of the two community structures detected by the *asynchronous label propagation* and the *Louvain* algorithms in 100 $\mathbb{S}^1/\mathbb{H}^2$ networks of different parametrisations. Each pair of subplots depicts the effect of changing $1/(\gamma - 1)$ (equivalent to the popularity fading parameter β in the E-PSO model) and $1/\alpha$ (analogous to the temperature T in the E-PSO model), with the number of nodes N and the expected average degree $\langle k \rangle$ given in the title of the subplot pair. We used $K = -1$ as the curvature of the hyperbolic plane in each case.

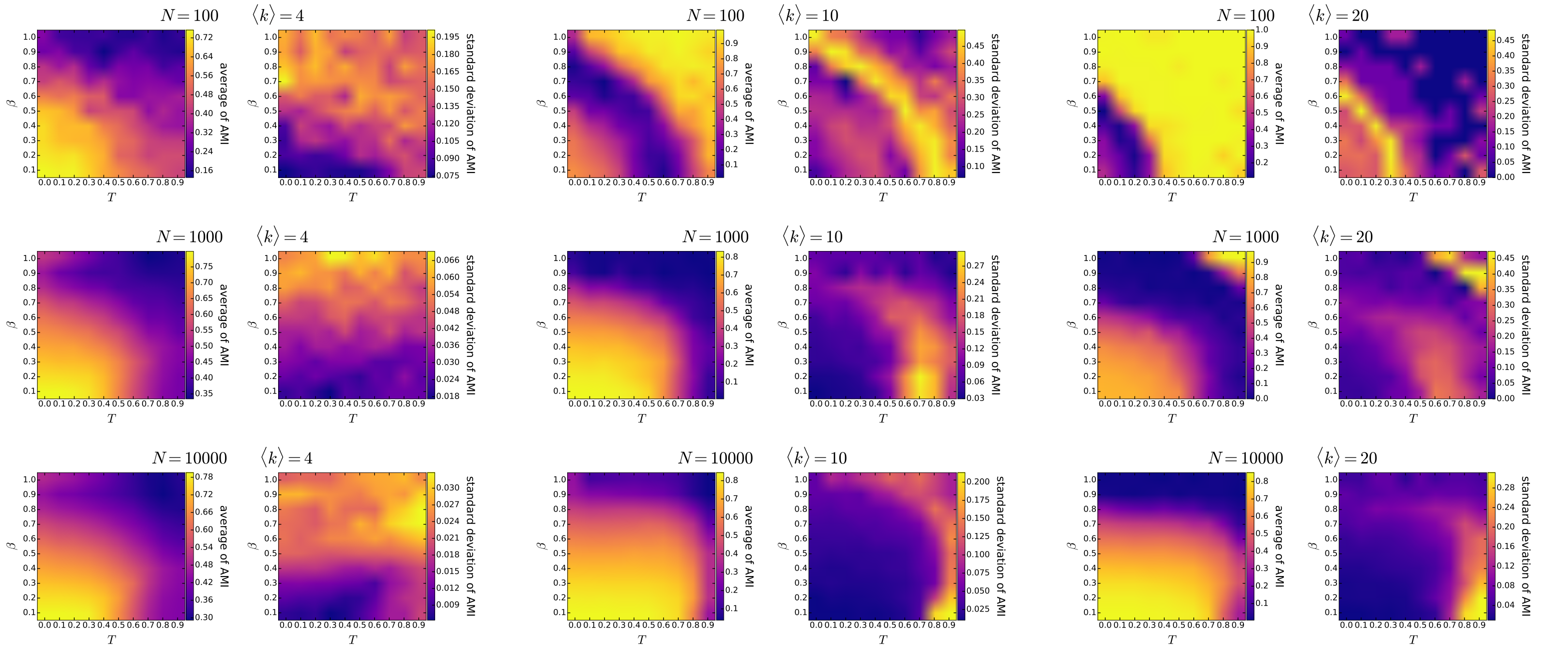

Figure D4. The mean and the standard deviation of the adjusted mutual information of the two community structures detected by the *asynchronous label propagation* and the *Infomap* algorithms in 100 *PSO* networks of different parametrisations. Each pair of subplots depicts the effect of changing the popularity fading parameter β and the temperature T, with the number of nodes N and the expected average degree $\langle k \rangle = 2m$ given in the title of the subplot pair. The curvature K of the hyperbolic plane was always set to -1, i.e. we used $\zeta = 1$.

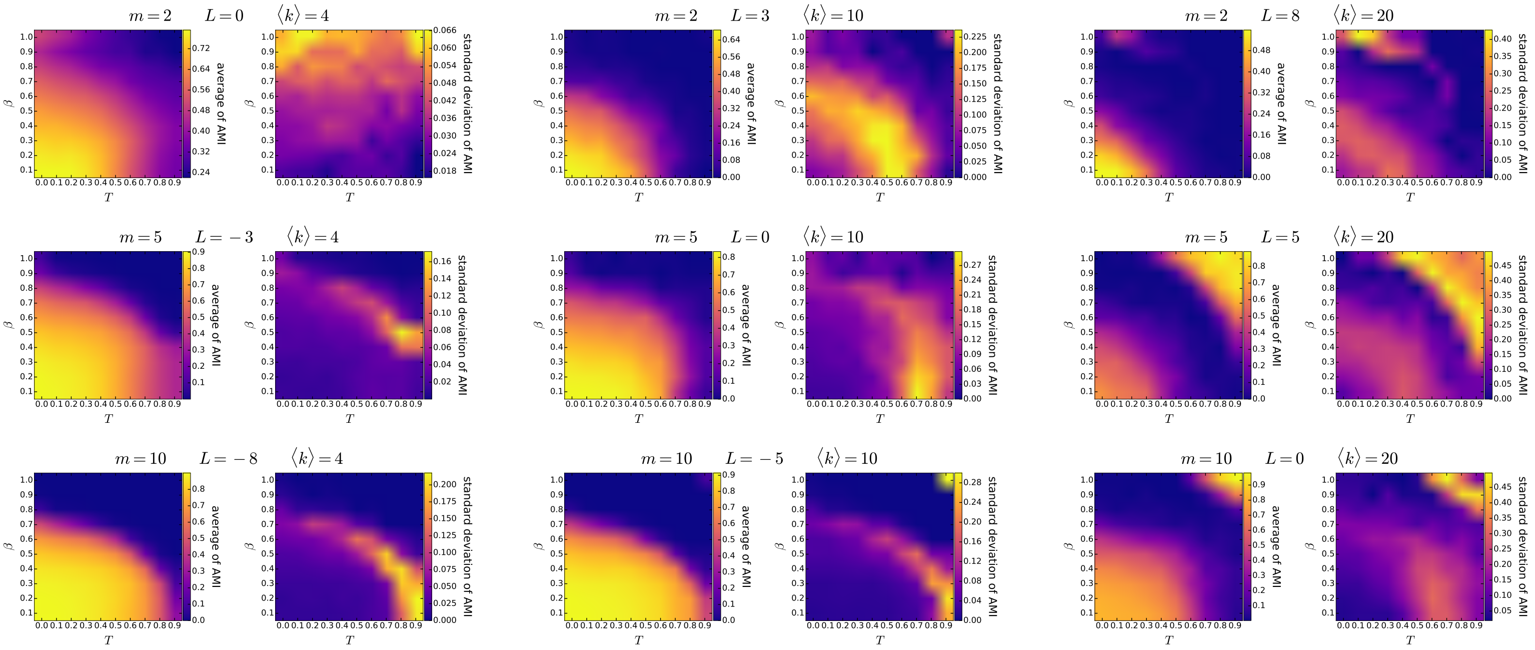

Figure D5. The mean and the standard deviation of the adjusted mutual information of the two community structures detected by the *asynchronous label propagation* and the *Infomap* algorithms in 100 *E-PSO* networks of different parametrisations. Each pair of subplots depicts the effect of changing the popularity fading parameter β and the temperature T, with the parameters m and L given in the title of the subplot pair together with the corresponding expected average degree $\langle k \rangle = 2(m+L)$. The number of nodes N was 1000 in each case. The curvature K of the hyperbolic plane was always set to -1, i.e. we used $\zeta = 1$.

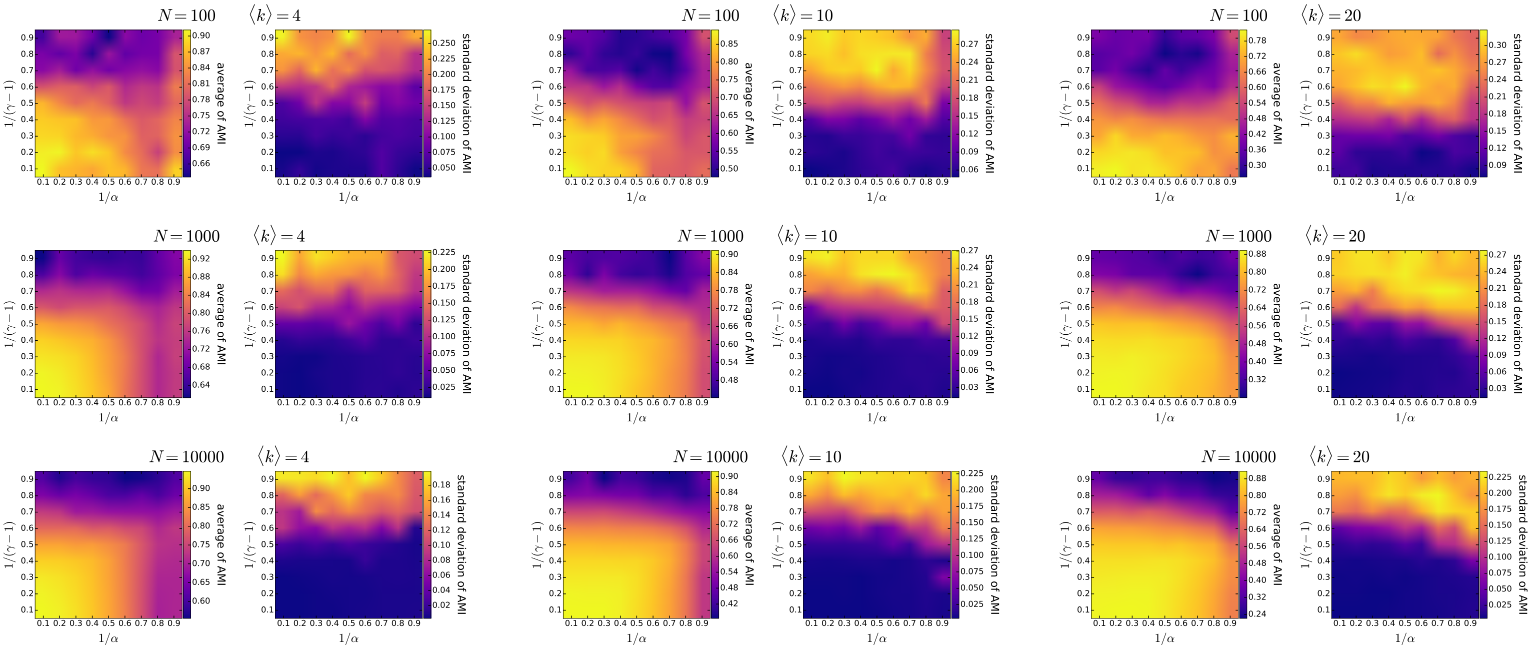

Figure D6. The mean and the standard deviation of the adjusted mutual information of the two community structures detected by the *asynchronous label propagation* and the *Infomap* algorithms in 100 $\mathbb{S}^1/\mathbb{H}^2$ networks of different parametrisations. Each pair of subplots depicts the effect of changing $1/(\gamma-1)$ (equivalent to the popularity fading parameter β in the E-PSO model) and $1/\alpha$ (analogous to the temperature T in the E-PSO model), with the number of nodes N and the expected average degree $\langle k \rangle$ given in the title of the subplot pair. We used $K=-1$ as the curvature of the hyperbolic plane in each case.

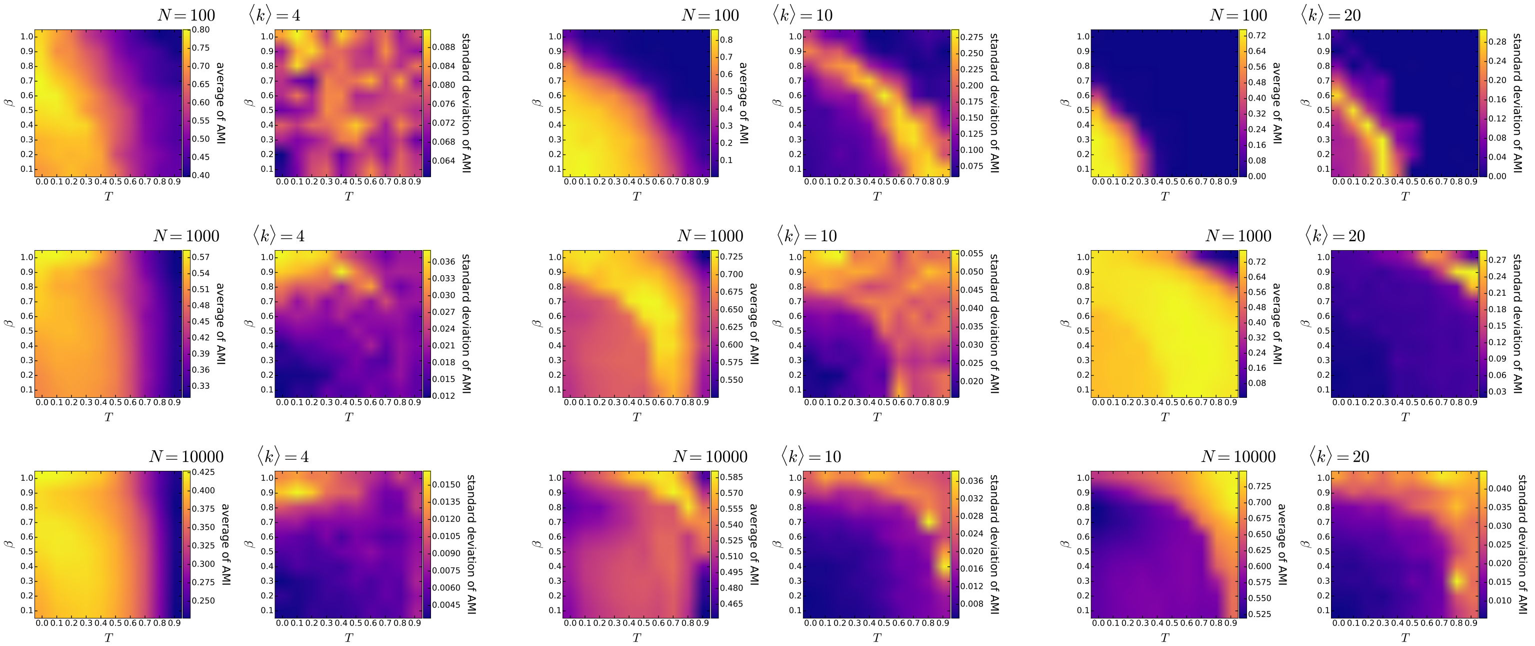

Figure D7. The mean and the standard deviation of the adjusted mutual information of the two community structures detected by the *Louvain* and the *Infomap* algorithms in 100 *PSO* networks of different parametrisations. Each pair of subplots depicts the effect of changing the popularity fading parameter β and the temperature T, with the number of nodes N and the expected average degree $\langle k\rangle = 2m$ given in the title of the subplot pair. The curvature K of the hyperbolic plane was always set to -1, i.e. we used $\zeta = 1$.

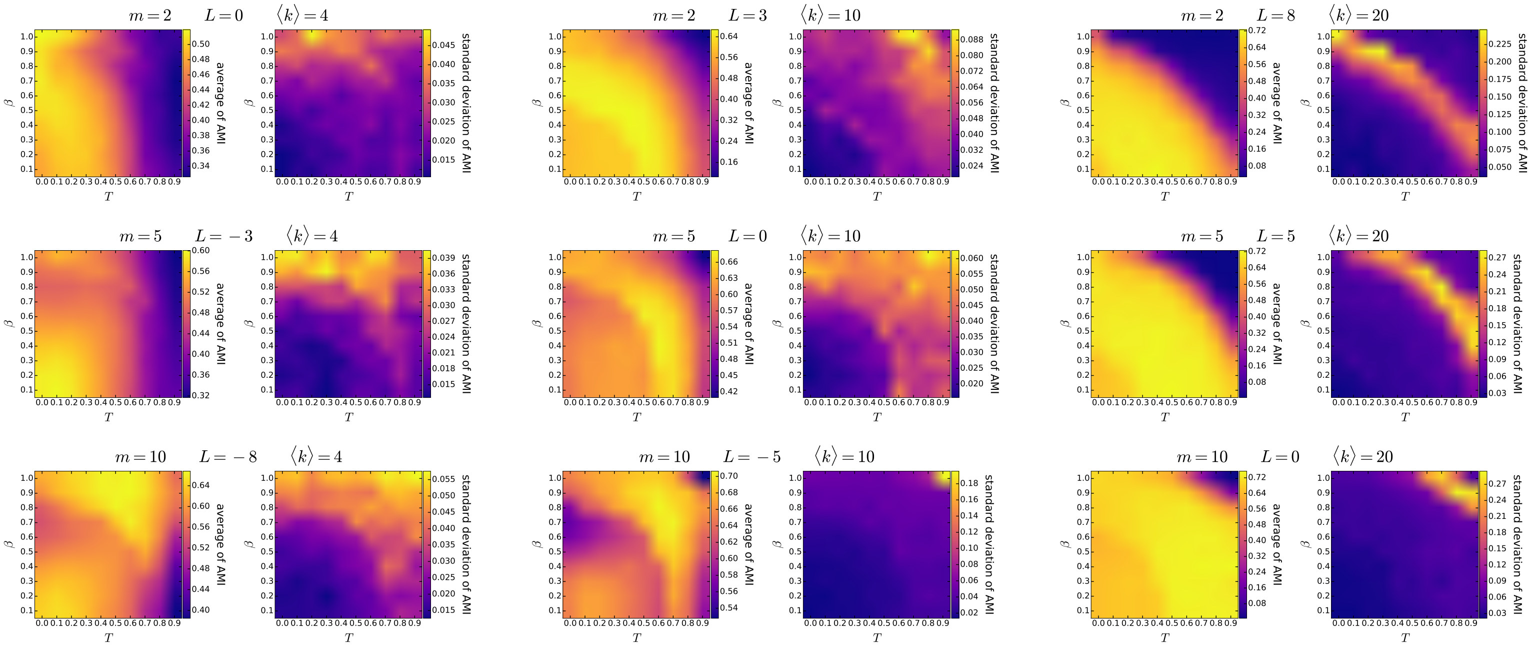

Figure D8. The mean and the standard deviation of the adjusted mutual information of the two community structures detected by the *Louvain* and the *Infomap* algorithms in 100 *E-PSO* networks of different parametrisations. Each pair of subplots depicts the effect of changing the popularity fading parameter β and the temperature T, with the parameters m and L given in the title of the subplot pair together with the corresponding expected average degree $\langle k\rangle = 2(m+L)$. The number of nodes N was 1000 in each case. The curvature K of the hyperbolic plane was always set to -1, i.e. we used $\zeta = 1$.

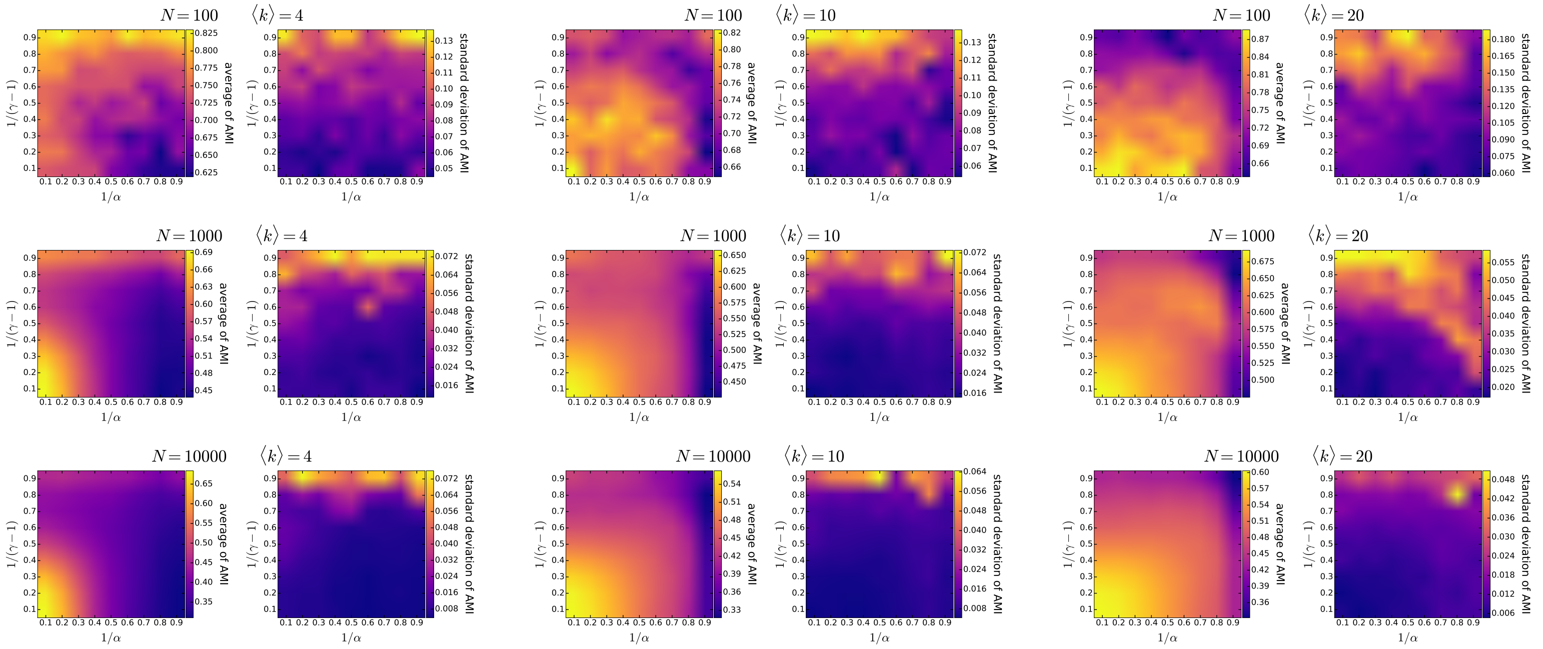

Figure D9. The mean and the standard deviation of the adjusted mutual information of the two community structures detected by the *Louvain* and the *Infomap* algorithms in 100 $\mathbb{S}^1/\mathbb{H}^2$ networks of different parametrisations. Each pair of subplots depicts the effect of changing $1/(\gamma - 1)$ (equivalent to the popularity fading parameter β in the E-PSO model) and $1/\alpha$ (analogous to the temperature T in the E-PSO model), with the number of nodes N and the expected average degree $\langle k \rangle$ given in the title of the subplot pair. We used $K = -1$ as the curvature of the hyperbolic plane in each case.

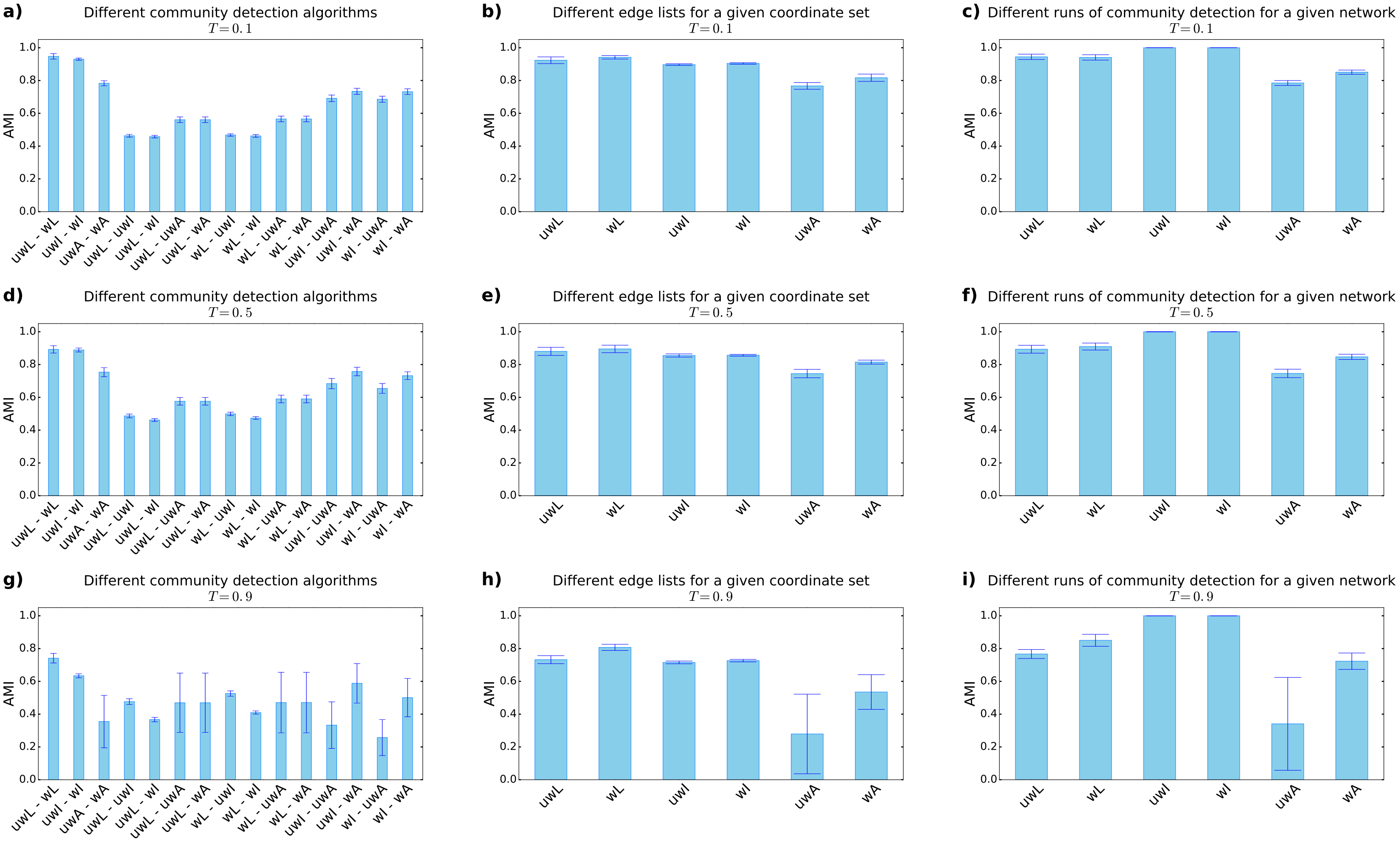

Figure D10. The adjusted mutual information of the community structures that were detected by different algorithms on the same PSO networks, by the same algorithm on different edge lists emerged in the PSO model from the same node coordinates, and in different runs of the same algorithm on the same PSO network. In the left column (panels (a), (d) and (g)) we averaged over 45 networks the AMI of the community structures found by the Louvain (L), the Infomap (I) and the asynchronous label propagation (A) algorithms in one run per network, using the hyperbolic distances for link weighting according to equation (9) of the main text or setting all the link weights to 1 (denoted by w or uw, respectively). For the middle column (panels (b), (e) and (h)) we generated one set of radial and angular node coordinates and repeated the link generation process using these coordinates 10 times. Then, we searched for communities on the obtained 10 networks with the 6 community detection methods that were examined in the left column of the figure, and averaged the adjusted mutual information over the possible 45 pairings of partitions found by the same algorithm on the 10 different edge lists. In the right column (panels (c), (f) and (i)) we show the AMI averaged over the possible 45 pairings of community structures found by each of the 6 examined methods in 10 runs on a single network. The error bars indicate the standard deviations in all panels. All the networks were generated setting the curvature K of the hyperbolic plane to -1, the number of nodes N to 10000, the expected average degree $2m$ to 10 and the popularity fading parameter β to $2/3$ (corresponding to the degree decay exponent $\gamma = 2.5$). The value of the temperature T, given in the title of each panel, increases from top to bottom in each column. Note that in the case of $T = 0$, the edge list is clearly determined for a given set of node coordinates (i.e., hyperbolic distances), and as the temperature becomes higher, more variance arises in the link creation even when using the same node coordinates.

References

1. Raghavan, U. N., Albert, R. & Kumara, S. Near linear time algorithm to detect community structures in large-scale networks. *Phys. Rev. E* **76**, 036106, DOI: 10.1103/PhysRevE.76.036106 (2007).
2. We used the python function 'asyn_lpa_communities', an implementation of the asynchronous label propagation algorithm available in the 'networkx.algorithms.community.label_propagation' package.
3. Blondel, V. D., Guillaume, J.-L., Lambiotte, R. & Lefebvre, E. Fast unfolding of communities in large networks. *J. Stat. Mech. Theory Exp.* **2008**, P10008, DOI: 10.1088/1742-5468/2008/10/p10008 (2008).
4. We used the python implementation of the louvain algorithm available at https://github.com/taynaud/python-louvain. (Accessed: 14/07/2020).
5. Rosvall, M. & Bergstrom, C. T. Multilevel compression of random walks on networks reveals hierarchical organization in large integrated systems. *PLOS ONE* **6**, 1–10, DOI: 10.1371/journal.pone.0018209 (2011).
6. We used the python package for the infomap algorithm available at https://pypi.org/project/infomap/. (Accessed: 14/07/2020).
7. Papadopoulos, F., Kitsak, M., Serrano, M. Á., Boguñá, M. & Krioukov, D. Popularity versus similarity in growing networks. *Nature* **489**, 537 EP –, DOI: 10.1038/nature11459 (2012).
8. Papadopoulos, F., Psomas, C. & Krioukov, D. Network mapping by replaying hyperbolic growth. *IEEE/ACM Transactions on Netw.* **23**, 198–211, DOI: 10.1109/TNET.2013.2294052 (2015).
9. Kovács, B. & Palla, G. Optimisation of the coalescent hyperbolic embedding of complex networks. *Sci. Reports* **11**, 8350, DOI: 10.1038/s41598-021-87333-5 (2021).
10. Serrano, M. A., Krioukov, D. & Boguñá, M. Self-similarity of complex networks and hidden metric spaces. *Phys. Rev. Lett.* **100**, 078701, DOI: 10.1103/PhysRevLett.100.078701 (2008).
11. García-Pérez, G., Allard, A., Serrano, M. Á. & Boguñá, M. Mercator: uncovering faithful hyperbolic embeddings of complex networks. *New J. Phys.* **21**, 123033, DOI: 10.1088/1367-2630/ab57d2 (2019).
12. We used the c++ implementation of the $\mathbb{S}^1/\mathbb{H}^2$ model available at https://github.com/networkgeometry/mercator. (Accessed: 14/07/2020).
13. Vinh, N. X., Epps, J. & Bailey, J. Information theoretic measures for clusterings comparison: Variants, properties, normalization and correction for chance. *J. Mach. Learn. Res.* **11**, 2837–2854 (2010).
14. McCarthy, A. D. & Matula, D. W. Normalized mutual information exaggerates community detection performance. In *SIAM Workshop on Network Science 2018*, 78–79 (2018).
15. We calculated the adjusted mutual information values with the python function 'adjusted_mutual_info_score' available in the 'sklearn.metrics.cluster' package.
