# Supplementary material for: The inherent community structure of hyperbolic networks
Source: Sci Rep. 2021 Aug 6;11:16050. doi: 10.1038/s41598-021-93921-2 (PMC8346486; doi:10.1038/s41598-021-93921-2)
Supplement: Supplementary file 5 — Supplementary Information 5. [file 41598_2021_93921_MOESM5_ESM.pdf]

The inherent community structure of hyperbolic networks
Supplementary E: Equidistant angular node arrangement

Bianka Kovács[1] **and Gergely Palla**[1,2,3,*]

[1]Dept. of Biological Physics, Eötvös Loránd University, H-1117 Budapest, Pázmány P. stny. 1/A, Hungary
[2]MTA-ELTE Statistical and Biological Physics Research Group, H-1117 Budapest, Pázmány P. stny. 1/A, Hungary
[3]Health Services Management Training Centre, Semmelweis University, H-1125 Budapest, Kútvölgyi út 2, Hungary.
[*]pallag@hal.elte.hu

In order to exclude the possibility that the emergence of communities is a result of the inhomogeneities in the angular node arrangement arising inevitably when a finite number of angular coordinates is sampled from a uniform distribution in $[0,2\pi)$, we modified the PSO model[1] to use strictly equidistant angular arrangement, i.e. instead of sampling the angular coordinates uniformly randomly, assign the coordinates $\theta_i = (i-1)\cdot\frac{2\pi}{N}$, $i = 1,2,...,N$ to the network nodes in a randomly chosen order. According to Figs. E1–E4, the networks generated by this modified PSO model possess a community structure with similarly high weighted modularity as the usual PSO networks (see Figs. B1 and B4–B6). Hence, we can conclude that the emergence of communities is not just an effect of the finite network size, but the inherent property of the studied hyperbolic network models. Figs. E5–E10 show similar results for the networks generated by the modified PSO model with regard to the community size distributions as Figs. C1–C6 in the case of the original PSO model. Lastly, the adjusted mutual information (AMI) between the community structures detected by asynchronous label propagation[2,3], Louvain[4,5] and Infomap[6,7] behaves the same way for the modified PSO model (Figs. E11–E13) as for the original PSO model (Figs. D1, D4 and D7).

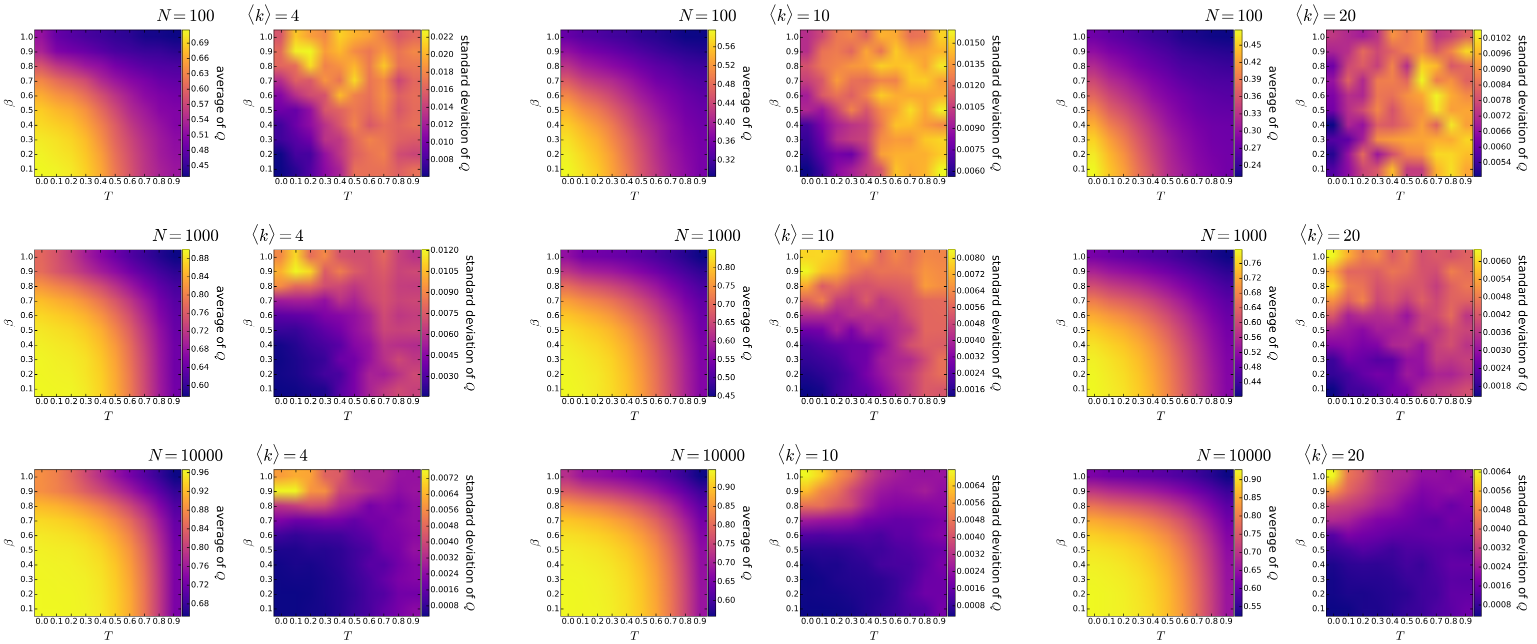

Figure E1. The mean and the standard deviation of the highest weighted modularity Q achieved among the *asynchronous label propagation*, the *Louvain* and the *Infomap* algorithms in 100 *PSO* networks of different parametrisations *with strictly equidistant angular arrangement*. Each pair of subplots depicts the effect of changing the popularity fading parameter β and the temperature T, with the number of nodes N and the expected average degree $\langle k\rangle = 2m$ given in the title of the subplot pair. The curvature of the hyperbolic plane K was always set to -1, i.e. we used $\zeta = 1$.

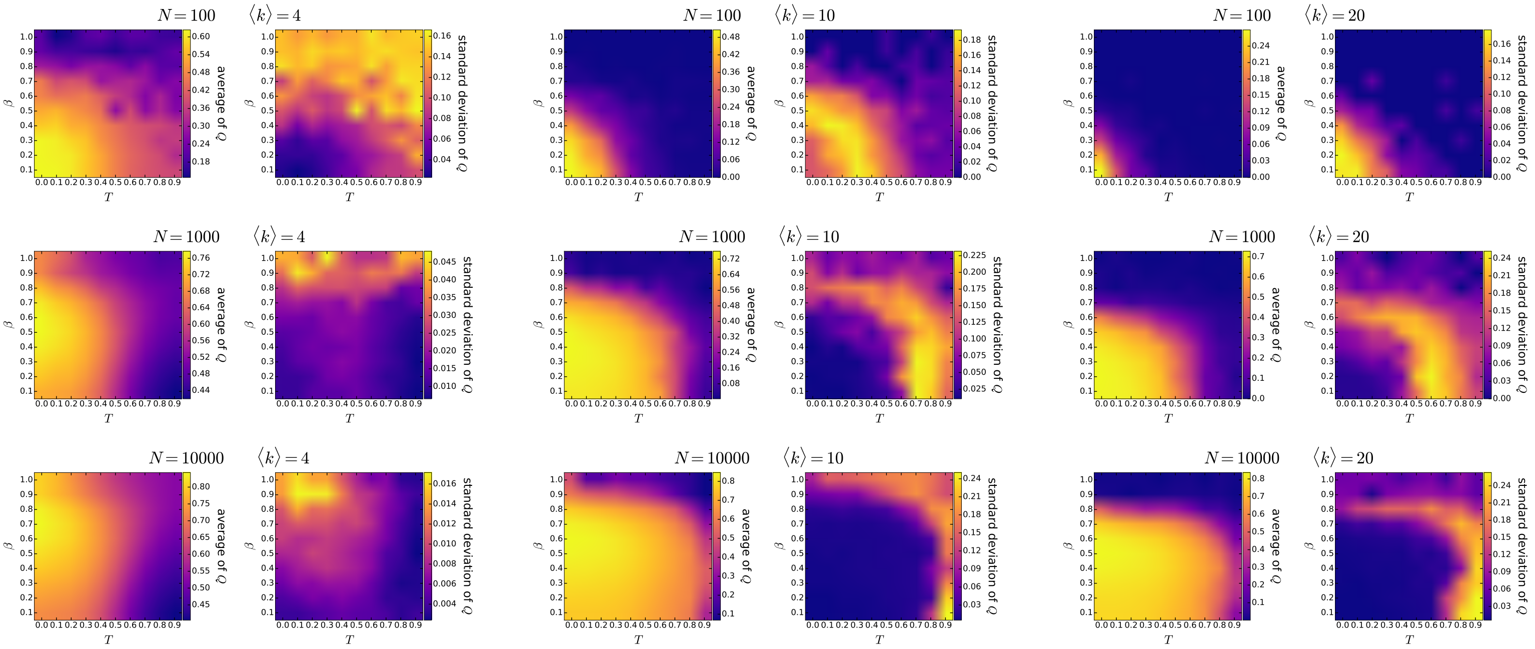

Figure E2. The mean and the standard deviation of the weighted modularity Q of the community structure detected by the *asynchronous label propagation* algorithm in 100 *PSO* networks of different parametrisations *with strictly equidistant angular arrangement*. Each pair of subplots depicts the effect of changing the popularity fading parameter β and the temperature T, with the number of nodes N and the expected average degree $\langle k \rangle = 2m$ given in the title of the subplot pair. The curvature of the hyperbolic plane K was always set to -1, i.e. we used $\zeta = 1$.

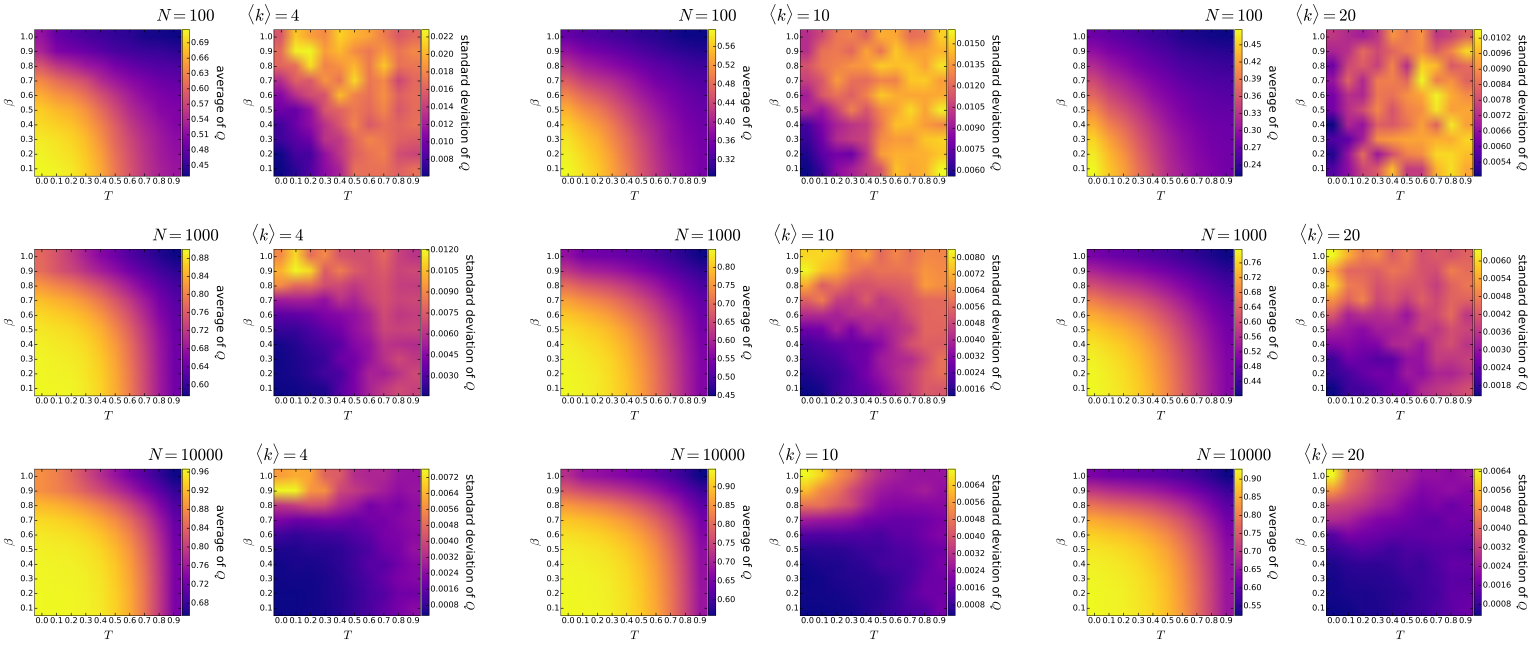

Figure E3. The mean and the standard deviation of the weighted modularity Q of the community structure detected by the *Louvain* algorithm in 100 *PSO* networks of different parametrisations *with strictly equidistant angular arrangement*. Each pair of subplots depicts the effect of changing the popularity fading parameter β and the temperature T, with the number of nodes N and the expected average degree $\langle k \rangle = 2m$ given in the title of the subplot pair. The curvature of the hyperbolic plane K was always set to -1, i.e. we used $\zeta = 1$.

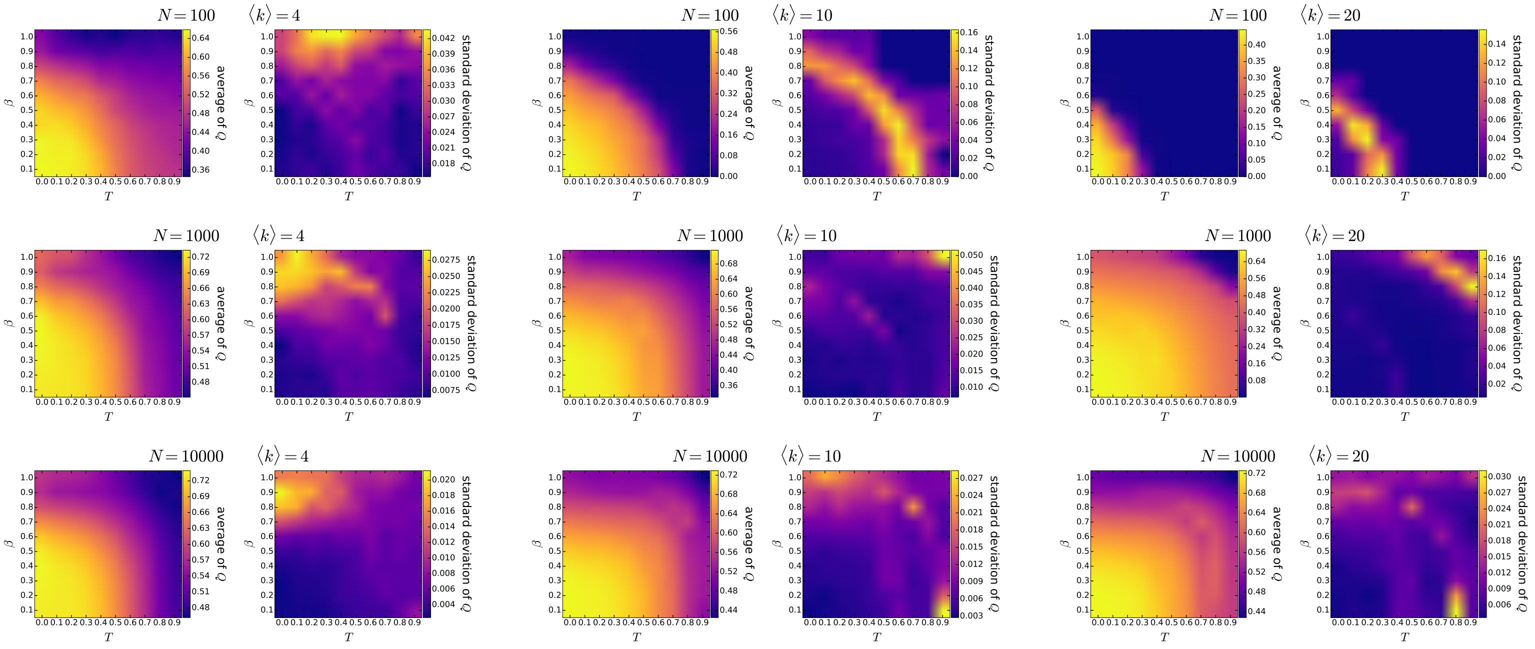

Figure E4. The mean and the standard deviation of the weighted modularity Q of the community structure detected by the *Infomap* algorithm in 100 *PSO* networks of different parametrisations *with strictly equidistant angular arrangement*. Each pair of subplots depicts the effect of changing the popularity fading parameter β and the temperature T, with the number of nodes N and the expected average degree $\langle k \rangle = 2m$ given in the title of the subplot pair. The curvature of the hyperbolic plane K was always set to -1, i.e. we used $\zeta = 1$.

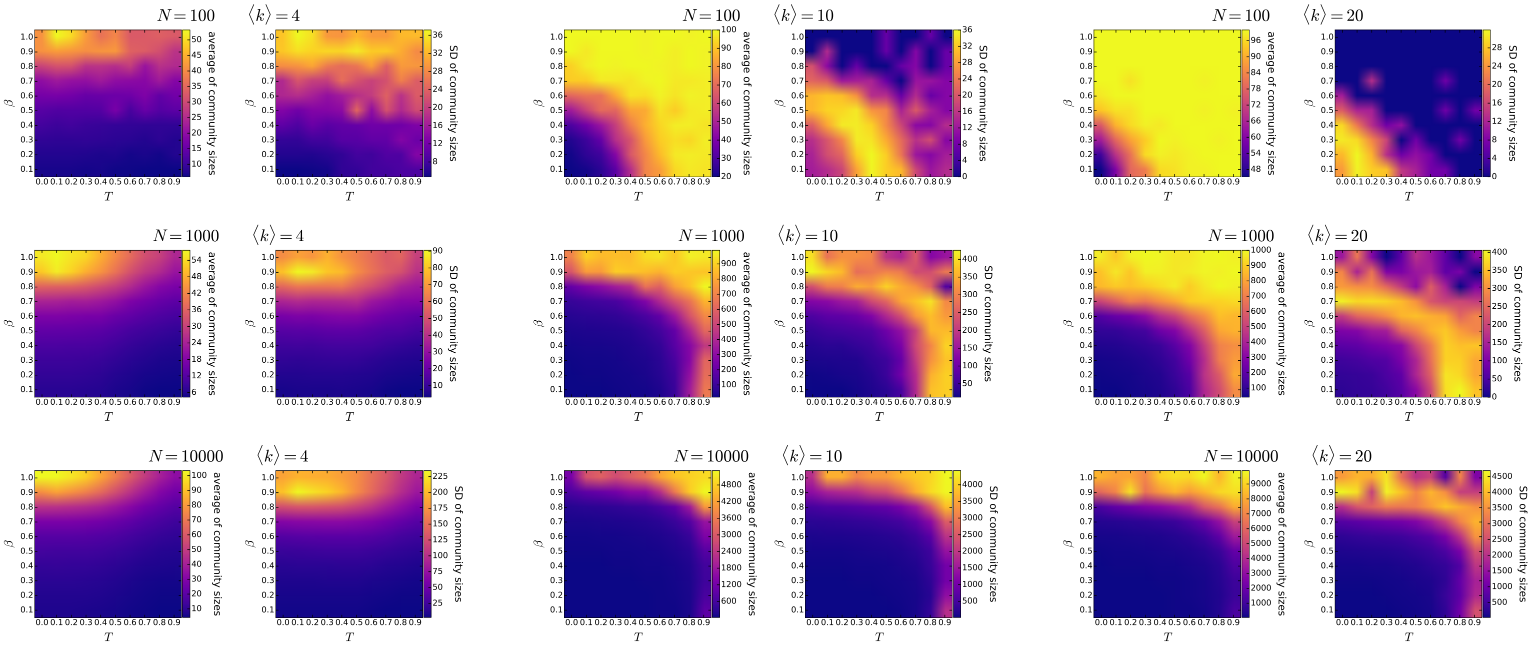

Figure E5. The mean and the standard deviation of the size of communities detected by the *asynchronous label propagation* algorithm in 100 *PSO* networks of different parametrisations *with strictly equidistant angular arrangement*. Each pair of subplots depicts the effect of changing the popularity fading parameter β and the temperature T, with the number of nodes N and the expected average degree $\langle k \rangle = 2m$ given in the title of the subplot pair. The curvature of the hyperbolic plane K was always set to -1, i.e. we used $\zeta = 1$.

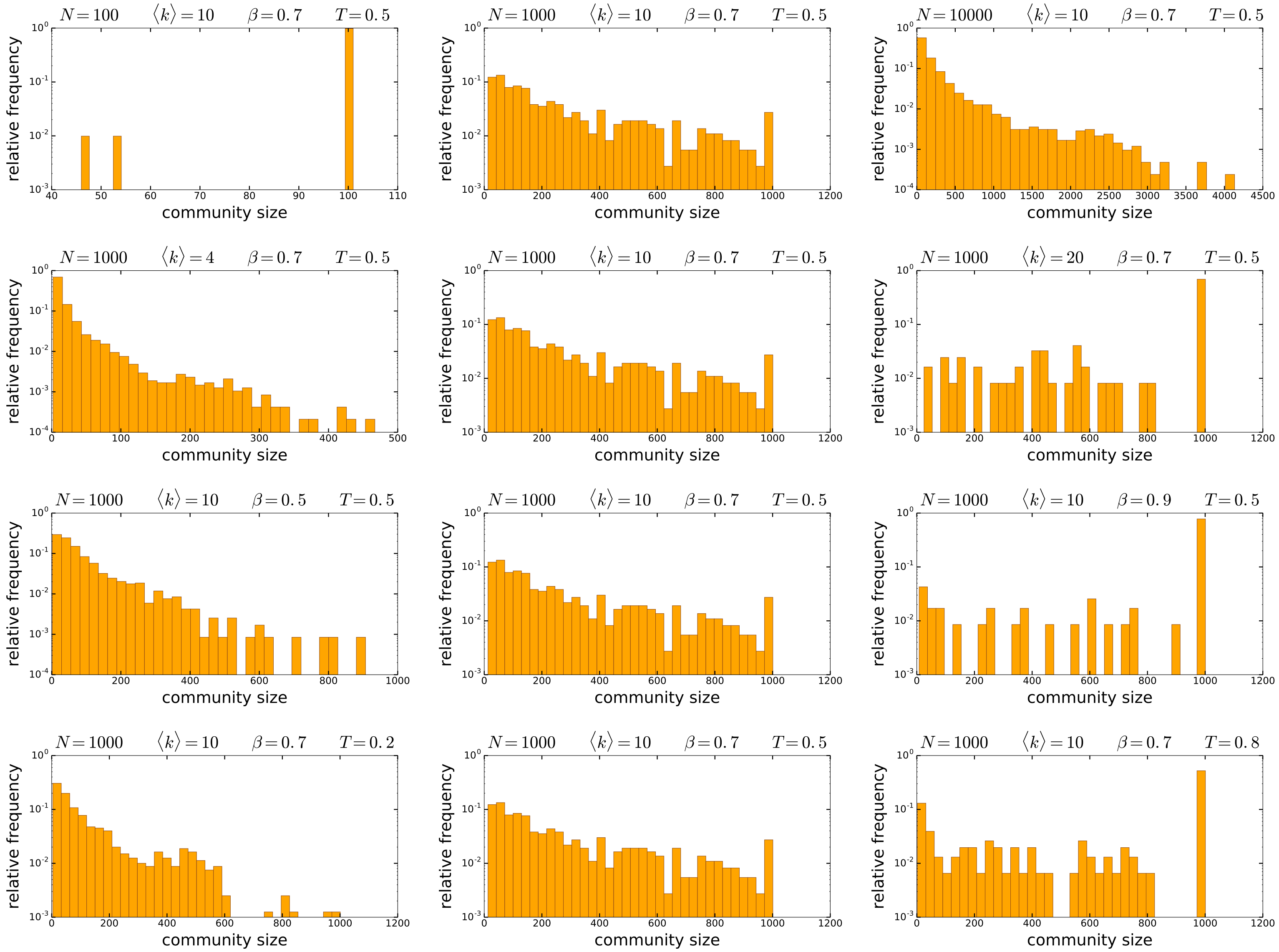

Figure E6. The size distribution of the communities detected by the *asynchronous label propagation* algorithm in 100 *PSO* networks of different parametrisations *with strictly equidistant angular arrangement*. The parameters of the network generation are listed in the title for each subplot. The curvature of the hyperbolic plane K was always set to -1, i.e. we used $\zeta = 1$. Each row of the figure demonstrates the effect of the change in a given network generation parameter: from top to bottom, the number of nodes N, the expected average degree $\langle k \rangle = 2m$, the popularity fading parameter β and the temperature T.

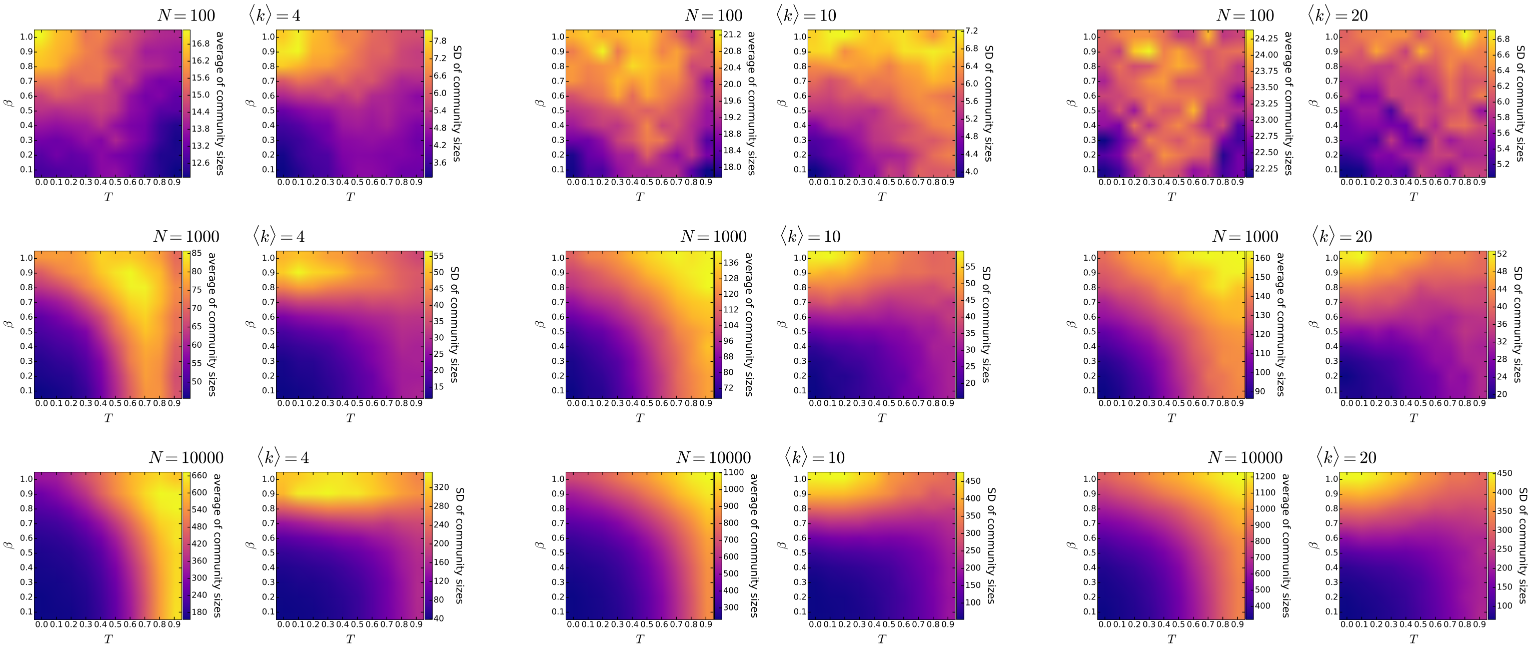

Figure E7. The mean and the standard deviation of the size of communities detected by the *Louvain* algorithm in 100 *PSO* networks of different parametrisations *with strictly equidistant angular arrangement*. Each pair of subplots depicts the effect of changing the popularity fading parameter β and the temperature T, with the number of nodes N and the expected average degree $\langle k \rangle = 2m$ given in the title of the subplot pair. The curvature of the hyperbolic plane K was always set to -1, i.e. we used $\zeta = 1$.

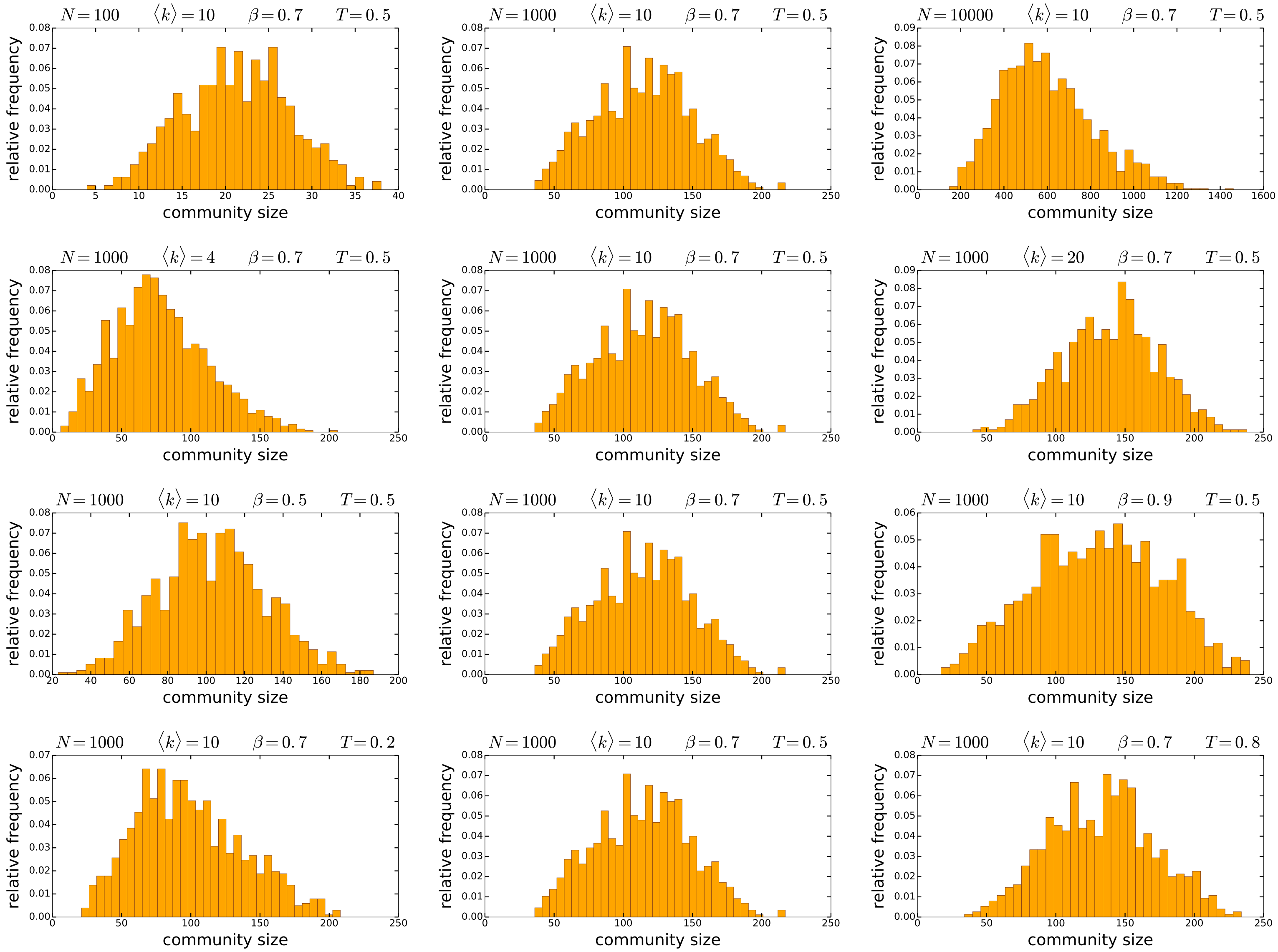

Figure E8. The size distribution of the communities detected by the *Louvain* algorithm in 100 *PSO* networks of different parametrisations *with strictly equidistant angular arrangement*. The parameters of the network generation are listed in the title for each subplot. The curvature of the hyperbolic plane K was always set to -1, i.e. we used $\zeta = 1$. Each row of the figure demonstrates the effect of the change in a given network generation parameter: from top to bottom, the number of nodes N, the expected average degree $\langle k \rangle = 2m$, the popularity fading parameter β and the temperature T.

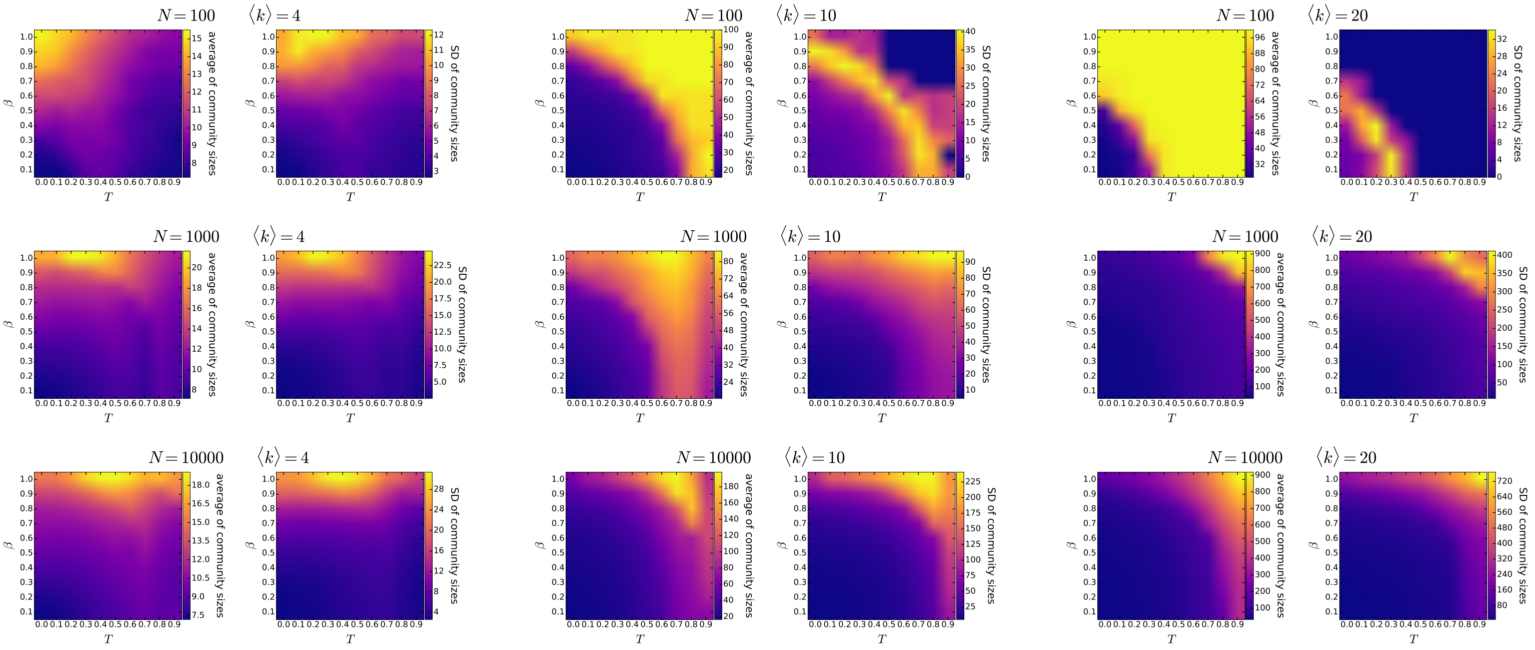

Figure E9. The mean and the standard deviation of the size of communities detected by the *Infomap* algorithm in 100 *PSO* networks of different parametrisations *with strictly equidistant angular arrangement*. Each pair of subplots depicts the effect of changing the popularity fading parameter β and the temperature T, with the number of nodes N and the expected average degree $\langle k \rangle = 2m$ given in the title of the subplot pair. The curvature of the hyperbolic plane K was always set to -1, i.e. we used $\zeta = 1$.

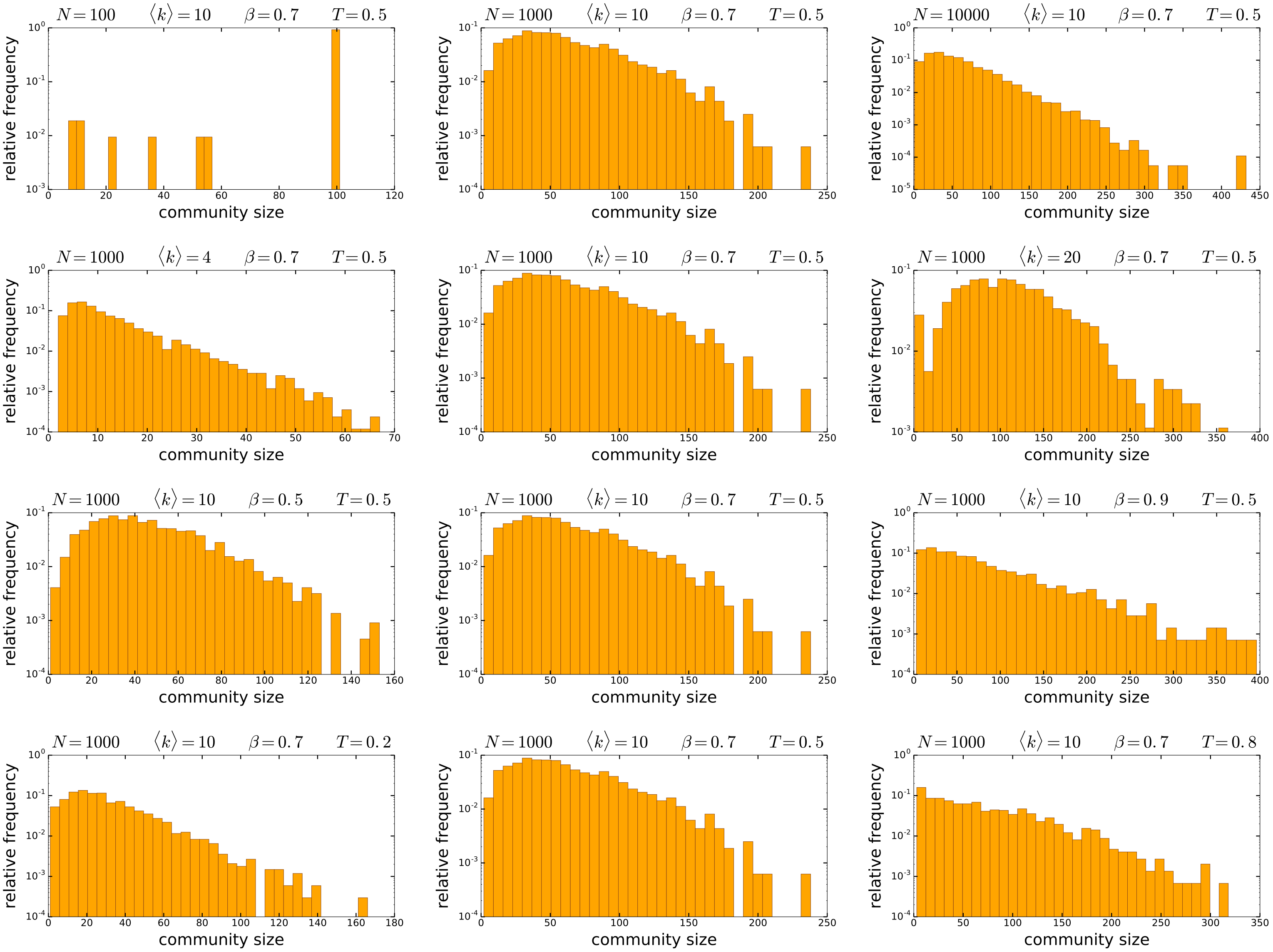

Figure E10. The size distribution of the communities detected by the *Infomap* algorithm in 100 *PSO* networks of different parametrisations *with strictly equidistant angular arrangement*. The parameters of the network generation are listed in the title for each subplot. The curvature of the hyperbolic plane K was always set to -1, i.e. we used $\zeta = 1$. Each row of the figure demonstrates the effect of the change in a given network generation parameter: from top to bottom, the number of nodes N, the expected average degree $\langle k\rangle = 2m$, the popularity fading parameter β and the temperature T.

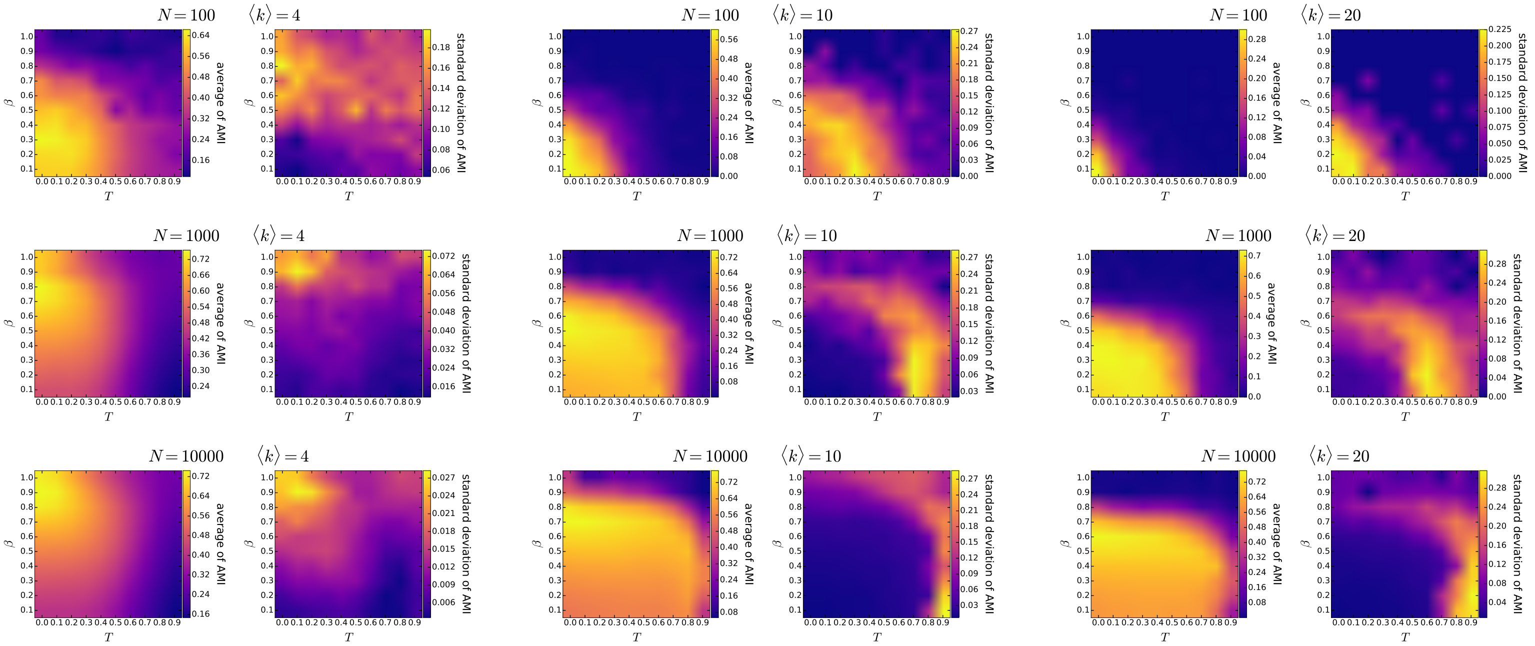

Figure E11. The mean and the standard deviation of the adjusted mutual information of the two community structures detected by the *asynchronous label propagation* and the *Louvain* algorithms in 100 *PSO* networks of different parametrisations *with strictly equidistant angular arrangement*. Each pair of subplots depicts the effect of changing the popularity fading parameter β and the temperature T, with the number of nodes N and the expected average degree $\langle k \rangle = 2m$ given in the title of the subplot pair. The curvature of the hyperbolic plane K was always set to -1, i.e. we used $\zeta = 1$.

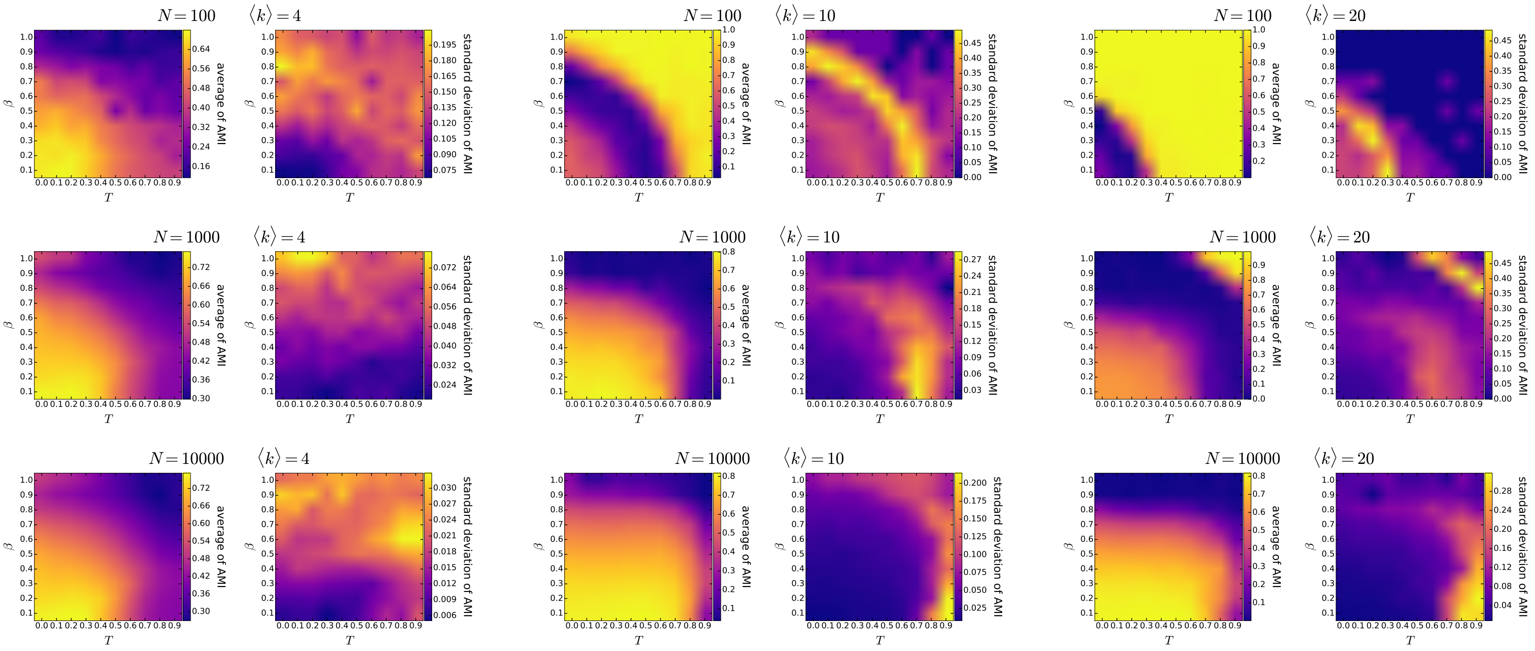

Figure E12. The mean and the standard deviation of the adjusted mutual information of the two community structures detected by the *asynchronous label propagation* and the *Infomap* algorithms in 100 *PSO* networks of different parametrisations *with strictly equidistant angular arrangement*. Each pair of subplots depicts the effect of changing the popularity fading parameter β and the temperature T, with the number of nodes N and the expected average degree $\langle k \rangle = 2m$ given in the title of the subplot pair. The curvature of the hyperbolic plane K was always set to -1, i.e. we used $\zeta = 1$.

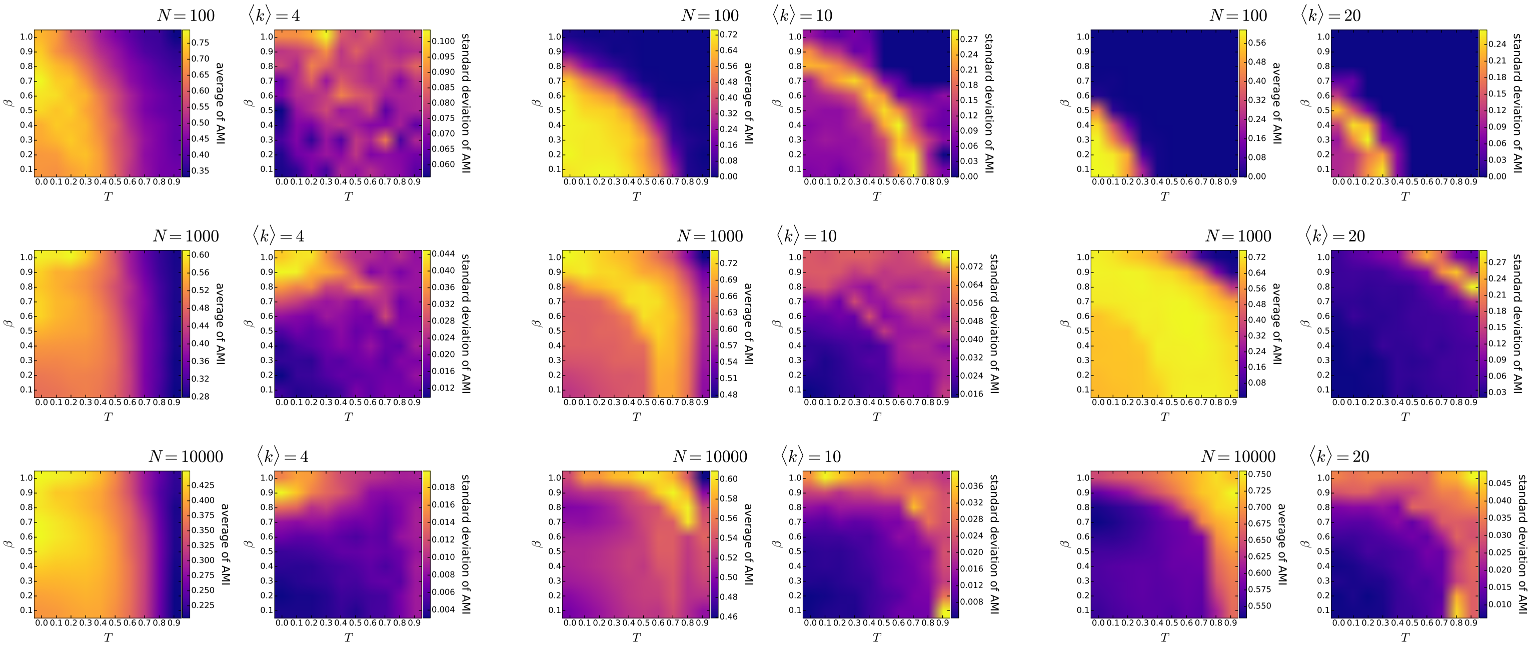

Figure E13. The mean and the standard deviation of the adjusted mutual information of the two community structures detected by the *Louvain* and the *Infomap* algorithms in 100 *PSO* networks of different parametrisations *with strictly equidistant angular arrangement*. Each pair of subplots depicts the effect of changing the popularity fading parameter β and the temperature T, with the number of nodes N and the expected average degree $\langle k \rangle = 2m$ given in the title of the subplot pair. The curvature of the hyperbolic plane K was always set to -1, i.e. we used $\zeta = 1$.

References

1. Papadopoulos, F., Kitsak, M., Serrano, M. Á., Boguñá, M. & Krioukov, D. Popularity versus similarity in growing networks. *Nature* **489**, 537 EP –, DOI: 10.1038/nature11459 (2012).
2. Raghavan, U. N., Albert, R. & Kumara, S. Near linear time algorithm to detect community structures in large-scale networks. *Phys. Rev. E* **76**, 036106, DOI: 10.1103/PhysRevE.76.036106 (2007).
3. We used the python function 'asyn_lpa_communities', an implementation of the asynchronous label propagation algorithm available in the 'networkx.algorithms.community.label_propagation' package.
4. Blondel, V. D., Guillaume, J.-L., Lambiotte, R. & Lefebvre, E. Fast unfolding of communities in large networks. *J. Stat. Mech. Theory Exp.* **2008**, P10008, DOI: 10.1088/1742-5468/2008/10/p10008 (2008).
5. We used the python implementation of the louvain algorithm available at https://github.com/taynaud/python-louvain. (Accessed: 14/07/2020).
6. Rosvall, M. & Bergstrom, C. T. Multilevel compression of random walks on networks reveals hierarchical organization in large integrated systems. *PLOS ONE* **6**, 1–10, DOI: 10.1371/journal.pone.0018209 (2011).
7. We used the python package for the infomap algorithm available at https://pypi.org/project/infomap/. (Accessed: 14/07/2020).
