# Supplementary material for: The inherent community structure of hyperbolic networks
Source: Sci Rep. 2021 Aug 6;11:16050. doi: 10.1038/s41598-021-93921-2 (PMC8346486; doi:10.1038/s41598-021-93921-2)
Supplement: Supplementary file 6 — Supplementary Information 6. [file 41598_2021_93921_MOESM6_ESM.pdf]

The inherent community structure of hyperbolic networks
Supplementary F: Community detection on unweighted hyperbolic networks

Bianka Kovács[1] **and Gergely Palla**[1,2,3,*]

[1]Dept. of Biological Physics, Eötvös Loránd University, H-1117 Budapest, Pázmány P. stny. 1/A, Hungary
[2]MTA-ELTE Statistical and Biological Physics Research Group, H-1117 Budapest, Pázmány P. stny. 1/A, Hungary
[3]Health Services Management Training Centre, Semmelweis University, H-1125 Budapest, Kútvölgyi út 2, Hungary.
[*]pallag@hal.elte.hu

We studied the quality of the community structures detected by the asynchronous label propagation[1,2], the Louvain[3,4] and the Infomap[5,6] algorithms in PSO[7], E-PSO[8,9] and $\mathbb{S}^1/\mathbb{H}^2$[10–12] networks of various parameter combinations. The isolated nodes emerging in the case of the $\mathbb{S}^1/\mathbb{H}^2$ model and occasionally also in the networks generated by the E-PSO model of $L<0$ were removed before the community detection, meaning that the actual size of the examined networks does not necessarily reach the number of nodes N inputted in these models. Each community detection algorithm was executed once for each network. This document presents the results obtained in the case of setting each link weight in the previously examined synthetic networks to 1, independently of the hyperbolic distance between the connected nodes. Similarly to Figs. B4–B12, Figs. F1–F9 show how the unweighted modularity[13,14] achieved by the three different community detection algorithms depends on the network generation parameters. As in Supplementary C, Figs. F10–F27 display how the mean and the standard deviation of the community sizes depend on the network generation parameters, as well as some examples for the corresponding community size distributions. Figs. F28–F36 are analogous to the figures of Supplementary D, showing the similarity between the community structures detected by the different methods in the unweighted networks by means of the adjusted mutual information (AMI)[15–17]. Lastly, Fig. 2 of the main article depicting the angular separation index (ASI) of the detected communities[18] is repeated for the unweighted case in Fig. F37.

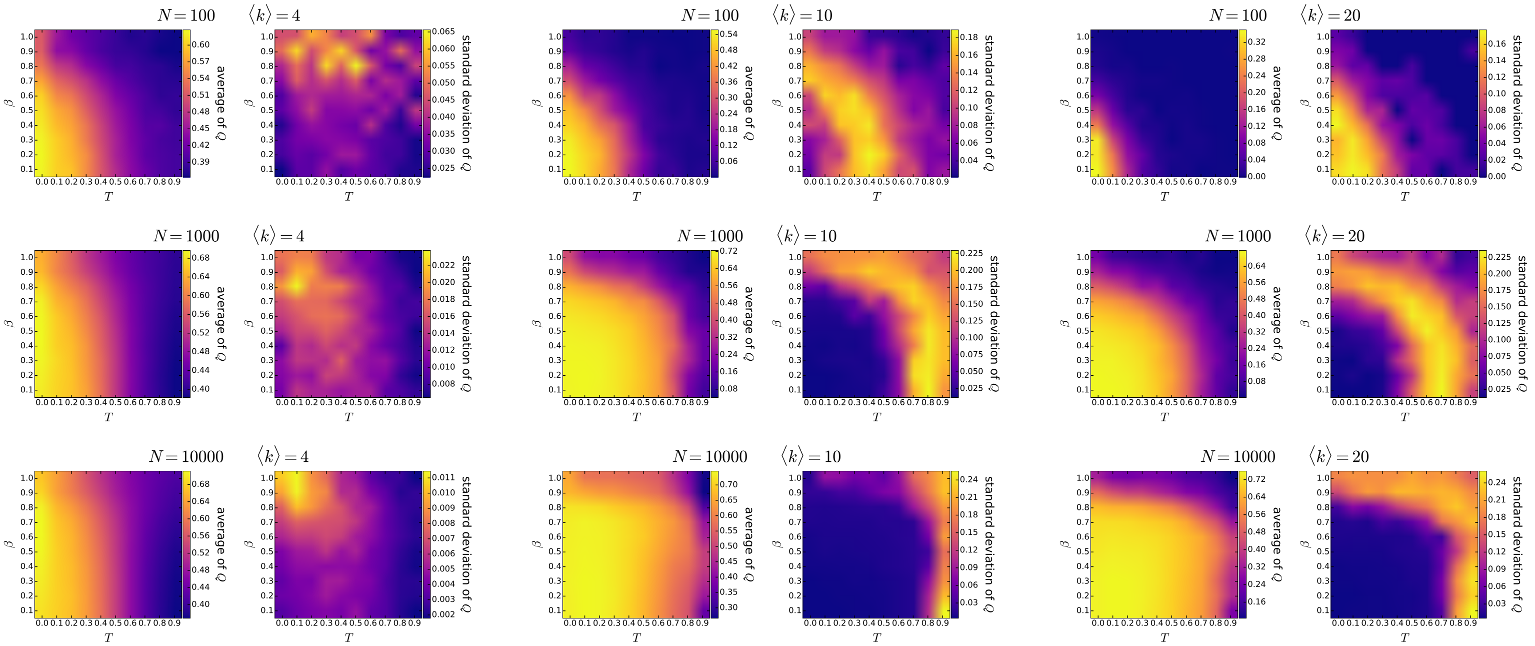

Figure F1. The mean and the standard deviation of the unweighted modularity Q of the community structure detected by the *asynchronous label propagation* algorithm in 100 *unweighted PSO* networks of different parametrisations. Each pair of subplots depicts the effect of changing the popularity fading parameter β and the temperature T, with the number of nodes N and the expected average degree $\langle k \rangle = 2m$ given in the title of the subplot pair. The curvature of the hyperbolic plane K was always set to -1, i.e. we used $\zeta = 1$.

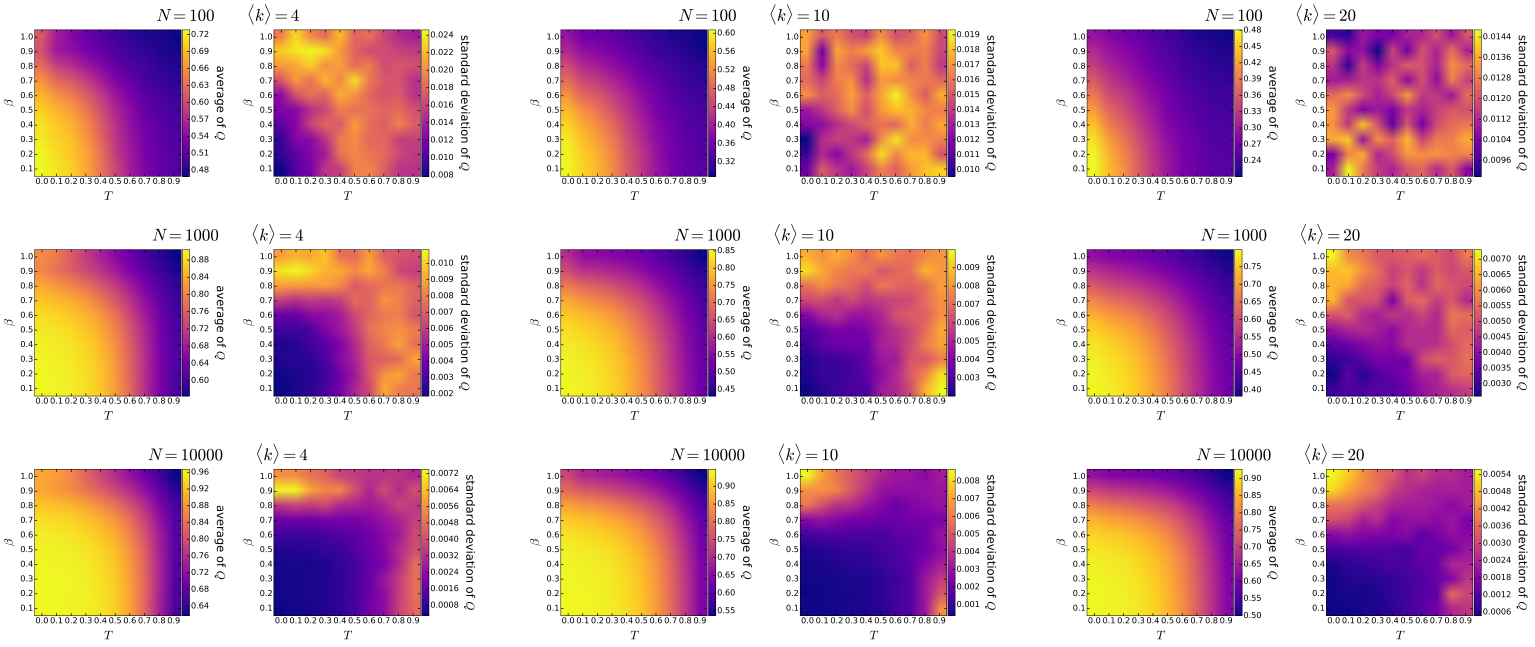

Figure F2. The mean and the standard deviation of the unweighted modularity Q of the community structure detected by the *Louvain* algorithm in 100 *unweighted PSO* networks of different parametrisations. Each pair of subplots depicts the effect of changing the popularity fading parameter β and the temperature T, with the number of nodes N and the expected average degree $\langle k \rangle = 2m$ given in the title of the subplot pair. The curvature of the hyperbolic plane K was always set to -1, i.e. we used $\zeta = 1$.

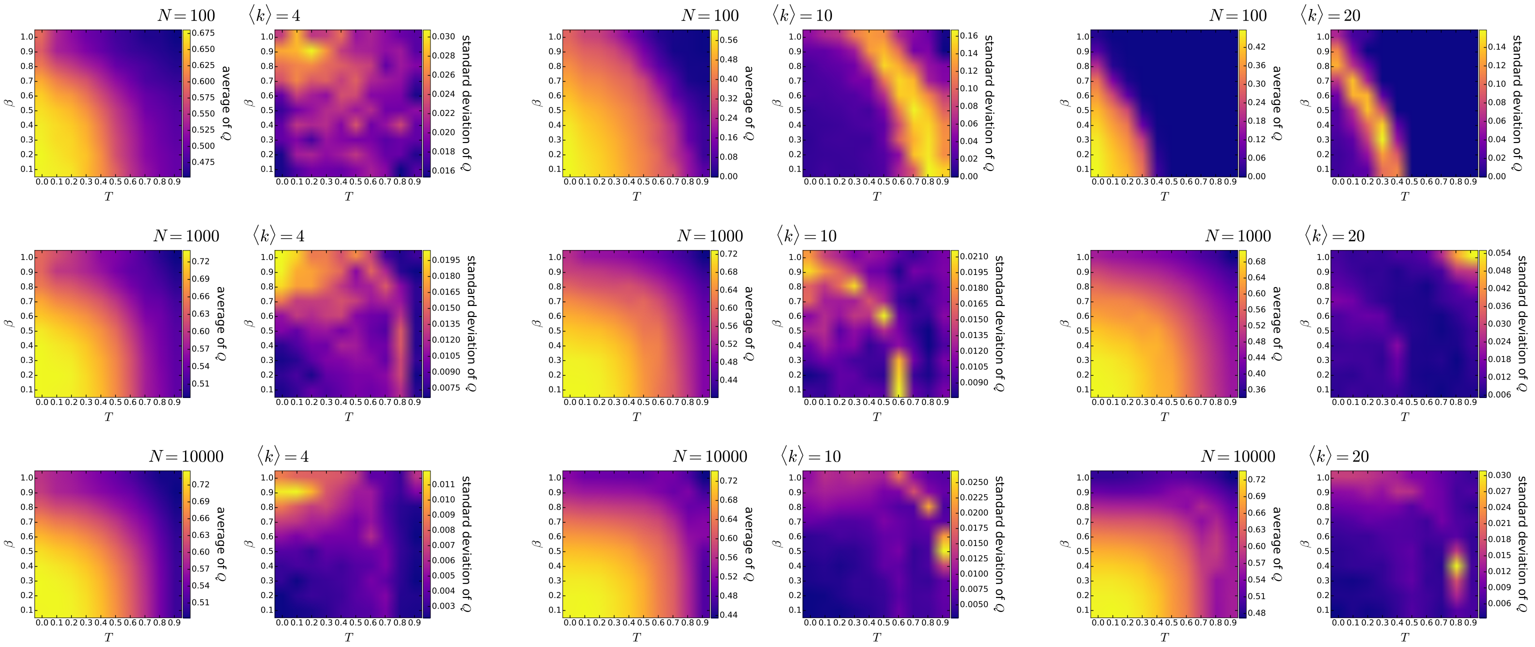

Figure F3. The mean and the standard deviation of the unweighted modularity Q of the community structure detected by the *Infomap* algorithm in 100 *unweighted PSO* networks of different parametrisations. Each pair of subplots depicts the effect of changing the popularity fading parameter β and the temperature T, with the number of nodes N and the expected average degree $\langle k \rangle = 2m$ given in the title of the subplot pair. The curvature of the hyperbolic plane K was always set to -1, i.e. we used $\zeta = 1$.

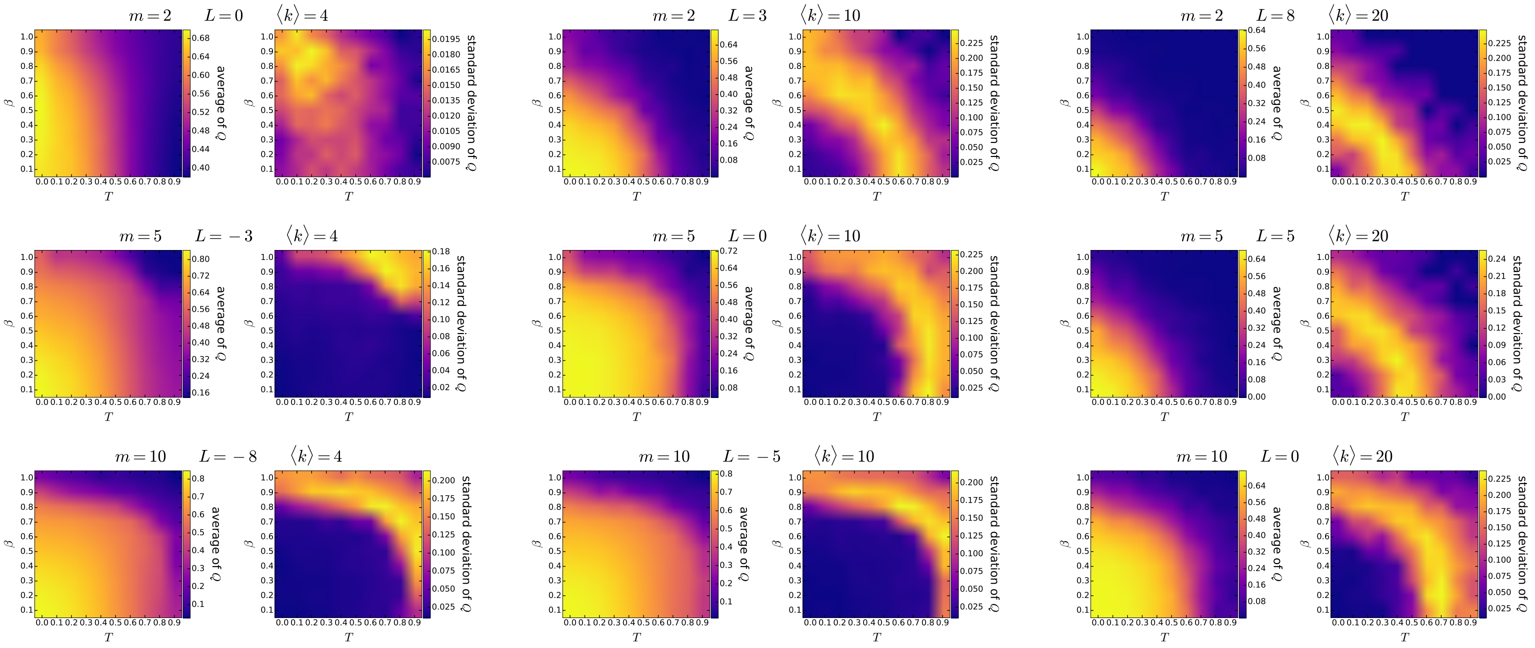

Figure F4. The mean and the standard deviation of the unweighted modularity Q of the community structure detected by the *asynchronous label propagation* algorithm in 100 *unweighted E-PSO* networks of different parametrisations. Each pair of subplots depicts the effect of changing the popularity fading parameter β and the temperature T, with the parameters m and L given in the title of the subplot pair together with the corresponding expected average degree $\langle k \rangle = 2(m+L)$. The number of nodes N was 1000 in each case. The curvature of the hyperbolic plane K was always set to -1, i.e. we used $\zeta = 1$.

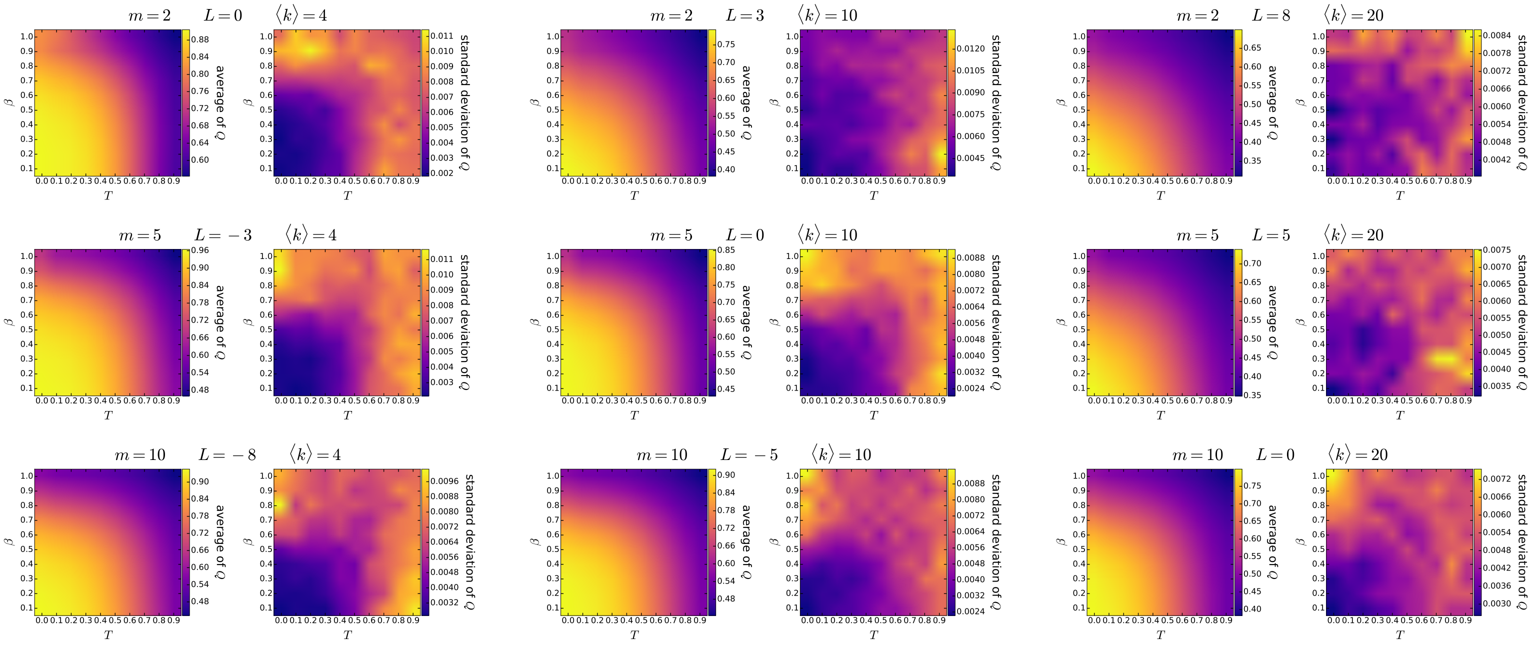

Figure F5. The mean and the standard deviation of the unweighted modularity Q of the community structure detected by the *Louvain* algorithm in 100 *unweighted E-PSO* networks of different parametrisations. Each pair of subplots depicts the effect of changing the popularity fading parameter β and the temperature T, with the parameters m and L given in the title of the subplot pair together with the corresponding expected average degree $\langle k \rangle = 2(m+L)$. The number of nodes N was 1000 in each case. The curvature of the hyperbolic plane K was always set to -1, i.e. we used $\zeta = 1$.

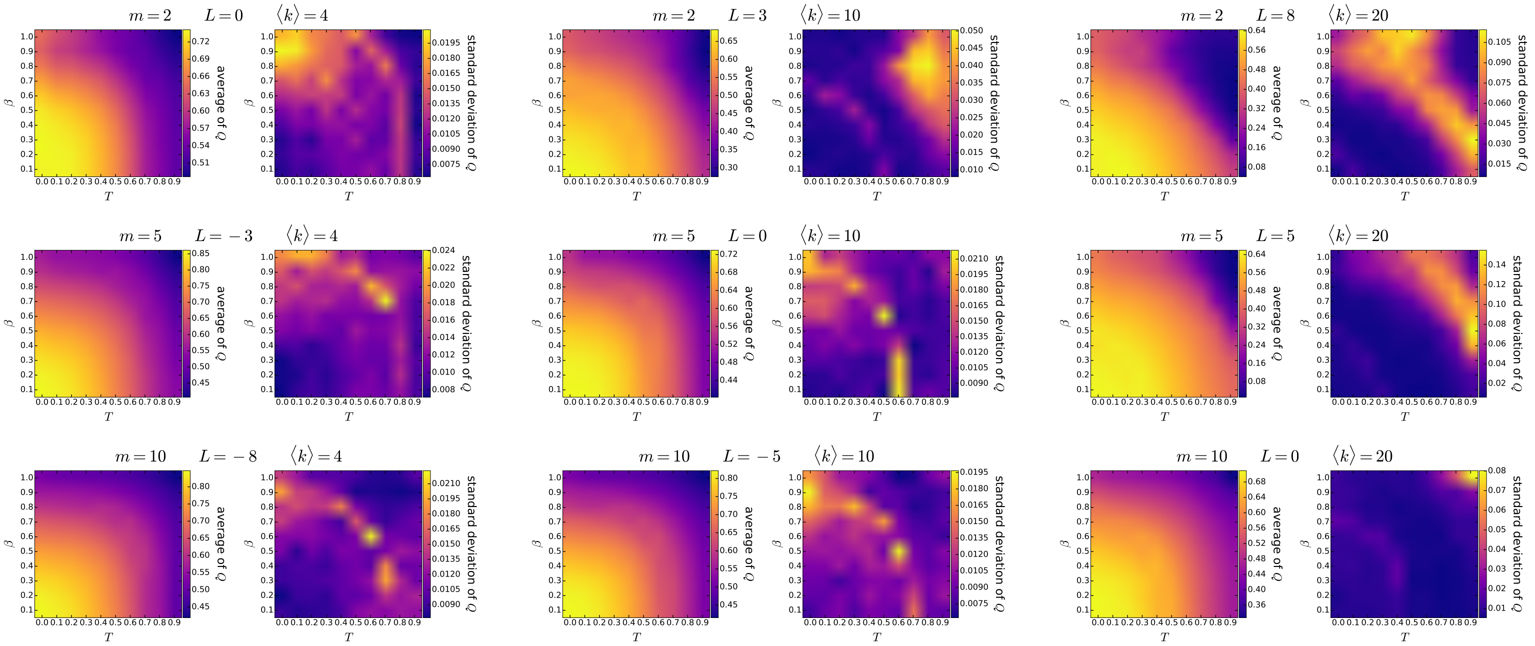

Figure F6. The mean and the standard deviation of the unweighted modularity Q of the community structure detected by the *Infomap* algorithm in 100 *unweighted E-PSO* networks of different parametrisations. Each pair of subplots depicts the effect of changing the popularity fading parameter β and the temperature T, with the parameters m and L given in the title of the subplot pair together with the corresponding expected average degree $\langle k \rangle = 2(m+L)$. The number of nodes N was 1000 in each case. The curvature of the hyperbolic plane K was always set to -1, i.e. we used $\zeta = 1$.

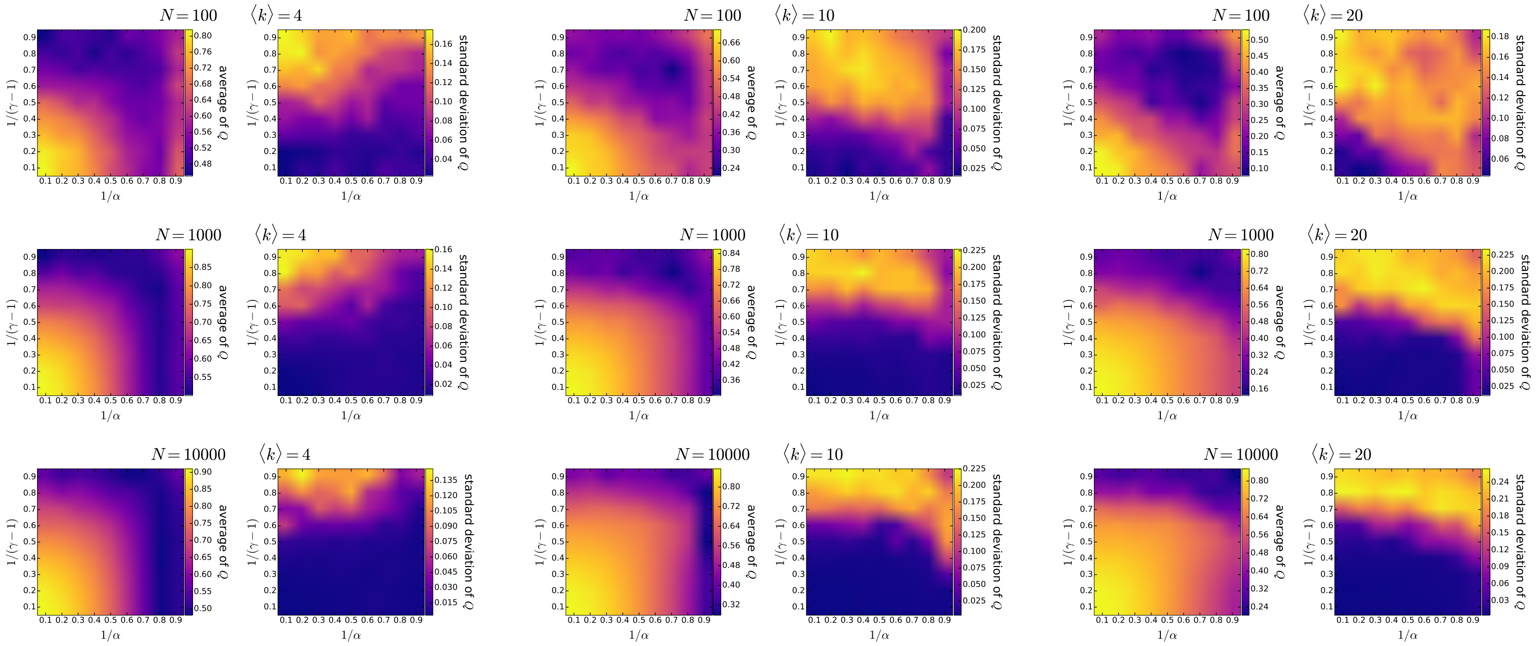

Figure F7. The mean and the standard deviation of the unweighted modularity Q of the community structure detected by the *asynchronous label propagation* algorithm in 100 *unweighted* $\mathbb{S}^1/\mathbb{H}^2$ networks of different parametrisations. Each pair of subplots depicts the effect of changing $1/(\gamma - 1)$ (equivalent to the popularity fading parameter β in the E-PSO model) and $1/\alpha$ (analogous to the temperature T in the E-PSO model), with the number of nodes N and the expected average degree $\langle k \rangle$ given in the title of the subplot pair. We used $K = -1$ as the curvature of the hyperbolic plane in each case.

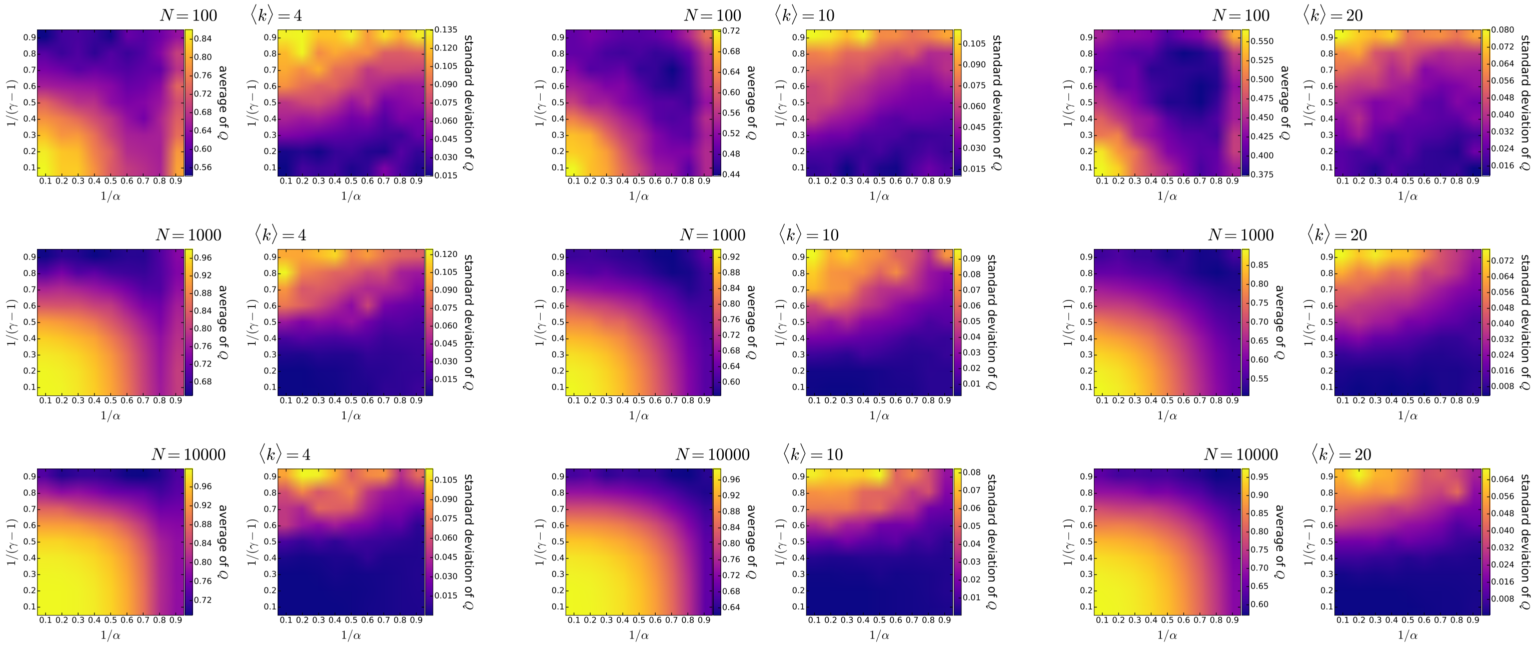

Figure F8. The mean and the standard deviation of the unweighted modularity Q of the community structure detected by the *Louvain* algorithm in 100 *unweighted* $\mathbb{S}^1/\mathbb{H}^2$ networks of different parametrisations. Each pair of subplots depicts the effect of changing $1/(\gamma - 1)$ (equivalent to the popularity fading parameter β in the E-PSO model) and $1/\alpha$ (analogous to the temperature T in the E-PSO model), with the number of nodes N and the expected average degree $\langle k \rangle$ given in the title of the subplot pair. We used $K = -1$ as the curvature of the hyperbolic plane in each case.

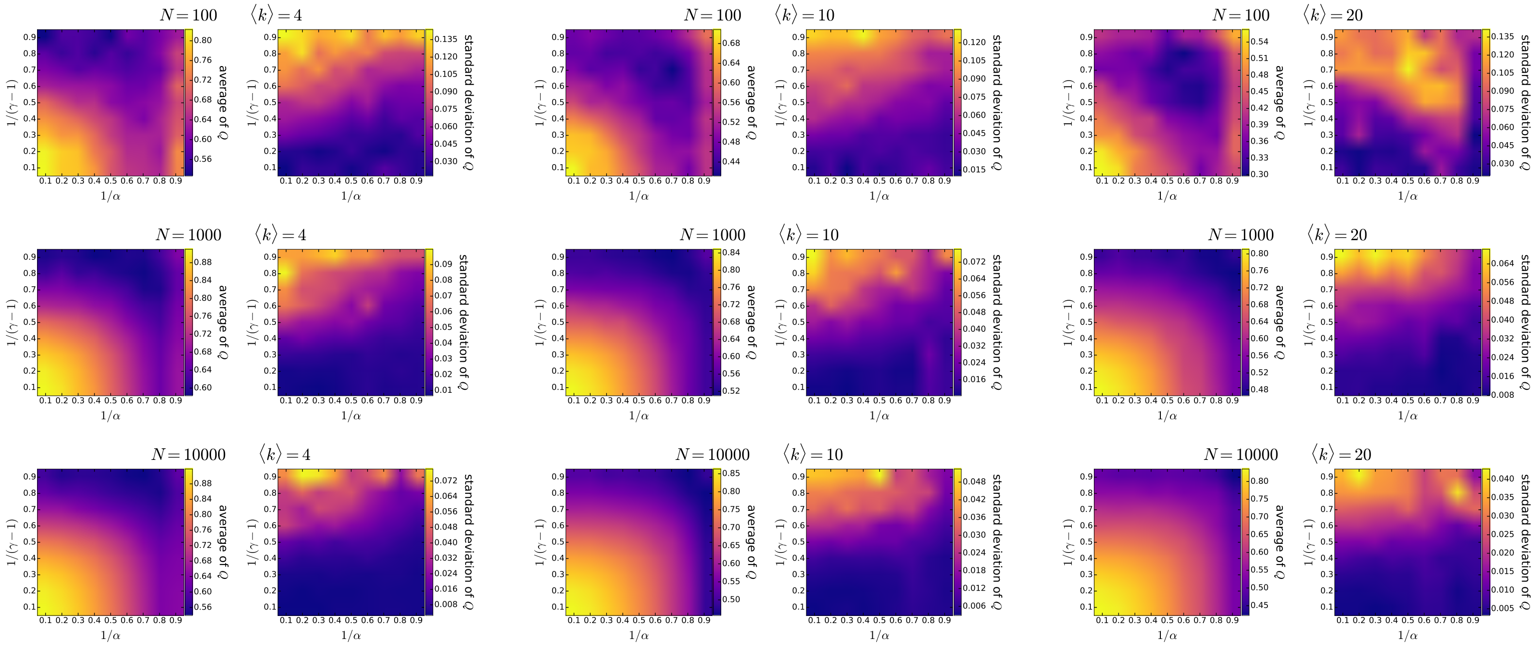

Figure F9. The mean and the standard deviation of the unweighted modularity Q of the community structure detected by the *Infomap* algorithm in 100 *unweighted* $\mathbb{S}^1/\mathbb{H}^2$ networks of different parametrisations. Each pair of subplots depicts the effect of changing $1/(\gamma-1)$ (equivalent to the popularity fading parameter β in the E-PSO model) and $1/\alpha$ (analogous to the temperature T in the E-PSO model), with the number of nodes N and the expected average degree $\langle k\rangle$ given in the title of the subplot pair. We used $K=-1$ as the curvature of the hyperbolic plane in each case.

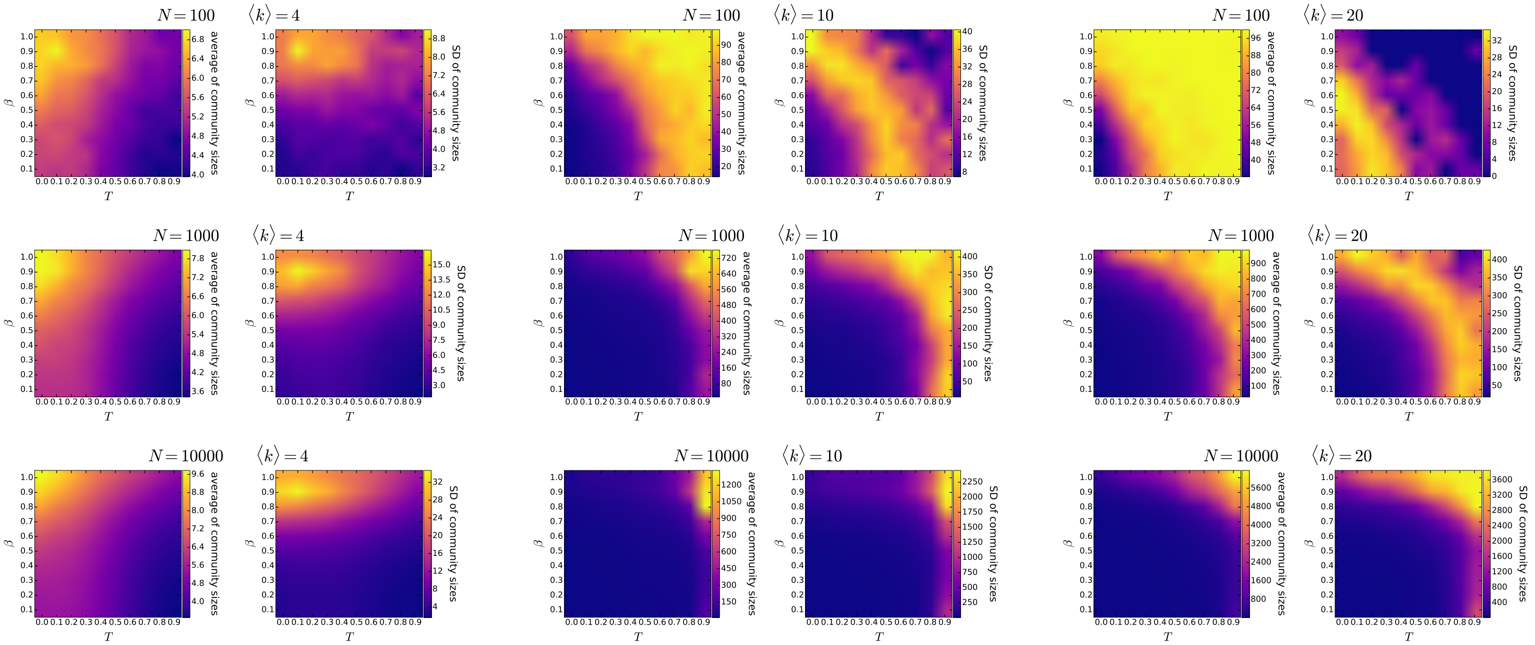

Figure F10. The mean and the standard deviation of the size of communities detected by the *asynchronous label propagation* algorithm in 100 *unweighted PSO* networks of different parametrisations. Each pair of subplots depicts the effect of changing the popularity fading parameter β and the temperature T, with the number of nodes N and the expected average degree $\langle k \rangle = 2m$ given in the title of the subplot pair. The curvature of the hyperbolic plane K was always set to -1, i.e. we used $\zeta = 1$.

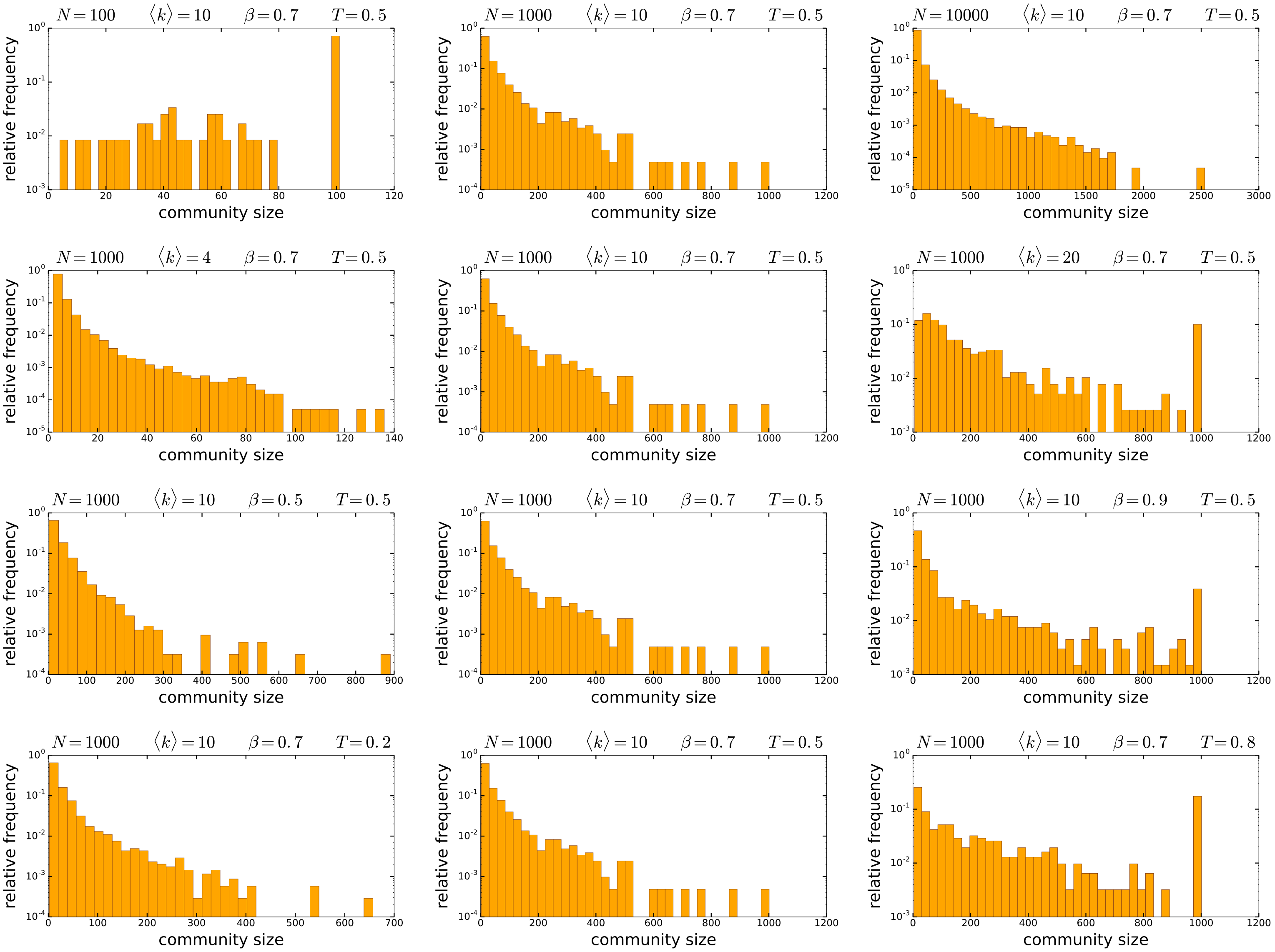

Figure F11. The size distribution of the communities detected by the *asynchronous label propagation* algorithm in 100 *unweighted PSO* networks of different parametrisations. The parameters of the network generation are listed in the title for each subplot. The curvature of the hyperbolic plane K was always set to -1, i.e. we used $\zeta = 1$. Each row of the figure demonstrates the effect of the change in a given network generation parameter: from top to bottom, the number of nodes N, the expected average degree $\langle k \rangle = 2m$, the popularity fading parameter β and the temperature T.

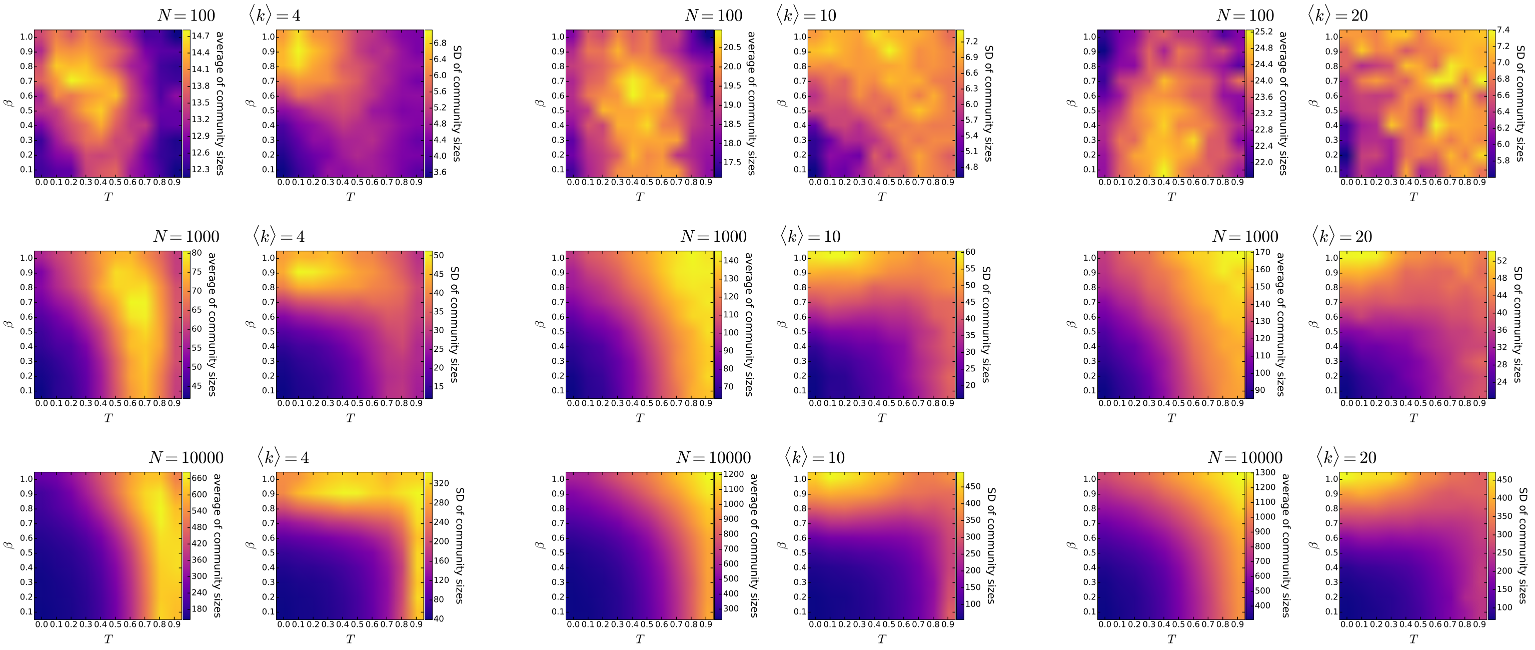

Figure F12. The mean and the standard deviation of the size of communities detected by the *Louvain* algorithm in 100 *unweighted PSO* networks of different parametrisations. Each pair of subplots depicts the effect of changing the popularity fading parameter β and the temperature T, with the number of nodes N and the expected average degree $\langle k \rangle = 2m$ given in the title of the subplot pair. The curvature of the hyperbolic plane K was always set to -1, i.e. we used $\zeta = 1$.

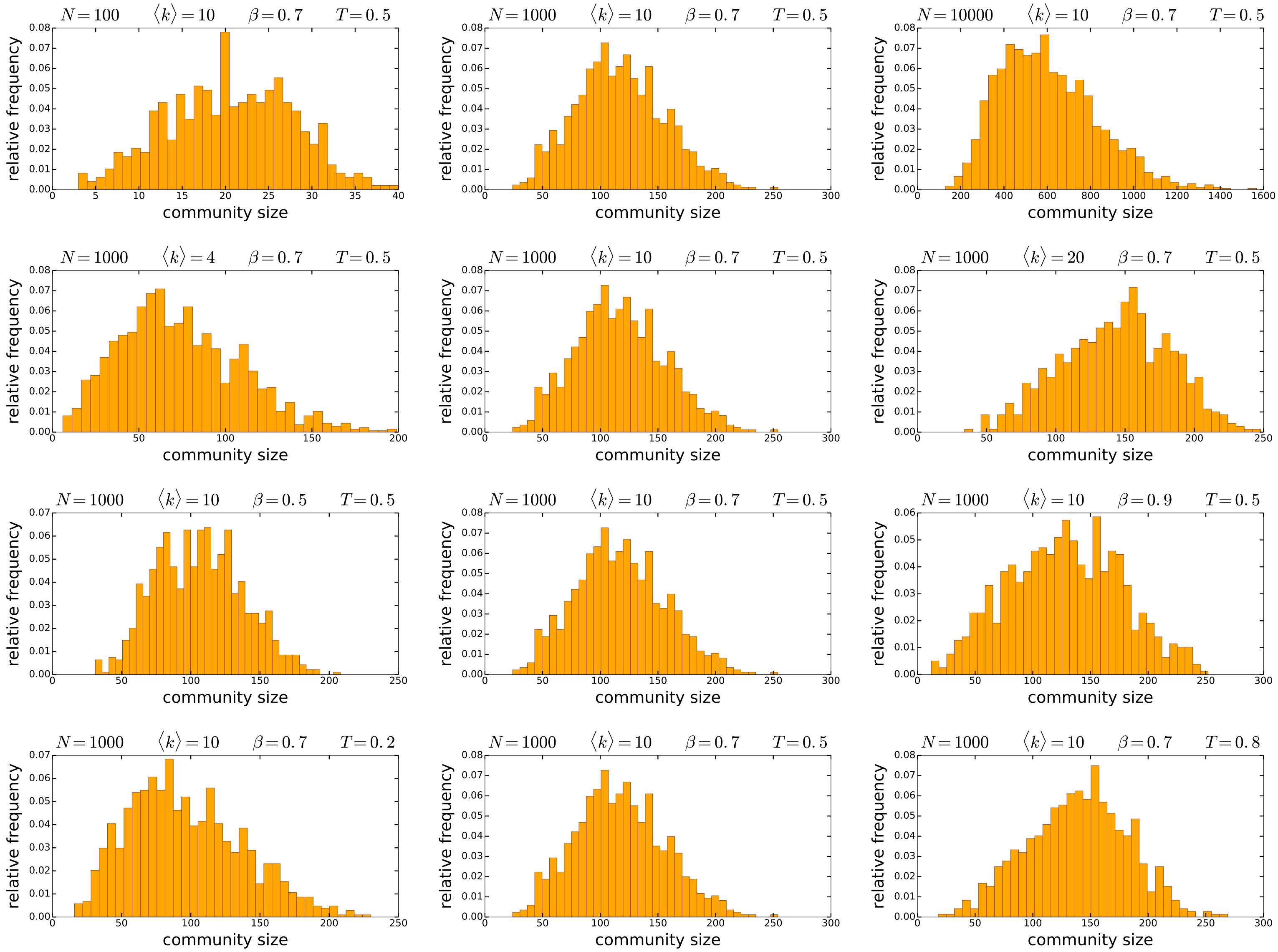

Figure F13. The size distribution of the communities detected by the *Louvain* algorithm in 100 *unweighted PSO* networks of different parametrisations. The parameters of the network generation are listed in the title for each subplot. The curvature of the hyperbolic plane K was always set to -1, i.e. we used $\zeta = 1$. Each row of the figure demonstrates the effect of the change in a given network generation parameter: from top to bottom, the number of nodes N, the expected average degree $\langle k \rangle = 2m$, the popularity fading parameter β and the temperature T.

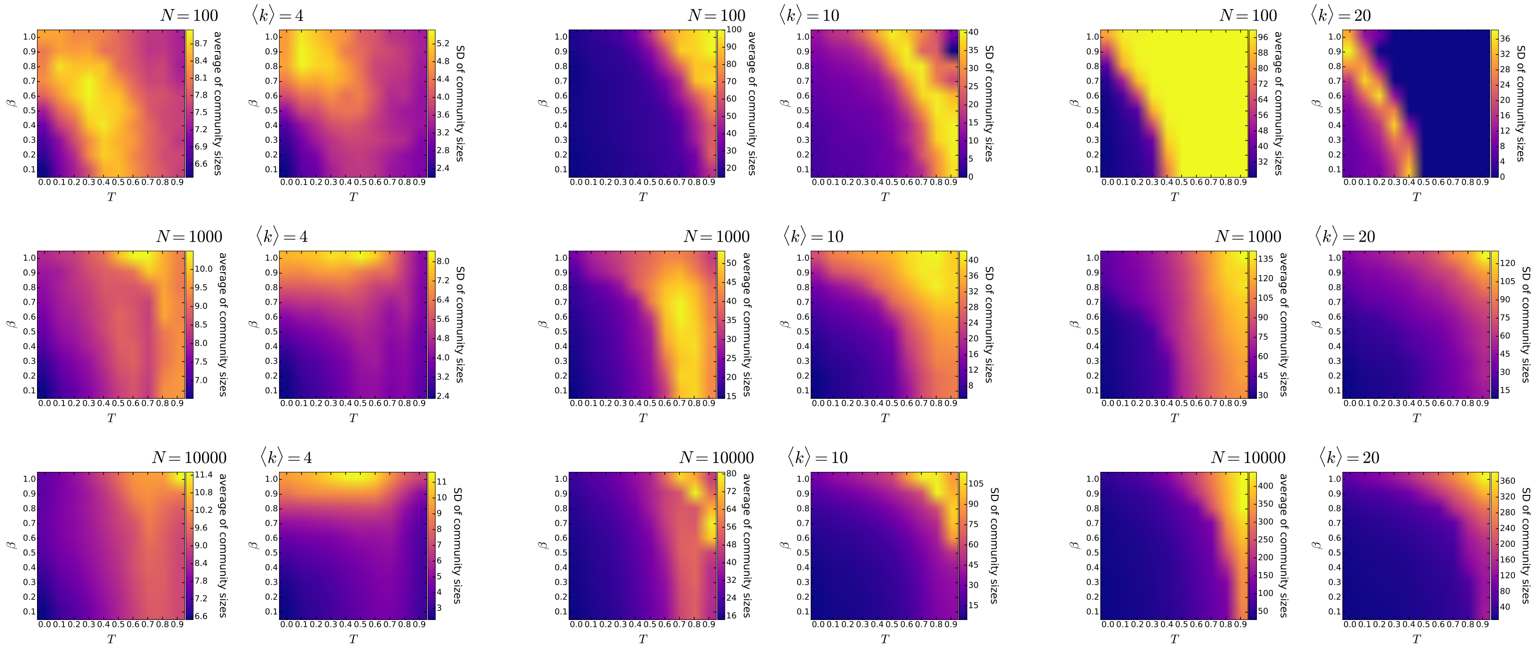

Figure F14. The mean and the standard deviation of the size of communities detected by the *Infomap* algorithm in 100 *unweighted PSO* networks of different parametrisations. Each pair of subplots depicts the effect of changing the popularity fading parameter β and the temperature T, with the number of nodes N and the expected average degree $\langle k \rangle = 2m$ given in the title of the subplot pair. The curvature of the hyperbolic plane K was always set to -1, i.e. we used $\zeta = 1$.

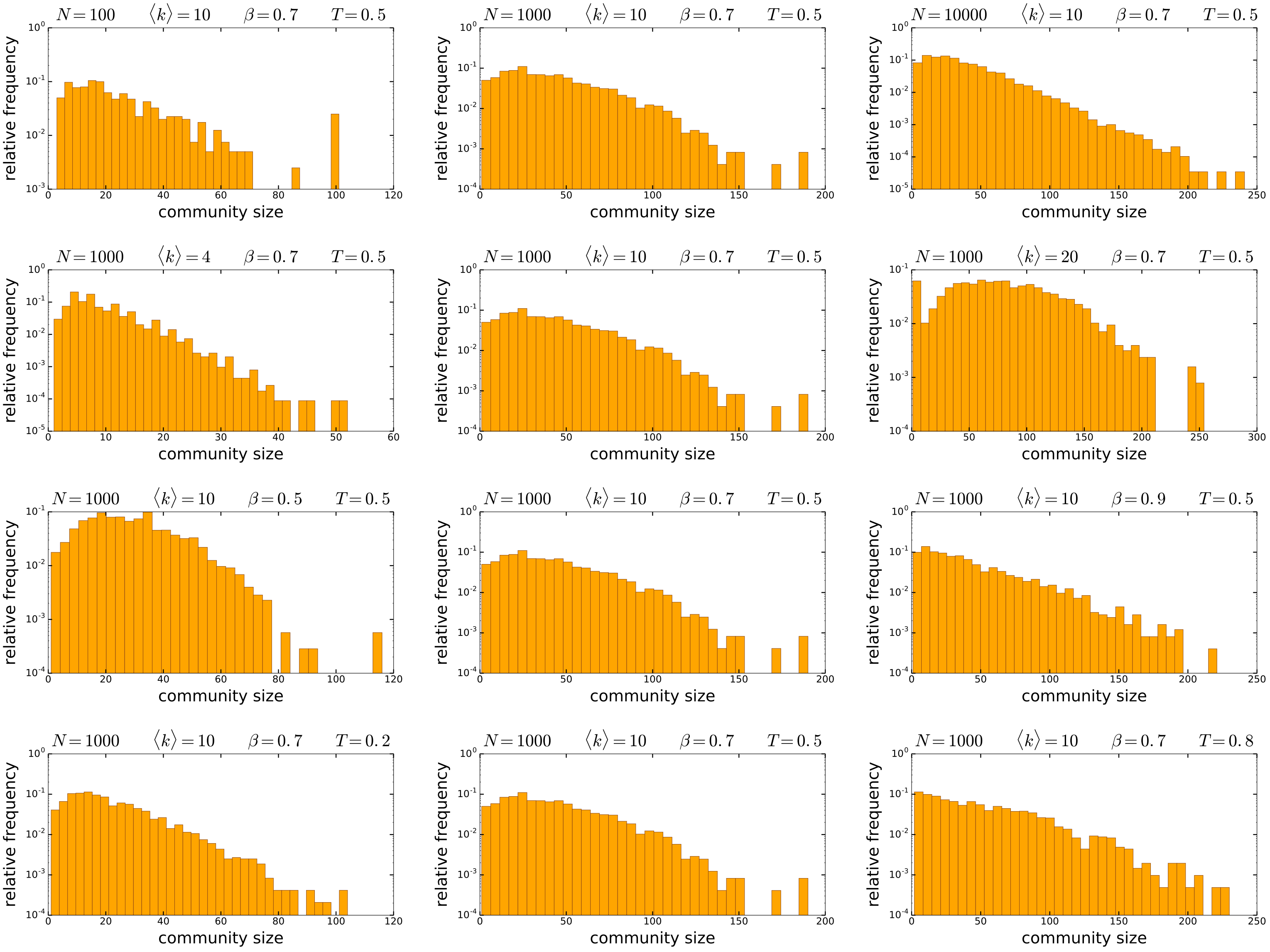

Figure F15. The size distribution of the communities detected by the *Infomap* algorithm in 100 *unweighted PSO* networks of different parametrisations. The parameters of the network generation are listed in the title for each subplot. The curvature of the hyperbolic plane K was always set to -1, i.e. we used $\zeta = 1$. Each row of the figure demonstrates the effect of the change in a given network generation parameter: from top to bottom, the number of nodes N, the expected average degree $\langle k \rangle = 2m$, the popularity fading parameter β and the temperature T.

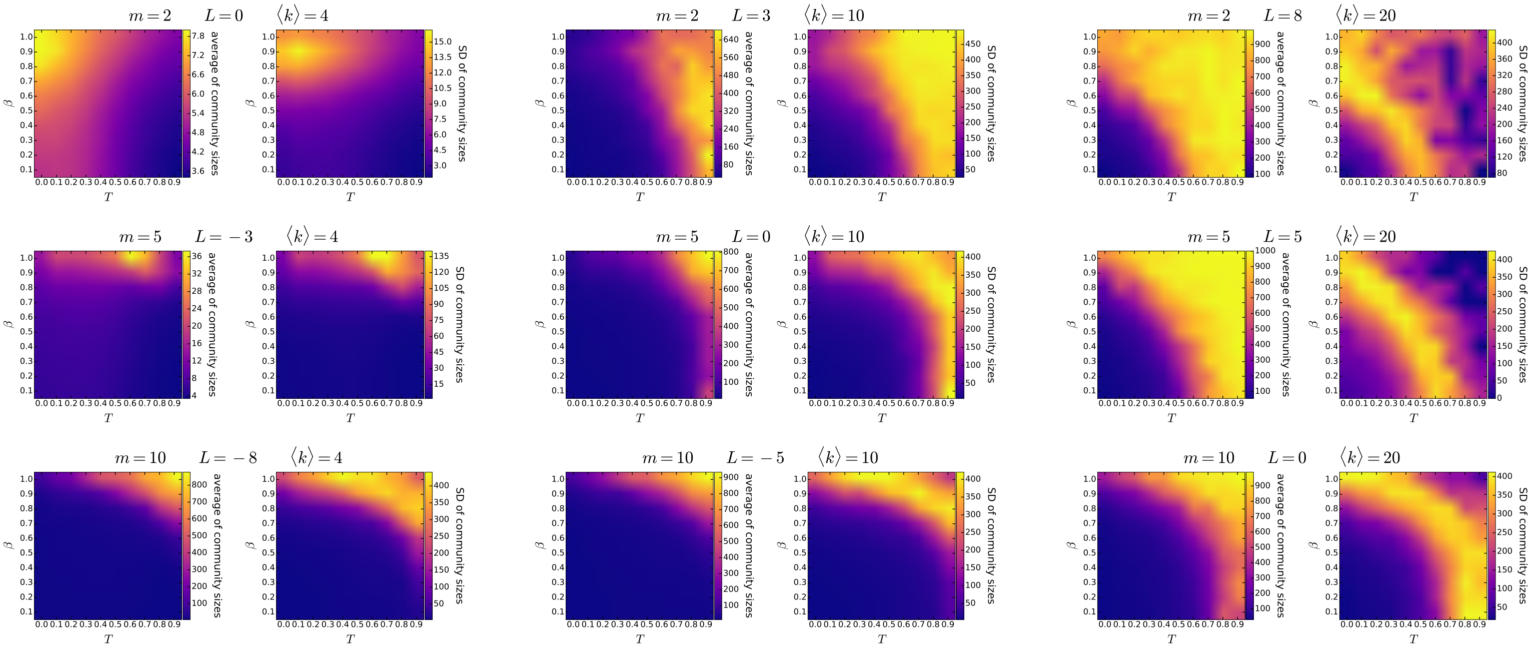

Figure F16. The mean and the standard deviation of the size of communities detected by the *asynchronous label propagation* algorithm in 100 *unweighted E-PSO* networks of different parametrisations. Each pair of subplots depicts the effect of changing the popularity fading parameter β and the temperature T, with the parameters m and L given in the title of the subplot pair together with the corresponding expected average degree $\langle k \rangle = 2(m+L)$. The number of nodes N was 1000 in each case. The curvature of the hyperbolic plane K was always set to -1, i.e. we used $\zeta = 1$.

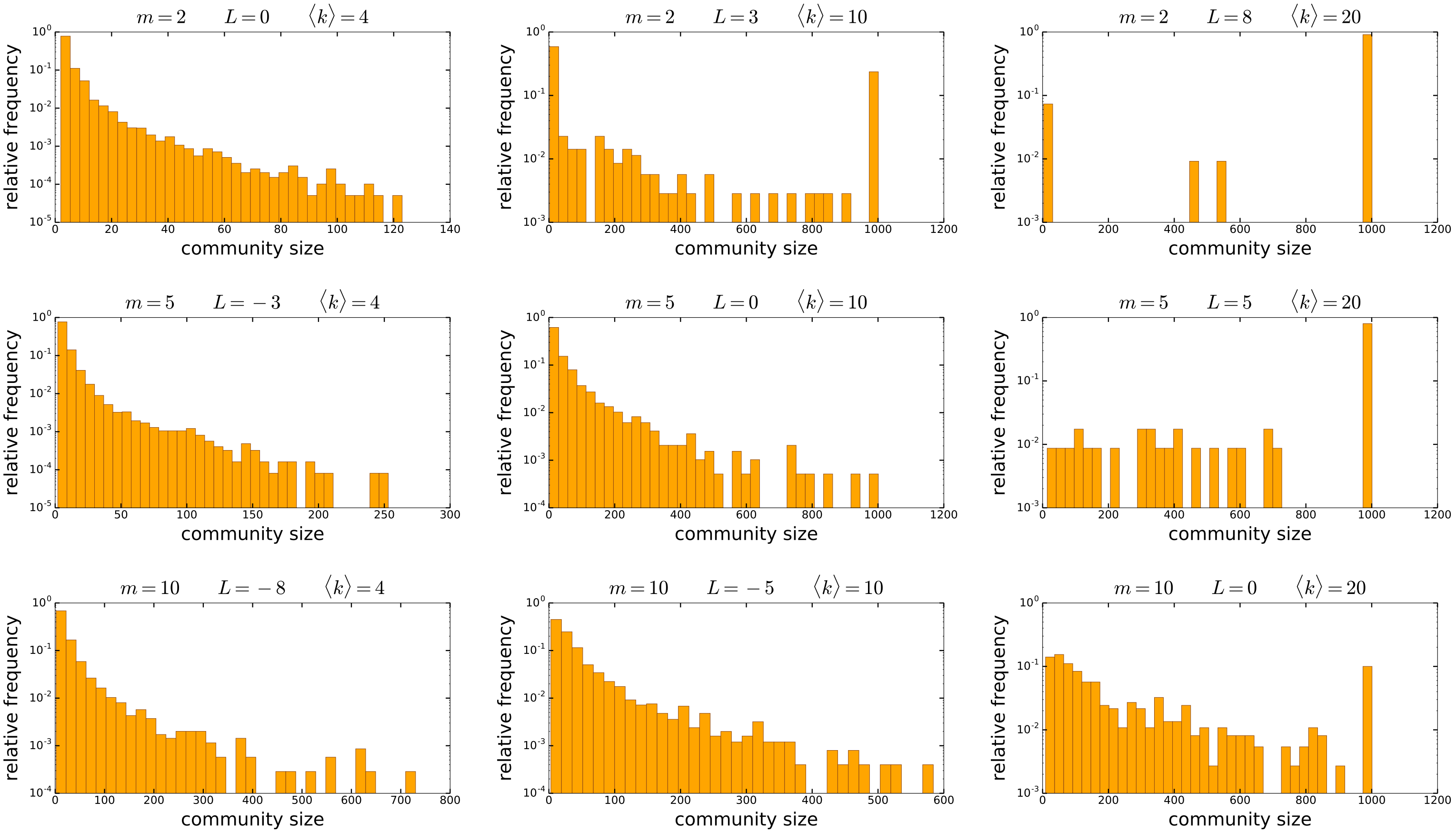

Figure F17. The size distribution of the communities detected by the *asynchronous label propagation* algorithm in 100 *unweighted E-PSO* networks of different parametrisations. We used $\zeta = 1$, i.e. $K = -1$ as the curvature of the hyperbolic plane, the number of nodes N was 1000, the popularity fading parameter β was 0.7 and the temperature T was 0.5 in each case. The parameters m and L are given in the title for each subplot together with the corresponding expected average degree $\langle k\rangle = 2(m+L)$.

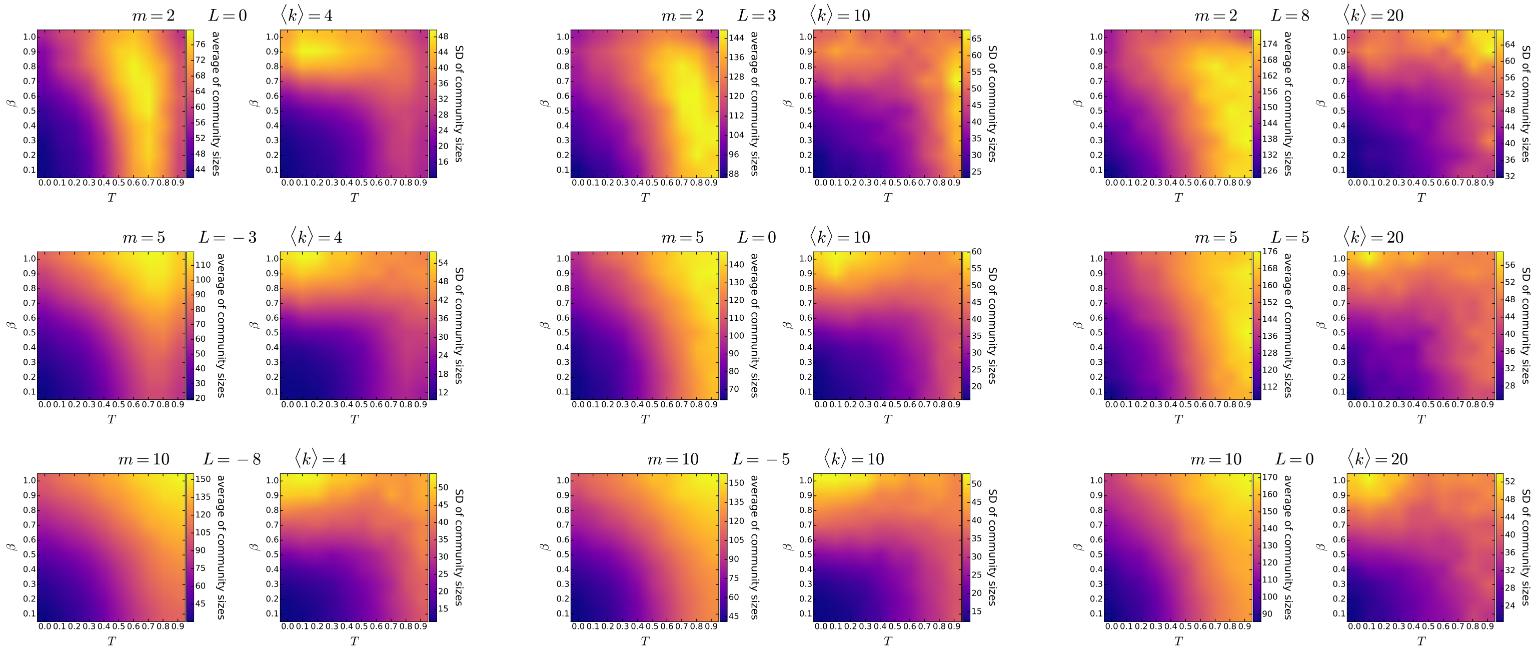

Figure F18. The mean and the standard deviation of the size of communities detected by the *Louvain* algorithm in 100 *unweighted E-PSO* networks of different parametrisations. Each pair of subplots depicts the effect of changing the popularity fading parameter β and the temperature T, with the parameters m and L given in the title of the subplot pair together with the corresponding expected average degree $\langle k \rangle = 2(m+L)$. The number of nodes N was 1000 in each case. The curvature of the hyperbolic plane K was always set to -1, i.e. we used $\zeta = 1$.

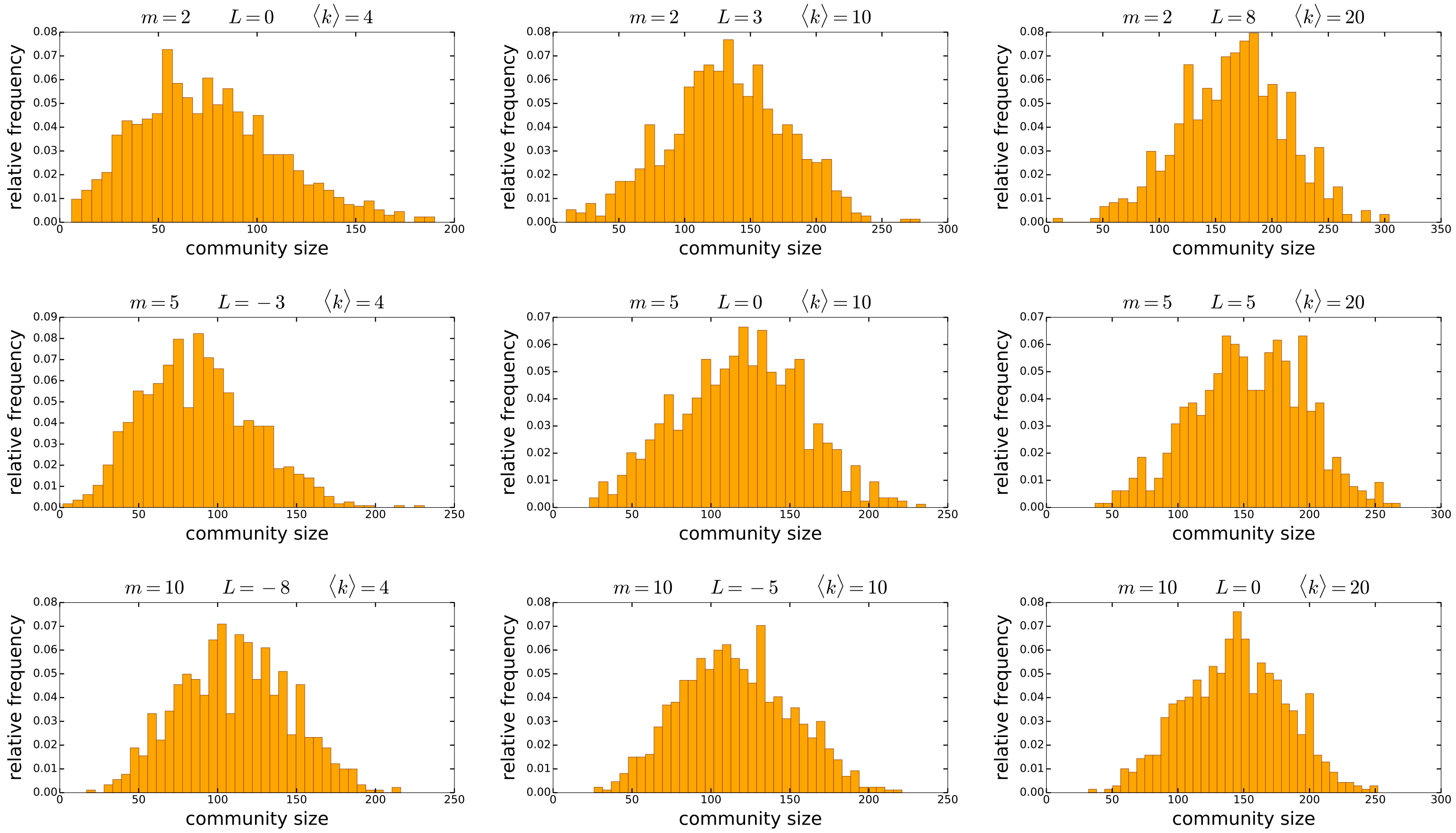

Figure F19. The size distribution of the communities detected by the *Louvain* algorithm in 100 *unweighted E-PSO* networks of different parametrisations. We used $\zeta = 1$, i.e. $K = -1$ as the curvature of the hyperbolic plane, the number of nodes N was 1000, the popularity fading parameter β was 0.7 and the temperature T was 0.5 in each case. The parameters m and L are given in the title for each subplot together with the corresponding expected average degree $\langle k \rangle = 2(m+L)$.

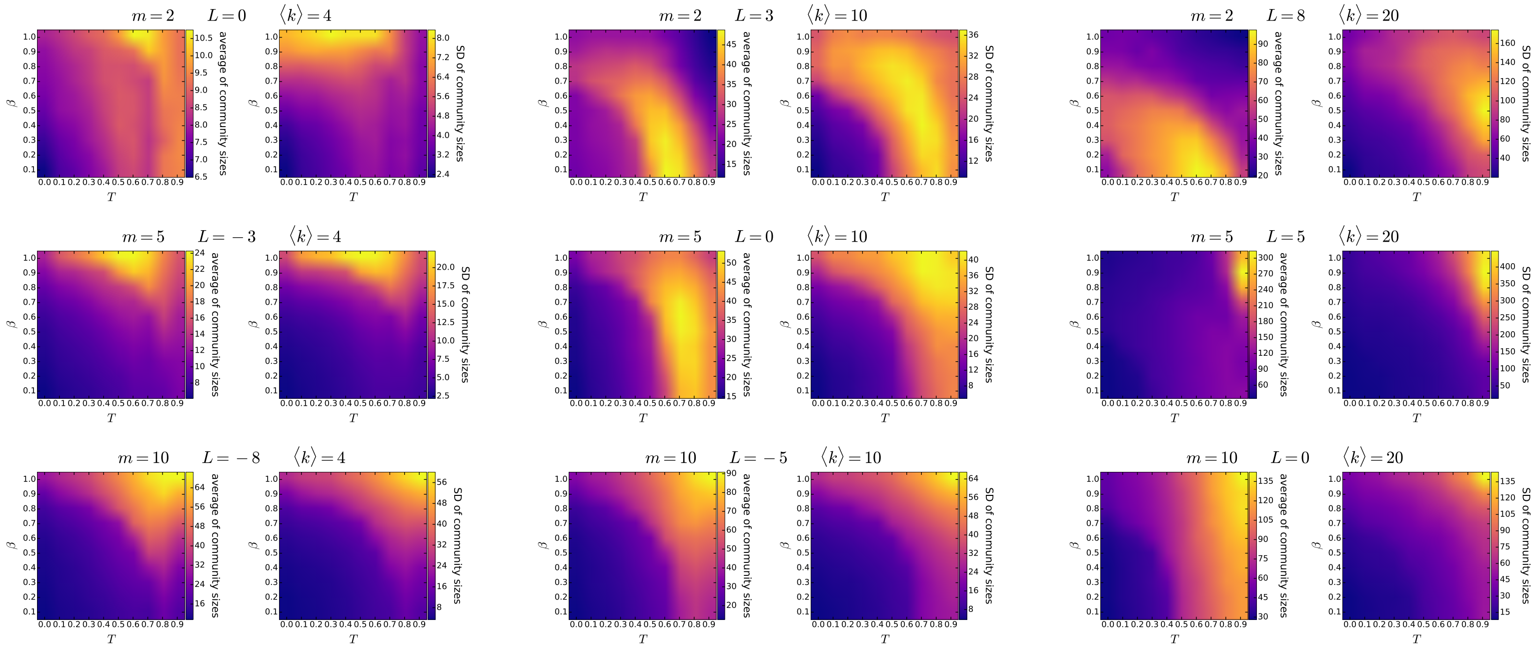

Figure F20. The mean and the standard deviation of the size of communities detected by the *Infomap* algorithm in 100 *unweighted E-PSO* networks of different parametrisations. Each pair of subplots depicts the effect of changing the popularity fading parameter β and the temperature T, with the parameters m and L given in the title of the subplot pair together with the corresponding expected average degree $\langle k \rangle = 2(m+L)$. The number of nodes N was 1000 in each case. The curvature of the hyperbolic plane K was always set to -1, i.e. we used $\zeta = 1$.

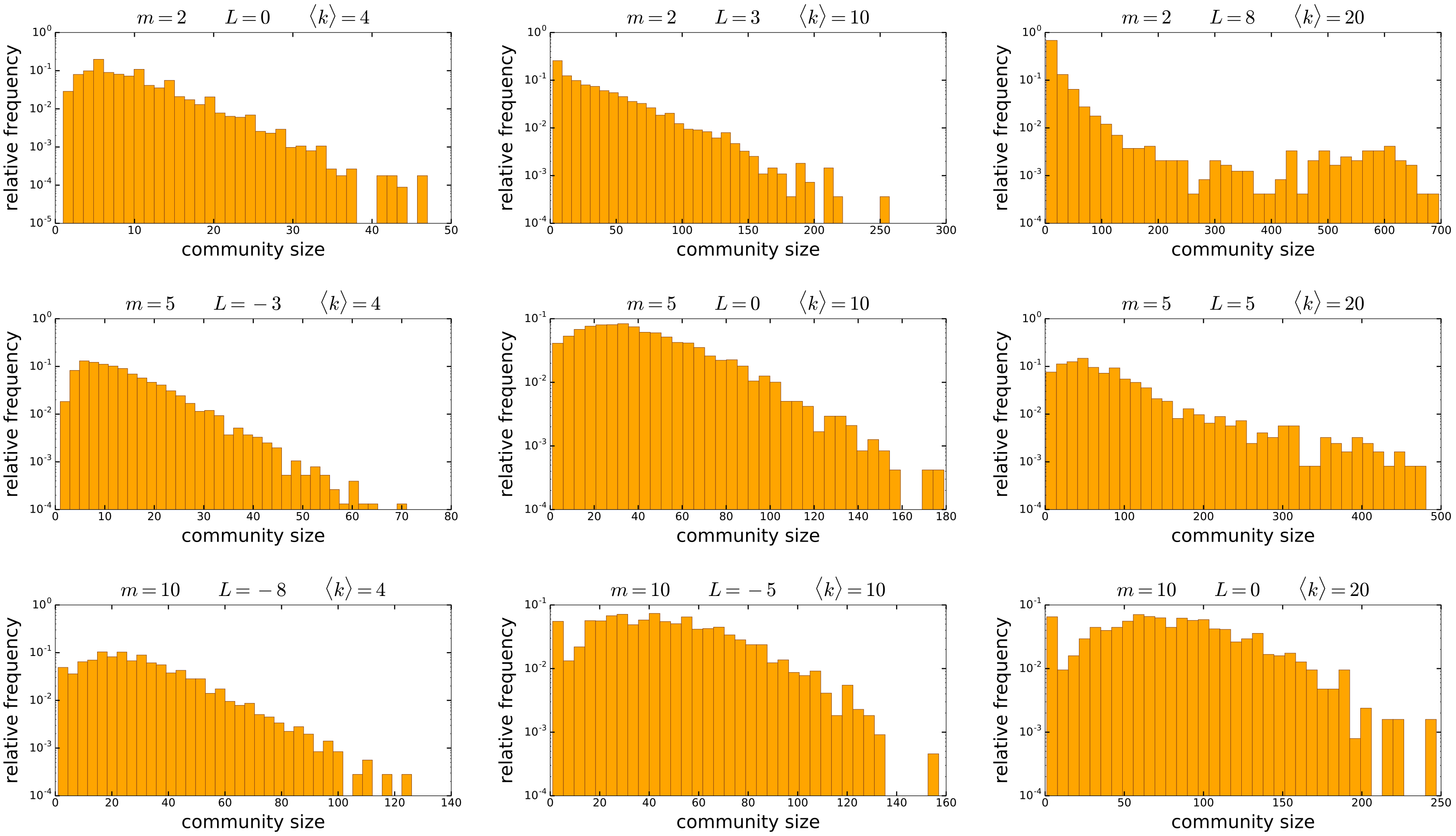

Figure F21. The size distribution of the communities detected by the *Infomap* algorithm in 100 *unweighted E-PSO* networks of different parametrisations. We used $\zeta = 1$, i.e. $K = -1$ as the curvature of the hyperbolic plane, the number of nodes N was 1000, the popularity fading parameter β was 0.7 and the temperature T was 0.5 in each case. The parameters m and L are given in the title for each subplot together with the corresponding expected average degree $\langle k\rangle = 2(m+L)$.

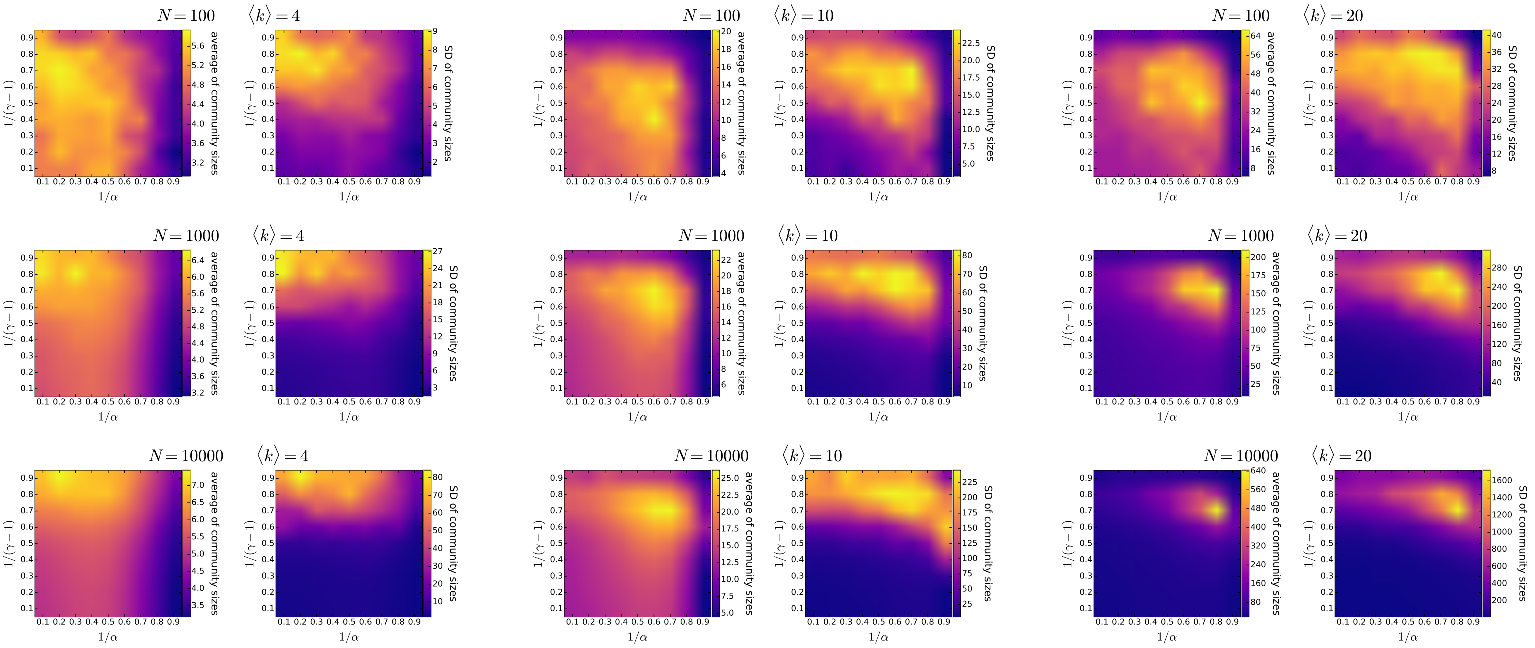

Figure F22. The mean and the standard deviation of the size of communities detected by the *asynchronous label propagation* algorithm in 100 *unweighted* $\mathbb{S}^1/\mathbb{H}^2$ networks of different parametrisations. Each pair of subplots depicts the effect of changing $1/(\gamma-1)$ (equivalent to the popularity fading parameter β in the E-PSO model) and $1/\alpha$ (analogous to the temperature T in the E-PSO model), with the number of nodes N and the expected average degree $\langle k \rangle$ given in the title of the subplot pair. We used $K=-1$ as the curvature of the hyperbolic plane in each case.

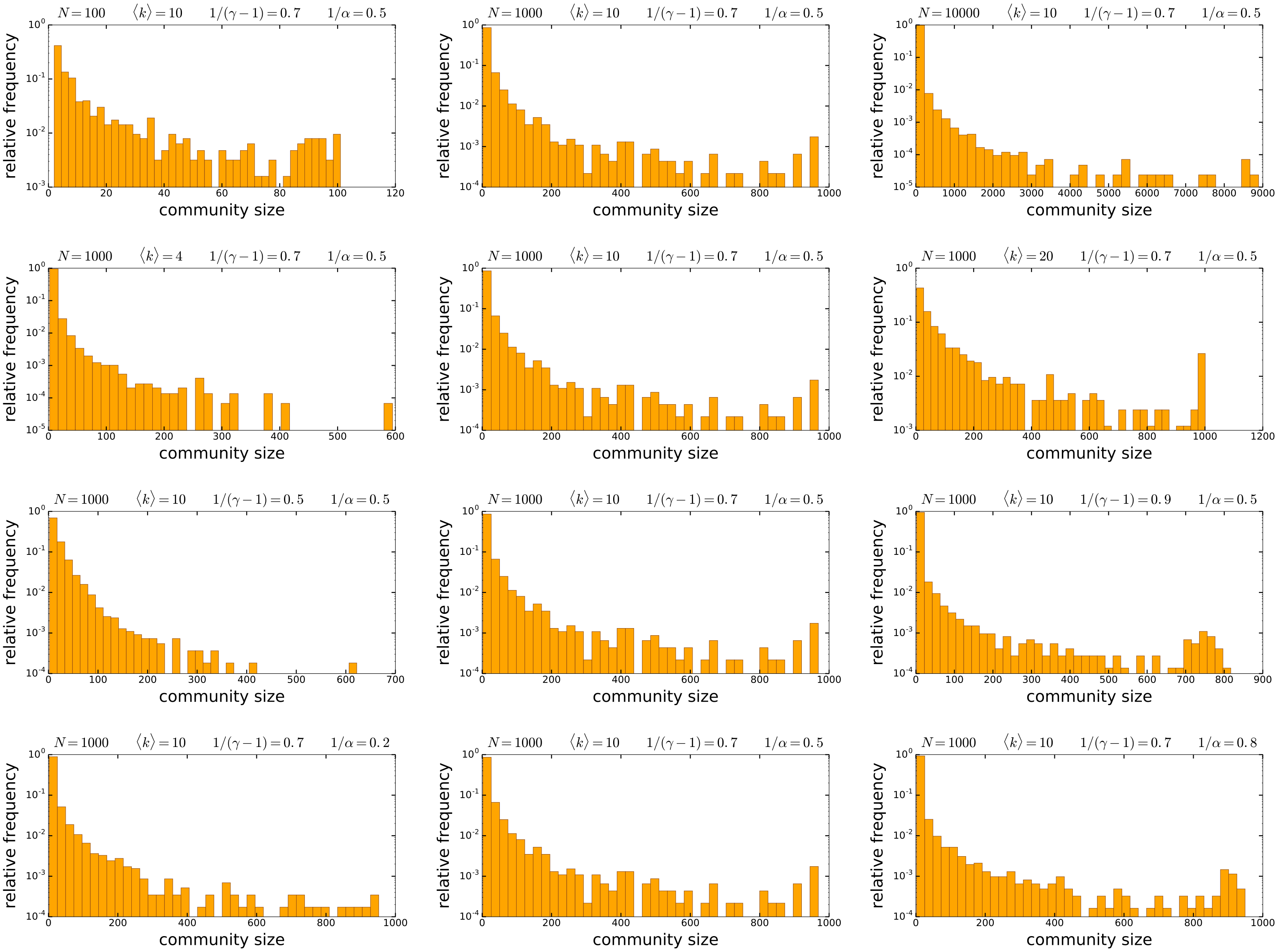

Figure F23. The size distribution of the communities detected by the *asynchronous label propagation* algorithm in 100 *unweighted* $\mathbb{S}^1/\mathbb{H}^2$ networks of different parametrisations. The parameters of the network generation are listed in the title for each subplot. We used $K=-1$ as the curvature of the hyperbolic plane in each case. Each row of the figure demonstrates the effect of the change in a given network generation parameter: from top to bottom, the number of nodes N, the expected average degree $\langle k\rangle$, $1/(\gamma-1)$ (equivalent to the popularity fading parameter β in the E-PSO model) and $1/\alpha$ (analogous to the temperature T in the E-PSO model).

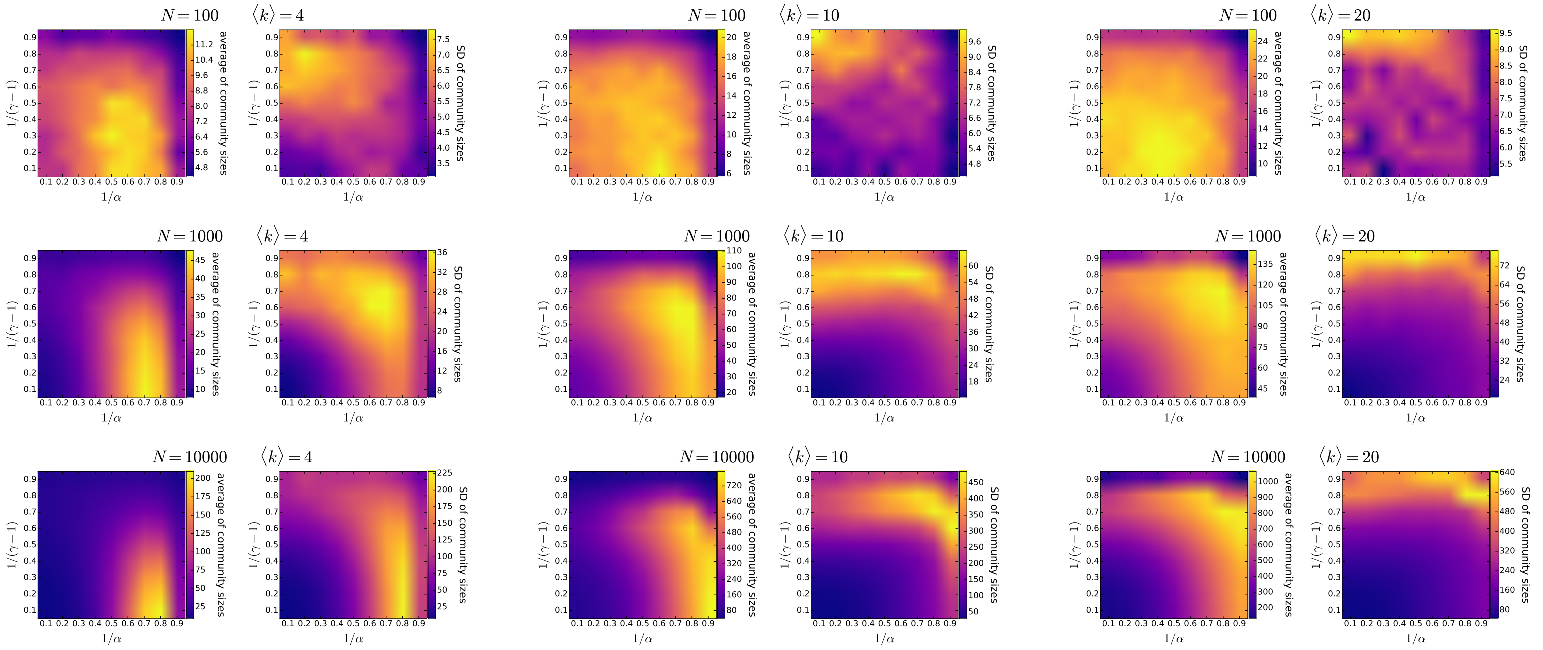

Figure F24. The mean and the standard deviation of the size of communities detected by the *Louvain* algorithm in 100 *unweighted* $\mathbb{S}^1/\mathbb{H}^2$ networks of different parametrisations. Each pair of subplots depicts the effect of changing $1/(\gamma-1)$ (equivalent to the popularity fading parameter β in the E-PSO model) and $1/\alpha$ (analogous to the temperature T in the E-PSO model), with the number of nodes N and the expected average degree $\langle k \rangle$ given in the title of the subplot pair. We used $K=-1$ as the curvature of the hyperbolic plane in each case.

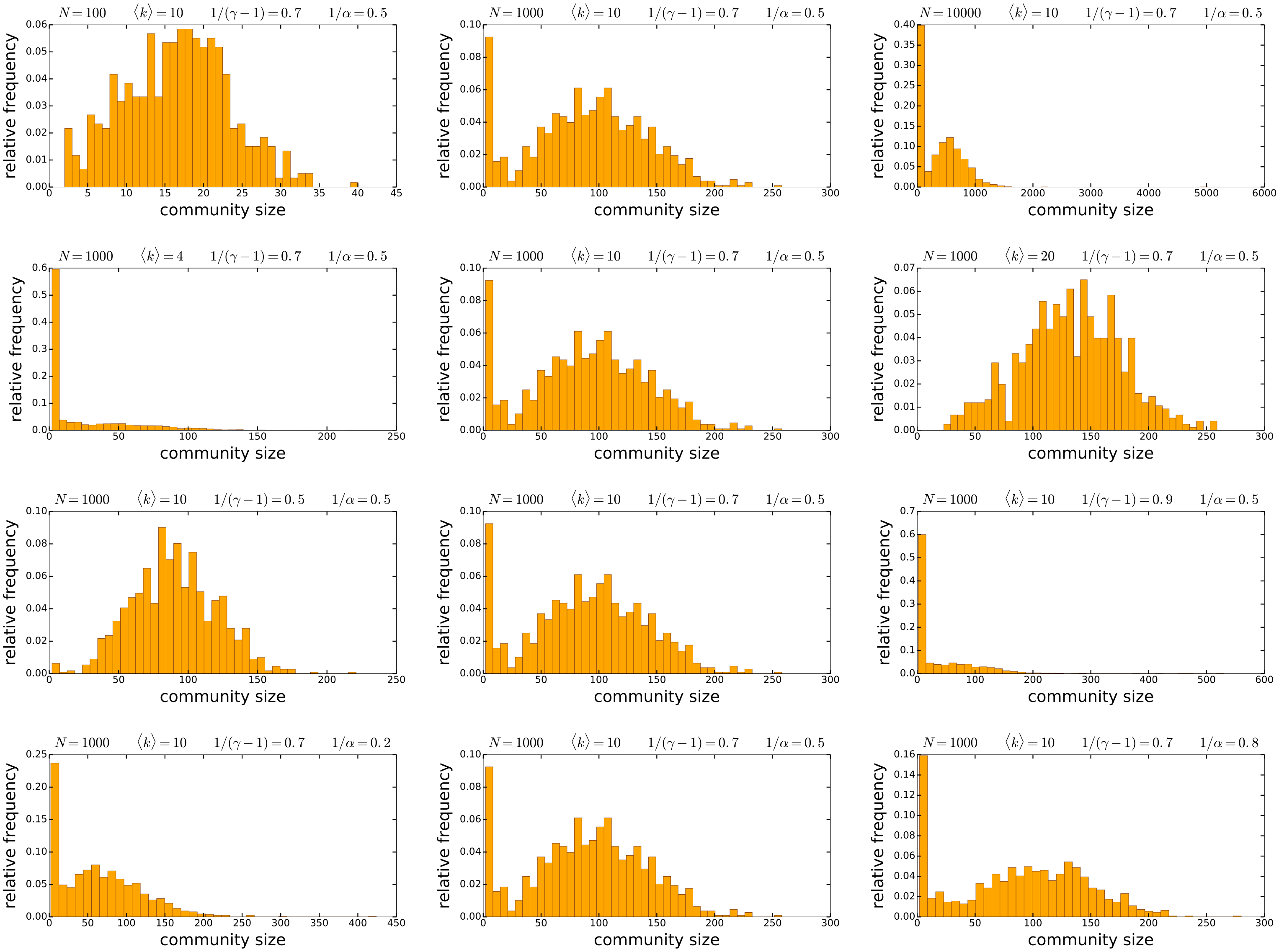

Figure F25. The size distribution of the communities detected by the *Louvain* algorithm in 100 *unweighted* $\mathbb{S}^1/\mathbb{H}^2$ networks of different parametrisations. The parameters of the network generation are listed in the title for each subplot. We used $K=-1$ as the curvature of the hyperbolic plane in each case. Each row of the figure demonstrates the effect of the change in a given network generation parameter: from top to bottom, the number of nodes N, the expected average degree $\langle k\rangle$, $1/(\gamma-1)$ (equivalent to the popularity fading parameter β in the E-PSO model) and $1/\alpha$ (analogous to the temperature T in the E-PSO model).

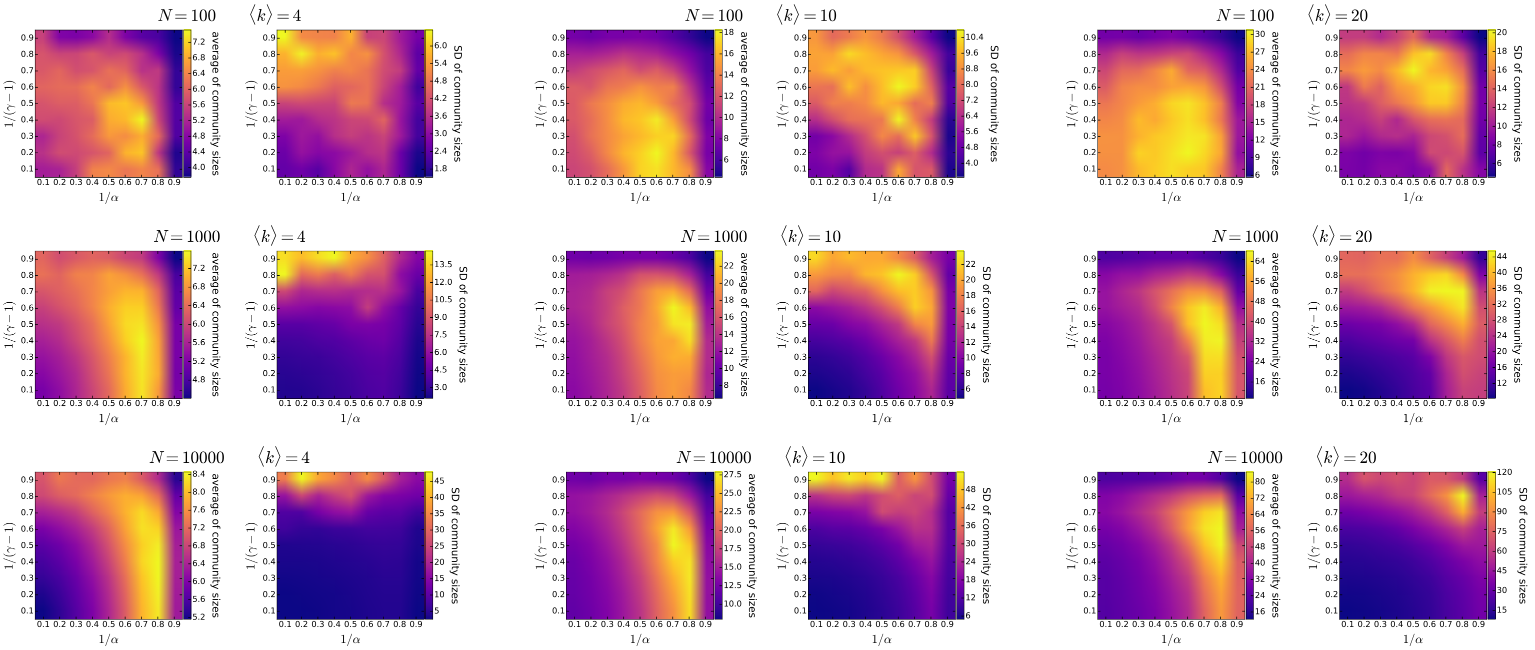

Figure F26. The mean and the standard deviation of the size of communities detected by the *Infomap* algorithm in 100 *unweighted* $\mathbb{S}^1/\mathbb{H}^2$ networks of different parametrisations. Each pair of subplots depicts the effect of changing $1/(\gamma-1)$ (equivalent to the popularity fading parameter β in the E-PSO model) and $1/\alpha$ (analogous to the temperature T in the E-PSO model), with the number of nodes N and the expected average degree $\langle k \rangle$ given in the title of the subplot pair. We used $K=-1$ as the curvature of the hyperbolic plane in each case.

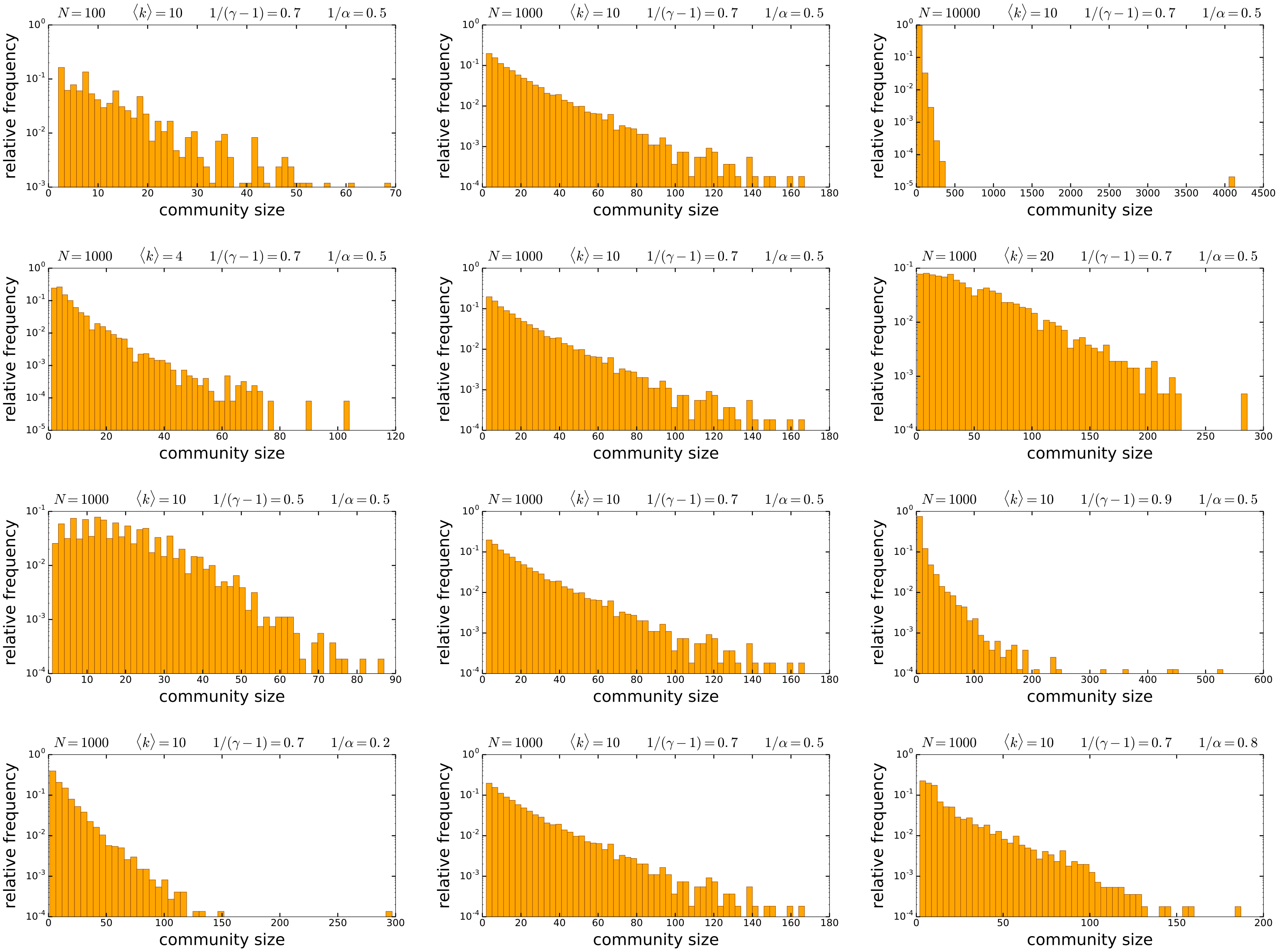

Figure F27. The size distribution of the communities detected by the *Infomap* algorithm in 100 *unweighted* $\mathbb{S}^1/\mathbb{H}^2$ networks of different parametrisations. The parameters of the network generation are listed in the title for each subplot. We used $K = -1$ as the curvature of the hyperbolic plane in each case. Each row of the figure demonstrates the effect of the change in a given network generation parameter: from top to bottom, the number of nodes N, the expected average degree $\langle k \rangle$, $1/(\gamma - 1)$ (equivalent to the popularity fading parameter β in the E-PSO model) and $1/\alpha$ (analogous to the temperature T in the E-PSO model).

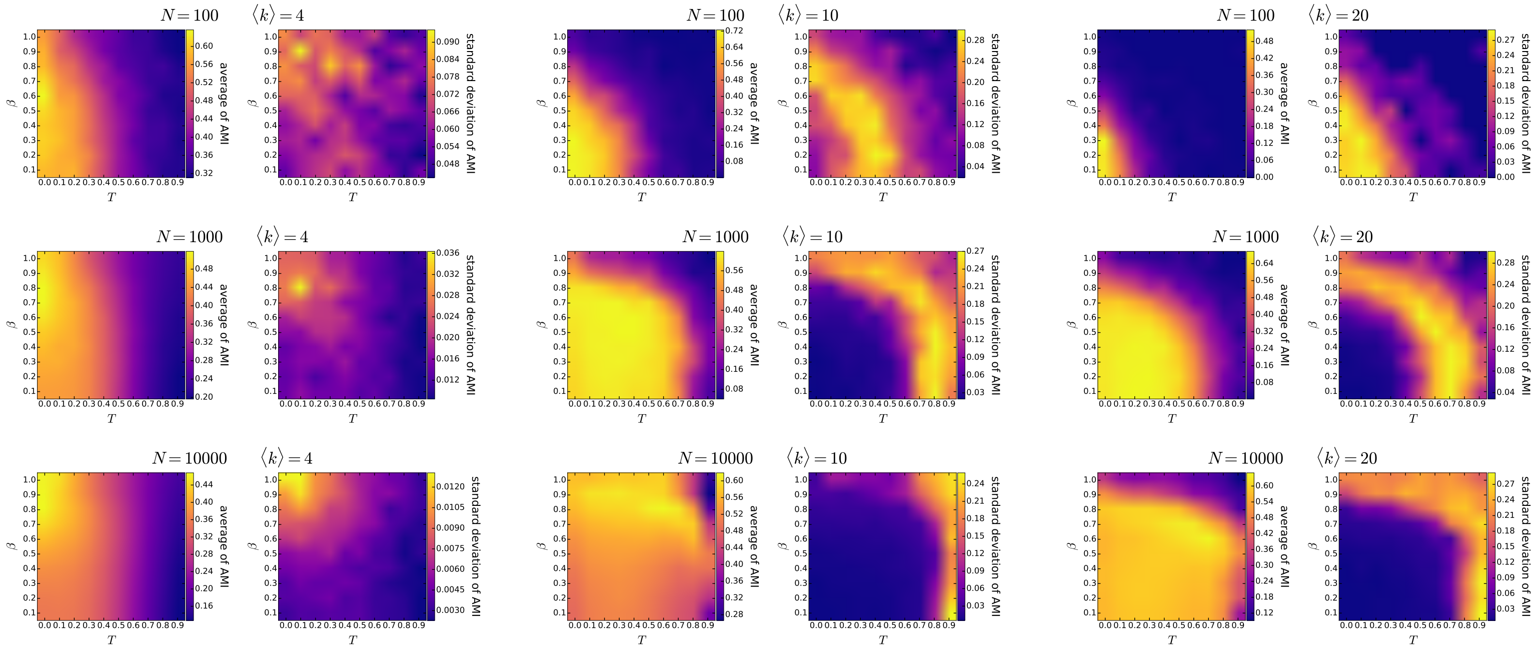

Figure F28. The mean and the standard deviation of the adjusted mutual information of the two community structures detected by the *asynchronous label propagation* and the *Louvain* algorithms in 100 *unweighted PSO* networks of different parametrisations. Each pair of subplots depicts the effect of changing the popularity fading parameter β and the temperature T, with the number of nodes N and the expected average degree $\langle k \rangle = 2m$ given in the title of the subplot pair. The curvature of the hyperbolic plane K was always set to -1, i.e. we used $\zeta = 1$.

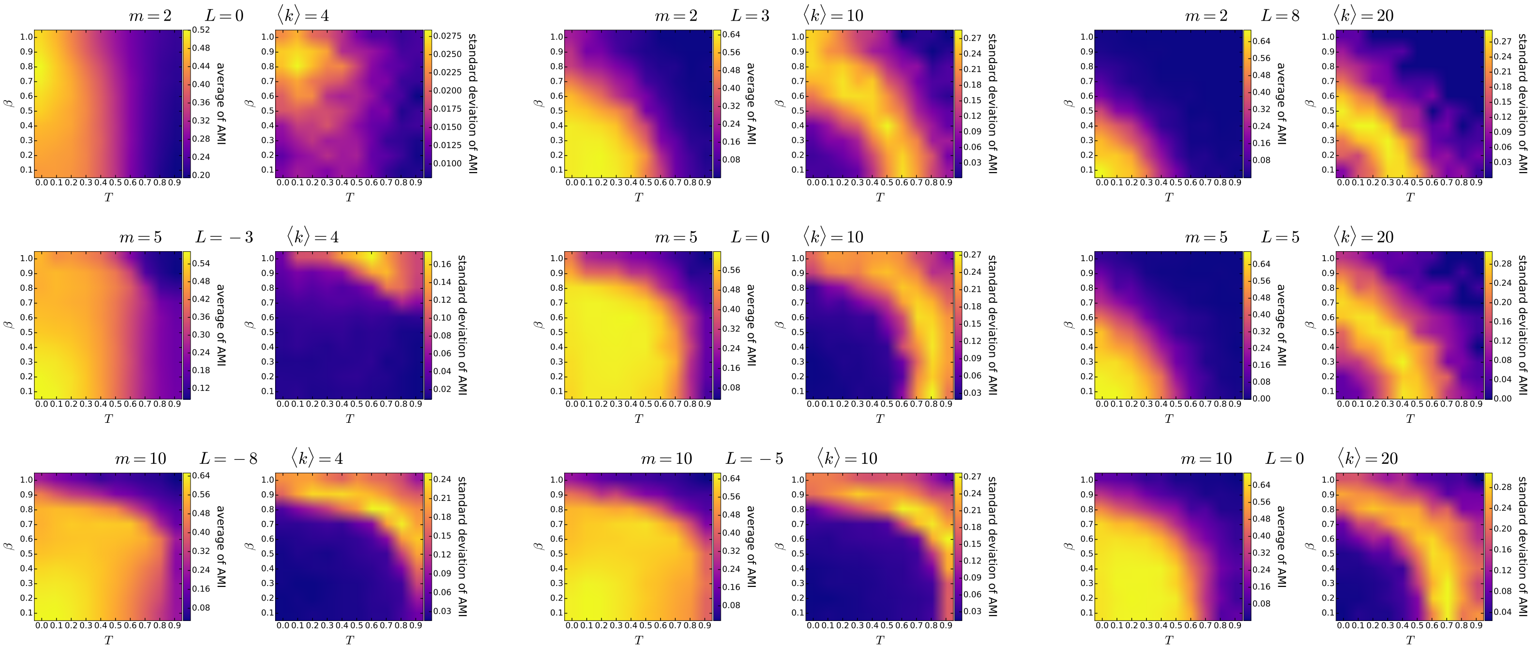

Figure F29. The mean and the standard deviation of the adjusted mutual information of the two community structures detected by the *asynchronous label propagation* and the *Louvain* algorithms in 100 *unweighted E-PSO* networks of different parametrisations. Each pair of subplots depicts the effect of changing the popularity fading parameter β and the temperature T, with the parameters m and L given in the title of the subplot pair together with the corresponding expected average degree $\langle k\rangle = 2(m+L)$. The number of nodes N was 1000 in each case. The curvature of the hyperbolic plane K was always set to -1, i.e. we used $\zeta = 1$.

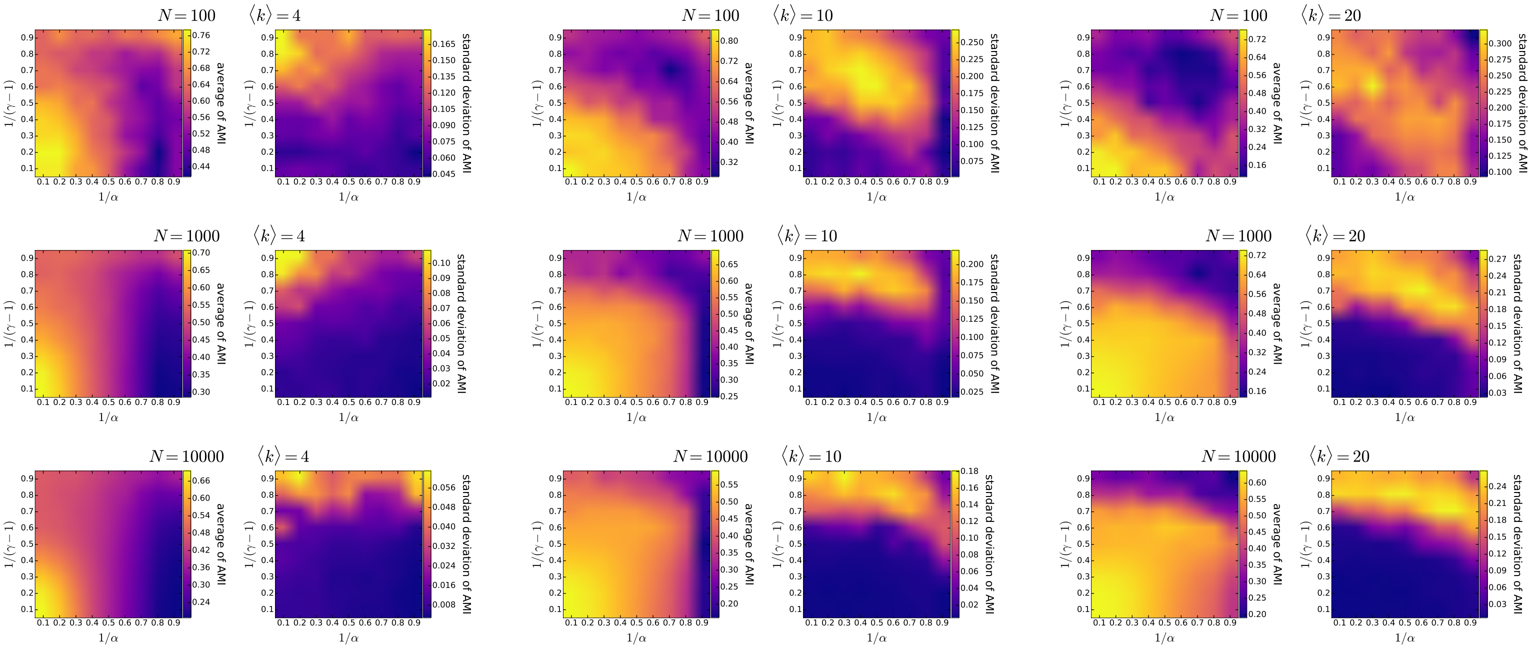

Figure F30. The mean and the standard deviation of the adjusted mutual information of the two community structures detected by the *asynchronous label propagation* and the *Louvain* algorithms in 100 *unweighted* $\mathbb{S}^1/\mathbb{H}^2$ networks of different parametrisations. Each pair of subplots depicts the effect of changing $1/(\gamma-1)$ (equivalent to the popularity fading parameter β in the E-PSO model) and $1/\alpha$ (analogous to the temperature T in the E-PSO model), with the number of nodes N and the expected average degree $\langle k\rangle$ given in the title of the subplot pair. We used $K=-1$ as the curvature of the hyperbolic plane in each case.

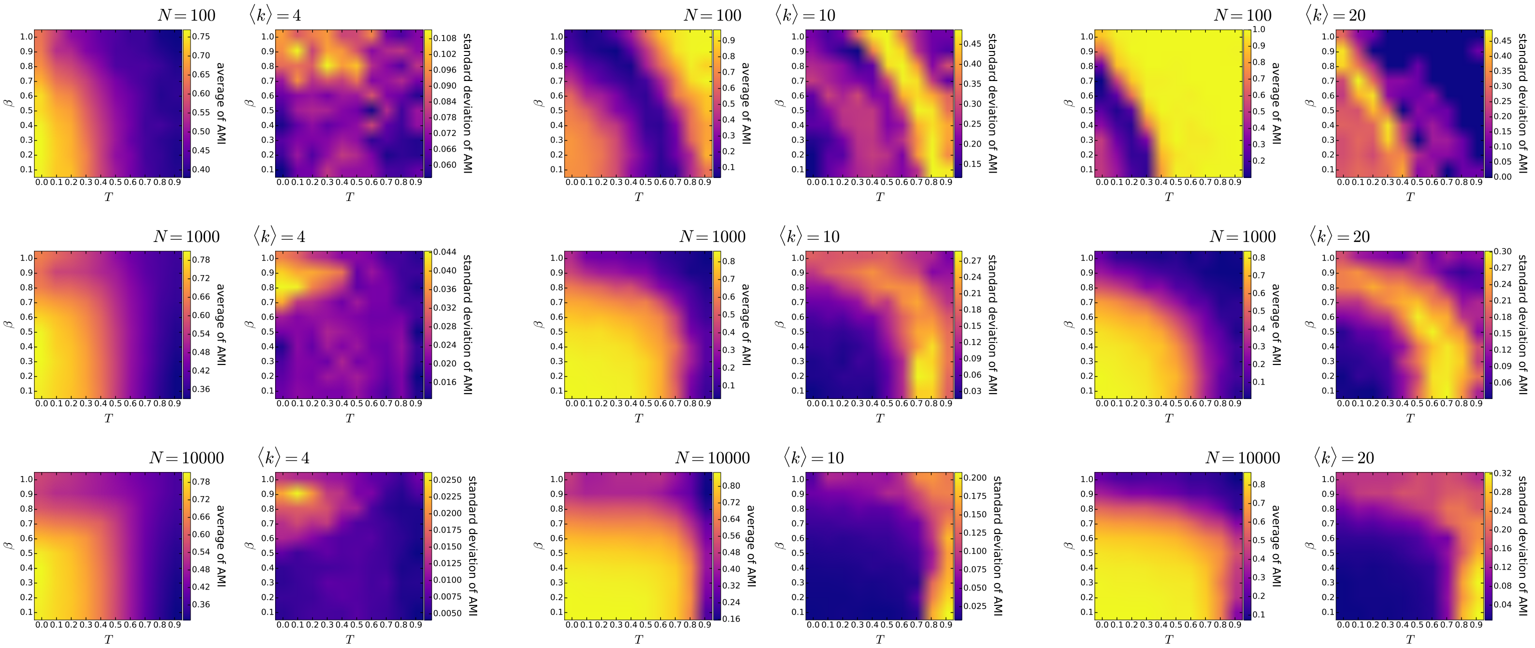

Figure F31. The mean and the standard deviation of the adjusted mutual information of the two community structures detected by the *asynchronous label propagation* and the *Infomap* algorithms in 100 *unweighted PSO* networks of different parametrisations. Each pair of subplots depicts the effect of changing the popularity fading parameter β and the temperature T, with the number of nodes N and the expected average degree $\langle k \rangle = 2m$ given in the title of the subplot pair. The curvature of the hyperbolic plane K was always set to -1, i.e. we used $\zeta = 1$.

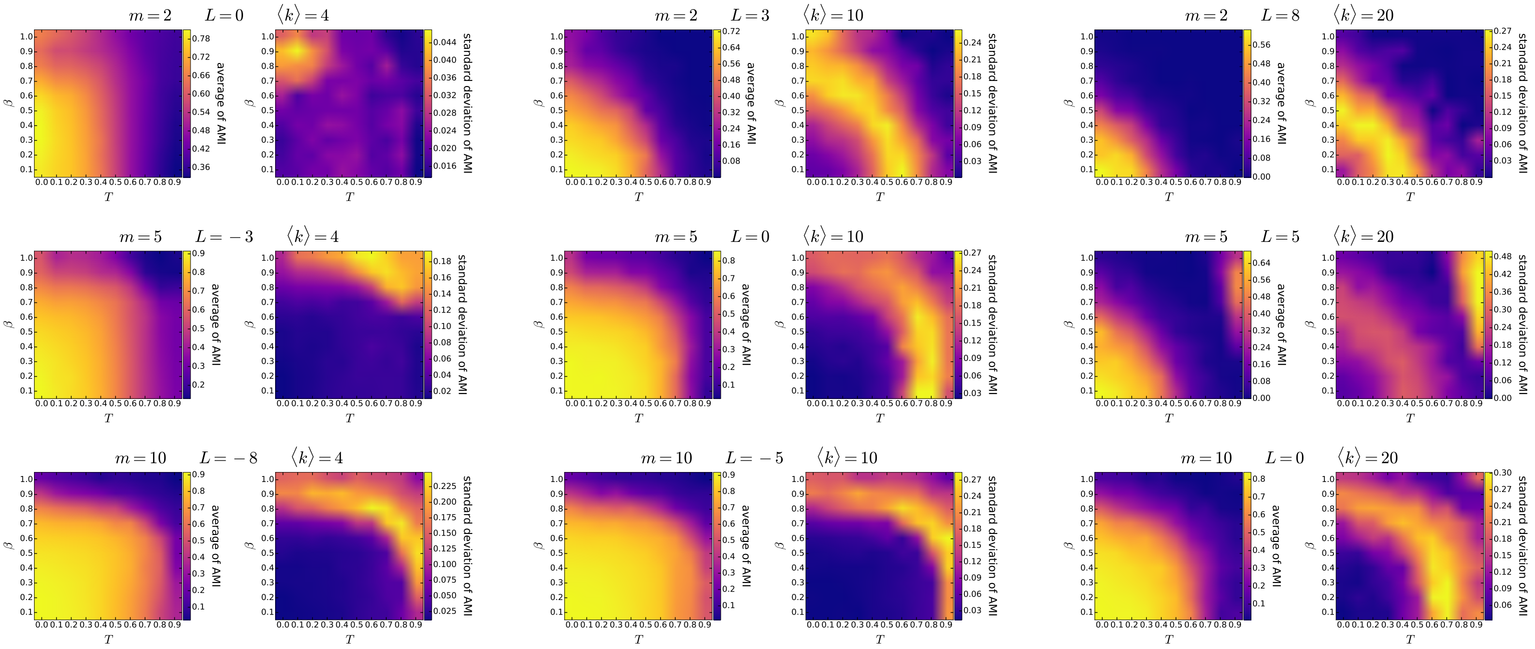

Figure F32. The mean and the standard deviation of the adjusted mutual information of the two community structures detected by the *asynchronous label propagation* and the *Infomap* algorithms in 100 *unweighted E-PSO* networks of different parametrisations. Each pair of subplots depicts the effect of changing the popularity fading parameter β and the temperature T, with the parameters m and L given in the title of the subplot pair together with the corresponding expected average degree $\langle k \rangle = 2(m+L)$. The number of nodes N was 1000 in each case. The curvature of the hyperbolic plane K was always set to -1, i.e. we used $\zeta = 1$.

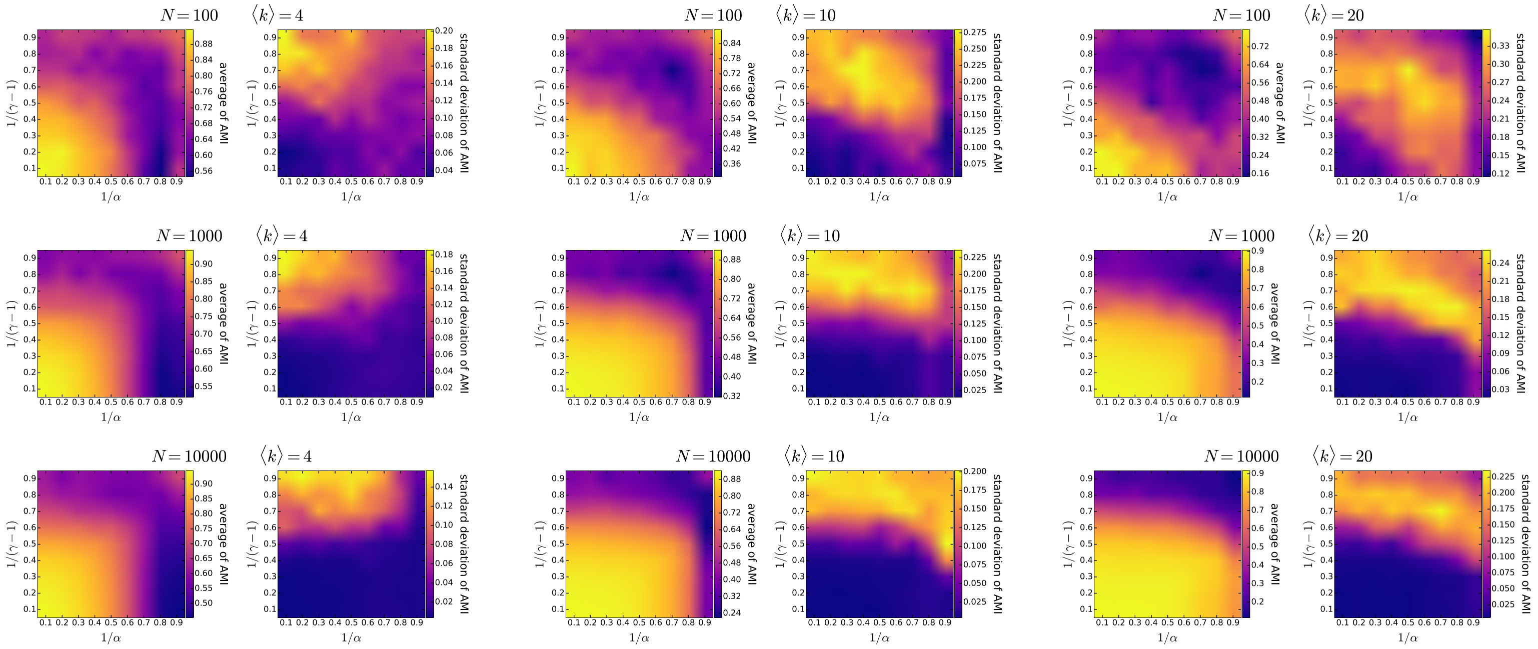

Figure F33. The mean and the standard deviation of the adjusted mutual information of the two community structures detected by the *asynchronous label propagation* and the *Infomap* algorithms in 100 *unweighted* $\mathbb{S}^1/\mathbb{H}^2$ networks of different parametrisations. Each pair of subplots depicts the effect of changing $1/(\gamma-1)$ (equivalent to the popularity fading parameter β in the E-PSO model) and $1/\alpha$ (analogous to the temperature T in the E-PSO model), with the number of nodes N and the expected average degree $\langle k \rangle$ given in the title of the subplot pair. We used $K=-1$ as the curvature of the hyperbolic plane in each case.

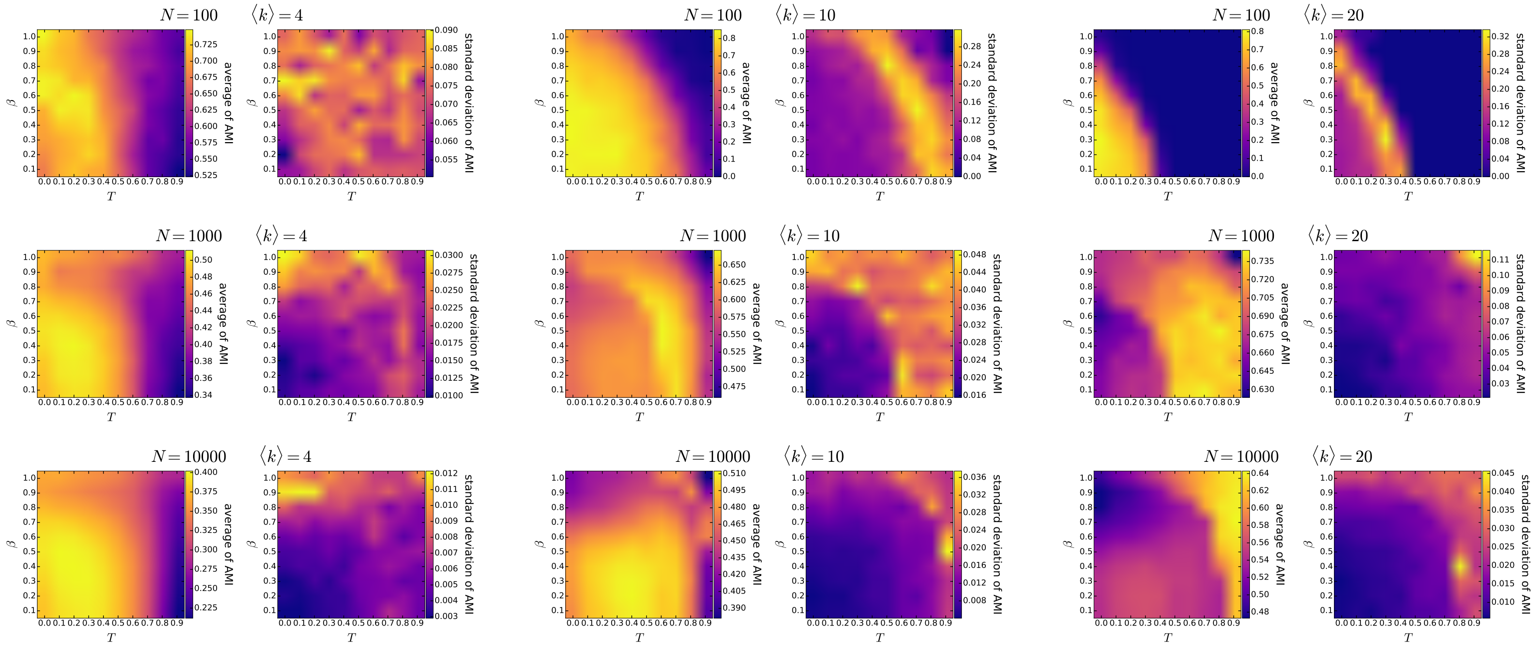

Figure F34. The mean and the standard deviation of the adjusted mutual information of the two community structures detected by the *Louvain* and the *Infomap* algorithms in 100 *unweighted PSO* networks of different parametrisations. Each pair of subplots depicts the effect of changing the popularity fading parameter β and the temperature T, with the number of nodes N and the expected average degree $\langle k \rangle = 2m$ given in the title of the subplot pair. The curvature of the hyperbolic plane K was always set to -1, i.e. we used $\zeta=1$.

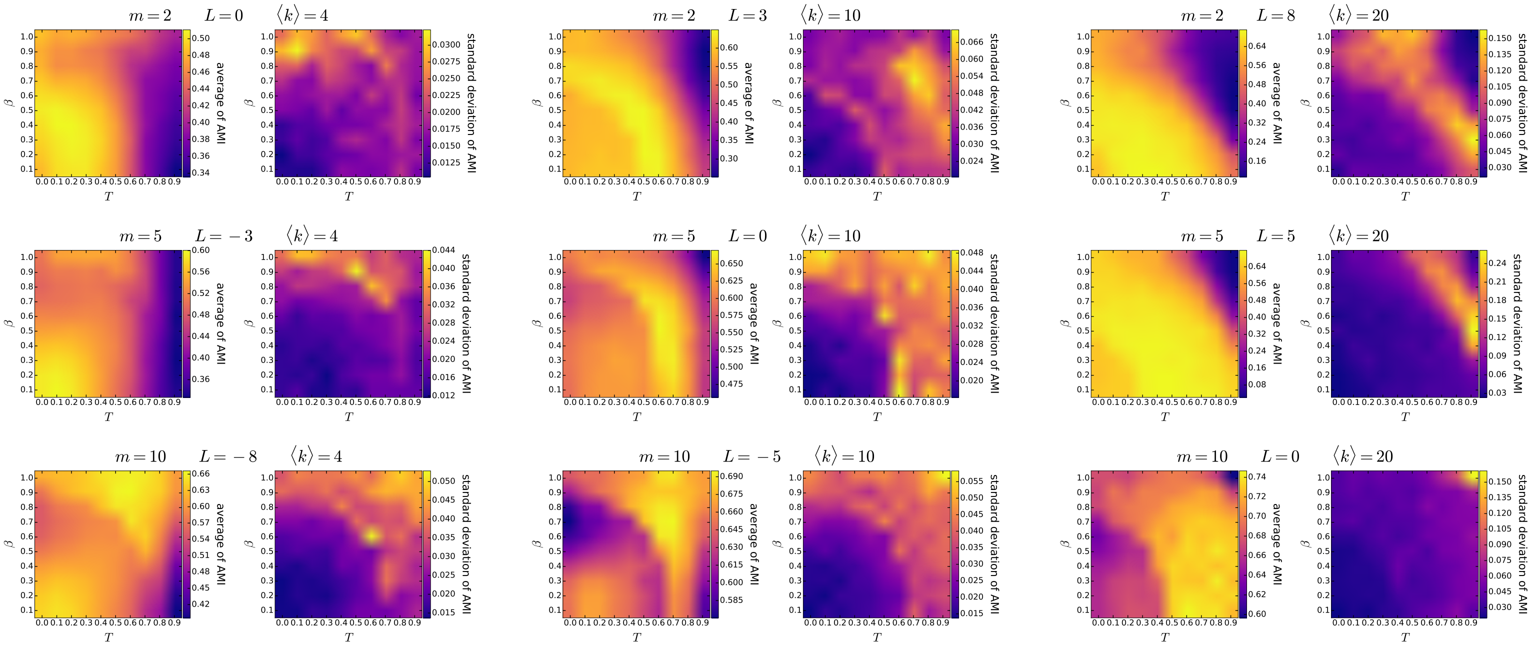

Figure F35. The mean and the standard deviation of the adjusted mutual information of the two community structures detected by the *Louvain* and the *Infomap* algorithms in 100 *unweighted E-PSO* networks of different parametrisations. Each pair of subplots depicts the effect of changing the popularity fading parameter β and the temperature T, with the parameters m and L given in the title of the subplot pair together with the corresponding expected average degree $\langle k \rangle = 2(m+L)$. The number of nodes N was 1000 in each case. The curvature of the hyperbolic plane K was always set to -1, i.e. we used $\zeta = 1$.

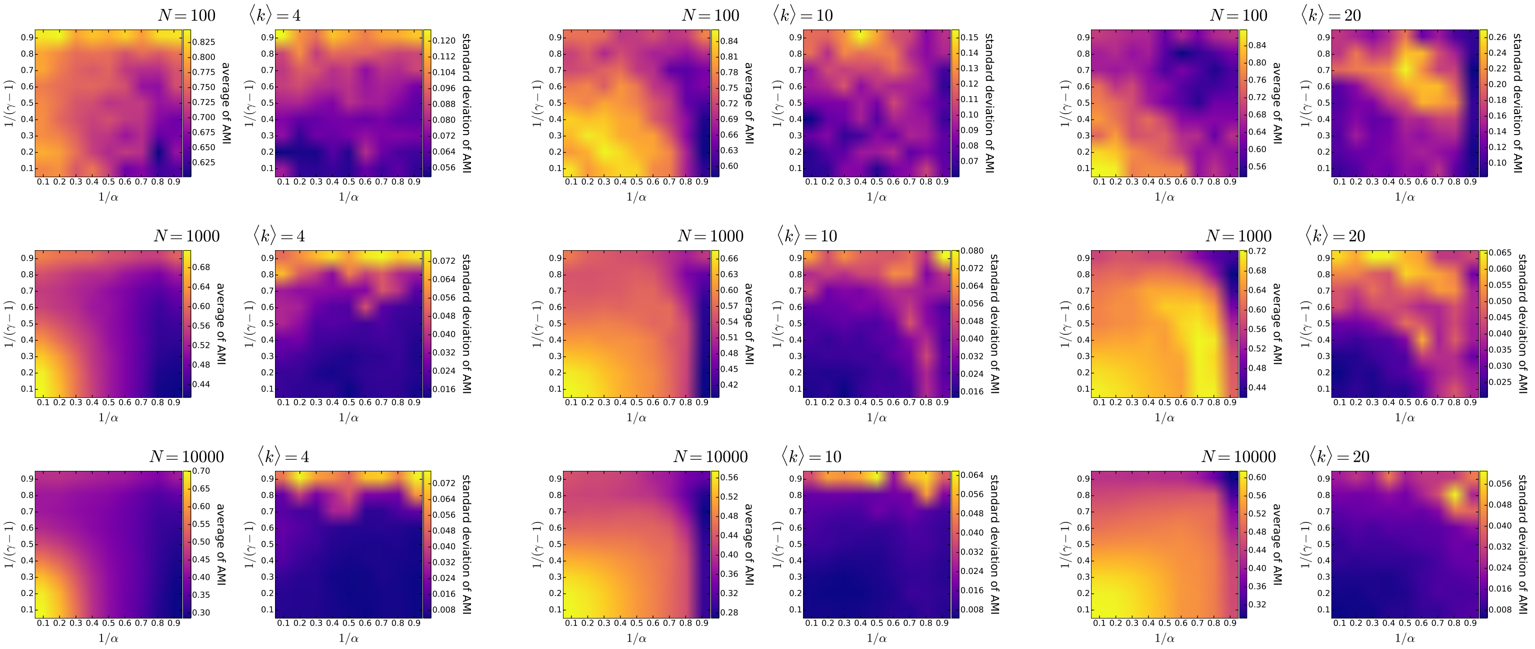

Figure F36. The mean and the standard deviation of the adjusted mutual information of the two community structures detected by the *Louvain* and the *Infomap* algorithms in 100 *unweighted* $\mathbb{S}^1/\mathbb{H}^2$ networks of different parametrisations. Each pair of subplots depicts the effect of changing $1/(\gamma-1)$ (equivalent to the popularity fading parameter β in the E-PSO model) and $1/\alpha$ (analogous to the temperature T in the E-PSO model), with the number of nodes N and the expected average degree $\langle k \rangle$ given in the title of the subplot pair. We used $K = -1$ as the curvature of the hyperbolic plane in each case.

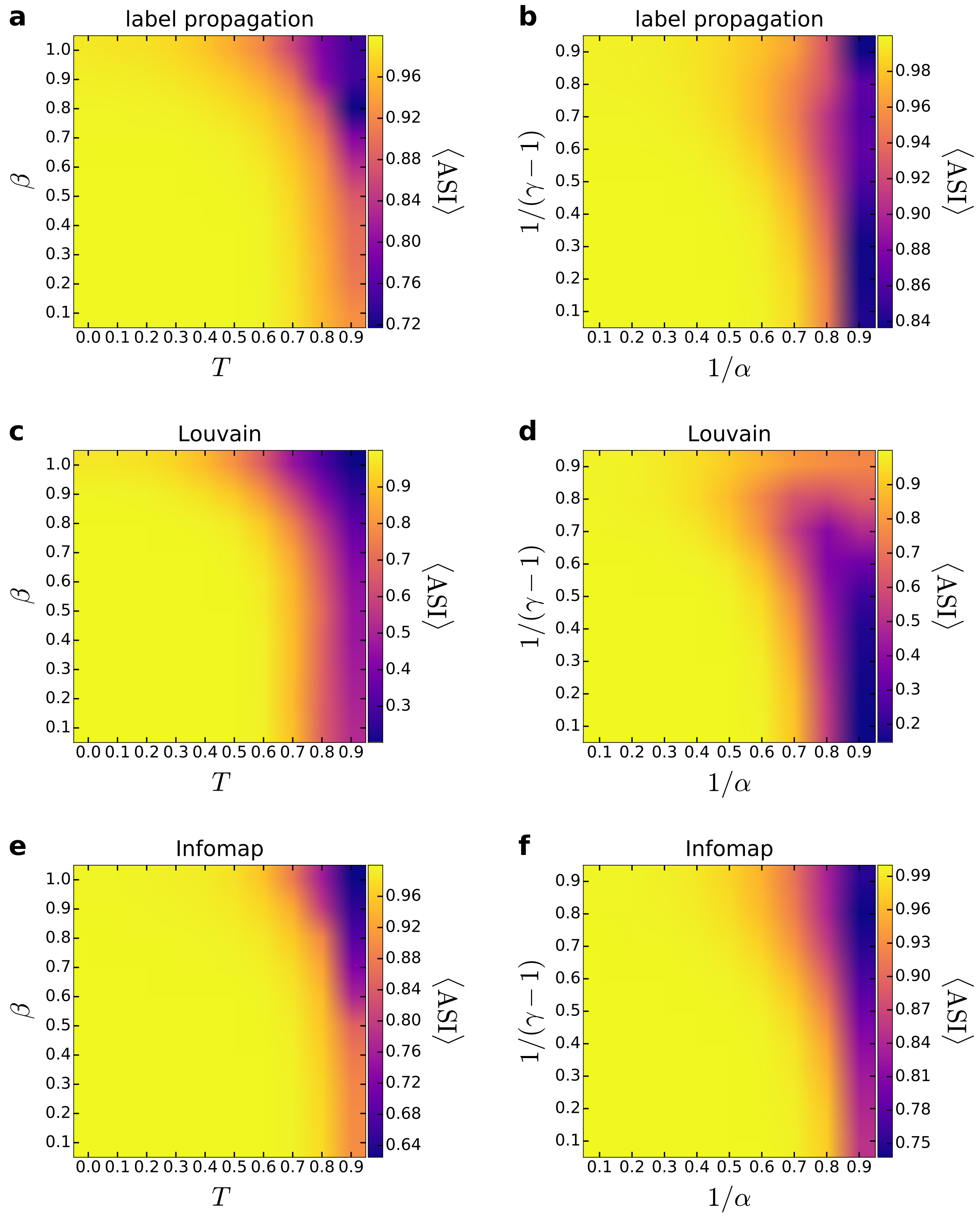

Figure F37. Angular separation index in the *unweighted* PSO and $\mathbb{S}^1/\mathbb{H}^2$ models. The results for the PSO model are given in the left column (panels (a), (c) and (e)), whereas the ASI obtained for the $\mathbb{S}^1/\mathbb{H}^2$ model appears in the right column (panels (b), (d) and (f)). The ASI for the communities detected by asynchronous label propagation is given in the top row (panels (a) and (b)), the ASI regarding the results of Louvain is shown in the middle row (panels (c) and (d)) and the ASI for the partitions found by Infomap is presented in the bottom row (panels (e) and (f)). We show the measured ASI (indicated by the color, averaged over 100 samples) as a function of the model parameters T and β, or $1/\alpha$ and $1/(\gamma-1)$ for networks of size $N = 10,000$ and expected average degree $\langle k \rangle = 10$.

References

1. Raghavan, U. N., Albert, R. & Kumara, S. Near linear time algorithm to detect community structures in large-scale networks. *Phys. Rev. E* **76**, 036106, DOI: 10.1103/PhysRevE.76.036106 (2007).
2. We used the python function ‘asyn_lpa_communities’, an implementation of the asynchronous label propagation algorithm available in the ‘networkx.algorithms.community.label_propagation’ package.
3. Blondel, V. D., Guillaume, J.-L., Lambiotte, R. & Lefebvre, E. Fast unfolding of communities in large networks. *J. Stat. Mech. Theory Exp.* **2008**, P10008, DOI: 10.1088/1742-5468/2008/10/p10008 (2008).
4. We used the python implementation of the louvain algorithm available at https://github.com/taynaud/python-louvain. (Accessed: 14/07/2020).
5. Rosvall, M. & Bergstrom, C. T. Multilevel compression of random walks on networks reveals hierarchical organization in large integrated systems. *PLOS ONE* **6**, 1–10, DOI: 10.1371/journal.pone.0018209 (2011).
6. We used the python package for the infomap algorithm available at https://pypi.org/project/infomap/. (Accessed: 14/07/2020).
7. Papadopoulos, F., Kitsak, M., Serrano, M. Á., Boguñá, M. & Krioukov, D. Popularity versus similarity in growing networks. *Nature* **489**, 537 EP –, DOI: 10.1038/nature11459 (2012).
8. Papadopoulos, F., Psomas, C. & Krioukov, D. Network mapping by replaying hyperbolic growth. *IEEE/ACM Transactions on Netw.* **23**, 198–211, DOI: 10.1109/TNET.2013.2294052 (2015).
9. Kovács, B. & Palla, G. Optimisation of the coalescent hyperbolic embedding of complex networks (2020). Preprint at https://arXiv:2009.04702 [cs.SI].
10. Serrano, M. A., Krioukov, D. & Boguñá, M. Self-similarity of complex networks and hidden metric spaces. *Phys. Rev. Lett.* **100**, 078701, DOI: 10.1103/PhysRevLett.100.078701 (2008).
11. García-Pérez, G., Allard, A., Serrano, M. Á. & Boguñá, M. Mercator: uncovering faithful hyperbolic embeddings of complex networks. *New J. Phys.* **21**, 123033, DOI: 10.1088/1367-2630/ab57d2 (2019).
12. We used the c++ implementation of the $\mathbb{S}^1/\mathbb{H}^2$ model available at https://github.com/networkgeometry/mercator. (Accessed: 14/07/2020).
13. Newman, M. E. J. & Girvan, M. Finding and evaluating community structure in networks. *Phys. Rev. E* **69**, 026113 (2004).
14. We calculated the modularity values with the python function ‘modularity’ available in the ‘networkx.algorithms.community.quality’ package.
15. Vinh, N. X., Epps, J. & Bailey, J. Information theoretic measures for clusterings comparison: Variants, properties, normalization and correction for chance. *J. Mach. Learn. Res.* **11**, 2837–2854 (2010).
16. McCarthy, A. D. & Matula, D. W. Normalized mutual information exaggerates community detection performance. In *SIAM Workshop on Network Science 2018*, 78–79 (2018).
17. We calculated the adjusted mutual information values with the python function ‘adjusted_mutual_info_score’ available in the ‘sklearn.metrics.cluster’ package.
18. Muscoloni, A. & Cannistraci, C. V. Angular separability of data clusters or network communities in geometrical space and its relevance to hyperbolic embedding (2019). Preprint at https://arXiv:1907.00025 [cs.LG].
